# Krishnolides A–D: New 2-Ketokhayanolides from the Krishna Mangrove, *Xylocarpus moluccensis*

**DOI:** 10.3390/md15110333

**Published:** 2017-10-27

**Authors:** Qun Zhang, Tirumani Satyanandamurty, Li Shen, Jun Wu

**Affiliations:** 1 Marine Drugs Research Center, College of Pharmacy, Jinan University, 601 Huangpu Avenue West, Guangzhou 510632, China; zhangqun0917@163.com; 2 Government Degree College at Amadala Valasa, Srikakulam District, Andhra Pradesh 532185, India; satyananda_murty@yahoo.com; 3 School of Pharmaceutical Sciences, Southern Medical University, 1838 Guangzhou Avenue North, Guangzhou 510515, China

**Keywords:** mangrove, *Xylocarpus moluccensis*, limonoid, khayanolide, anti-HIV

## Abstract

Four new khayanolide-type limonoids with a 2-carbonyl group, named krishnolides A–D (**1**–**4**), were isolated from the seeds of an Indian mangrove, *Xylocarpus moluccensis*, collected in the mangrove swamp of Krishna estuary, Andhra Pradesh. The relative and absolute configurations of these compounds were established by HR-ESIMS, extensive NMR investigations, single-crystal X-ray diffraction analysis with CuKα radiation, and experimental electronic circular dichroism (ECD) spectra. Krishnolides A–D are unusual khayanolides containing two large ester substituents of five or four carbon atoms at the C-3 and C-30 positions, respectively. Krishnolide A, containing an 8,14-epoxy group, exhibited moderate anti-Human Immunodeficiency Virus (HIV) activity with an IC_50_ value of 17.45 ± 1.65 μM and a CC_50_ value of 78.45 ± 1.69 μM, respectively. This is not only the first report of natural khayanolides from Indian mangroves of the genus *Xylocarpus*, but also the first report of the anti-HIV activity of khayanolide.

## 1. Introduction

**Figure 1:**
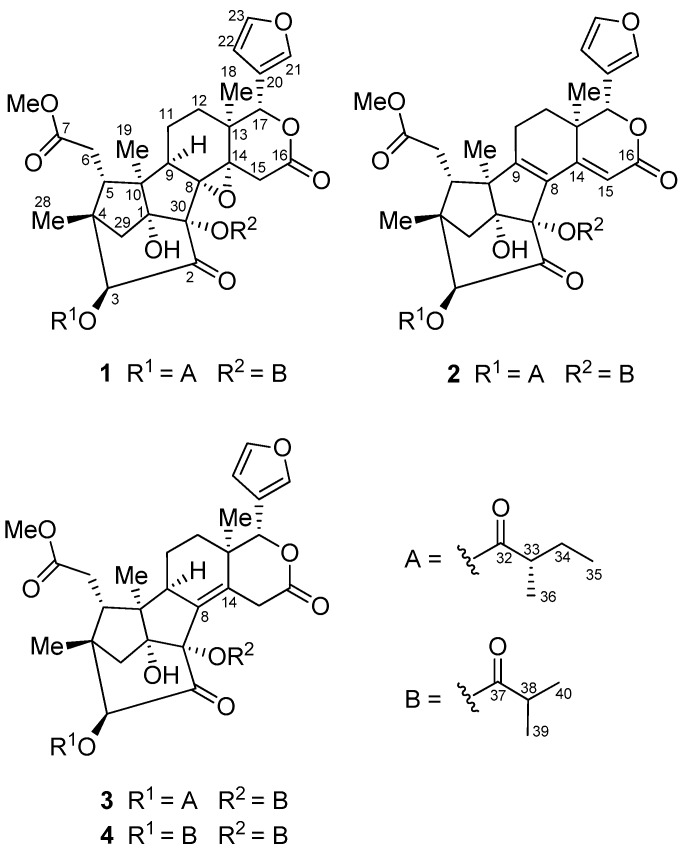
Structures of compounds **1**–**4**.

Plants of the genus *Xylocarpus*, belonging to the family Meliaceae, are mangroves mainly found in East Africa, India, Bangladesh, Southeast Asia, Southern China, and Northern Australia. Limonoids are the main secondary metabolites of these mangroves [1,2,3]. Previously, a variety of limonoids with abundant structural diversity and various bioactivities, such as antiviral and antitumor activities, were identified from seeds of the mangrove *X. moluccensis* [4,5,6,7]. Thaixylomolin I, a khayanolide-type limonoid obtained from the Thai *X. moluccensis*, exhibited stronger inhibitory activity against pandemic influenza A virus (subtype H1N1) than that of the antiviral drug, ribavirin [6]. Khayanolides are a small group of limonoids containing an octahydro-1*H*-1,6-methanoindene moiety as the ring A/B-fused carbotricyclic core. To date, only 36 khayanolides have been reported [8,9,10,11,12,13,14,15,16,17,18,19,20,21]. In order to search for new antiviral limonoids, seeds of the Indian *X. moluccensis*, collected in the mangrove swamp of Krishna estuary, Andhra Pradesh, were further investigated to afford four new khayanolides, named krishnolides A–D (**1**–**4**) (Figure 1). Herein, we report the isolation, structural elucidation, and anti-HIV activities of these khayanolides.

## 2. Results and Discussion

**Figure 2:**
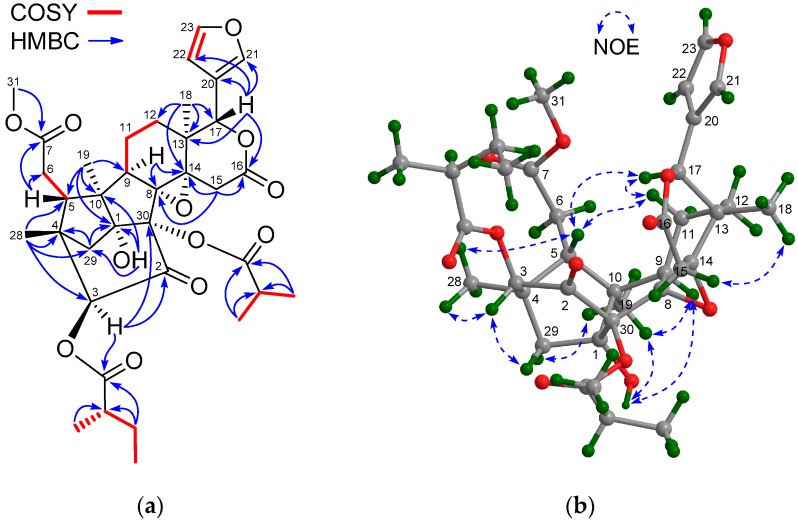
(**a**) Selected ^1^H-^1^H COSY and HMBC correlations for compound **1** (measured in DMSO-*d*_6_); (**b**) Diagnostic Nuclear Overhauser Effect (NOE) interactions for compound **1** (measured in DMSO-*d*_6_).

Compound **1** was obtained as a colorless crystal. Its molecular formula was established as C_36_H_46_O_12_ by the positive HR-ESIMS ion peak at *m*/*z* 693.2871 (calcd. for [M + Na]^+^ 671.2881), indicating 14 degrees of unsaturation. According to the ^1^H and ^13^C NMR spectroscopic data of **1** (Table 1 and Table 2), seven degrees of unsaturation were due to a keto carbonyl group, two carbon-carbon double bonds, and four ester functionalities; thus, the molecule was heptacyclic. The NMR spectroscopic data of **1** resembled those of thaixylomolin K [6], except for the presence of an 8,14-epoxy group (δ_C_ 73.6 (C-8), 65.1 (C-14)), a 3-(2-methyl)butyryloxy function (δ_H_ 2.35 (m), 1.46 (m), 1.55 (m), 0.80 (t, *J* = 7.2 Hz), 1.04 (d, *J* = 7.2 Hz); δ_C_ 175.2 (qC), 40.8 (CH), 26.7 (CH_2_), 11.6 (CH_3_), 16.3 (CH_3_)), and a 30-isobutyryloxy moiety (δ_H_ 2.63 (m), 1.09 (d, *J* = 7.2 Hz), 1.10 (d, *J* = 7.2 Hz); δ_C_ 175.6 (qC), 33.2 (CH_3_), 19.0 (CH_3_), 18.7 (CH)) (Table 1 and Table 2, recorded in DMSO-*d*_6_), and the absence of the Δ^8,14^ double bond, the 3-acetoxy group, and the C-30 methine moiety. HMBC cross-peaks between H-9/C-8, H-9/C-14, H_2_-15/C-8, H_2_-15/C-14, and H_3_-18/C-14 confirmed the existence of the 8,14-epoxy group, being corroborated by the degrees of unsaturation of **1**. Those from H-3 (δ 5.44 (s)) to the carbonyl carbon (δ_C_ 175.2 (C-32)) of the 2-methylbutyryloxy function placed it at C-3, whereas the location of an isobutyroxy moiety at C-30 was supported by the deshielded quaternary C-30 signal (δ 87.2) in **1**, as compared to that (δ 63.1) in thaixylomolin K. HMBC correlations from 1-OH (δ 5.86 (br s)) and H_2_-29 (δ 2.43 (1H, d, *J* = 12.6 Hz), 2.09 (1H, d, *J* = 12.6 Hz)) to C-30 (Figure 2a) demonstrated the above deduction.

The relative configuration of **1** was assigned by analysis of the Nuclear Overhauser Effect (NOE) interactions (Figure 2b), of which those between H-17/H-11β, H-11β/H-5, H-17/H-5, and H-5/H_3_-28 revealed their cofacial relationship and were arbitrarily assigned as the β-oriented H-5, H-17, and H_3_-28. In turn, those between H_3_-18/H-15α, H_3_-19/H-9, H_3_-19/1-OH, and H-9/1-OH assigned the α-orientation for H_3_-18, H_3_-19, H-9, and 1-OH. The significant NOE interaction from H-3 to H*_pro-R_*-29 (δ 2.43 (d, *J* = 12.6 Hz)), but not from H-3 to H-5, established 3α-H and the corresponding 3β-(2-methyl)butyryloxy function. However, the orientation of the 8,14-epoxy group could not be determined by NOE interactions.

**Figure 3:**
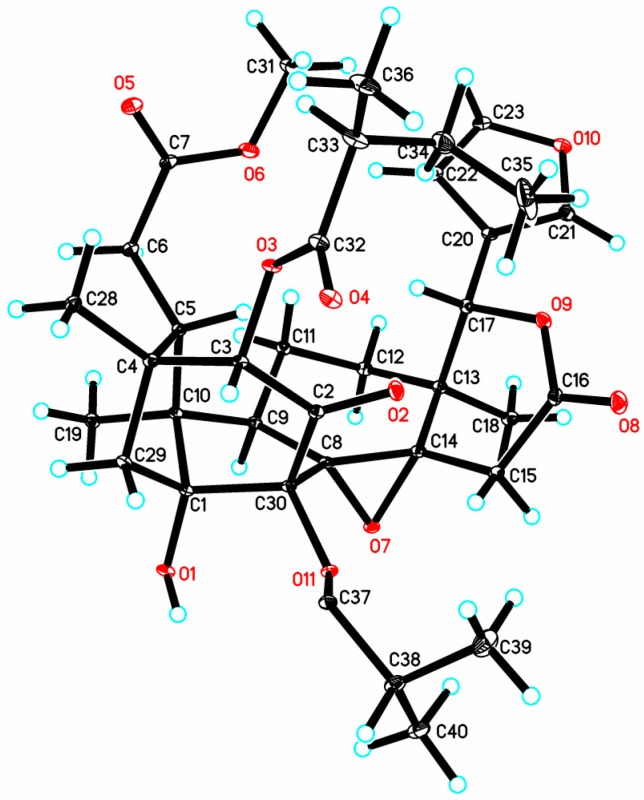
Oak Ridge Thermal-Ellipsoid Plot Program (ORTEP) illustration of the X-ray structure of compound **1**. Ellipsoids are given at the 30% probability level.

After considerable effort, suitable crystals of **1** were obtained in acetone/n-hexane (4:1) at room temperature. Thus, single-crystal X-ray diffraction analysis, conducted with CuKα radiation, was carried out to establish the relative and absolute configurations of **1**. The absolute configuration of **1**, named krishnolide A, was unequivocally established as 1*R*,3*S*,4*R*,5*S*,8*R*,9*R*,10*S*,13*S*,14*S*,17*S*,30*R*,33*S* (Flack parameter of −0.06 (10), Flack x of −0.00(9), and Hooft y of −0.00(4)) [22]. Computer-generated perspective drawings of the X-ray structure of **1** are shown in Figure 3 and Figure S1.

**Figure 4:**
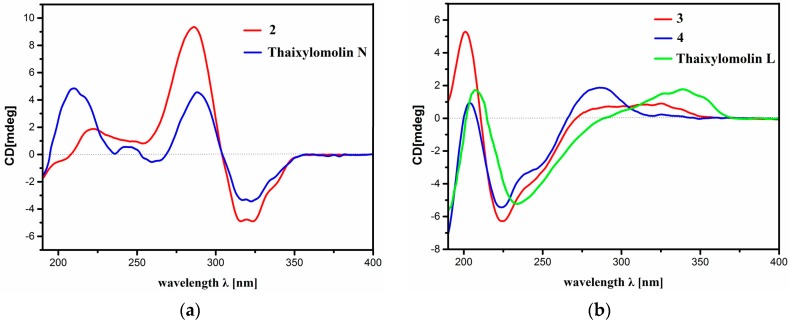
(**a**) Comparison of the experimental circular dichroism (CD) spectra of compound **2** and the known compound, thaixylomolin N, containing conjugated Δ^8,9^ and Δ^14,15^ double bonds; (**b**) Comparison of the experimental CD spectra of compounds **3**, **4**, and the known compound, thaixylomolin L, containing a Δ^8,9^ double bond.

Compound **2**, a white amorphous powder, had the molecular formula C_36_H_44_O_11_ as established by the positive HR-ESIMS ion peak at *m*/*z* 653.2956 (calcd. for [M + H]^+^ 653.2956). The ^1^H and ^13^C NMR spectroscopic data of **2** (Table 1 and Table 2) were similar to those of **1** except for the replacement of the 8,14-epoxy group in **1** by conjugated Δ^8,9^ and Δ^14,15^ double bonds (δ_C_ 124.6 (C-8, qC), 161.9 (C-9, qC), 151.7 (C-14, qC), 113.8 (C-15, CH)), which was corroborated by HMBC correlations between H-15/C-8, H-15/C-13, H-15/C-14, H-15/C-16, H_3_-18/C-14, and H_3_-19/C-9. The relative configuration of **2** was determined to be the same as that of **1** by NOE interactions between H-17/H-12β, H-5/H-11β, H-5/H_3_-28, H-19/H*_pro-S_*-29, and those between H_3_-18/H-11α, H-3/H*_pro-R_*-29. The absolute configuration of **2**, named krishnolide B, was established to be the same as that of thaixylomolin N (1*R*,3*S*,4*R*,5*S*,10*S*,13*R*,17*R*,30*S*-configured) by the similarity of their electronic circular dichroism (ECD) spectra (Figure 4a) [6].

Compound **3** provided the molecular formula C_36_H_46_O_11_, as established by the positive HR-ESIMS ion peak at *m*/*z* 677.2917 (calcd. for [M + Na]^+^ 677.2932). The NMR spectroscopic data of **3** (Table 1 and Table 2) displayed structural similarity to that of **1**, except for the replacement of the 8,14-epoxy group in **1** by a Δ^8,^^14^ double bond (δ_C_ 132.2 (C-8, qC), 139.4 (C-14, qC)) in **3**. ^1^H-^1^H COSY homoallylic couplings between H_2_-15/H-9 and HMBC correlations between H_2_-15/C-8, H_2_-15/C-14, H_3_-18/C-14, H-17/C-14, and H-9/C-8 confirmed the existence of the Δ^8,^^14^ double bond. The relative configuration of **3** was deduced to be identical to that of **1** by NOE interactions between H-17/H-12β, H-11β/H-5, H-17/H-5, H-5/H_3_-28, and those between H_3_-18/H-15α, H_3_-18/H-9, H_3_-19/H-9, H-3/H*_pro-R_* -29. The absolute configuration of **3**, named krishnolide C, was established to be the same as that of thaixylomolin L (1*R*,3*S*,4*R*,5*S*,9*R*,10*S*,13*R*,17*R*,30*S*-configured) by the similarity of their ECD spectra (Figure 4b) [6].

The molecular formula of **4** was assigned as C_35_H_44_O_11_ by the positive HR-ESIMS ion peak at *m*/*z* 663.2771 (calcd. for [M + Na]^+^ 663.2776). The NMR spectroscopic data of **4** (Table 1 and Table 2) were similar to those of **3**, except for the replacement of the 3-(2-methyl)butyryloxy group in **3** by an isobutyryloxy moiety (δ_H_ 2.65 (1H, m, H-33), 1.19 (3H, d, *J* = 7.2 Hz, H_3_-34), 1.20 (3H, d, *J* = 7.2 Hz, H_3_-35); δ_C_ 176.0 (C-32, qC), 33.8 (C-33, CH), 18.5 (C-34, CH_3_), 18.9 (C-35, CH_3_)) in **4**. The existence of the isobutyryloxy group was corroborated by ^1^H-^1^H COSY cross-peaks between H-33/H_3_-34 and H-33/H_3_-35, and HMBC correlations between H-33/C-32, H_3_-34/C-32, and H_3_-35/C-32. The strong HMBC correlation from H-3 (δ 5.21, s) to the carbonyl carbon (δ 176.0 (C-32)) of the isobutyryloxy group confirmed its location at C-3. The relative configuration of **4** was determined to be the same as that of **3** based on NOE interactions between H-17/H-12β, H-11β/H-5, H-5/H_3_-28, and those between H_3_-18/H-15α, H_3_-18/H-9, H_3_-19/H-9, H-3/H*_pro-R_*-29. The absolute configuration of **4**, named krishnolide D, was established to be the same as that of **3** (1*R*,3*S*,4*R*,5*S*,9*R*,10*S*,13*R*,17*R*,30*S*-configured) by the similarity of their ECD spectra (Figure 4b) [6].

Anti-HIV activities of **1**–**4** were tested in vitro by HIV-I virus-transfected 293 T cells [23]. At the concentration of 20 μM, only **1** showed a strong inhibitory rate of 79.75 ± 0.77%. Efavirenz was used as the positive control, with an inhibitory rate of 88.54 ± 0.45% at the same concentration. Values of IC_50_ and CC_50_ for **1** were 17.45 ± 1.65 and 78.45 ± 1.69 μM, respectively. Structure-activity relationship analyses of **1**–**4** concluded that the 8,14-epoxy group is pivotal for the anti-HIV activity of khayanolides. However, only two other khayanolides with an 8,14-epoxy group, viz. khayanolide A and 1-*O*-acetylkhayanolide A, have hitherto been reported [9,12].

Previously, 36 khayanolides were reported. These khayanolides exhibited antifeedant, anti-acetylcholinesterase, anti-butyrycholinesterase, anti-lipoxygenase, antitumor, antimicrobial, and anti-H1N1 (antiviral) activities [8,9,10,11,12,13,14,15,16,17,18,19,20,21], but no khayanolide has previously been reported to have anti-HIV activity. In this paper, the anti-HIV activities of khayanolides were reported for the first time.

## 3. Materials and Methods 

### 3.1. General Methods

Optical rotations were determined with an MCP 200 modular circular polarimeter (Anton Paar Opto Tec GmbH, Seelze, Germany). UV spectra were recorded on a GENESYS 10S UV-Vis spectrophotometer (Thermo Fisher Scientific, Shanghai, China), and HR-ESIMS was obtained on an LC-ESIMS system in the positive-ion mode (Bruker Daltonics, Bremen, Germany). NMR spectra were measured on a Bruker AV-400 NMR spectrometer. Preparative HPLC was carried out on C18 reversed-phase columns (YMC, 250 × 10 mm i.d., 5 μm) using a Waters 2535 pump equipped with a Waters 2489 UV detector (Waters Corporation, Milford, MA, USA). Single-crystal X-ray diffraction analysis of **1** was performed on an Agilent Xcalibur Atlas Gemini Ultra-diffractometer with mirror monochromated CuKα radiation (λ = 1.54184 Å) at 150 K. Silica gel (100–200 mesh) (Qingdao Mar. Chem. Ind. Co. Ltd., Qingdao, China) and C18 reversed-phase silica gel (50 μm, YMC Co. Ltd., Kyoto, Japan) were used for column chromatography.

### 3.2. Plant Material

Seeds of *Xylocarpus moluccensis* were collected in September 2007 at the mangrove swamp of Krishna estuary, Andhra Pradesh, India. Identification of the mangrove was done by one of the authors (T.S.). A voucher sample (No. IXM200701) is maintained in Marine Drugs Research Center, College of Pharmacy, Jinan University.

### 3.3. Extraction and Isolation

The air-dried seeds (6.0 kg) of *X. moluccensis* were powdered and extracted with 95% EtOH (6 × 15 L) at room temperature to afford the resulting extract (824.6 g), which was suspended in water (2.5 L) and extracted with EtOAc (6 × 7.5 L) to afford the EtOAc portion (299.1 g). The EtOAc portion was chromatographed on a silica gel column (150 × 8.5 cm i.d.), eluted with a gradient mixture of CHCl_3_/MeOH (100:0 to 5:1, *v*/*v*) to afford 223 fractions. Fractions 68–71 (22.9 g) were combined and further purified by C18 reversed-phase silica gel column chromatography (60 × 3 cm i.d.), and eluted with a gradient mixture of acetone/H_2_O (40:60 to 100:0, *v*/*v*) to yield 111 subfractions, among which subfractions 63–65 (351.2 mg) were combined and purified by preparative HPLC (MeCN/H_2_O, 55:45) to give compounds **1** (t*_R_* = 51.5 min, 20.2 mg) and **2** (t*_R_* = 46.6 min, 4.4 mg). Fractions 58–67 (43.0 g) were combined and separated by C18 reversed-phase silica gel column chromatography (58 × 5.5 cm i.d.), and eluted with a gradient mixture of acetone/H_2_O (40:60 to 100:0, *v*/*v*) to give 136 subfractions, among which the subfraction 98 (1.5 g) was subjected to preparative HPLC (MeCN/H_2_O, 55:45) to yield compound **3** (t*_R_* = 45.5 min, 7.1 mg), whereas the subfraction 100 (820.2 mg) was purified by preparative HPLC (MeOH/H_2_O, 65:35) to give compound **4** (t*_R_* = 48.3 min, 2.5 mg).

Krishnolide A (**1**). Colorless crystal, ${\left[a\right]}_{D}^{25}$ = −48 (*c* 0.1, acetone); UV (MeCN) λ_max_ (logε) 208 (3.80) nm; ECD (*c* 1.49 mM, MeCN) λ_max_ (Δε) 195 (−0.4), 208 (−0.5), 229 (−0.8), 326 (+0.5) nm; HR-ESIMS *m*/*z* 693.2871 [M + Na]^+^ (calcd. for C_36_H_46_NaO_12_, 693.2887); ^1^H and ^13^C NMR spectroscopic data see Table 1 and Table 2.

Krishnolide B (**2**). White, amorphous solid, ${\left[a\right]}_{D}^{25}$ = +36 (*c* 0.1, acetone); UV (MeCN) λ_max_ (logε) 207 (3.81), 282 (3.99) nm; ECD (*c* 0.19 mM, MeCN) λ_max_ (Δε) 222 (+3.0), 286 (+14.8), 324 (−7.7) nm; HR-ESIMS *m*/*z* 653.2956 [M + H]^+^ (calcd. for C_36_H_45_O_11_, 653.2962); ^1^H and ^13^C NMR spectroscopic data see Table 1 and Table 2.

Krishnolide C (**3**). White, amorphous solid, ${\left[a\right]}_{D}^{25}$ = −38 (*c* 0.1, acetone); UV (MeCN) λ_max_ (logε) 206 (3.84) nm; ECD (*c* 0.38 mM, MeCN) λ_max_ (Δε) 201 (+4.2), 225 (−5.0), 325 (+0.7) nm; HR-ESIMS *m*/*z* 677.2917 [M + Na]^+^ (calcd. for C_36_H_46_NaO_11_, 677.2932); ^1^H and ^13^C NMR spectroscopic data see Table 1 and Table 2.

Krishnolide D (**4**). White, amorphous solid, ${\left[a\right]}_{D}^{25}$ = −54 (*c* 0.1, acetone); UV (MeCN) λ_max_ (logε) 204 (4.35) nm; ECD (*c* 0.39 mM, MeCN) λ_max_ (Δε) 204 (+0.7), 224 (−4.2), 286 (+1.5) nm; HR-ESIMS *m*/*z* 663.2771 [M + Na]^+^ (calcd. for C_35_H_44_NaO_11_, 663.2776); ^1^H and ^13^C NMR spectroscopic data see Table 1 and Table 2.

### 3.4. X-ray Crystal Data for Krishnolide A *(**1**)*

Monoclinic, C_36_H_46_O_12_, space group *P*2(1), a = 9.33350 (10) Å, b = 18.00600 (10) Å, c = 10.73330 (10) Å, α = 90°, β = 113.9990 (10)°, γ = 90°, V = 1647.89 (3) Å^3^, Z = 2, D_calcd._ = 1.352 Mg/m^3^, μ = 0.839 mm^−1^. Crystal size: 0.40 × 0.34 × 0.30 mm^3^. 5929 measured reflections, 5810 (*R*_int_ = 0.0350) independent reflections, 442 parameters, one restraints, *F*(000) = 716, *R*_1_ = 0.0312, *wR*_2_ = 0.0800 (all data), *R*_1_ = 0.0304, *wR*_2_ = 0.0789 (*I* > 2σ(*I*)), and goodness-of-fit (*F*^2^) = 1.048. The absolute structural parameter is −0.06(10), the Flack x parameter is −0.00(9), and the value of Hooft y is −0.00(4).

CCDC-1573579 (**1**) contains the supplementary crystallographic data for this paper (excluding structure factors). These data are provided free of charge by The Cambridge Crystallographic Data Centre.

### 3.5. HIV-Inhibitory Bioassay

A total of 293 T cells (2 × 10^5^) were co-transfected with 0.6 μg of pNL-Luv-E^−^-Vpu^−^ and 0.4 μg of pHIT/G. After 48 h, the VSV-G pseudotyped viral supernatant (HIV-1) was harvested by filtration through a 0.45-μm filter and the concentration of viral capsid protein was determined by p24 antigen capture ELISA (Biomerieux). SupT1 cells were exposed to VSV-G pseudotyped HIV-1 (MOI = 1) at 37 °C for 48 h in the absence or presence of the test compounds **1**–**4** (Efavirenz was used as the positive control). The inhibition rates were determined by using a firefly Luciferase Assay System (Promega) [23].

## 4. Conclusions

In conclusion, four new khayanolides, containing a 2-methylbutyryloxy or an isobutyroxy substituent at the C-3 and C-30 positions, respectively, were first obtained from the seeds of an Indian mangrove, *Xylocarpus moluccensis*. The relative and absolute configurations of these compounds were established by HR-ESIMS, extensive NMR investigations, single-crystal X-ray diffraction analysis with CuKα radiation, and experimental electronic circular dichroism (ECD) spectra. Krishnolide A exhibited moderate anti-HIV activity. The 8,14-epoxy group is pivotal for its anti-HIV activity. This is the first anti-HIV report of khayanolides.

## Figures and Tables

**Table 1:** ^1^H (400 MHz) NMR spectroscopic data of compounds **1**–**4** (δ in ppm, *J* in Hz).

Position	1 ^a^	1 ^b^	2 ^a^	3 ^a^	4 ^a^
3	5.68 s	5.44 s	5.51 s	5.22 s	5.21 s
5	2.37 m	2.16 br d (8.0)	2.63 m	2.49 ^c^	2.48 ^c^
6a	2.22 br d (14.0)	2.36 ^c^	2.41 ^c^	2.30 br d (12.8)	2.32 br d (12.8)
6b	2.43 br d (14.0)	2.42 m	2.41 ^c^	2.47 ^c^	2.48 ^c^
9	2.07 m	1.97 m		2.58 m	2.59 m
11α	1.36 ^c^	1.41 ^c^	2.20 m	1.77 m	1.78 m
11β	1.36 ^c^	1.19 m	2.50 m	1.50 ^c^	1.50 ^c^
12α	1.28 m	1.13 m	1.59 m	1.44 m	1.44 m
12β	1.34 ^c^	1.17 ^c^	1.45 m	1.49 m	1.50 ^c^
15α	3.06 d (19.0)	3.15 d (19.0)	6.45 br s	3.62 dd (20.0, 3.8)	3.62 dd (20.0, 3.8)
15β	3.28 d (19.0)	3.00 d (19.0)		3.73 dd (20.0, 3.2)	3.74 dd (20.0, 3.2)
17	5.25 s	5.06 s	5.08 s	5.10 s	5.13 s
18	1.13 s	1.02 s	1.05 s	1.05 s	1.05 s
19	1.08 s	0.98 s	1.04 s	1.07 s	1.07 s
21	7.59 br s	7.69 br s	7.47 br s	7.48 br s	7.47 br s
22	6.45 br s	6.52 br s	6.45 br s	6.43 br s	6.42 br s
23	7.43 br s	7.75 br s	7.43 br s	7.43 br s	7.42 br s
28	1.05 s	0.97 s	1.04 s	0.98 s	0.98 s
29*_pro-S_*	2.13 d (12.6)	2.09 d (12.6)	1.97 d (12.6)	2.03 d (12.8)	2.03 d (12.8)
29*_pro-R_*	2.62 d (12.6)	2.43 d (12.6)	2.65 d (12.6)	2.50 d (12.8)	2.50 d (12.8)
7-OMe-31	3.70 s	3.63 s	3.70 s	3.66 s	3.66 s
3-OAcyl					
33	2.43, m	2.35 ^c^	2.53 m	2.49 ^c^	2.65 m
34	1.52 m	1.46 m	1.51 m	1.51 ^c^	1.19 d (7.2)
	1.70 m	1.55 m	1.73 m	1.68 m	
35	0.93 t (7.2)	0.80 t (7.2)	0.95 t (7.2)	0.91 t (7.2)	1.20 d (7.2)
36	1.15 d (7.2)	1.04 d (7.2)	1.23 d (7.2)	1.15 d (7.2)	
30-OAcyl					
38	2.66 m	2.63 m	2.66 m	2.71 m	2.72 m
39	1.21 d (7.0)	1.09 d (7.2)	1.20 d (7.0)	1.20 d (7.0)	1.20 d (7.0)
40	1.21 d (7.0)	1.10 d (7.2)	1.20 d (7.0)	1.26 d (7.0)	1.26 d (7.0)
1-OH		5.86 s			

^a^ Recorded in CDCl_3_; ^b^ Recorded in DMSO-*d*_6_; ^c^ Overlapped signals assigned by ^1^H-^1^H COSY, HSQC, and HMBC spectra without designating multiplicity.

**Table 2:** ^13^C (100 MHz) NMR spectroscopic data of compounds **1**–**4** (δ in ppm).

Position	1 ^a^	1 ^b^	2 ^a^	3 ^a^	4 ^a^
1	85.5 qC	85.0 qC	88.6 qC	86.2 qC	86.2 qC
2	203.2 qC	205.9 qC	197.9 qC	199.2 qC	199.4 qC
3	83.1 CH	83.4 CH	79.1 CH	79.9 CH	80.2 CH
4	40.6 qC	40.8 qC	41.9 qC	40.5 qC	40.6 qC
5	42.0 CH	42.4 CH	46.4 CH	39.4 CH	39.4 CH
6	33.5 CH_2_	33.4 CH_2_	34.1 CH_2_	34.3 CH_2_	34.3 CH_2_
7	172.7 qC	173.6 qC	173.4 qC	172.9 qC	172.9 qC
8	73.5 qC	73.6 qC	124.6 qC	132.2 qC	132.3 qC
9	51.3 qC	51.2 qC	161.9 qC	46.8 CH	46.7 CH
10	53.1 qC	53.5 qC	60.5 qC	56.4 qC	56.4 qC
11	16.7 CH_2_	16.5 CH_2_	20.6 CH_2_	18.5 CH_2_	18.6 CH_2_
12	30.1 CH_2_	30.3 CH_2_	30.7 CH_2_	31.3 CH_2_	31.3 CH_2_
13	38.1 qC	37.9 qC	37.8 qC	41.1 qC	41.1 qC
14	65.4 qC	65.1 qC	151.7 qC	139.4 qC	139.3 qC
15	35.2 CH_2_	35.5 CH_2_	113.8 CH	32.8 CH_2_	32.9 CH_2_
16	169.1 qC	169.0 qC	165.4 qC	169.5 qC	169.5 qC
17	76.9 CH	76.9 CH	80.2 CH	80.3 CH	80.3 CH
18	16.2 CH_3_	16.6 CH_3_	16.3 CH_3_	16.6 CH_3_	16.7 CH_3_
19	16.2 CH_3_	16.0 CH_3_	13.7 CH_3_	15.6 CH_3_	15.6 CH_3_
20	119.8 qC	120.3 qC	120.3 qC	120.4 qC	120.4 qC
21	141.7 CH	141.7 CH	141.2 CH	141.3 CH	141.3 CH
22	109.5 CH	110.0 CH	110.1 CH	110.0 CH	110.0 CH
23	143.4 CH	144.6 CH	143.0 CH	143.1 CH	143.0 CH
28	19.1 CH_3_	18.8 CH_3_	21.2 CH_3_	19.9 CH_3_	19.9 CH_3_
29	42.9 CH_2_	43.3 CH_2_	41.2 CH_2_	42.9 CH_2_	42.9 CH_2_
30	86.3 qC	87.2 qC	92.6 qC	91.4 qC	91.4 qC
7-OMe-31	51.9 CH_3_	52.1 CH_3_	52.0 CH_3_	51.7 CH_3_	51.8 CH_3_
3-OAcyl					
32	175.3 qC	175.2 qC	175.7 qC	175.6 qC	176.0 qC
33	40.9 CH	40.8 CH	40.5 CH	40.7 CH	33.9 CH
34	26.8 CH_2_	26.7 CH_2_	26.6 CH_2_	27.0 CH_2_	18.5 CH_3_
35	11.3 CH_3_	11.6 CH_3_	11.6 CH_3_	11.5 CH_3_	18.9 CH_3_
36	16.1 CH_3_	16.3 CH_3_	16.5 CH_3_	16.3 CH_3_	
30-OAcyl					
37	175.4 qC	175.6 qC	175.9 qC	177.3 qC	177.4 qC
38	33.5 CH	33.2 CH	33.8 CH	33.6 CH	33.6 CH
39	18.7 CH_3_	19.0 CH_3_	18.8 CH_3_	19.1 CH_3_	19.1 CH_3_
40	18.8 CH_3_	18.7 CH_3_	19.1 CH_3_	18.9 CH_3_	19.1 CH_3_

^a^ Recorded in CDCl_3_; ^b^ Recorded in DMSO-*d*_6_.

## Supplementary Materials

The following are available online at www.mdpi.com/1660-3397/15/11/333/s1. Copies of HR-ESIMS, and 1D, 2D NMR spectra of Krishnolides A–D (**1**–**4**).
