# Supplementary material for: Krishnolides A–D: New 2-Ketokhayanolides from the Krishna Mangrove, Xylocarpus moluccensis
Source: Mar Drugs. 2017 Oct 27;15(11):333. doi: 10.3390/md15110333 (PMC5706023; doi:10.3390/md15110333)
Supplement: Supplementary file 1 [file marinedrugs-15-00333-s001.pdf]

Supporting Information

Copies of HR-ESIMS, and 1D, 2D NMR spectra of Krishnolides A-D (**1-4**).

Table of Contents:

1. Positive HR-ESIMS for Krishnolide A (**1**) ····· S4
2. UV spectrum of Krishnolide A (**1**) in MeCN ····· S5
3. Experimental ECD spectrum of Krishnolide A (**1**) ····· S6
4. ^{1}H NMR (400 MHz) spectrum of Krishnolide A (**1**) in $CDCl_3$ ····· S7-S10
5. ^{13}C NMR (100 MHz) spectrum of Krishnolide A (**1**) in $CDCl_3$ ····· S11-S15
6. DEPT 135 spectrum of Krishnolide A (**1**) in $CDCl_3$ ····· S16-S19
7. ^{1}H-^{1}H COSY spectrum of Krishnolide A (**1**) in $CDCl_3$ ····· S20-S24
8. HSQC spectrum of Krishnolide A (**1**) in $CDCl_3$ ····· S25-S29
9. HMBC spectrum of Krishnolide A (**1**) in $CDCl_3$ ····· S30-S35
10. NOESY spectrum of Krishnolide A (**1**) in $CDCl_3$ ····· S36-S40
11. ^{1}H NMR (400 MHz) spectrum of Krishnolide A (**1**) in DMSO-d_6 ····· S41-S44
12. ^{13}C NMR (100 MHz) spectrum of Krishnolide A (**1**) in DMSO-d_6 ····· S45-S49
13. DEPT 135 spectrum of Krishnolide A (**1**) in DMSO-d_6 ····· S50-S53
14. ^{1}H-^{1}H COSY spectrum of Krishnolide A (**1**) in DMSO-d_6 ····· S54-S58
15. HSQC spectrum of Krishnolide A (**1**) in DMSO-d_6 ····· S59-S65
16. HMBC spectrum of Krishnolide A (**1**) in DMSO-d_6 ····· S66-S72
17. NOESY spectrum of Krishnolide A (**1**) in DMSO-d_6 ····· S73-S78
18. Positive HR-ESIMS for Krishnolide B (**2**) ····· S79
19. UV spectrum of Krishnolide B (**2**) in MeCN ····· S80
20. ^{1}H NMR (400 MHz) spectrum of Krishnolide B (**2**) in $CDCl_3$ ····· S81-S84
21. ^{13}C NMR (100 MHz) spectrum of Krishnolide B (**2**) in $CDCl_3$ ····· S85-S89
22. DEPT 135 spectrum of Krishnolide B (**2**) in $CDCl_3$ ····· S90-S92

22. ^{1}H-^{1}H COSY spectrum of Krishnolide B (**2**) in $CDCl_3$ ·······························S93-S97
23. HSQC spectrum of Krishnolide B (**2**) in $CDCl_3$ ··································S98-S103
24. HMBC spectrum of Krishnolide B (**2**) in $CDCl_3$ ································S104-S113
25. NOESY spectrum of Krishnolide B (**2**) in $CDCl_3$ ································S114-S118
26. Positive HR-ESIMS for Krishnolide C (**3**) ··S119
27. UV spectrum of Krishnolide C (**3**) in MeCN··S120
28. ^{1}H NMR (400 MHz) spectrum of Krishnolide C (**3**) in $CDCl_3$····················S121-S124
29. ^{13}C NMR (100 MHz) spectrum of Krishnolide C (**3**) in $CDCl_3$···················S125-S128
30. DEPT 135 spectrum of Krishnolide C (**3**) in $CDCl_3$ ······························S129-S131
31. ^{1}H-^{1}H COSY spectrum of Krishnolide C (**3**) in $CDCl_3$ ·························S132-S136
32. HSQC spectrum of Krishnolide C (**3**) in $CDCl_3$ ································S137-S141
33. HMBC spectrum of Krishnolide C (**3**) in $CDCl_3$ ································S142-S150
34. NOESY spectrum of Krishnolide C (**3**) in $CDCl_3$ ································S151-S156
35. Positive HR-ESIMS for Krishnolide D (**4**) ··S157
36. UV spectrum of Krishnolide D (**4**) in MeCN··S158
37. ^{1}H NMR (400 MHz) spectrum of Krishnolide D (**4**) in $CDCl_3$····················S159-S162
38. ^{13}C NMR (100 MHz) spectrum of Krishnolide D (**4**) in $CDCl_3$···················S163-S167
39. DEPT 135 spectrum of Krishnolide D (**4**) in $CDCl_3$ ······························S168-S171
40. ^{1}H-^{1}H COSY spectrum of Krishnolide D (**4**) in $CDCl_3$ ·························S172-S176
41. HSQC spectrum of Krishnolide D (**4**) in $CDCl_3$ ································S177-S182
42. HMBC spectrum of Krishnolide D (**4**) in $CDCl_3$ ································S183-S191
43. NOESY spectrum of Krishnolide D (**4**) in $CDCl_3$ ································S192-S197
44. Figure S1. ORTEP illustration of the X-ray structure of Krishnolide A (**1**) ············S198

HR-ESIMS of Krishnolide A (**1**)

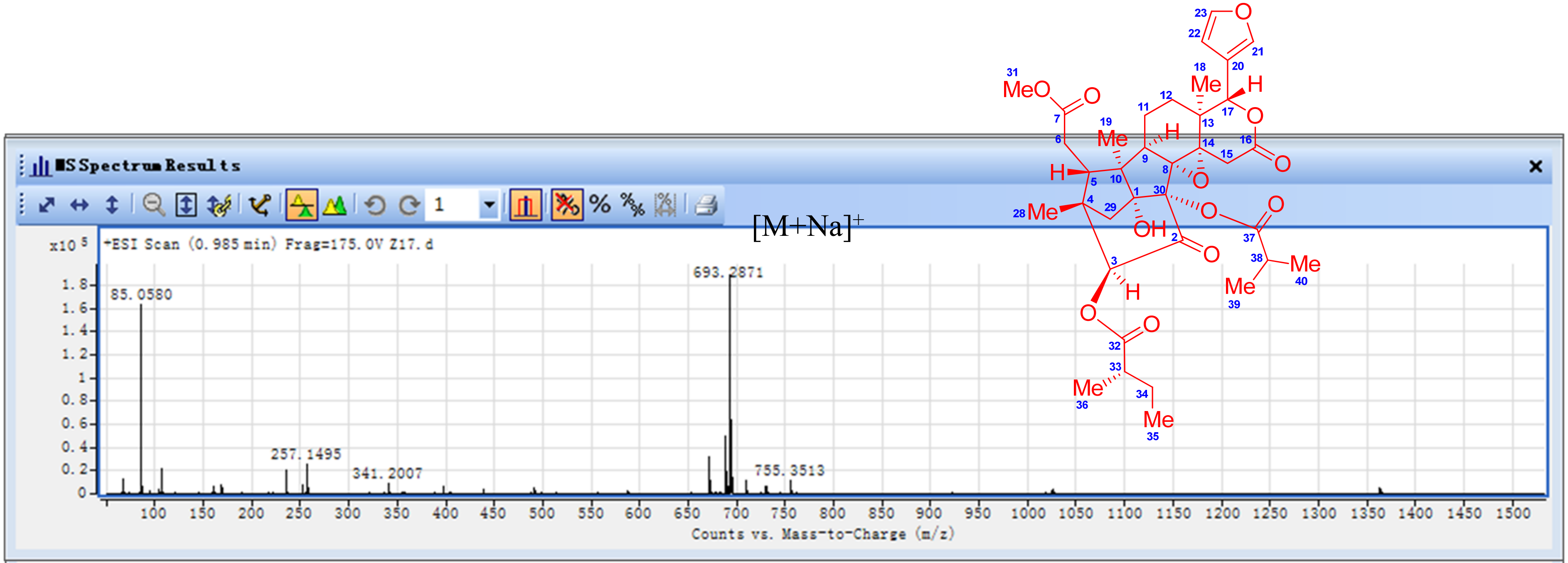

MS Formula Results: + Scan (0.985 min)

m/z	Ion	Formula	Abundance
693.2871	(M+Na)+	C36 H46 Na O12	188389.9

Best	Formula (M)	Ion Formula	Score	Cross Score	Calc m/z	Diff (ppm)	Mass Match	Abund Match	Spacing Match
☑	C36 H46 O12	C36 H46 Na O12	94.81		693.2881	1.49	97.14	87.75	98.64

Isotope	Abund Sum%	Calc Abund Sum%	m/z	Calc m/z	Diff (ppm)
1	69.67	65.74	693.2871	693.2881	1.46
2	23.78	26.25	694.2908	694.2916	1.04
3	5.53	6.72	695.292	695.2943	3.38
4	1.03	1.29	696.2927	696.2971	6.31

UV spectrum of Krishnolide A (**1**) in MeCN

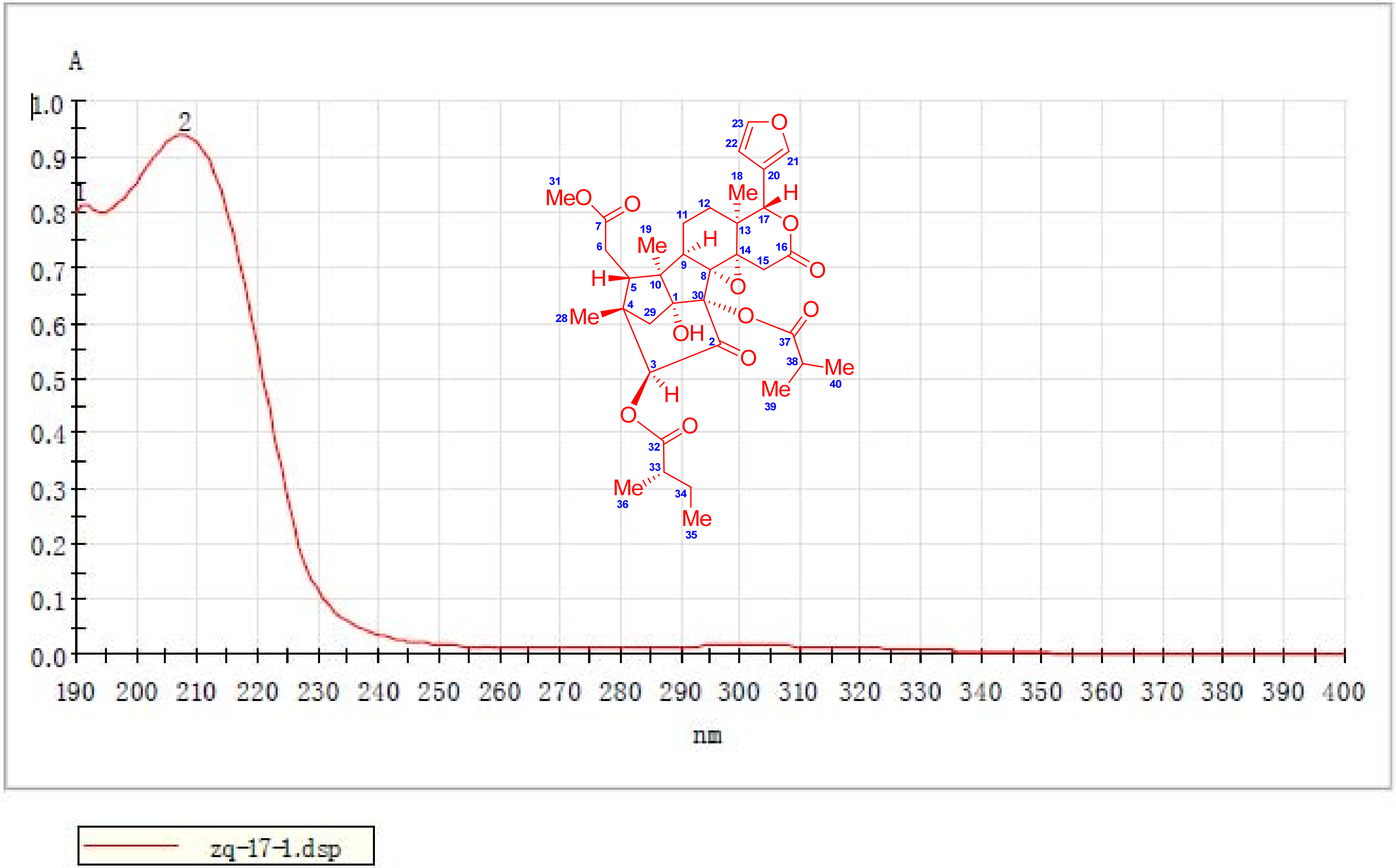

Experimental ECD spectrum of Krishnolide A (**1**) (recorded in MeCN at 250 μg/mL)

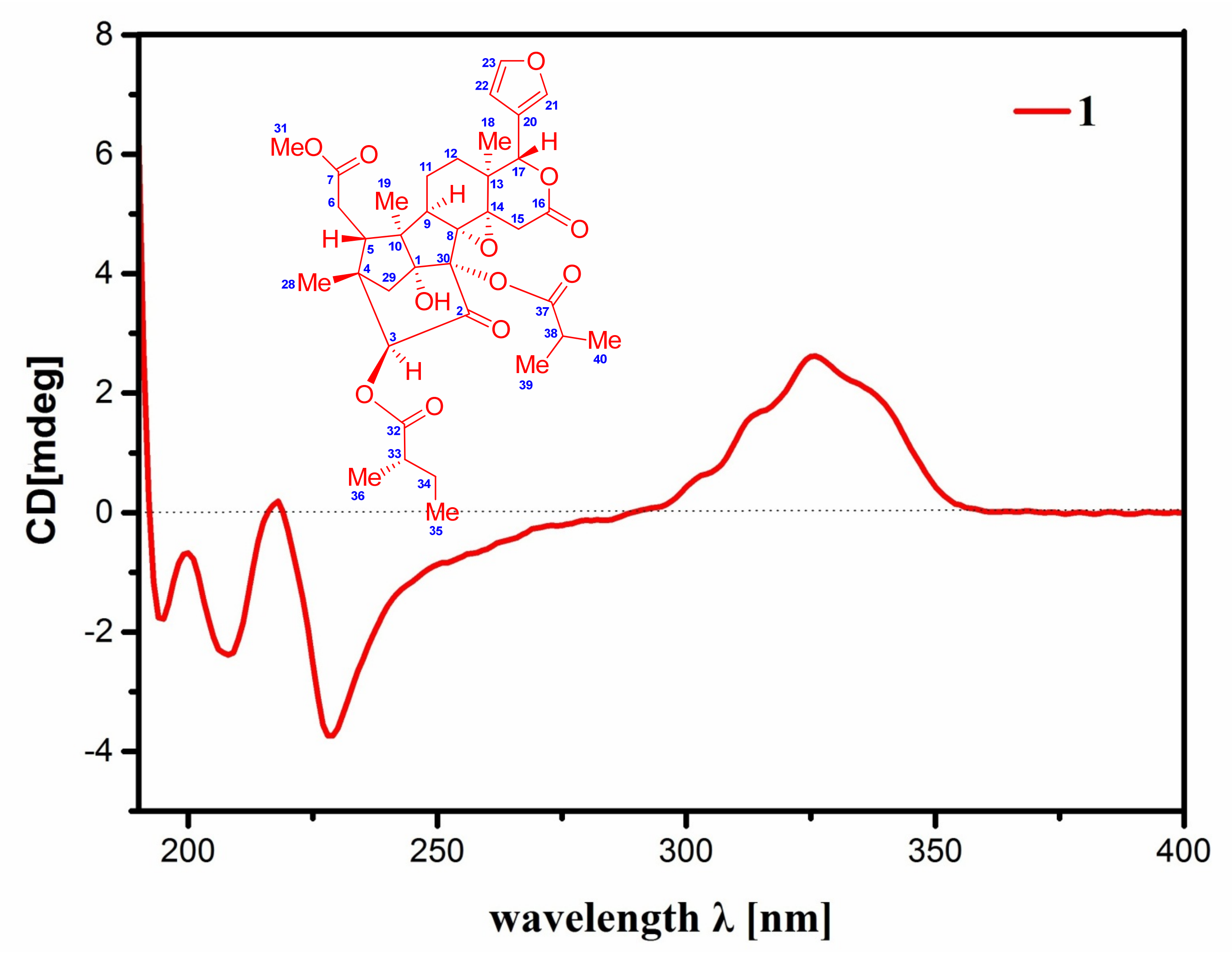

^{1}H NMR (400 MHz) spectrum of Krishnolide A (**1**) in $CDCl_3$

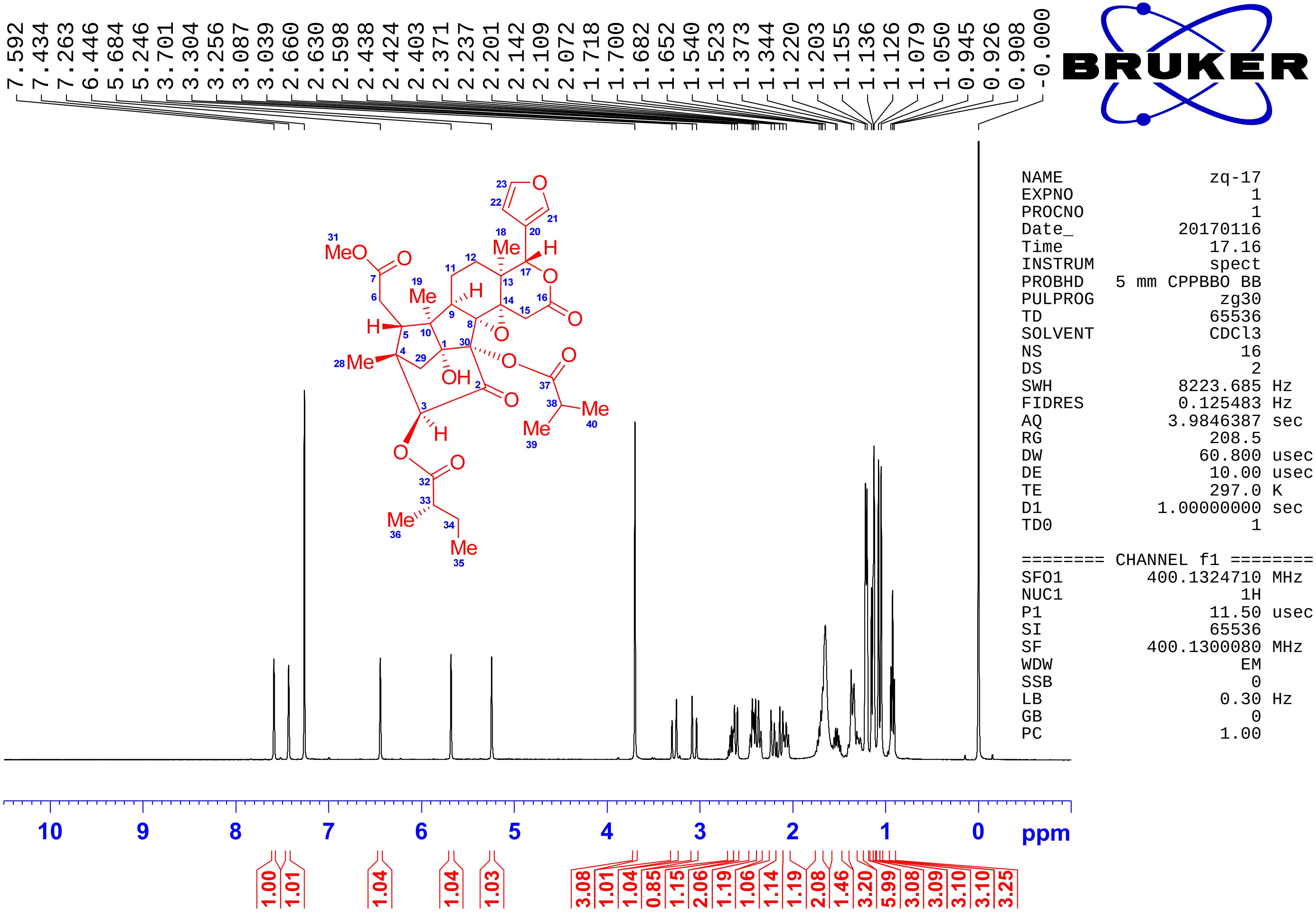

^{1}H NMR (400 MHz) spectrum of Krishnolide A (**1**) in $CDCl_3$

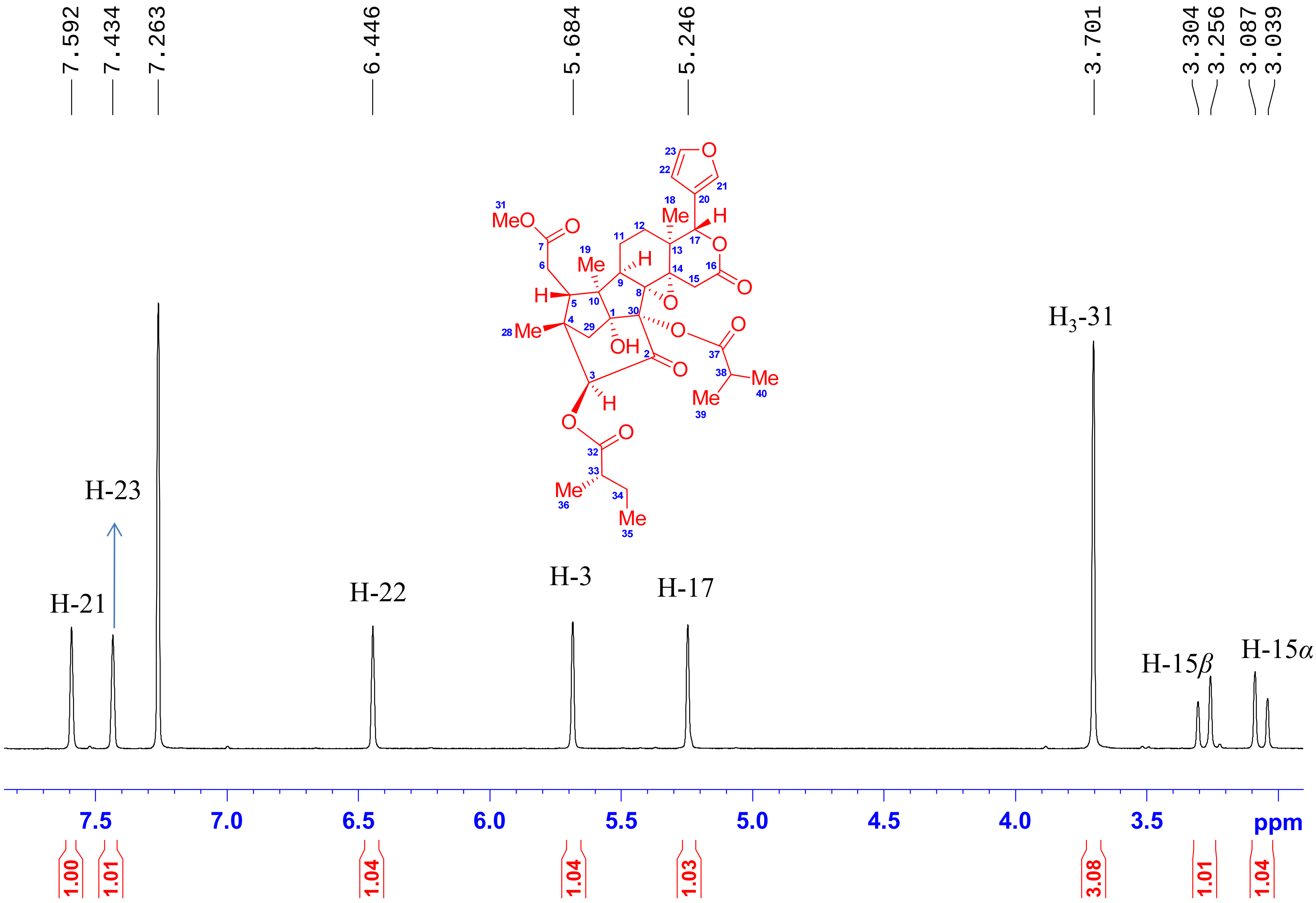

^{1}H NMR (400 MHz) spectrum of Krishnolide A (**1**) in $CDCl_3$

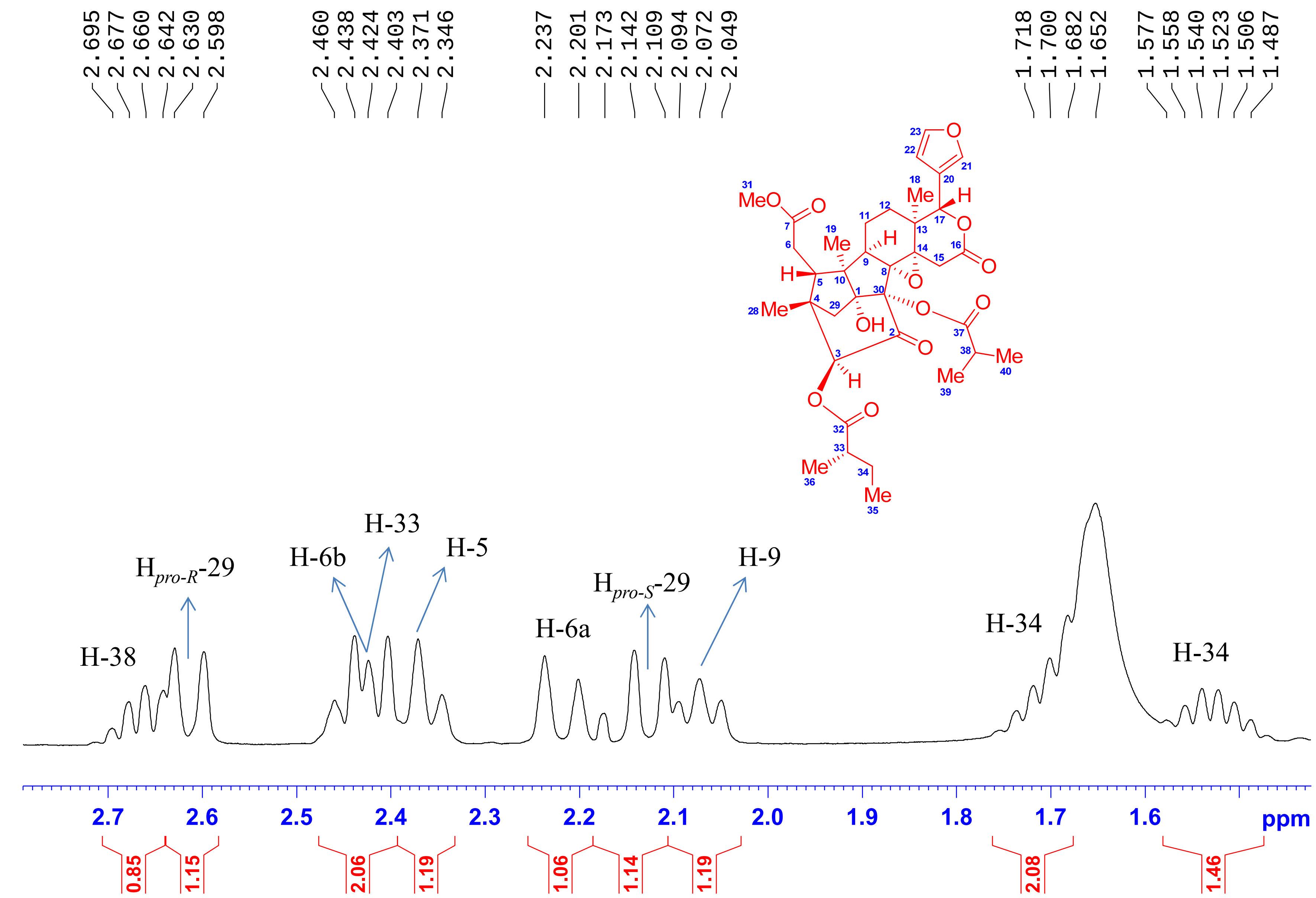

^{1}H NMR (400 MHz) spectrum of Krishnolide A (**1**) in $CDCl_3$

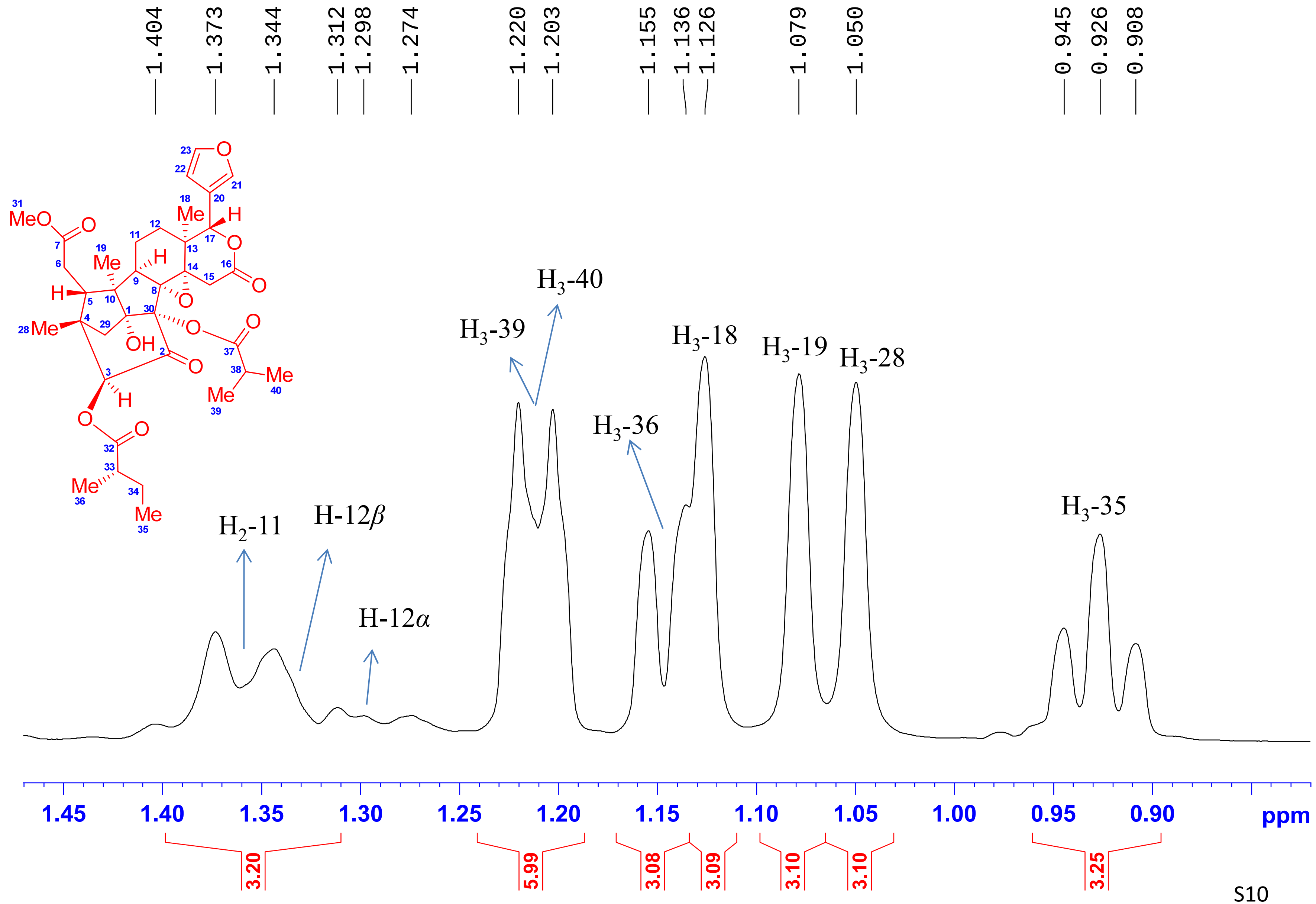

^{13}C NMR (100 MHz) spectrum of Krishnolide A (**1**) in $CDCl_3$

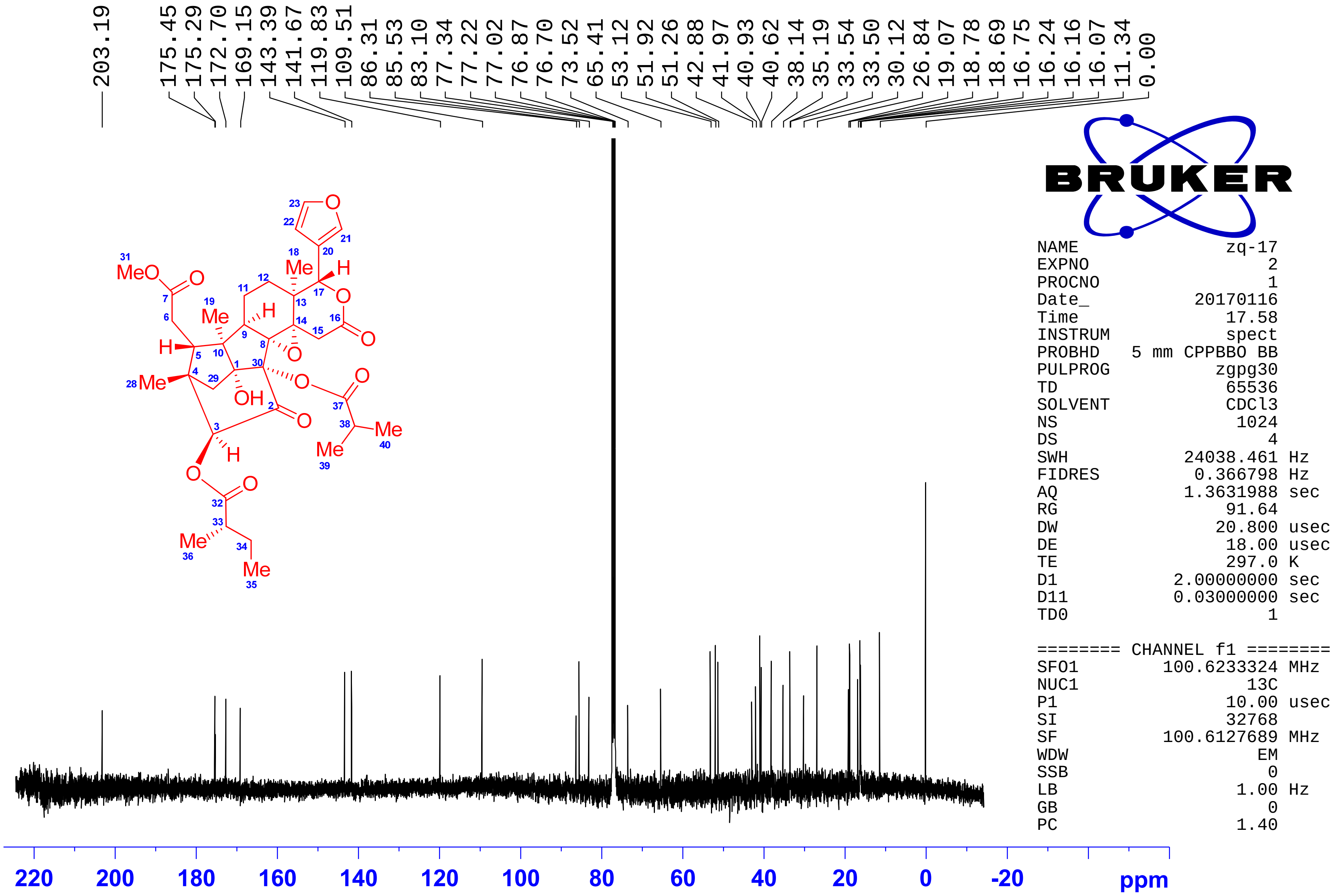

^{13}C NMR (100 MHz) spectrum of Krishnolide A (**1**) in $CDCl_3$

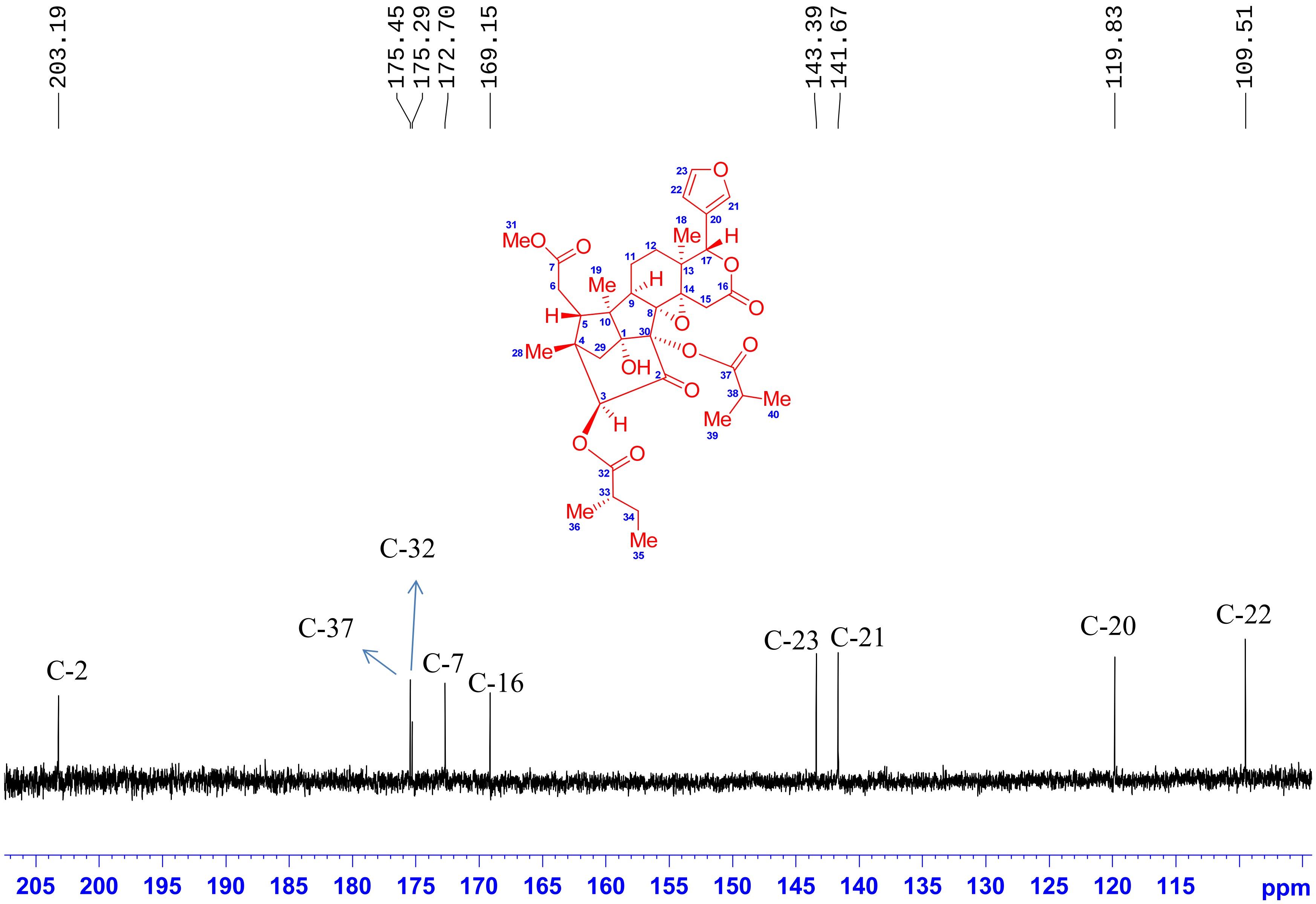

^{13}C NMR (100 MHz) spectrum of Krishnolide A (**1**) in $CDCl_3$

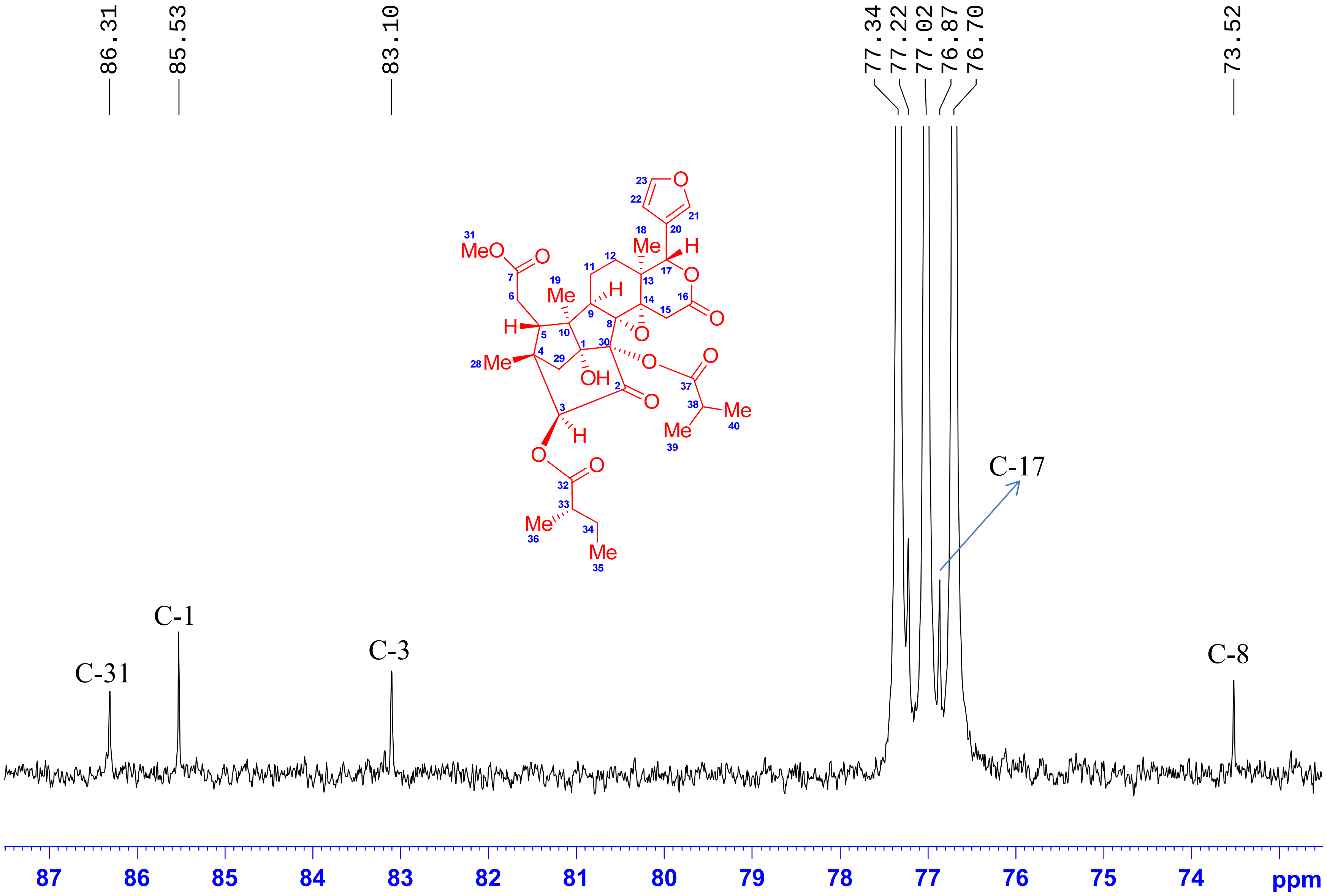

^{13}C NMR (100 MHz) spectrum of Krishnolide A (**1**) in $CDCl_3$

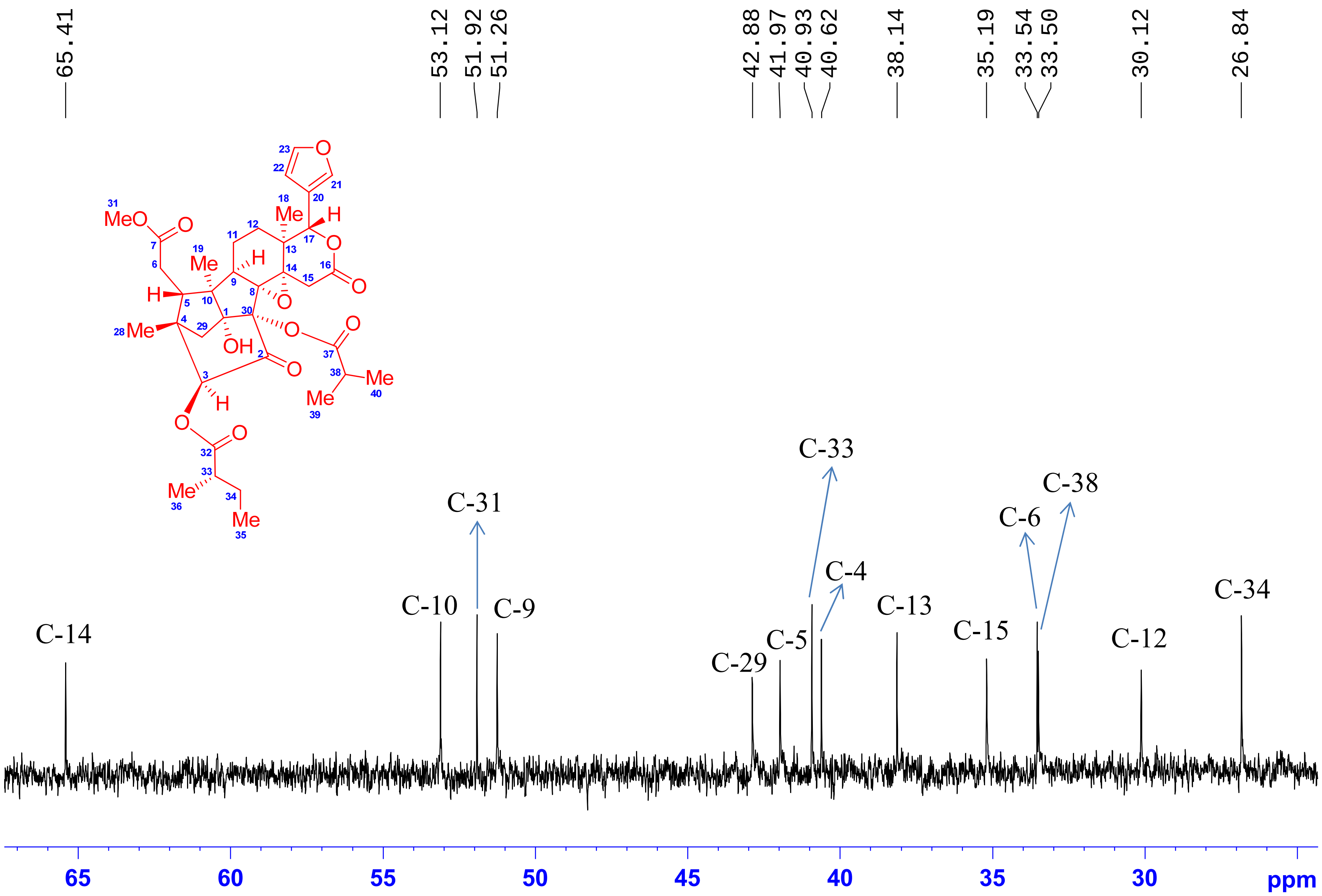

^{13}C NMR (100 MHz) spectrum of Krishnolide A (**1**) in $CDCl_3$

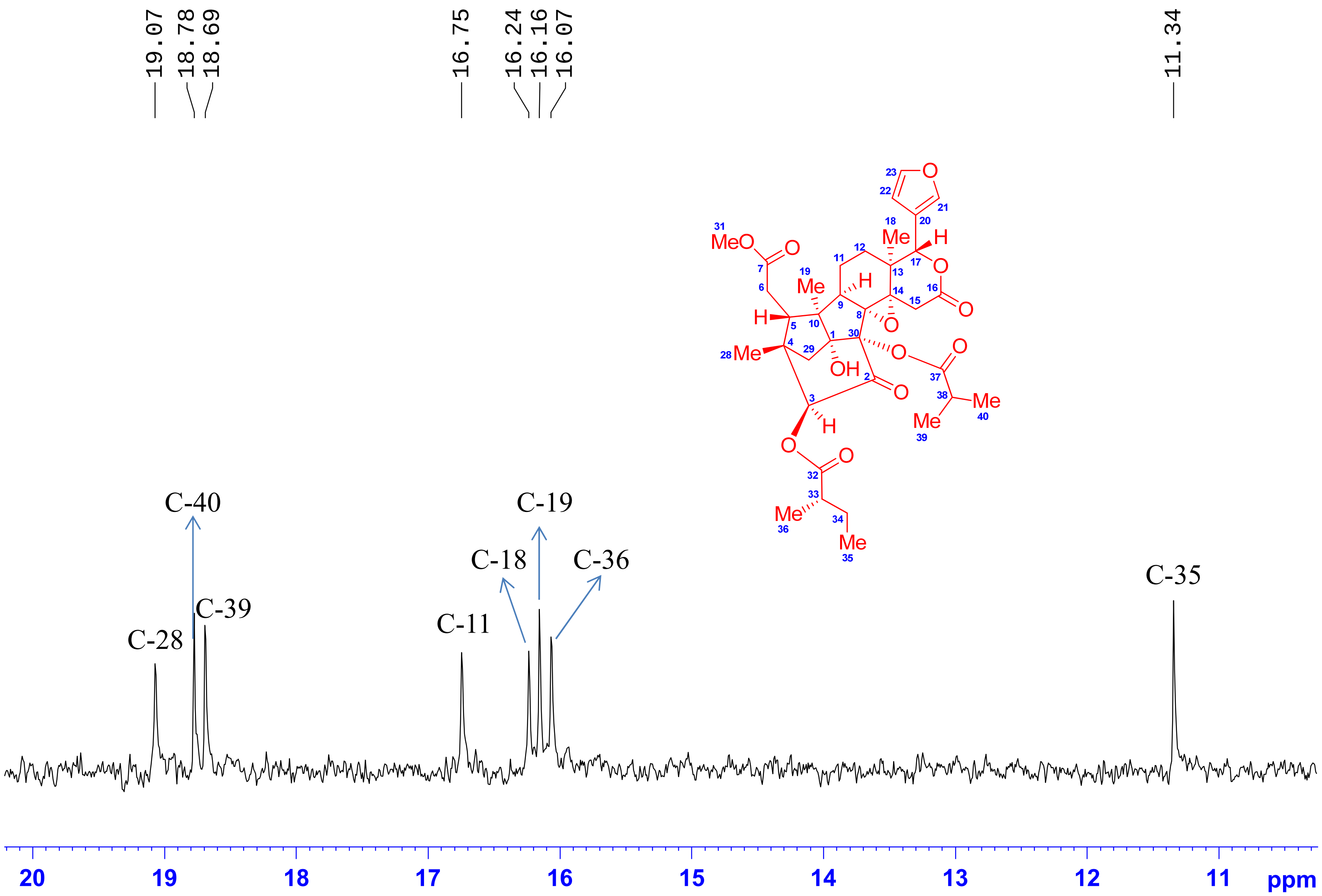

DEPT 135 spectrum of Krishnolide A (**1**) in $CDCl_3$

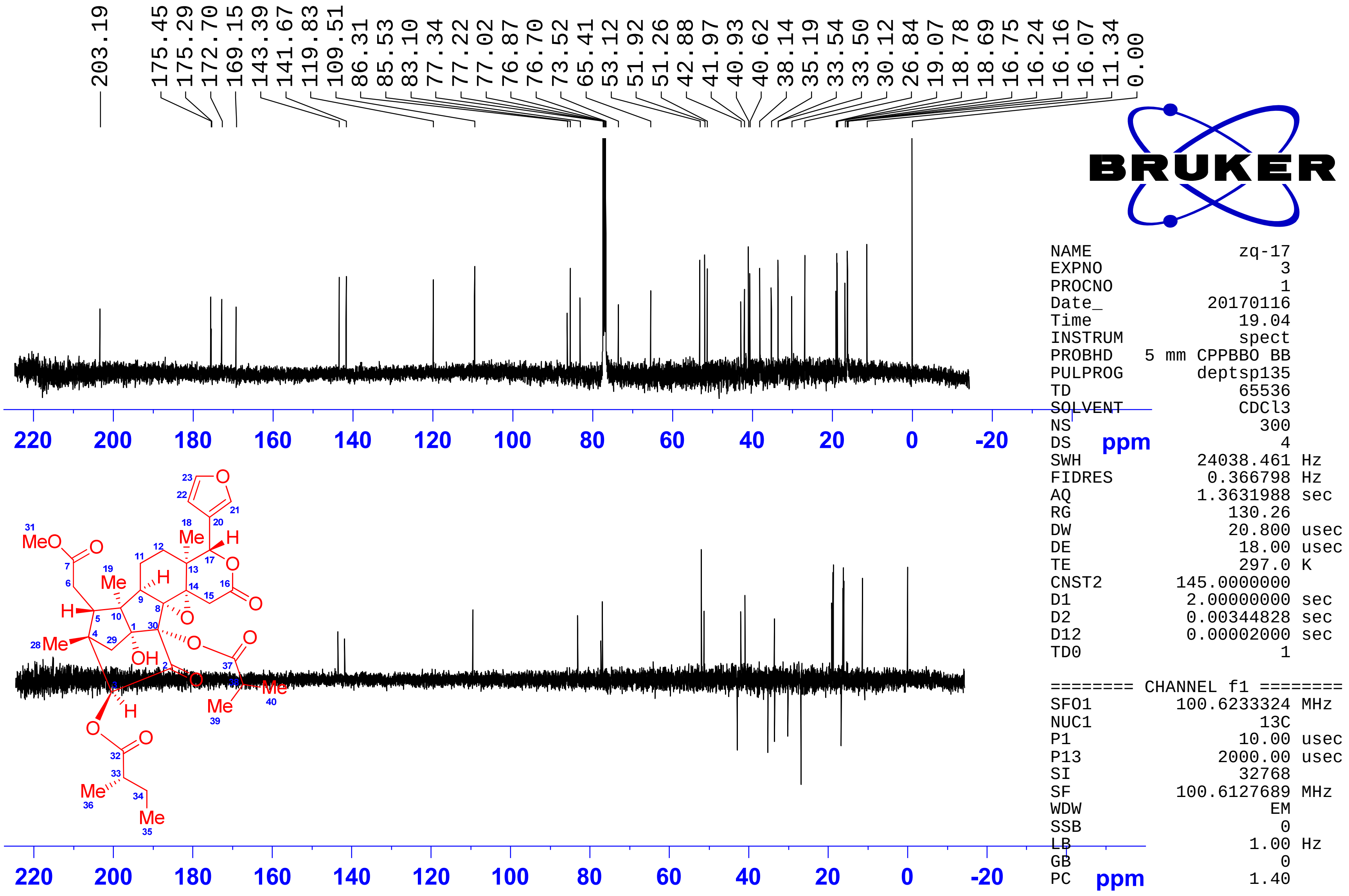

DEPT 135 spectrum of Krishnolide A (**1**) in $CDCl_3$

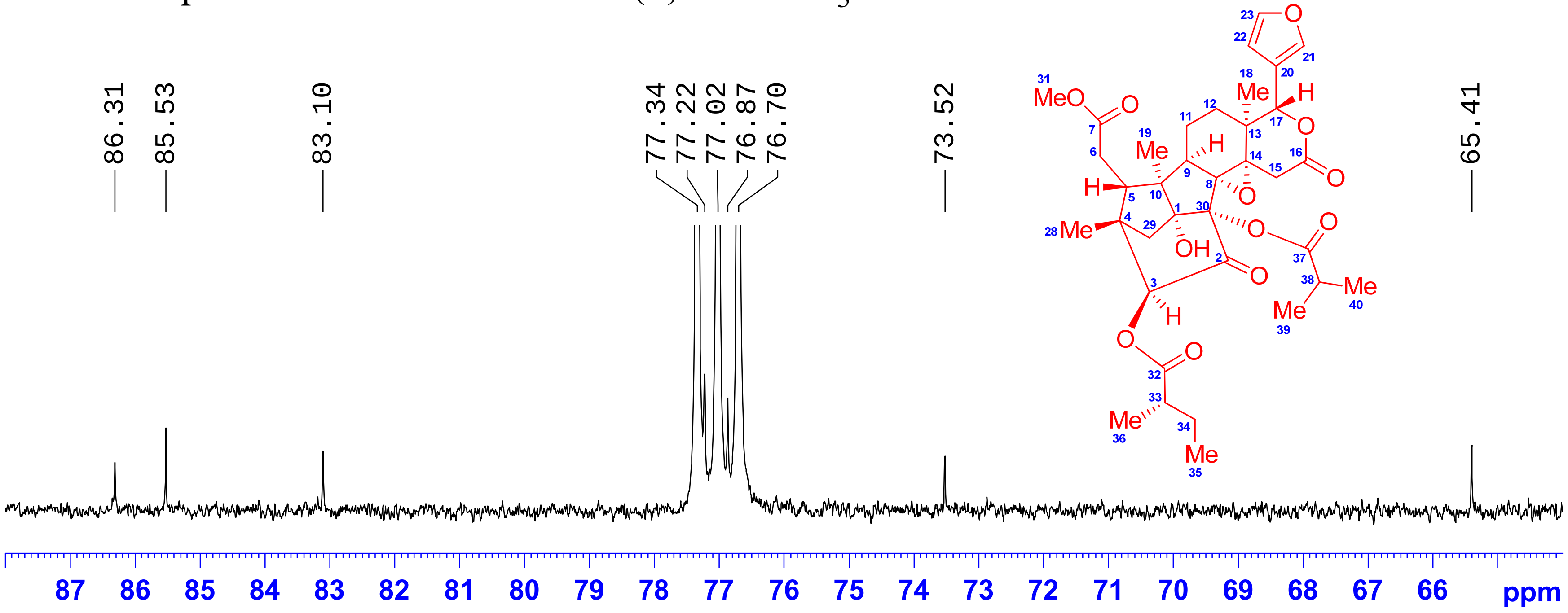

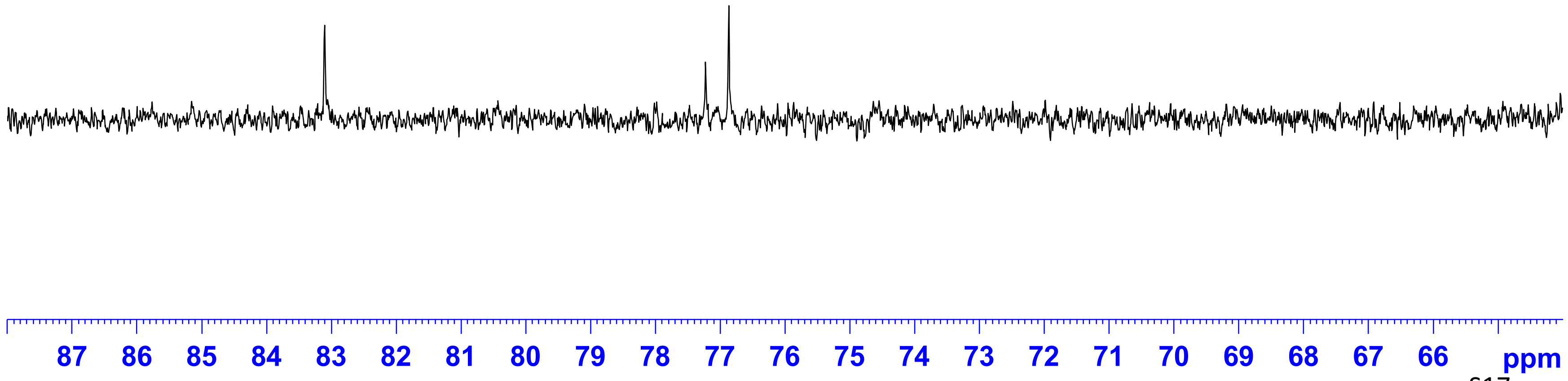

DEPT 135 spectrum of Krishnolide A (**1**) in $CDCl_3$

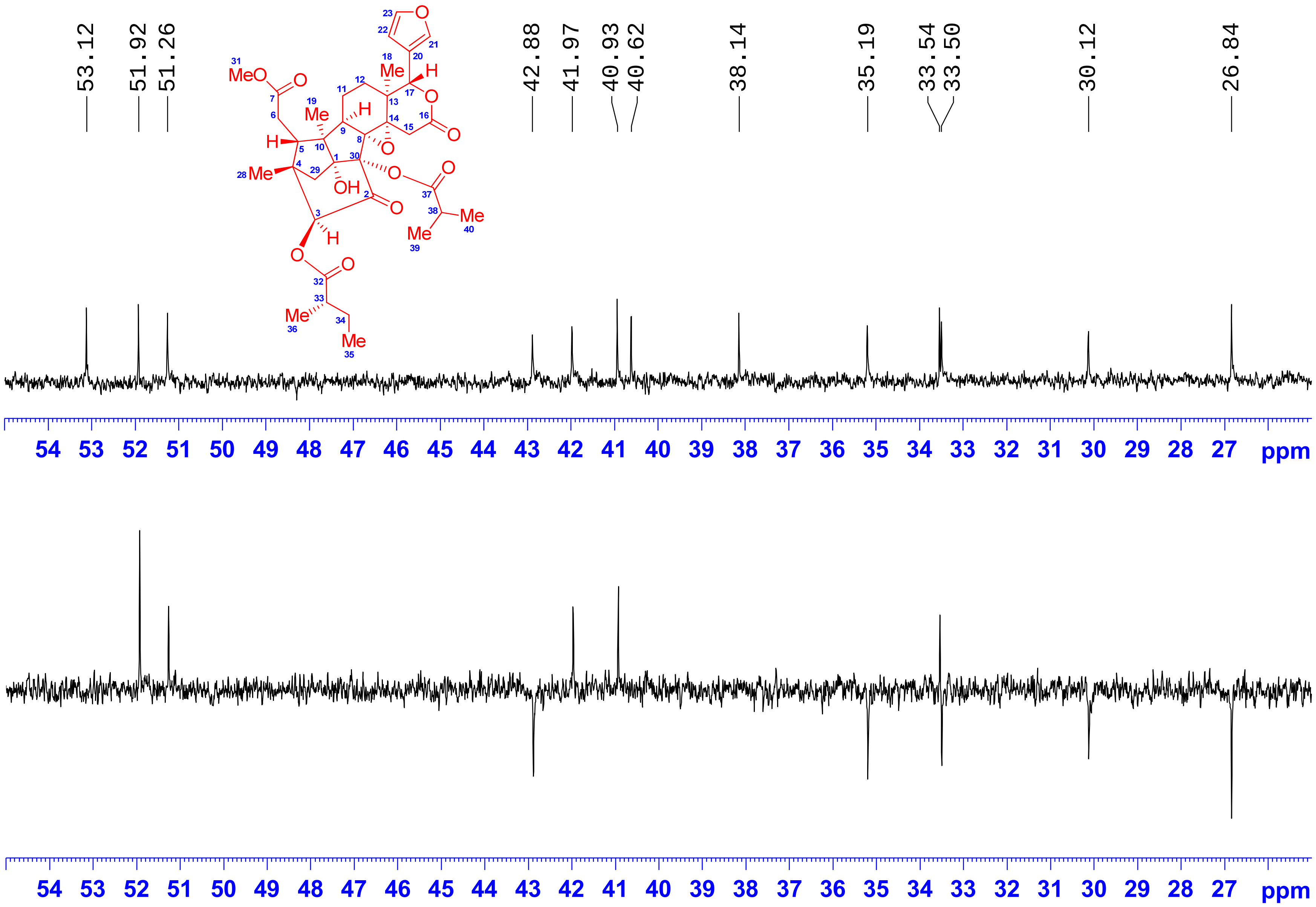

DEPT 135 spectrum of Krishnolide A (**1**) in $CDCl_3$

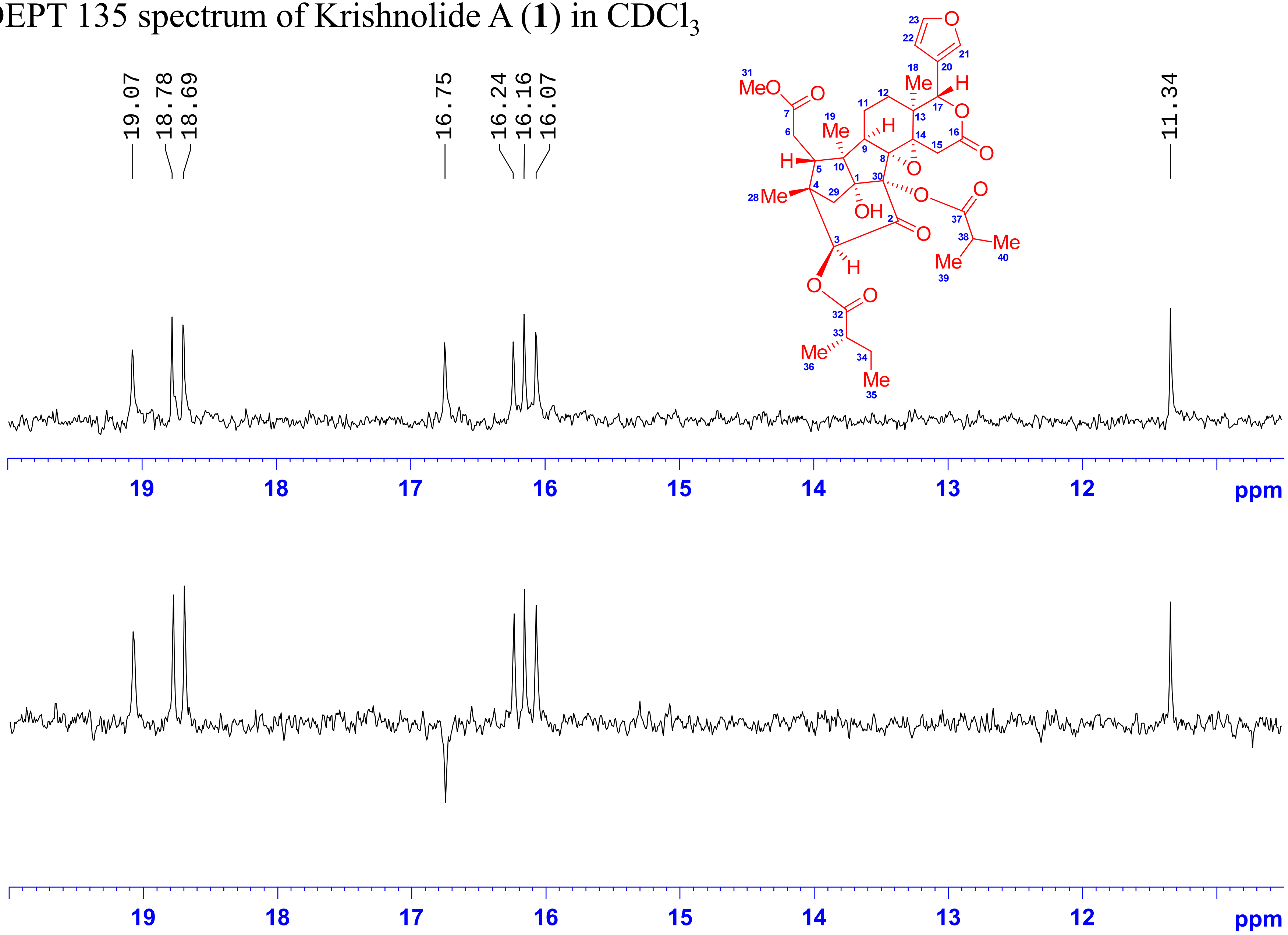

^{1}H-^{1}H COSY spectrum of Krishnolide A (**1**) in $CDCl_3$

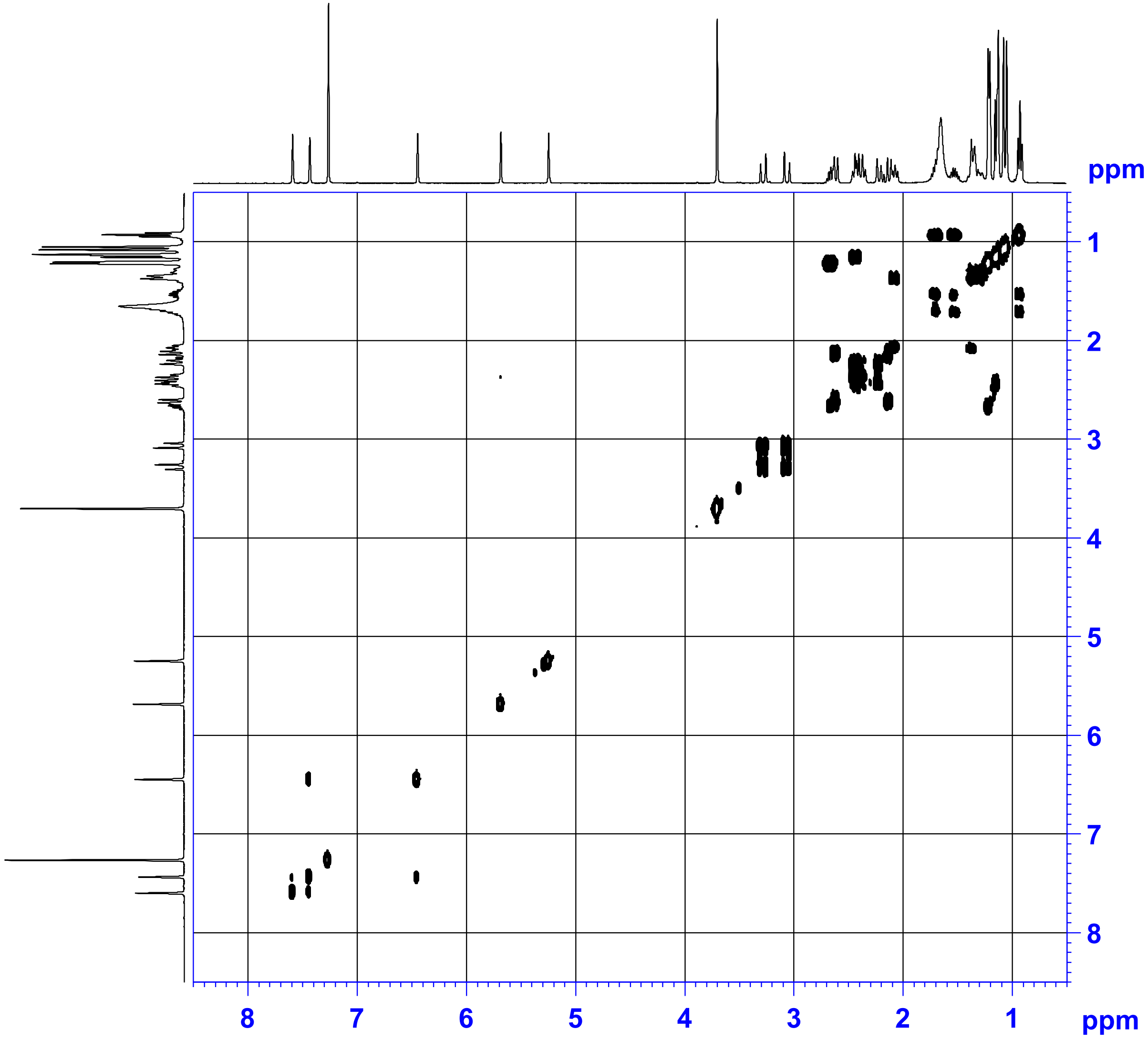

NAME	zq-17
EXPNO	4
PROCNO	1
Date_	20161219
Time	22.48
INSTRUM	spect
PROBHD	5 mm CPPBBO BB
PULPROG	cosygpppqf
TD	2048
SOLVENT	CDCl3
NS	8
DS	8
SWH	3906.250 Hz
FIDRES	1.907349 Hz
AQ	0.2621940 sec
RG	208.5
DW	128.000 usec
DE	10.00 usec
TE	297.0 K
D0	0.00000300 sec
D1	1.89678097 sec
D11	0.03000000 sec
D12	0.00002000 sec
D13	0.00000400 sec
D16	0.00020000 sec
IN0	0.00025600 sec

======== CHANNEL f1 ========

SFO1	400.1318006 MHz
NUC1	1H
P0	11.50 usec
P1	11.50 usec
P17	2500.00 usec
ND0	1
TD	128
SFO1	400.1318 MHz
FIDRES	30.517578 Hz
SW	9.762 ppm
FnMODE	QF
SI	1024
SF	400.1300055 MHz
WDW	QSINE
SSB	0
LB	0.00 Hz
GB	0
PC	1.40
SI	1024
MC2	QF
SF	400.1300055 MHz
WDW	QSINE
SSB	0
LB	0.00 Hz
GB	0

^{1}H-^{1}H COSY spectrum of Krishnolide A (**1**) in $CDCl_3$

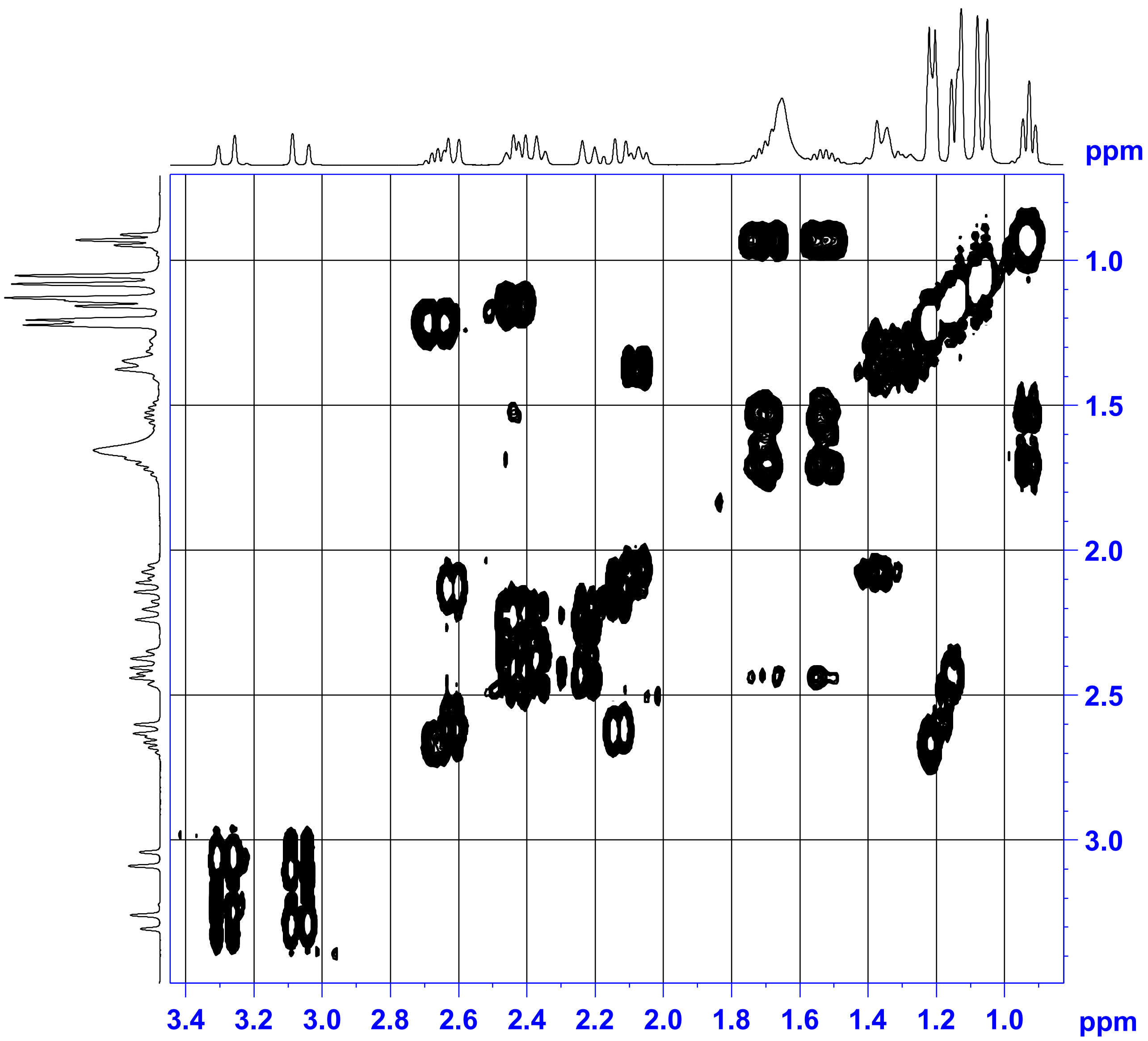

^{1}H-^{1}H COSY spectrum of Krishnolide A (**1**) in $CDCl_3$

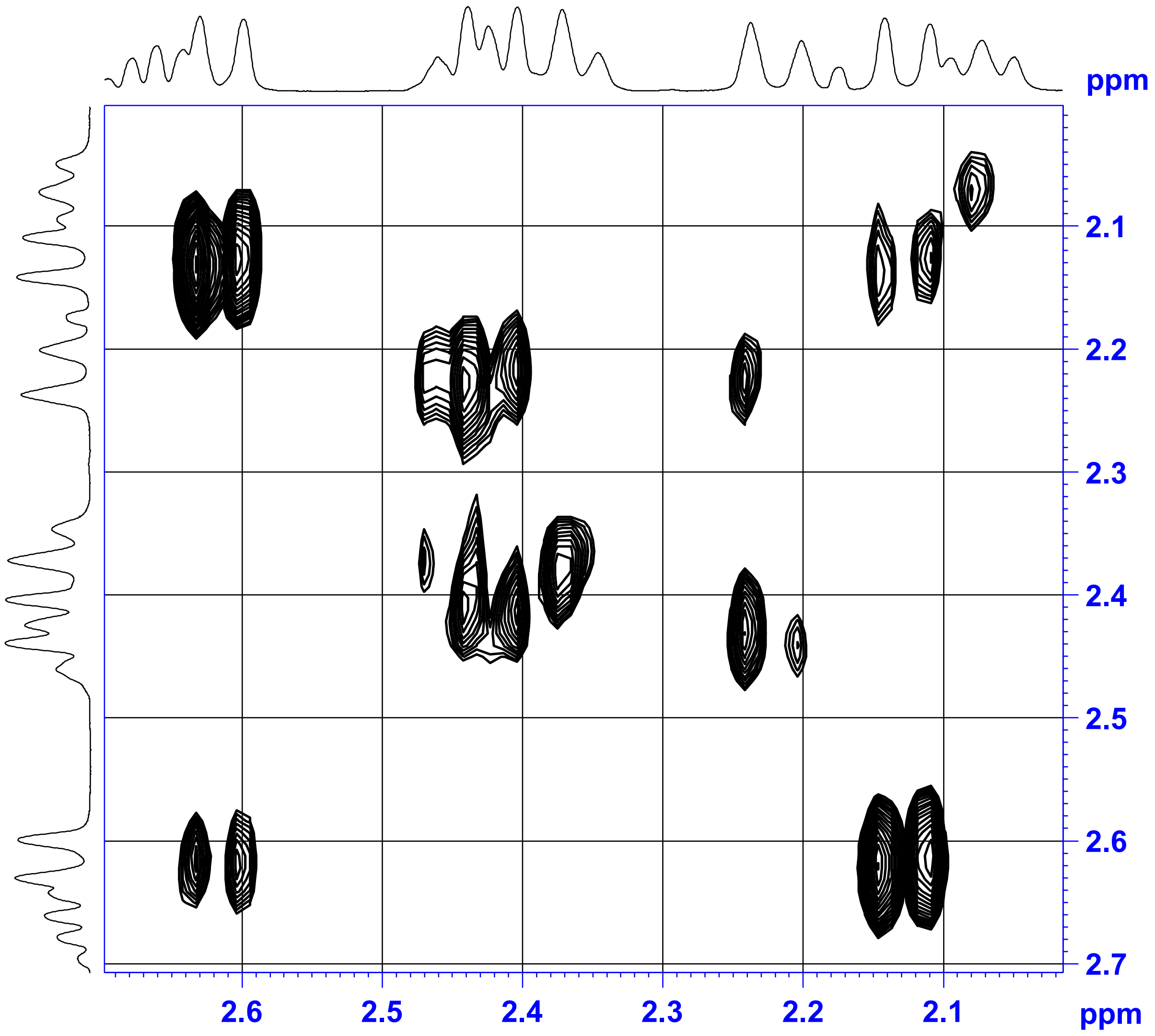

^{1}H-^{1}H COSY spectrum of Krishnolide A (**1**) in $CDCl_3$

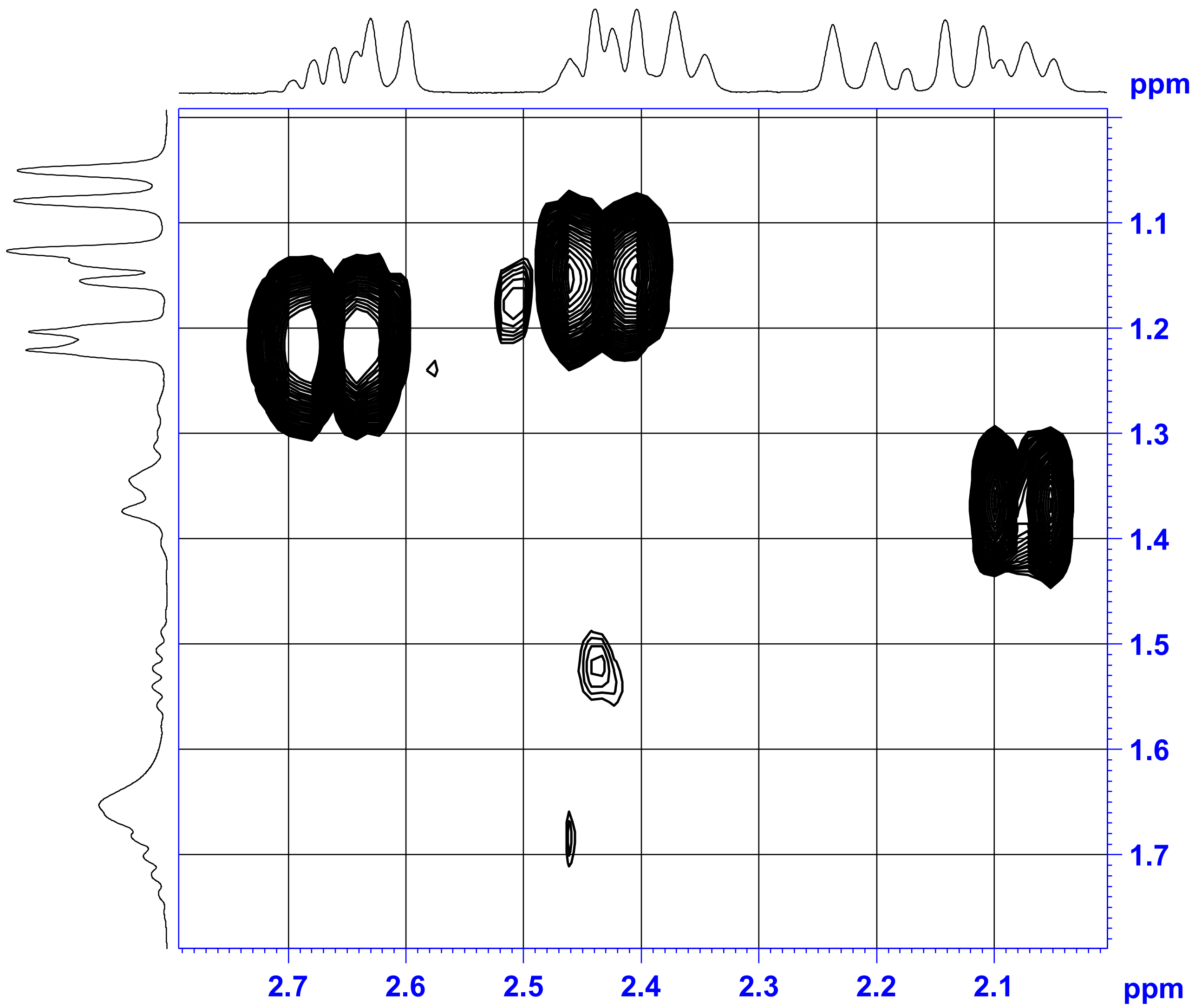

^{1}H-^{1}H COSY spectrum of Krishnolide A (**1**) in $CDCl_3$

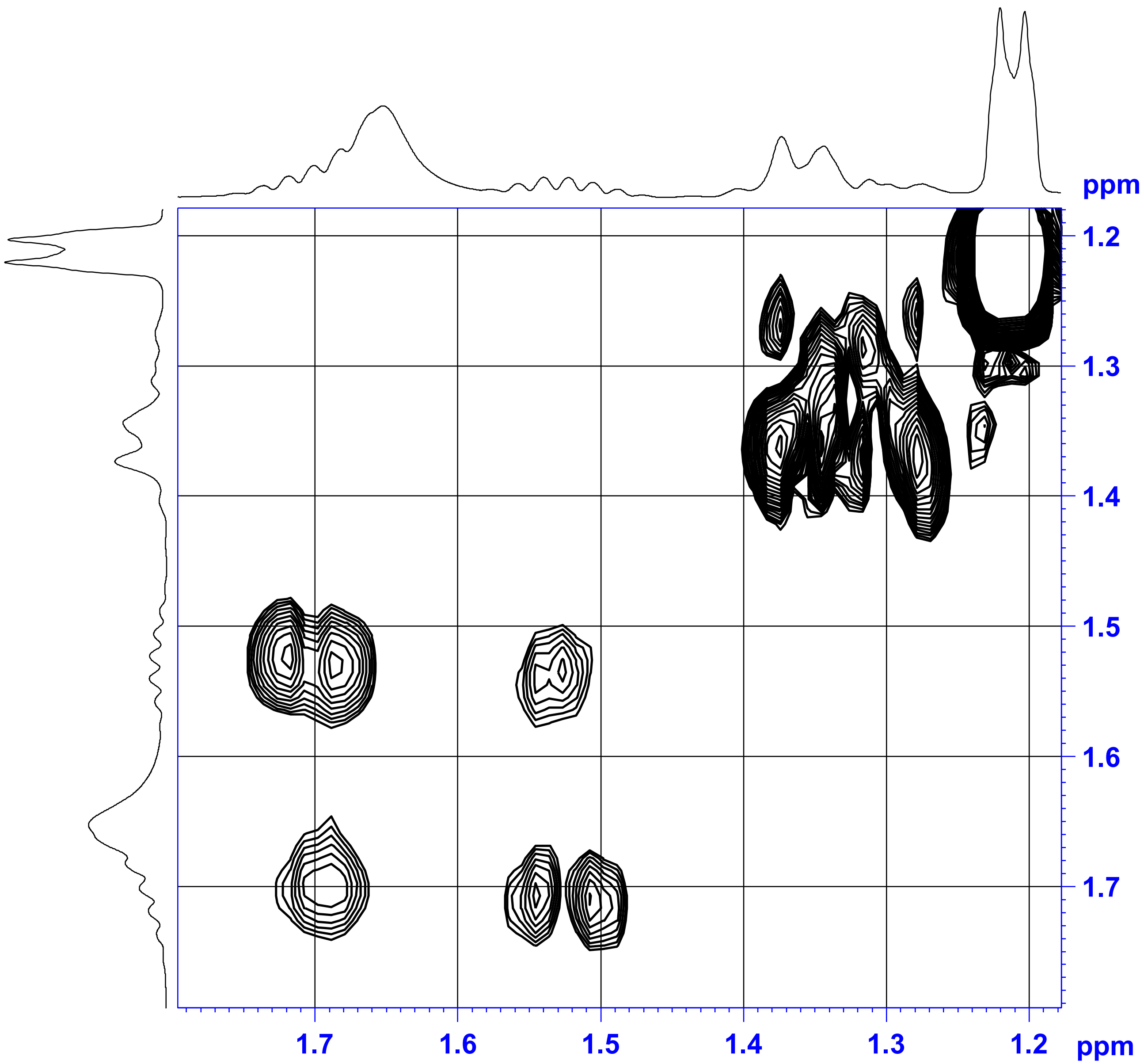

HSQC spectrum of Krishnolide A (**1**) in $CDCl_3$

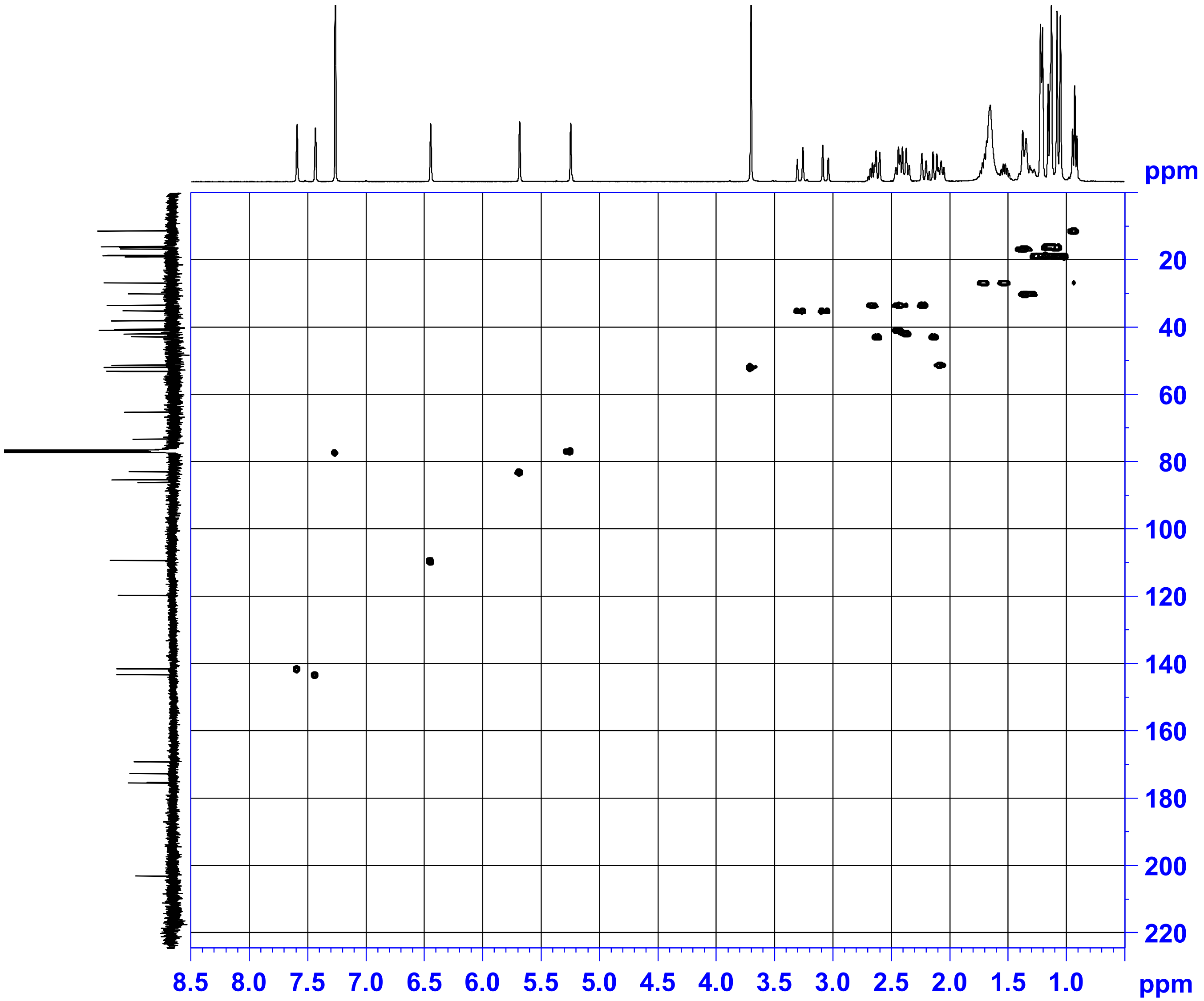

NAME	zq-17
EXPNO	5
PROCNO	1
Date_	20161219
Time	23.28
INSTRUM	spect
PROBHD	5 mm CPPBBO BB
PULPROG	hsqcetgpsi2
TD	1024
SOLVENT	CDCl3
NS	16
DS	16
SWH	4302.926 Hz
FIDRES	4.202076 Hz
AQ	0.1190388 sec
RG	208.5
DW	116.200 usec
DE	10.00 usec
TE	297.0 K
CNST2	145.0000000
D0	0.00000300 sec
D1	1.46497905 sec
D4	0.00172414 sec
D11	0.03000000 sec
D16	0.00020000 sec
D24	0.00086207 sec
IN0	0.00002080 sec
ZGOPTNS	

======== CHANNEL f1 ========

SFO1	400.1320007 MHz
NUC1	1H
P1	11.50 usec
P2	23.00 usec
P28	0.00 usec
ND0	2
TD	256
SFO1	100.6233 MHz
FIDRES	93.900238 Hz
SW	238.896 ppm
FnMODE	Echo-Antiecho
SI	1024
SF	400.1300055 MHz
WDW	QSINE
SSB	2
LB	0.00 Hz
GB	0
PC	1.40
SI	1024
MC2	echo-antiecho
SF	100.6127570 MHz
WDW	QSINE
SSB	2
LB	0.00 Hz
GB	0

HSQC spectrum of Krishnolide A (**1**) in $CDCl_3$

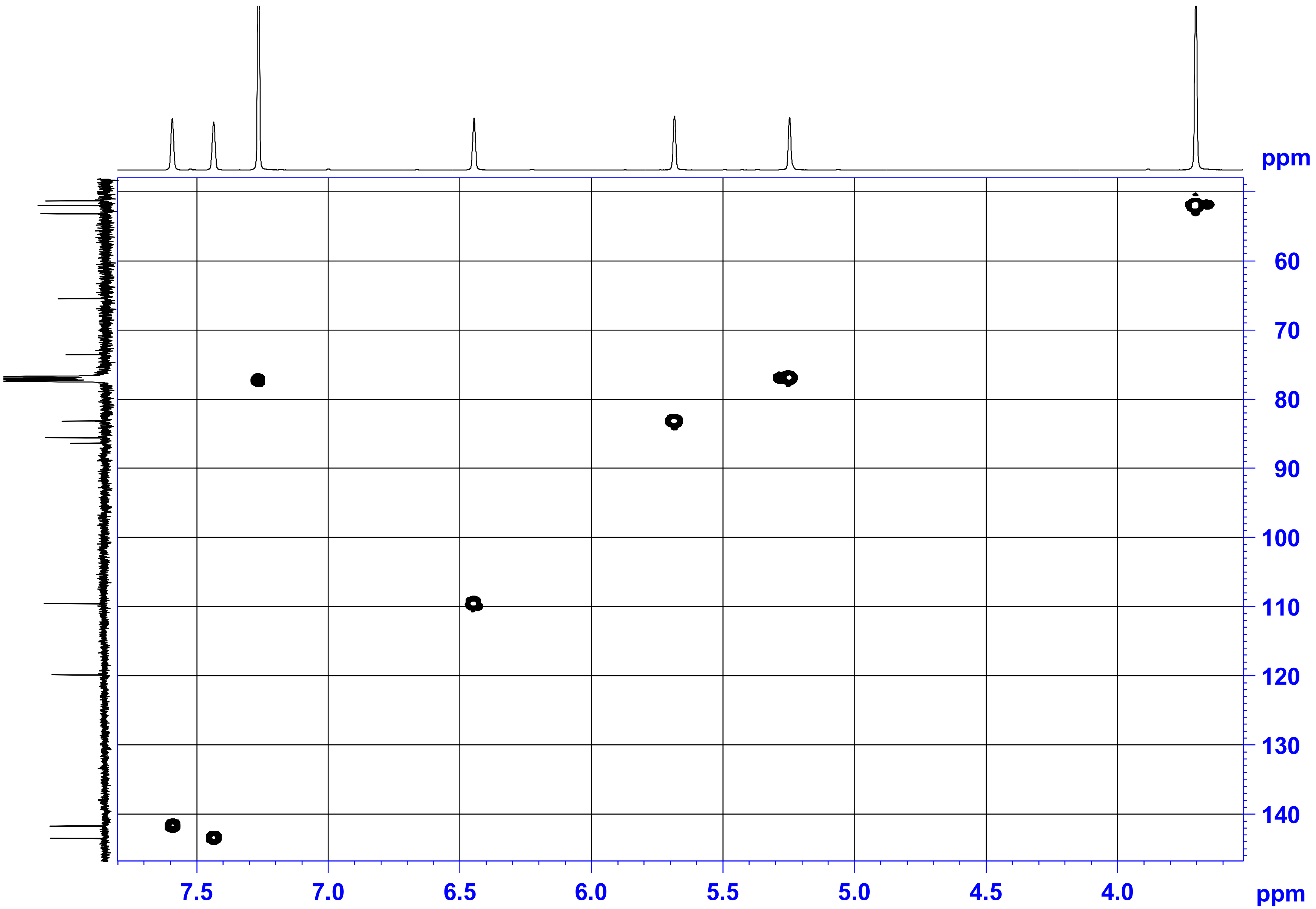

HSQC spectrum of Krishnolide A (**1**) in $CDCl_3$

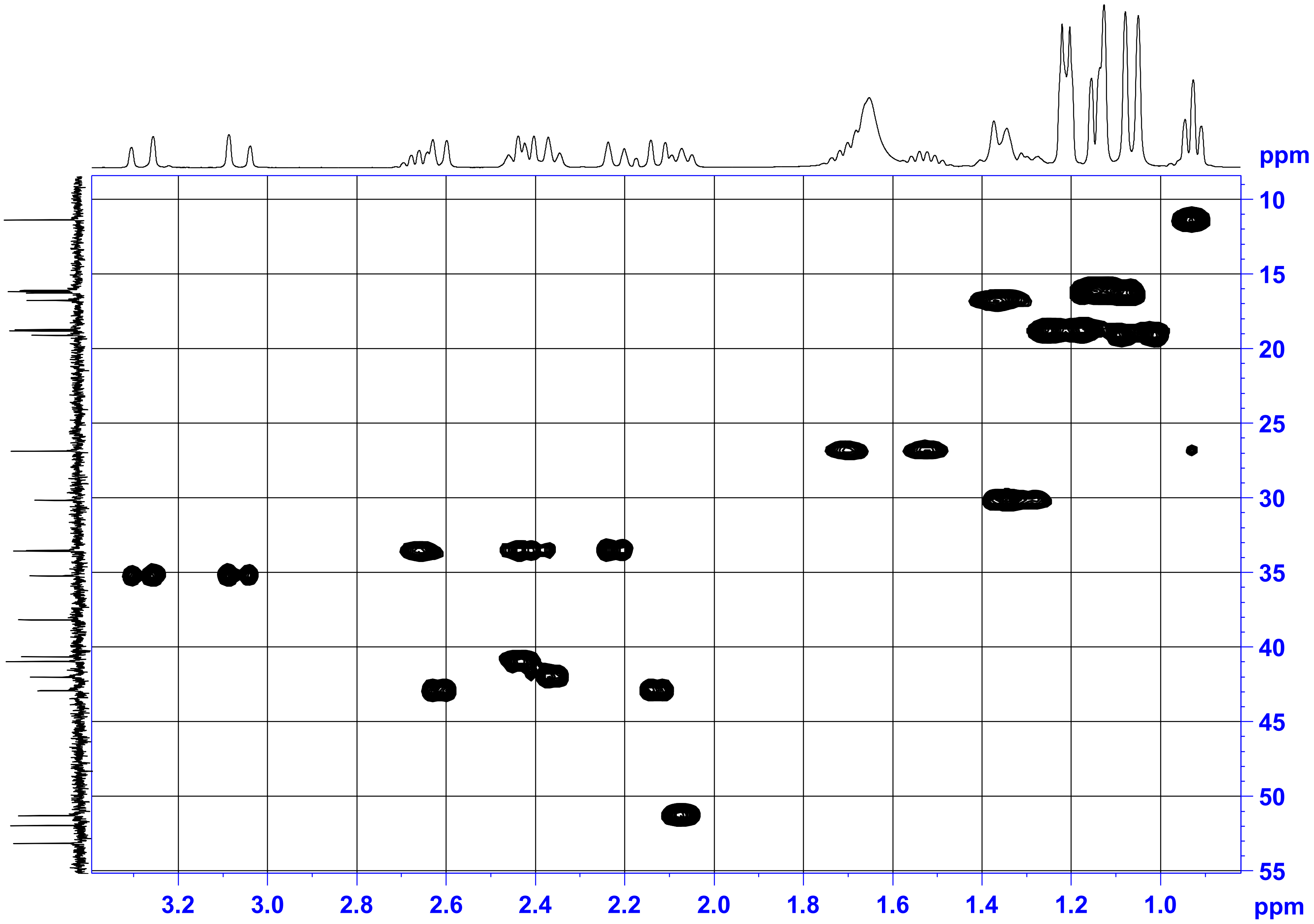

HSQC spectrum of Krishnolide A (**1**) in $CDCl_3$

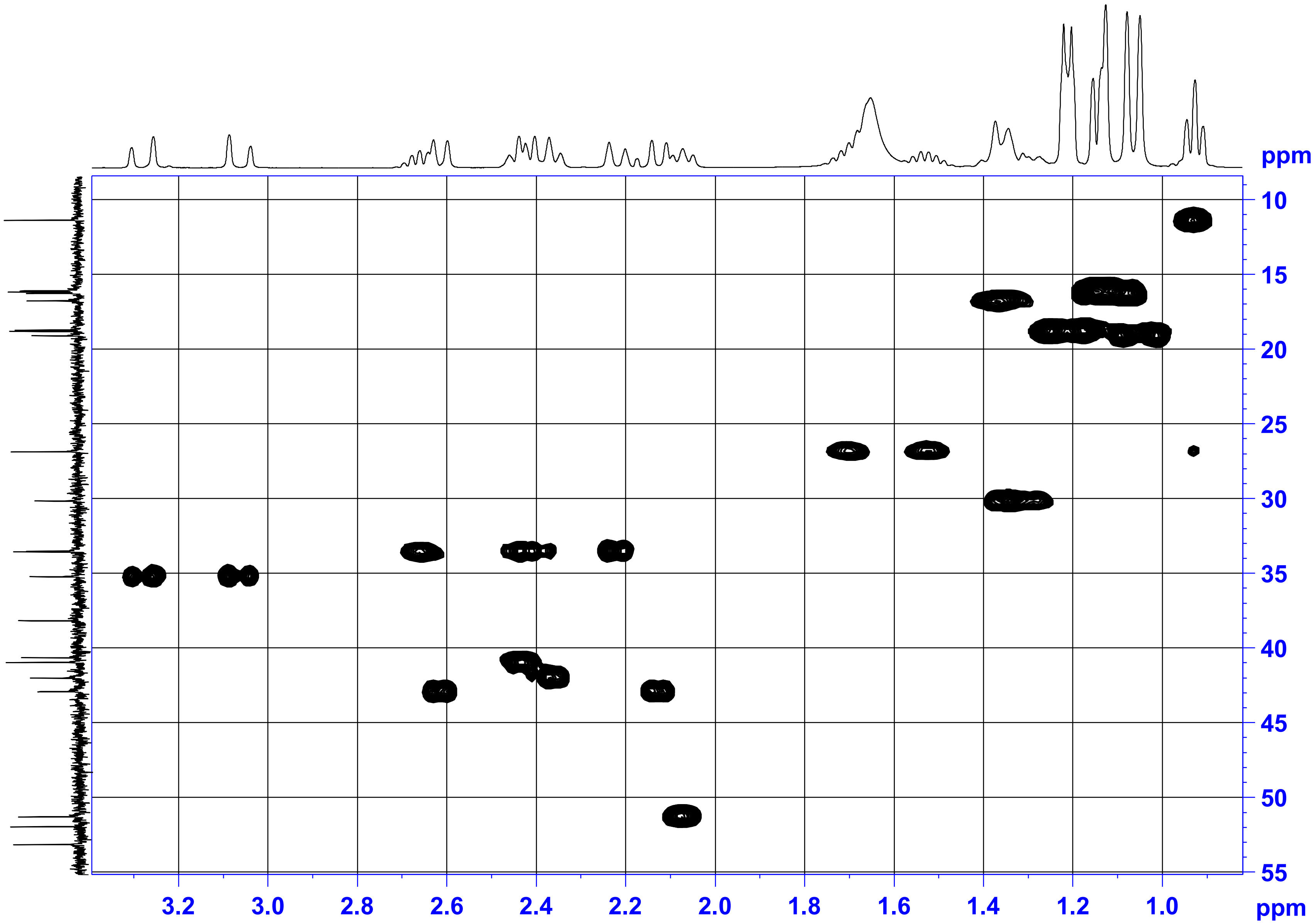

HSQC spectrum of Krishnolide A (**1**) in $CDCl_3$

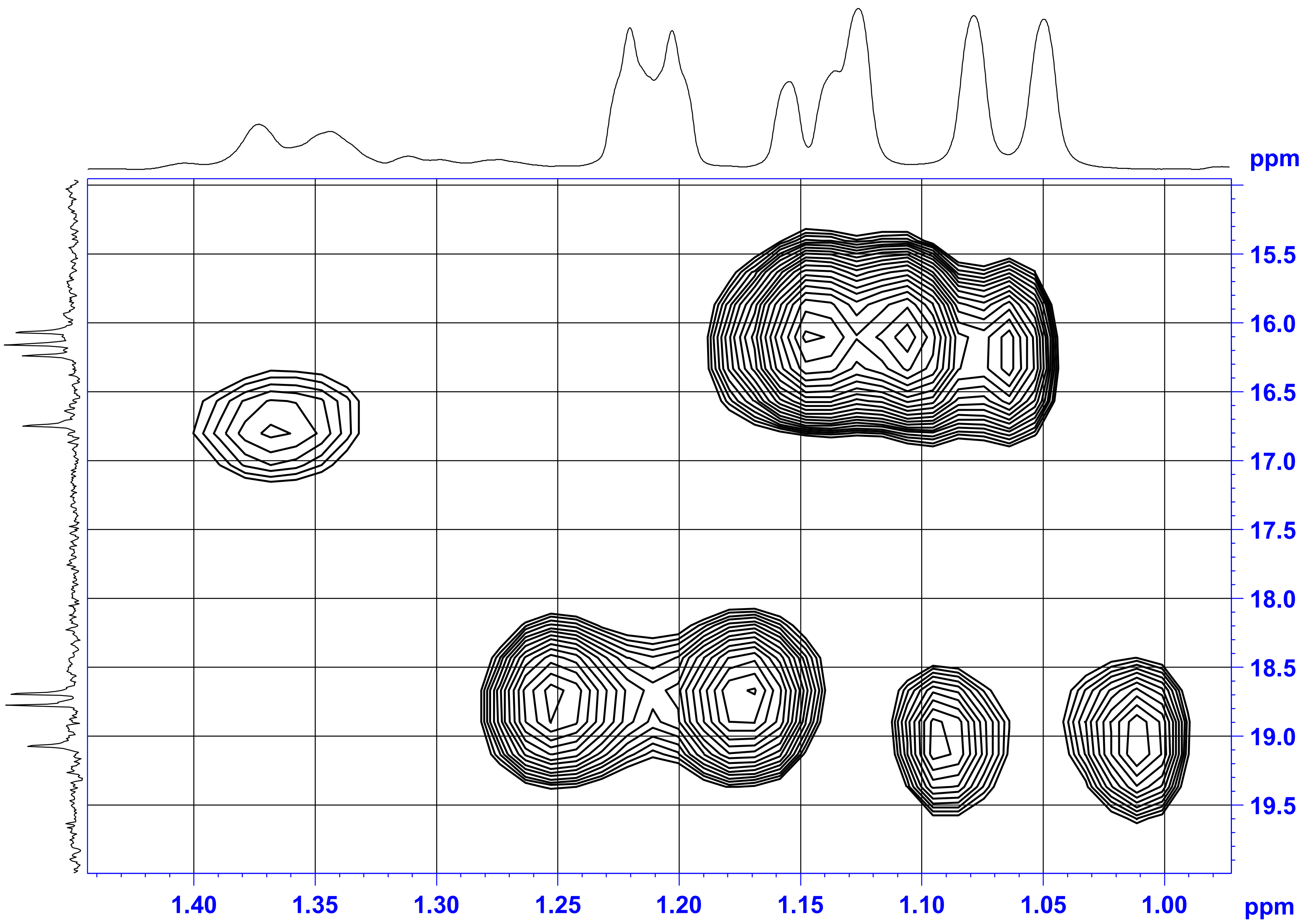

HMBC spectrum of Krishnolide A (**1**) in $CDCl_3$

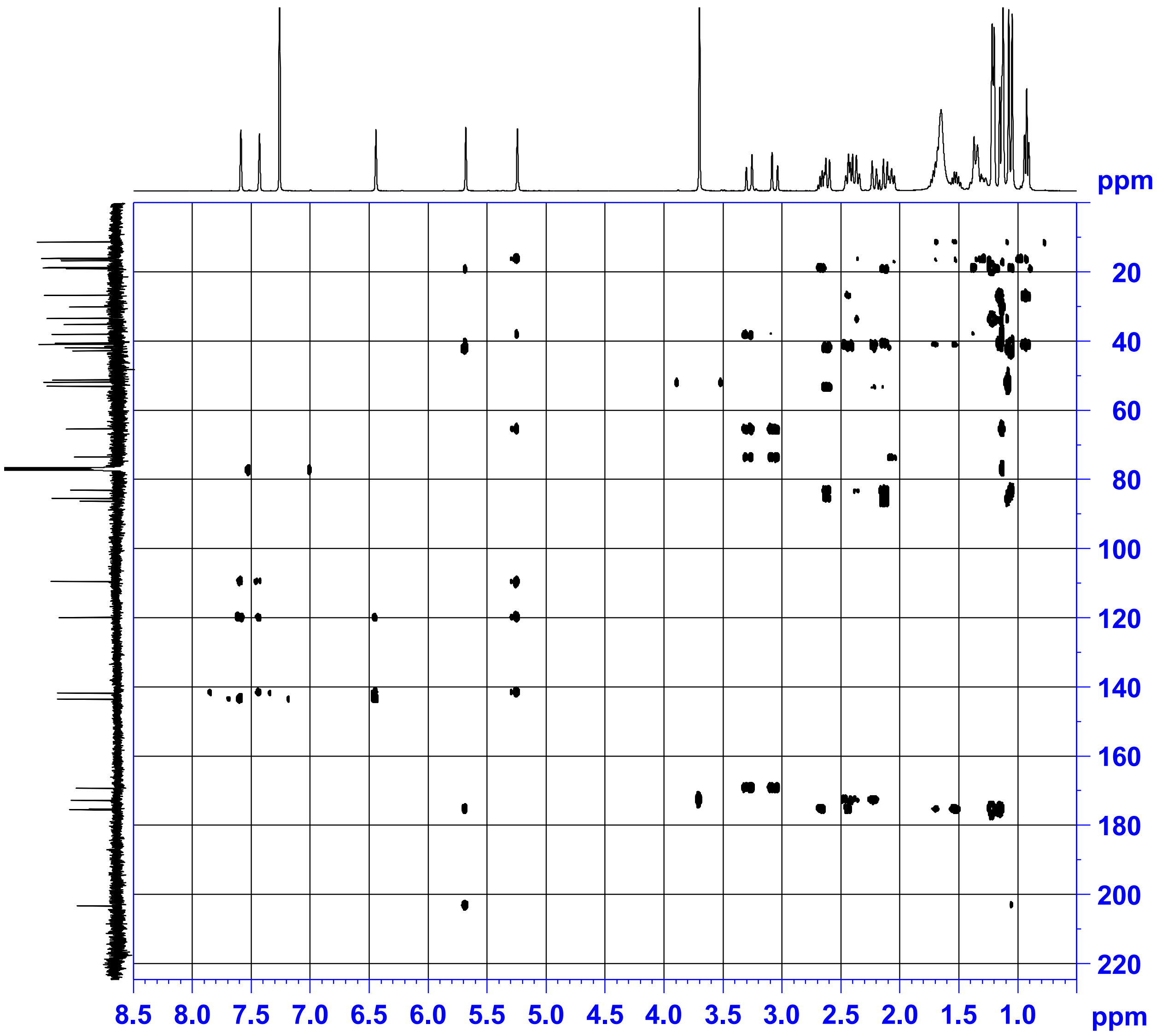

NAME	zq-17
EXPNO	6
PROCNO	1
Date_	20161220
Time	1.19
INSTRUM	spect
PROBHD	5 mm CPPBBO BB
PULPROG	hmbcgplpndqf
TD	4096
SOLVENT	CDCl3
NS	32
DS	16
SWH	5197.505 Hz
FIDRES	1.268922 Hz
AQ	0.3940852 sec
RG	208.5
DW	96.200 usec
DE	10.00 usec
TE	297.0 K
CNST2	145.0000000
CNST13	10.0000000
D0	0.00000300 sec
D1	1.50000000 sec
D2	0.00344828 sec
D6	0.05000000 sec
D16	0.00020000 sec
IN0	0.00002080 sec
======== CHANNEL f1 ========	
SFO1	400.1323208 MHz
NUC1	1H
P1	11.50 usec
P2	23.00 usec
ND0	2
TD	128
SFO1	100.6233 MHz
FIDRES	187.800476 Hz
SW	238.896 ppm
FnMODE	QF
SI	2048
SF	400.1300055 MHz
WDW	SINE
SSB	0
LB	0.00 Hz
GB	0
PC	1.40
SI	1024
MC2	QF
SF	100.6127570 MHz
WDW	SINE
SSB	0
LB	0.00 Hz
GB	0

HMBC spectrum of Krishnolide A (**1**) in $CDCl_3$

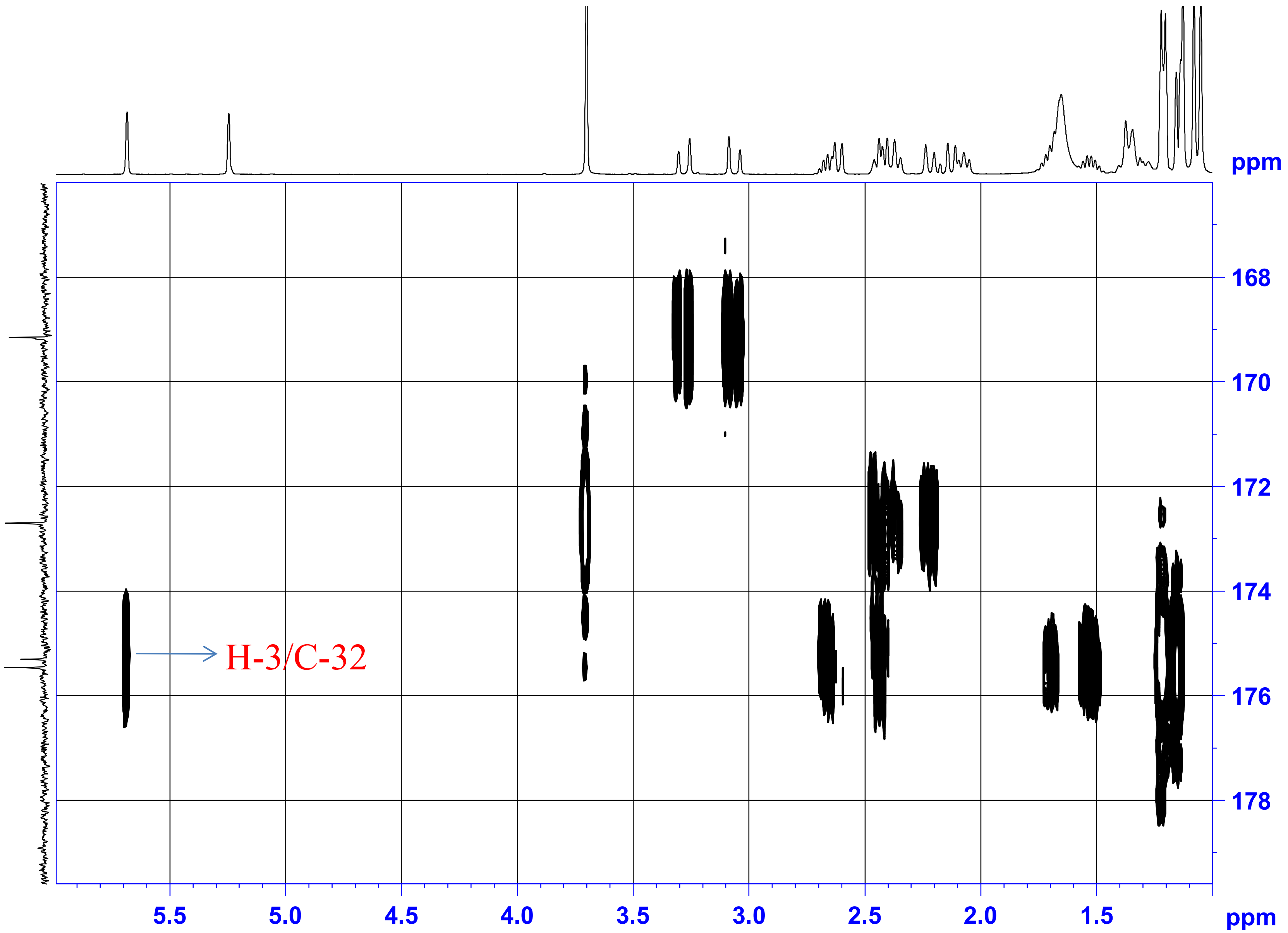

HMBC spectrum of Krishnolide A (**1**) in $CDCl_3$

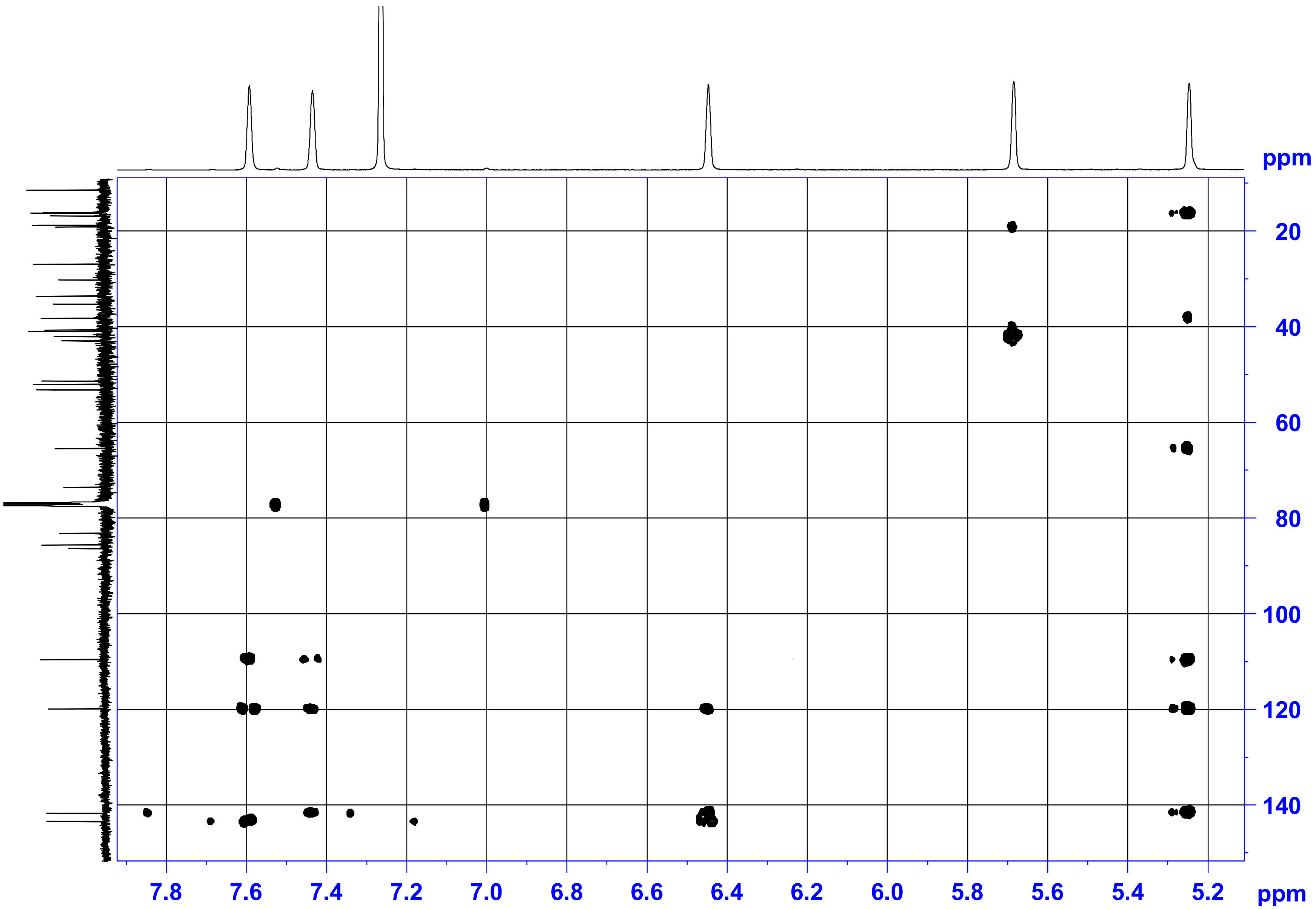

HMBC spectrum of Krishnolide A (**1**) in $CDCl_3$

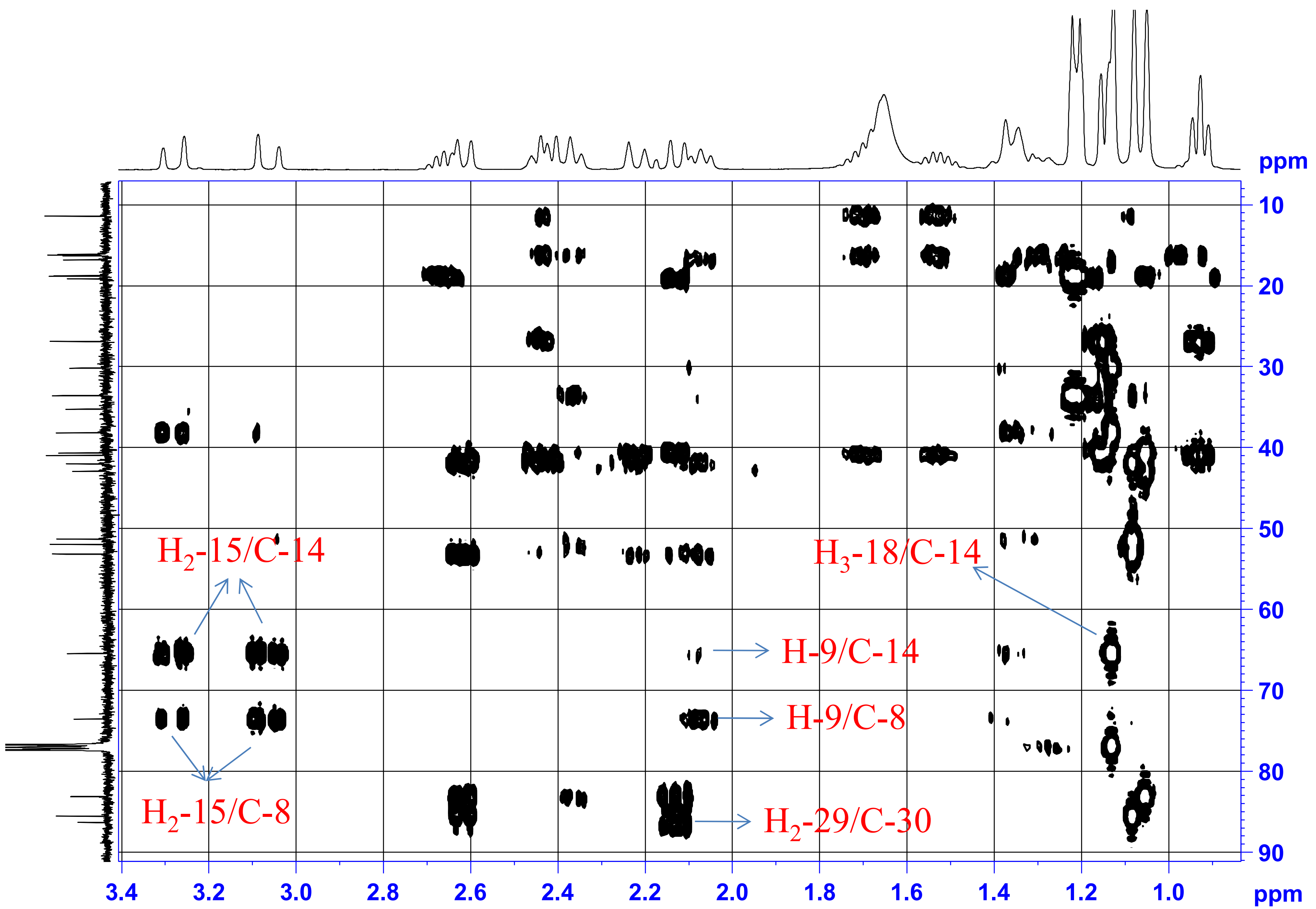

HMBC spectrum of Krishnolide A (**1**) in $CDCl_3$

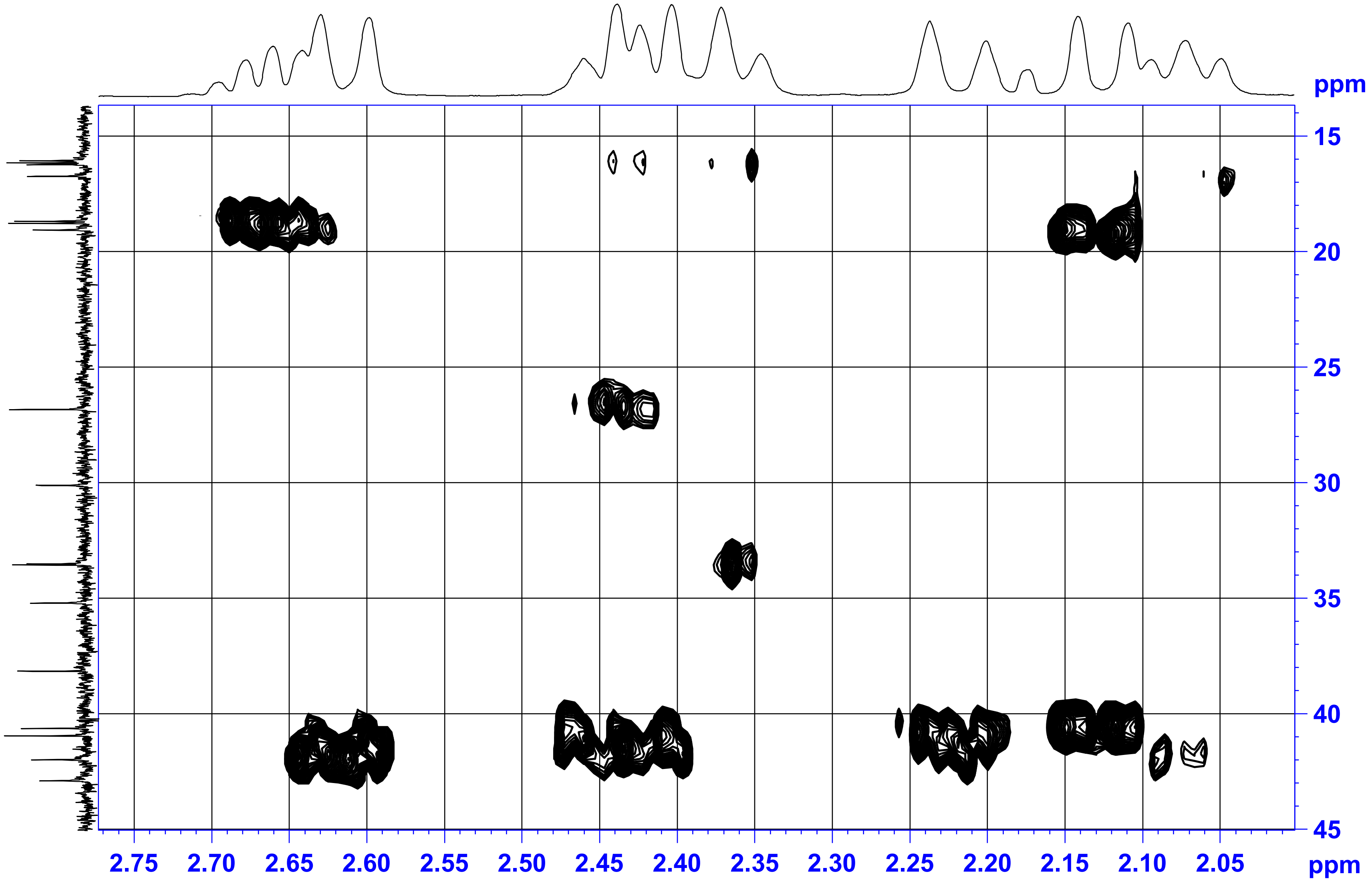

HMBC spectrum of Krishnolide A (**1**) in $CDCl_3$

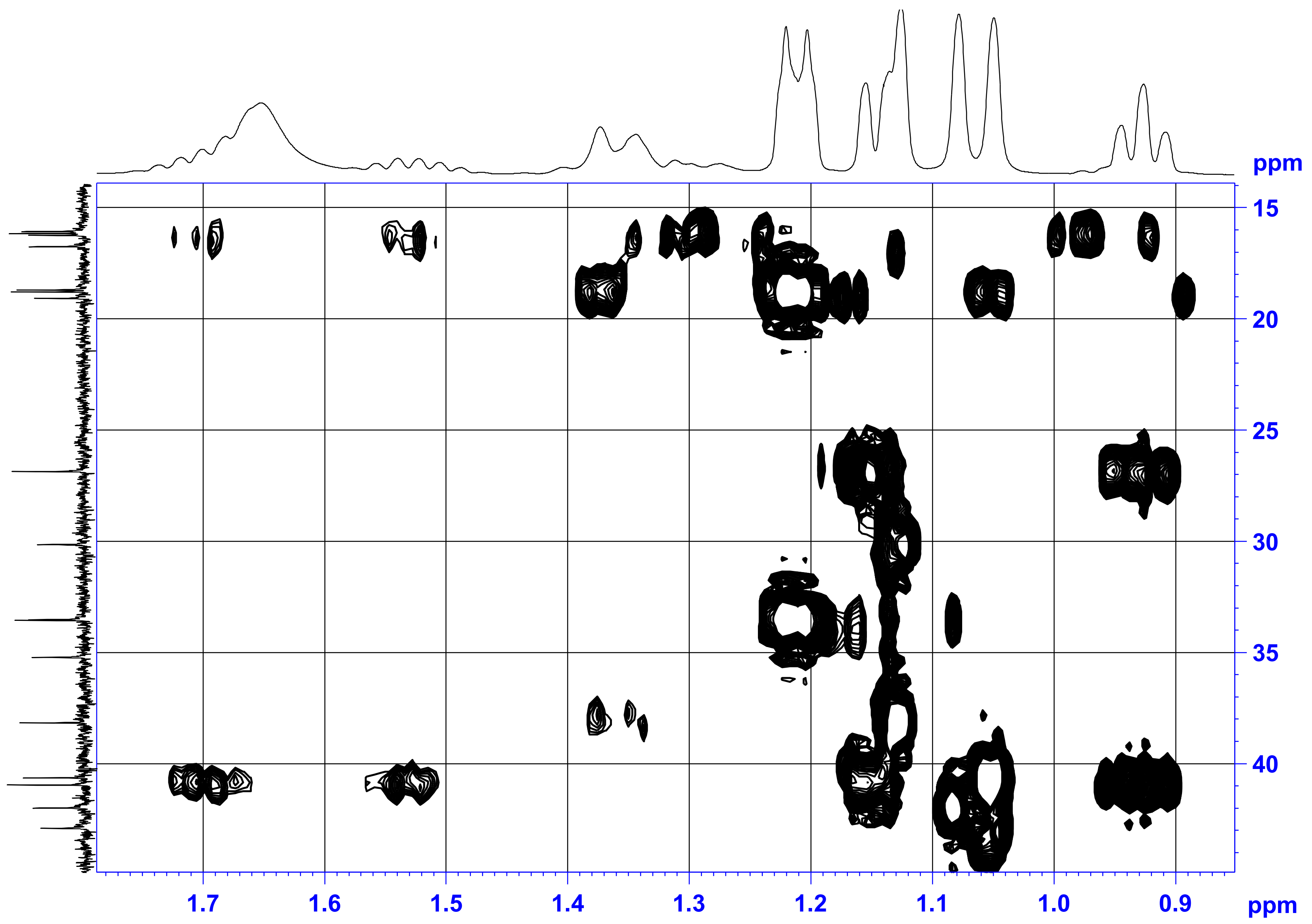

NOESY spectrum of Krishnolide A (**1**) in $CDCl_3$

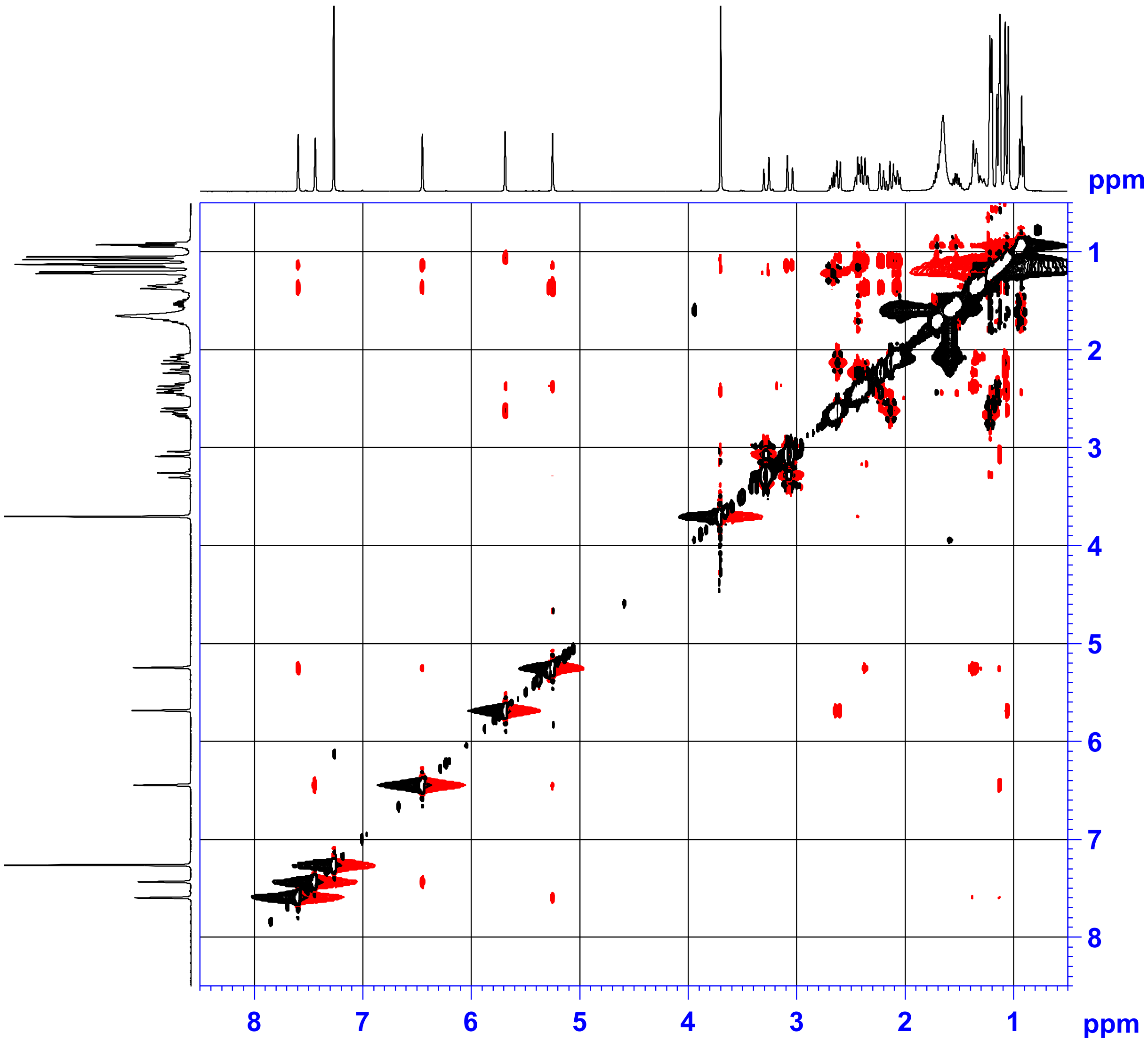

NAME	zq-17	
EXPNO	7	
PROCNO	1	
Date_	20161220	
Time	3.35	
INSTRUM	spect	
PROBHD	5 mm CPPBBO BB	
PULPROG	noesygpphpp	
TD	2048	
SOLVENT	CDCl3	
NS	16	
DS	32	
SWH	4000.000	Hz
FIDRES	1.953125	Hz
AQ	0.2560500	sec
RG	208.5	
DW	125.000	usec
DE	10.00	usec
TE	297.0	K
D0	0.00011036	sec
D1	1.99385595	sec
D8	0.30000001	sec
D11	0.03000000	sec
D12	0.00002000	sec
D16	0.00020000	sec
IN0	0.00025000	sec

======== CHANNEL f1 ========

SFO1	400.1318006	MHz
NUC1	1H	
P1	11.50	usec
P2	23.00	usec
P17	2500.00	usec
ND0	1	
TD	256	
SFO1	400.1318	MHz
FIDRES	15.625000	Hz
SW	9.997	ppm
FnMODE	States-TPPI	
SI	1024	
SF	400.1300055	MHz
WDW	QSINE	
SSB	2	
LB	0.00	Hz
GB	0	
PC	1.00	
SI	1024	
MC2	States-TPPI	
SF	400.1300055	MHz
WDW	QSINE	
SSB	2	
LB	0.00	Hz
GB	0	

NOESY spectrum of Krishnolide A (**1**) in $CDCl_3$

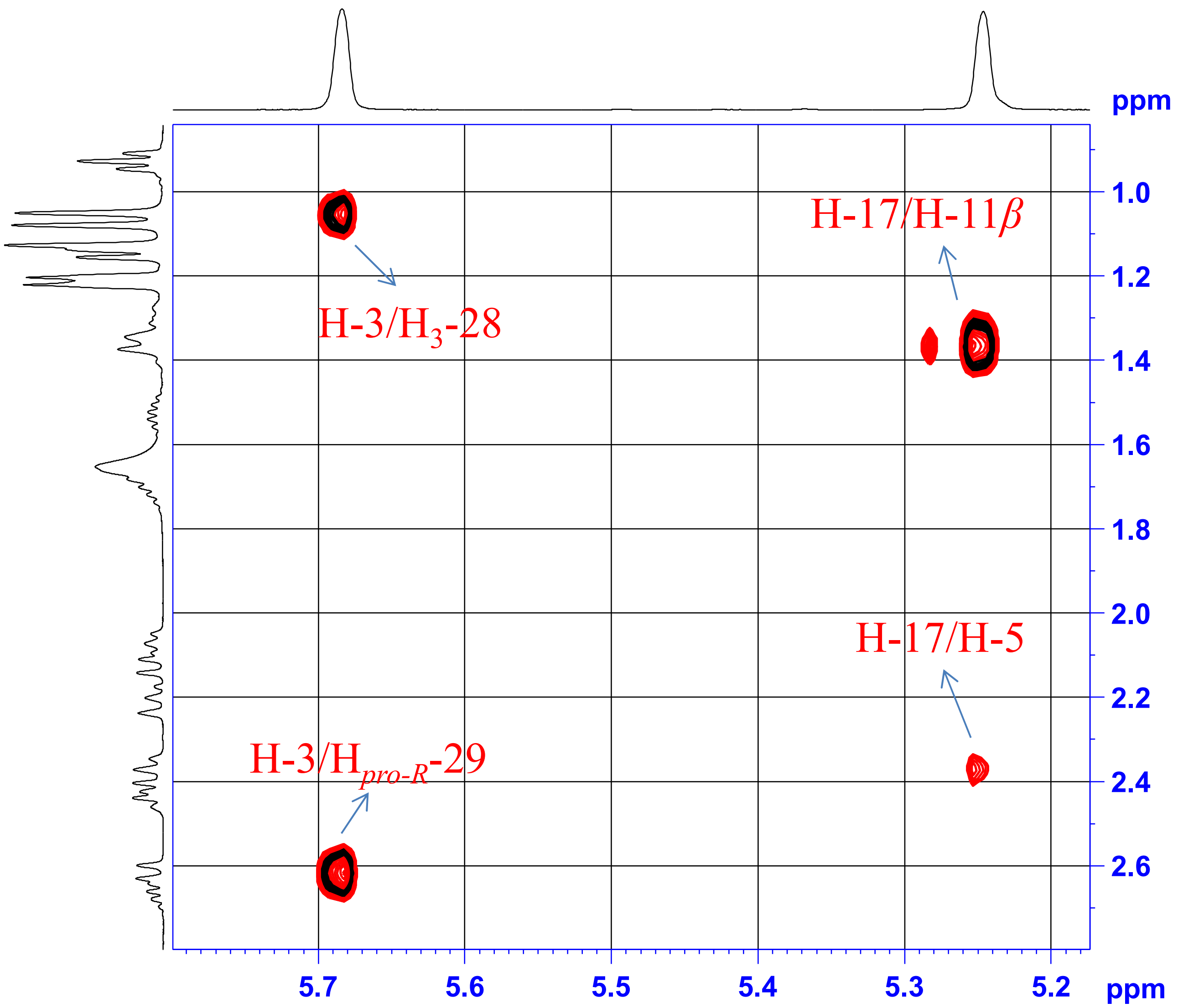

NOESY spectrum of Krishnolide A (**1**) in $CDCl_3$

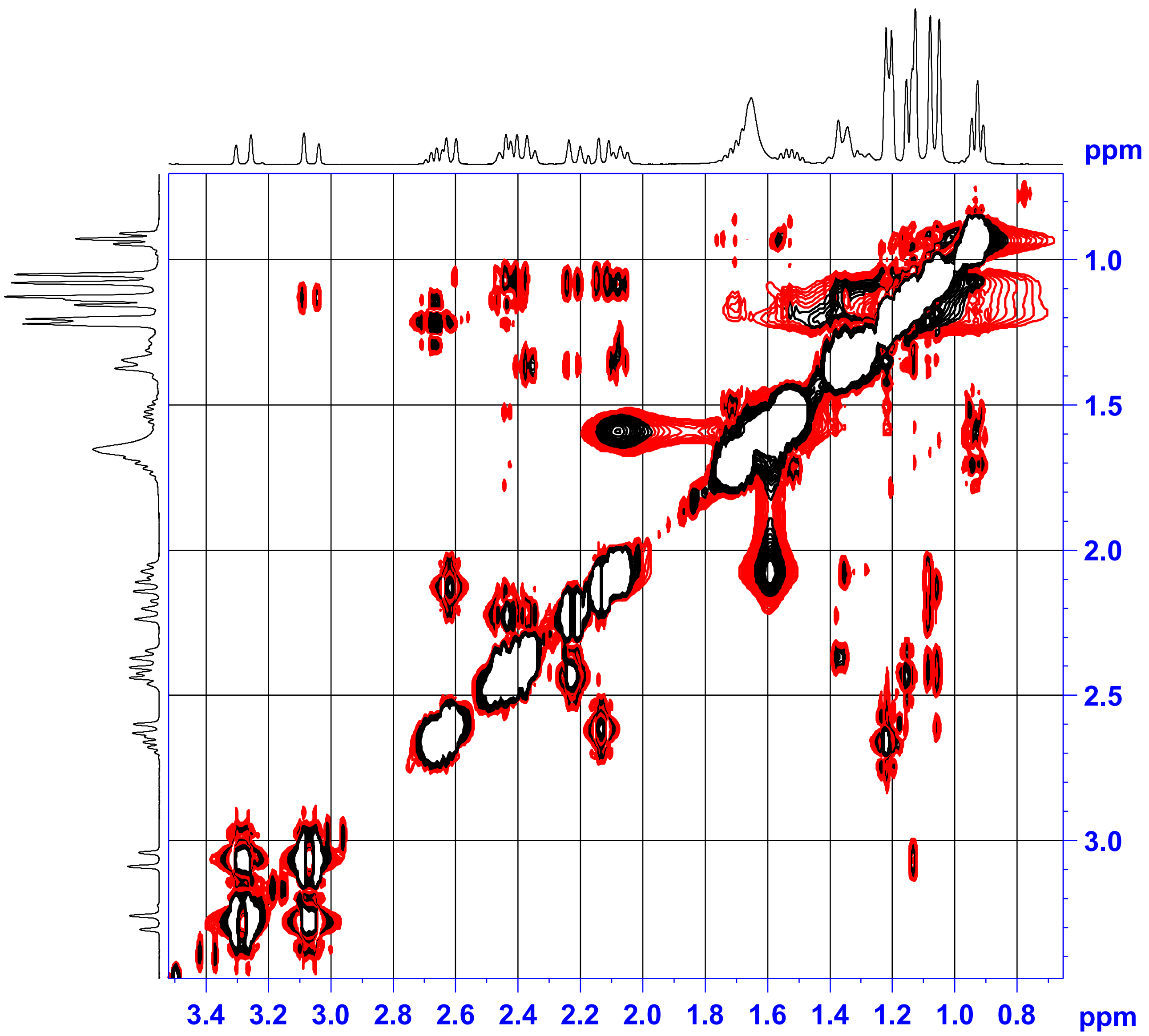

NOESY spectrum of Krishnolide A (**1**) in $CDCl_3$

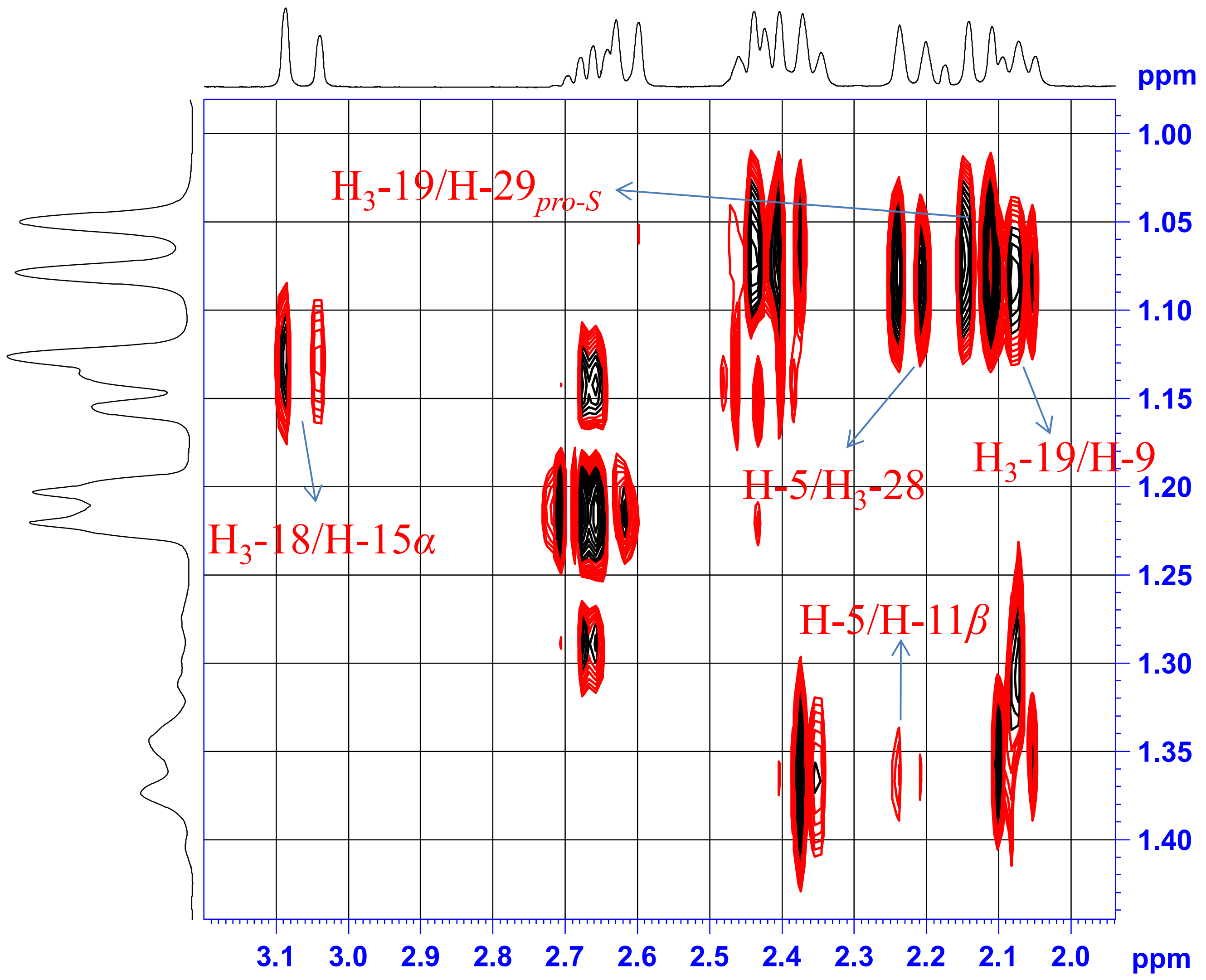

NOESY spectrum of Krishnolide A (**1**) in $CDCl_3$

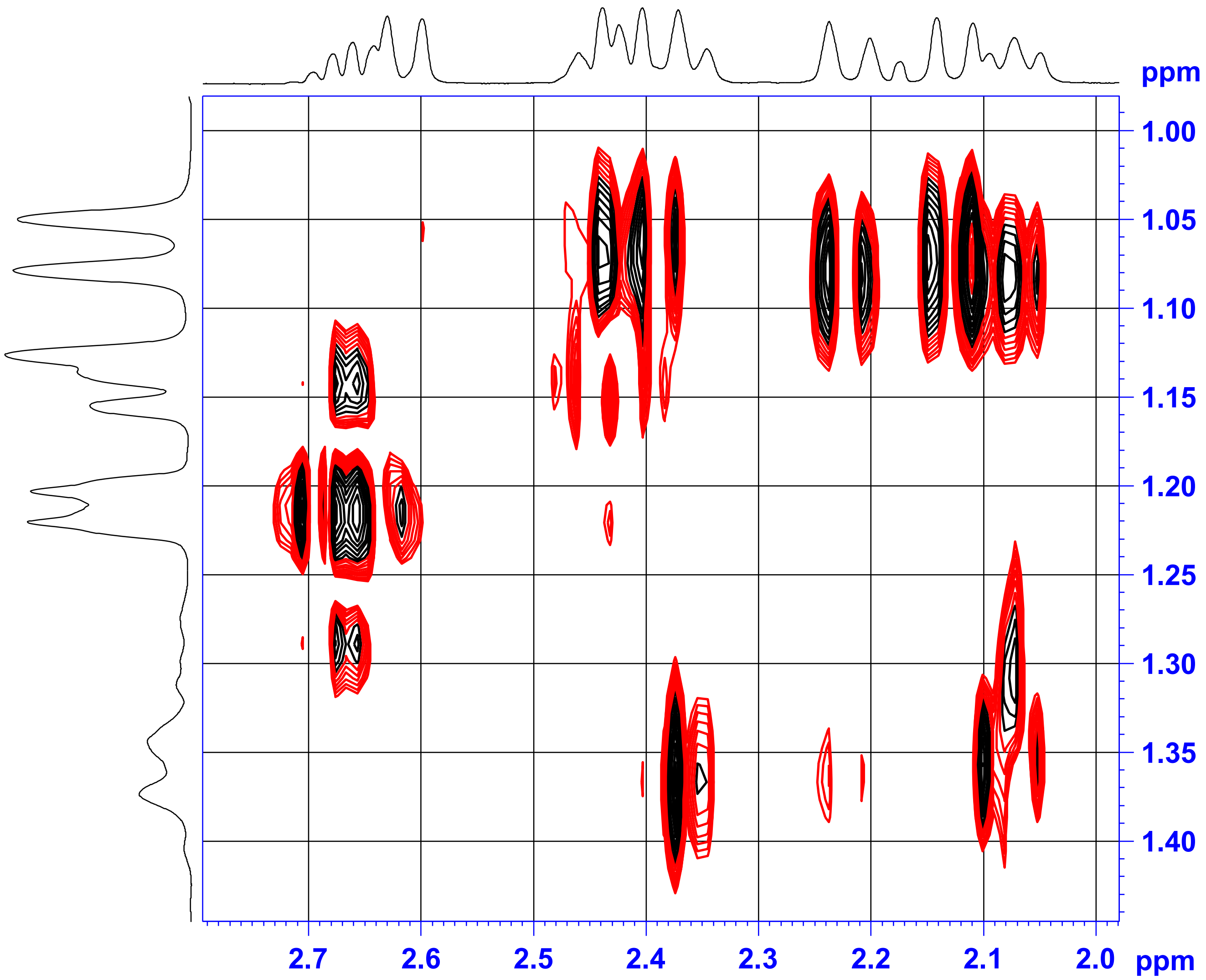

^{1}H NMR (400 MHz) spectrum of Krishnolide A (**1**) in DMSO-d_6

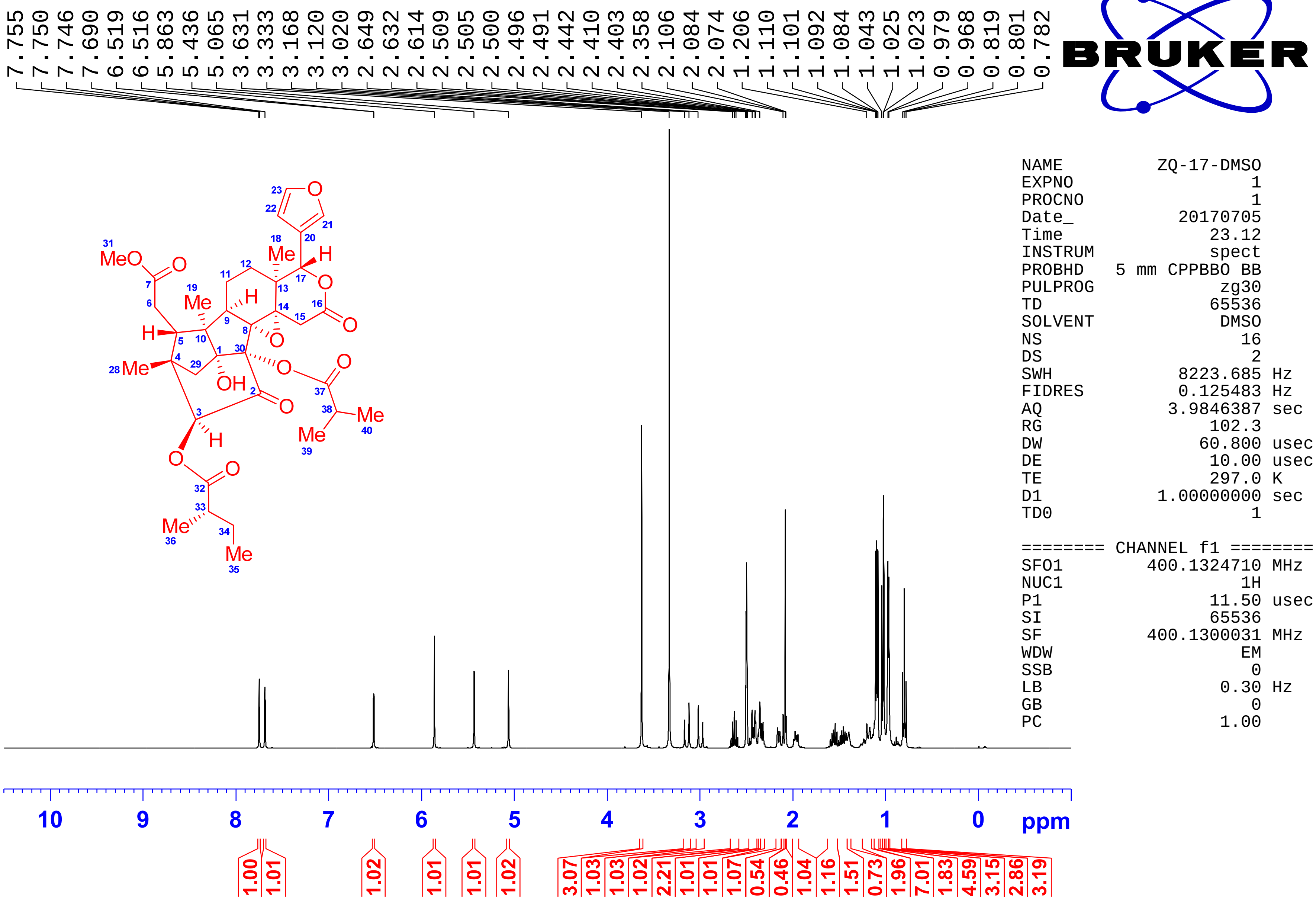

^1H NMR (400 MHz) spectrum of Krishnolide A (**1**) in DMSO-d_6

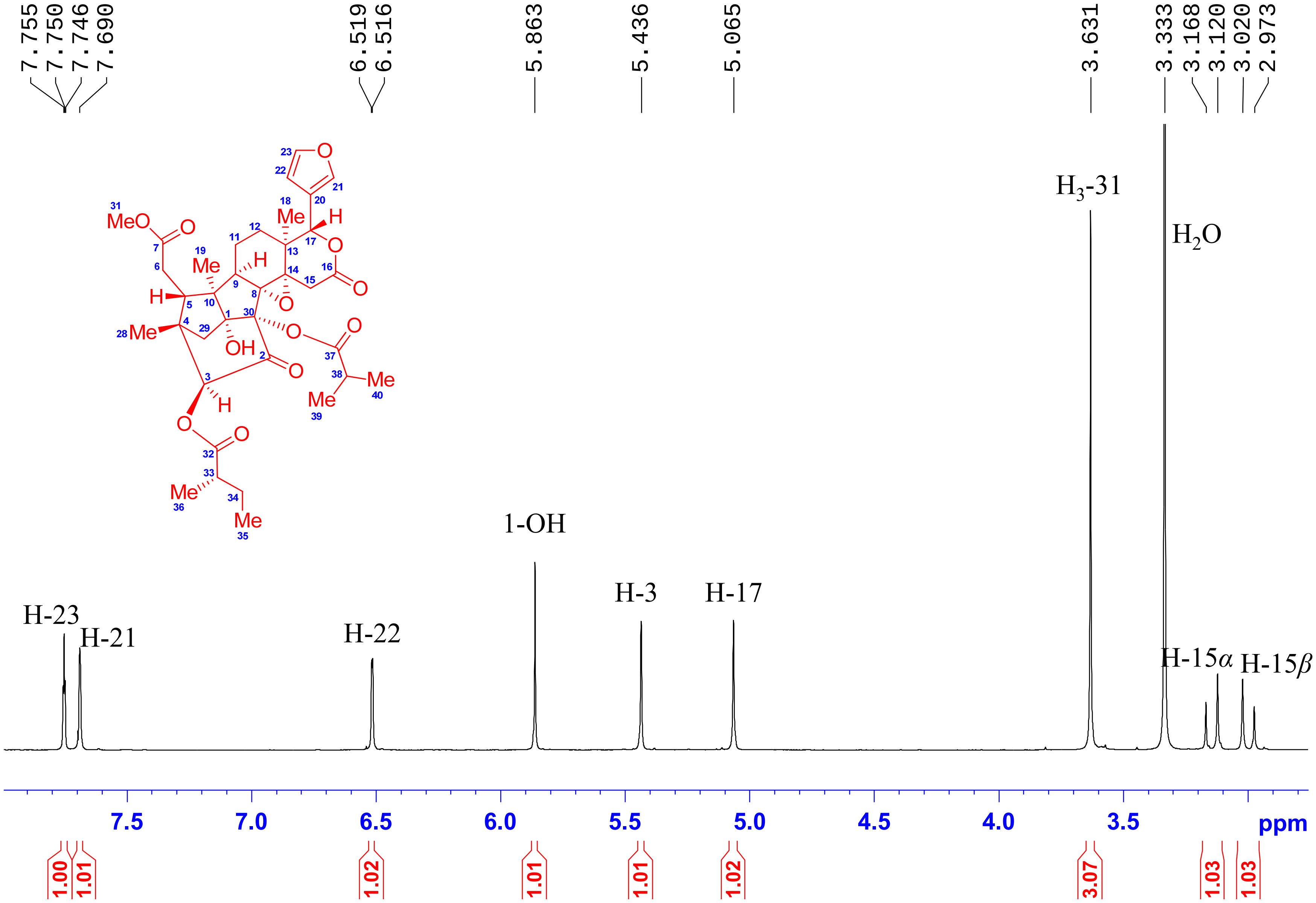

^{1}H NMR (400 MHz) spectrum of Krishnolide A (**1**) in DMSO-d_6

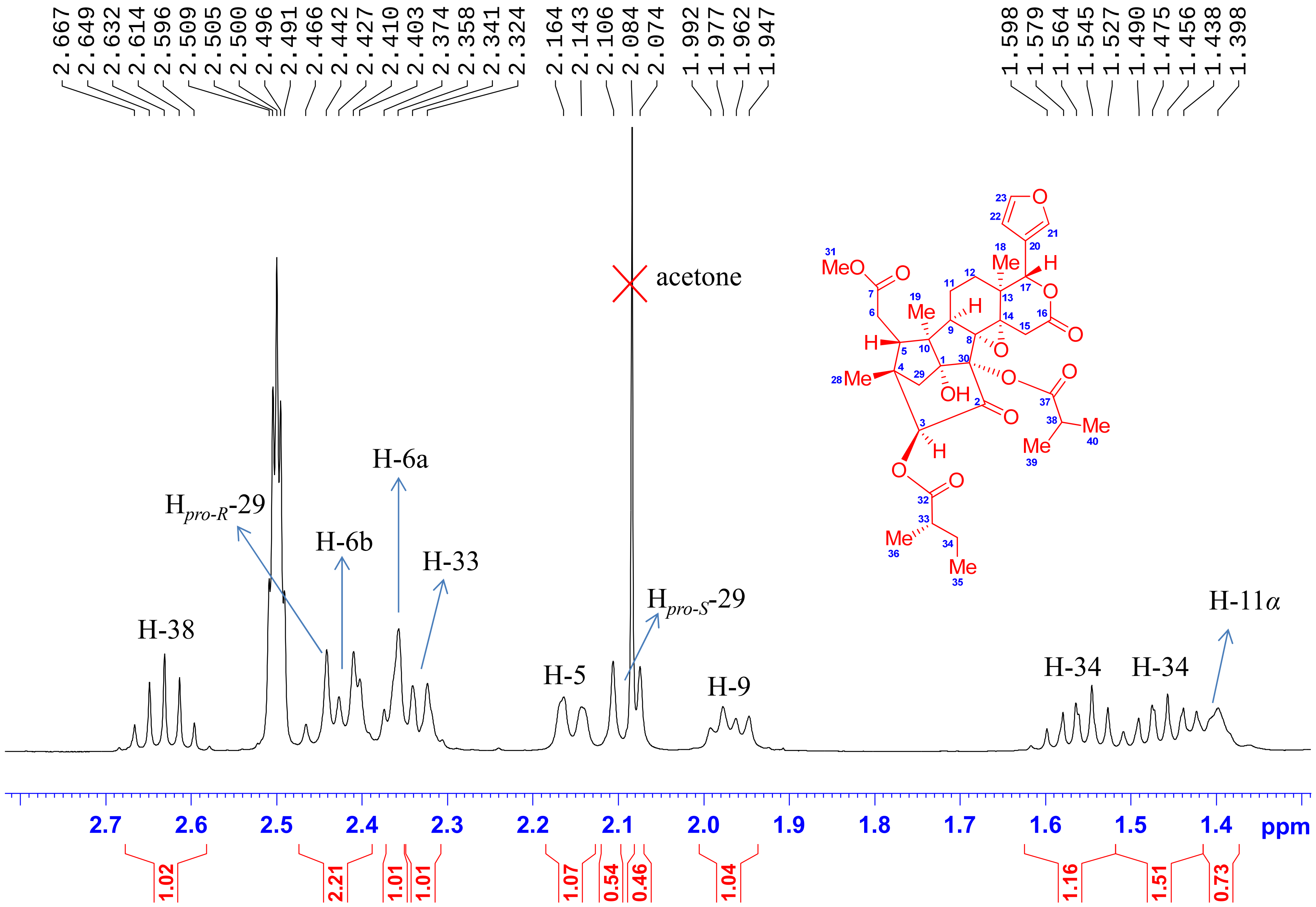

^{1}H NMR (400 MHz) spectrum of Krishnolide A (**1**) in DMSO-d_6

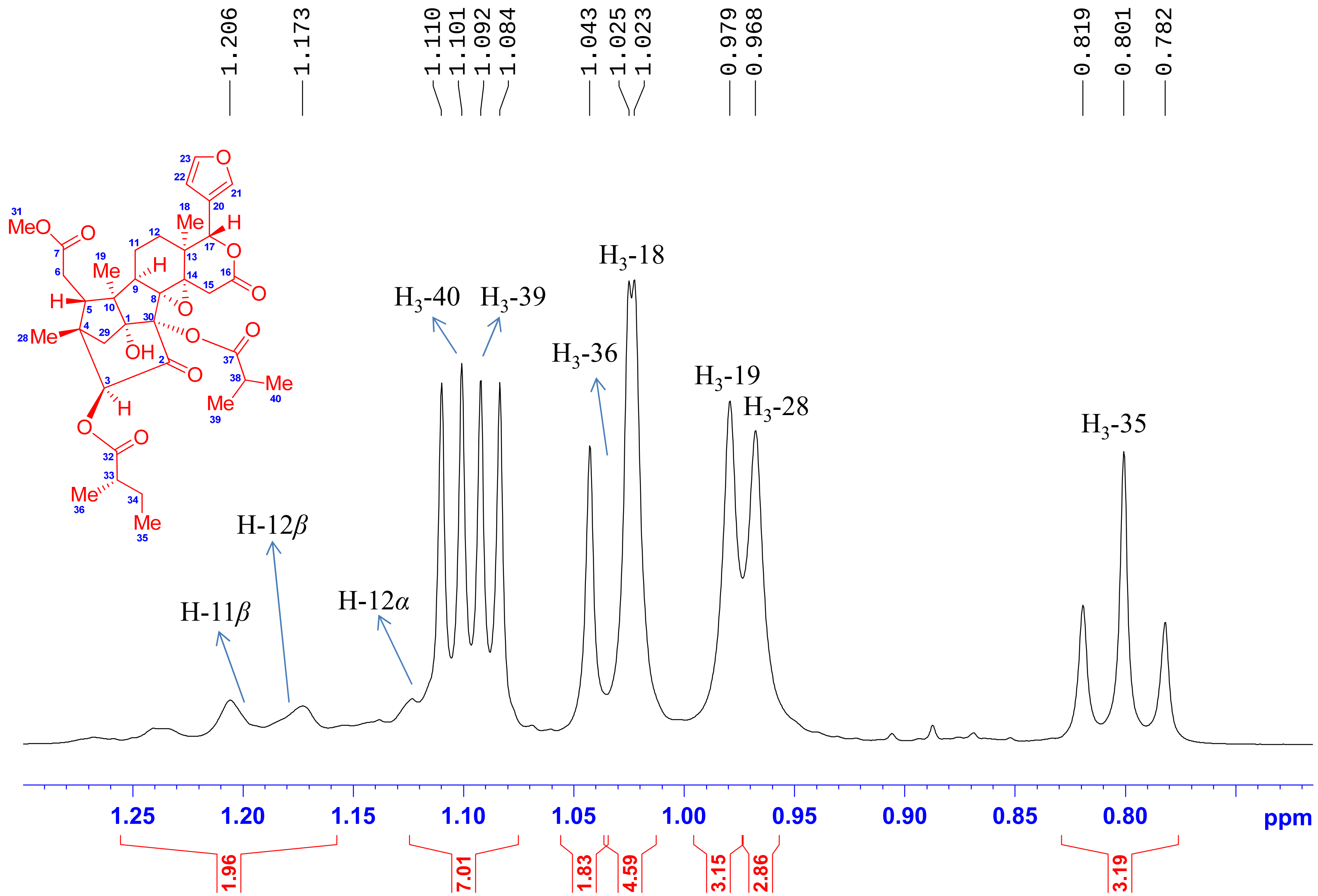

^{13}C NMR (100 MHz) spectrum of Krishnolide A (**1**) in DMSO-d_6

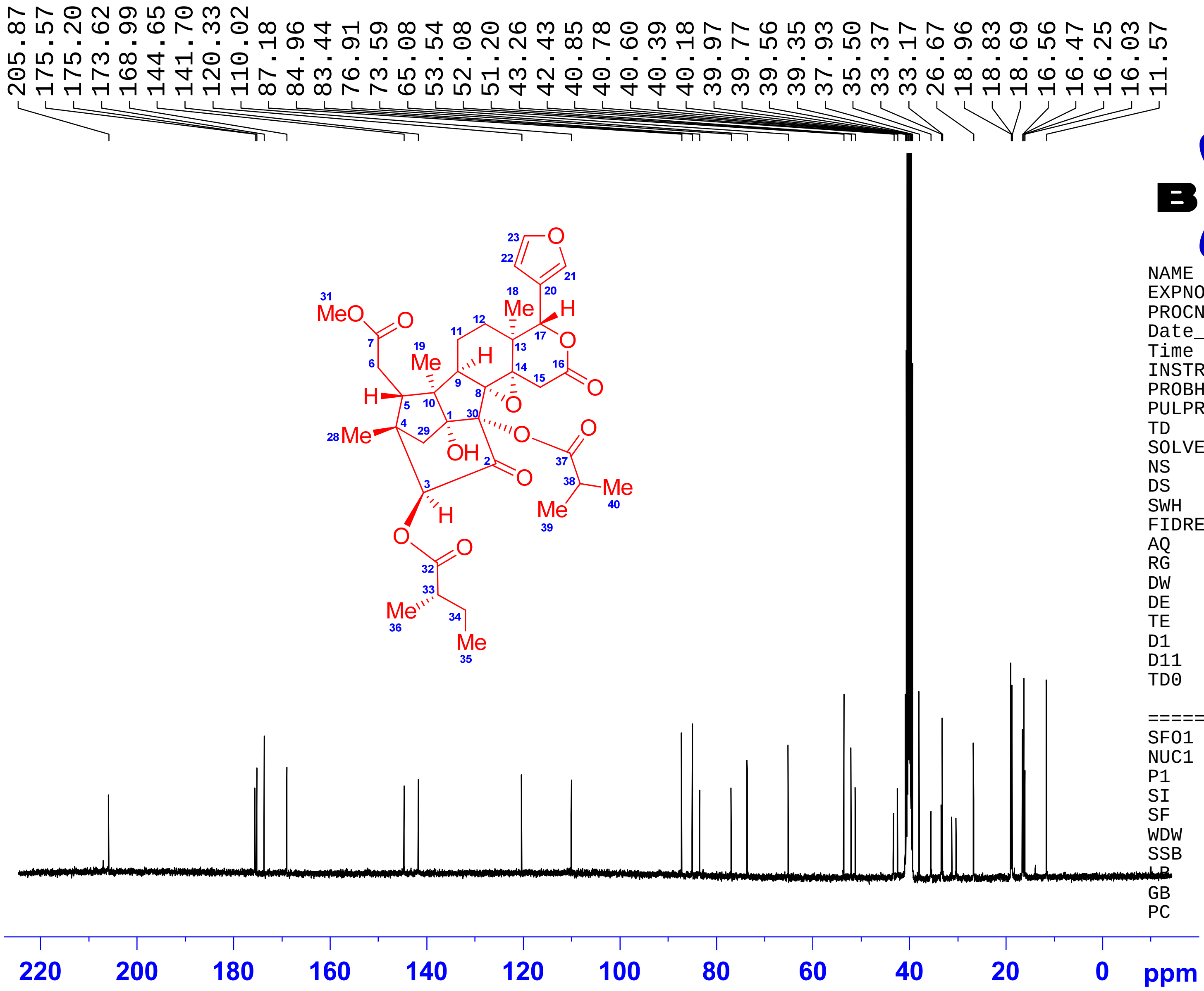


```
NAME          ZQ-17-DMSO
EXPNO                  2
PROCNO                 1
Date_           20170706
Time                0.12
INSTRUM            spect
PROBHD   5 mm CPPBBO BB
PULPROG          zgpg30
TD                 65536
SOLVENT             DMSO
NS                  1024
DS                     4
SWH            24038.461 Hz
FIDRES          0.366798 Hz
AQ             1.3631988 sec
RG                117.37
DW                20.800 usec
DE                 18.00 usec
TE                 297.0 K
D1            2.00000000 sec
D11           0.03000000 sec
TD0                    1

======== CHANNEL f1 ========
SFO1         100.6233324 MHz
NUC1                 13C
P1                 10.00 usec
SI                 32768
SF           100.6127685 MHz
WDW                   EM
SSB                    0
LB                  1.00 Hz
GB                     0
PC                  1.40
```

^{13}C NMR (100 MHz) spectrum of Krishnolide A (**1**) in DMSO-d_6

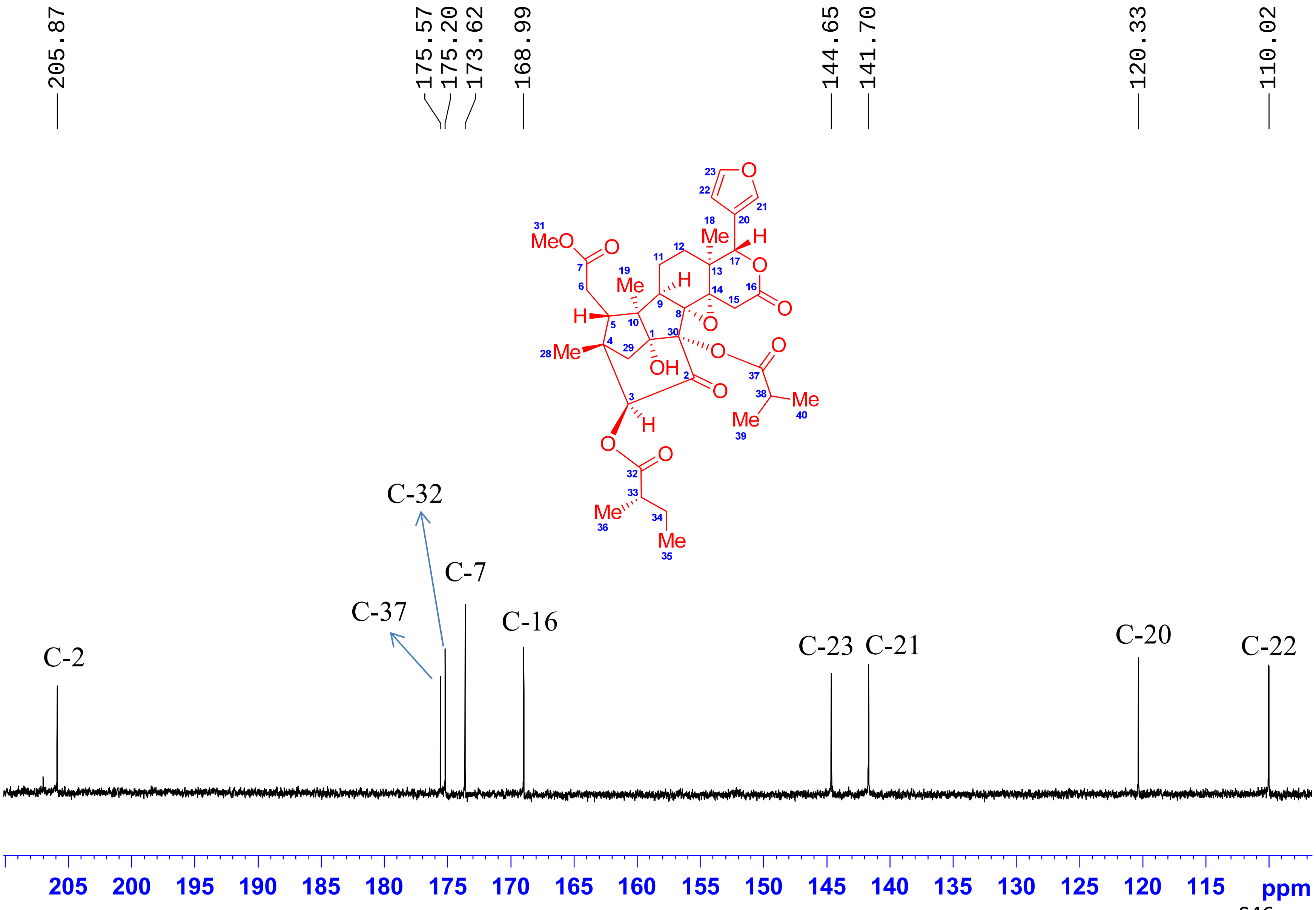

^{13}C NMR (100 MHz) spectrum of Krishnolide A (**1**) in DMSO-d_6

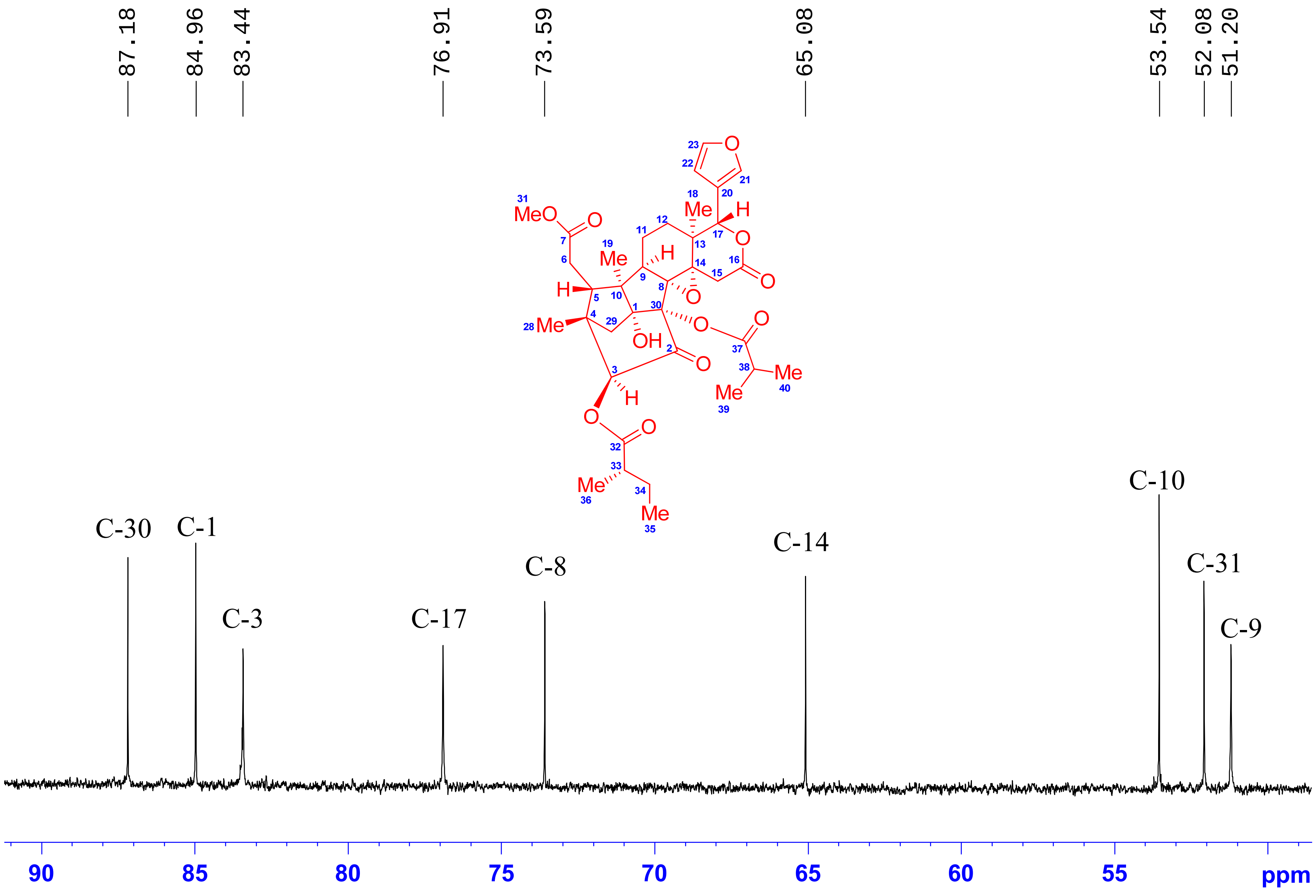

^{13}C NMR (100 MHz) spectrum of Krishnolide A (**1**) in DMSO-d_6

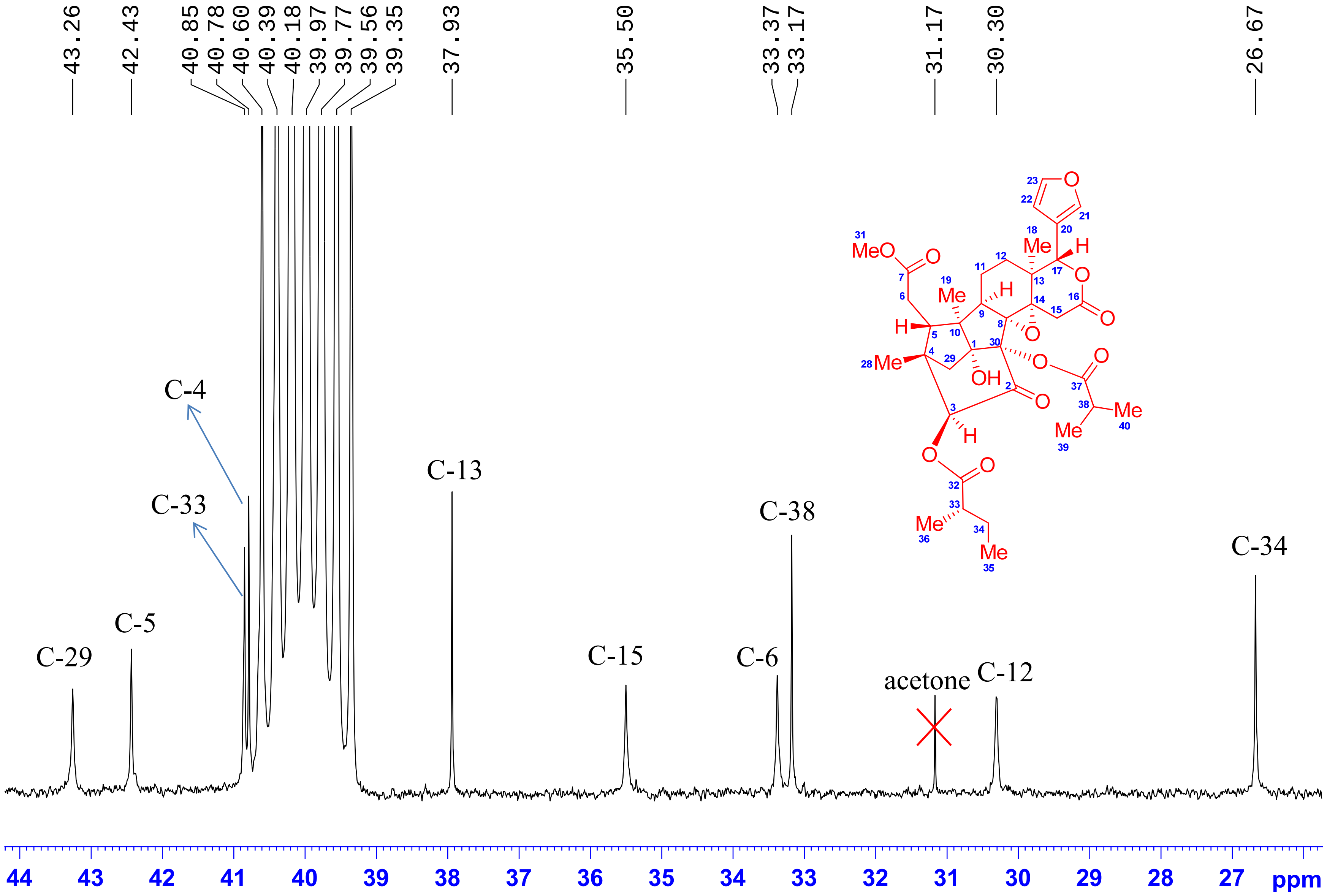

^{13}C NMR (100 MHz) spectrum of Krishnolide A (**1**) in DMSO-d_6

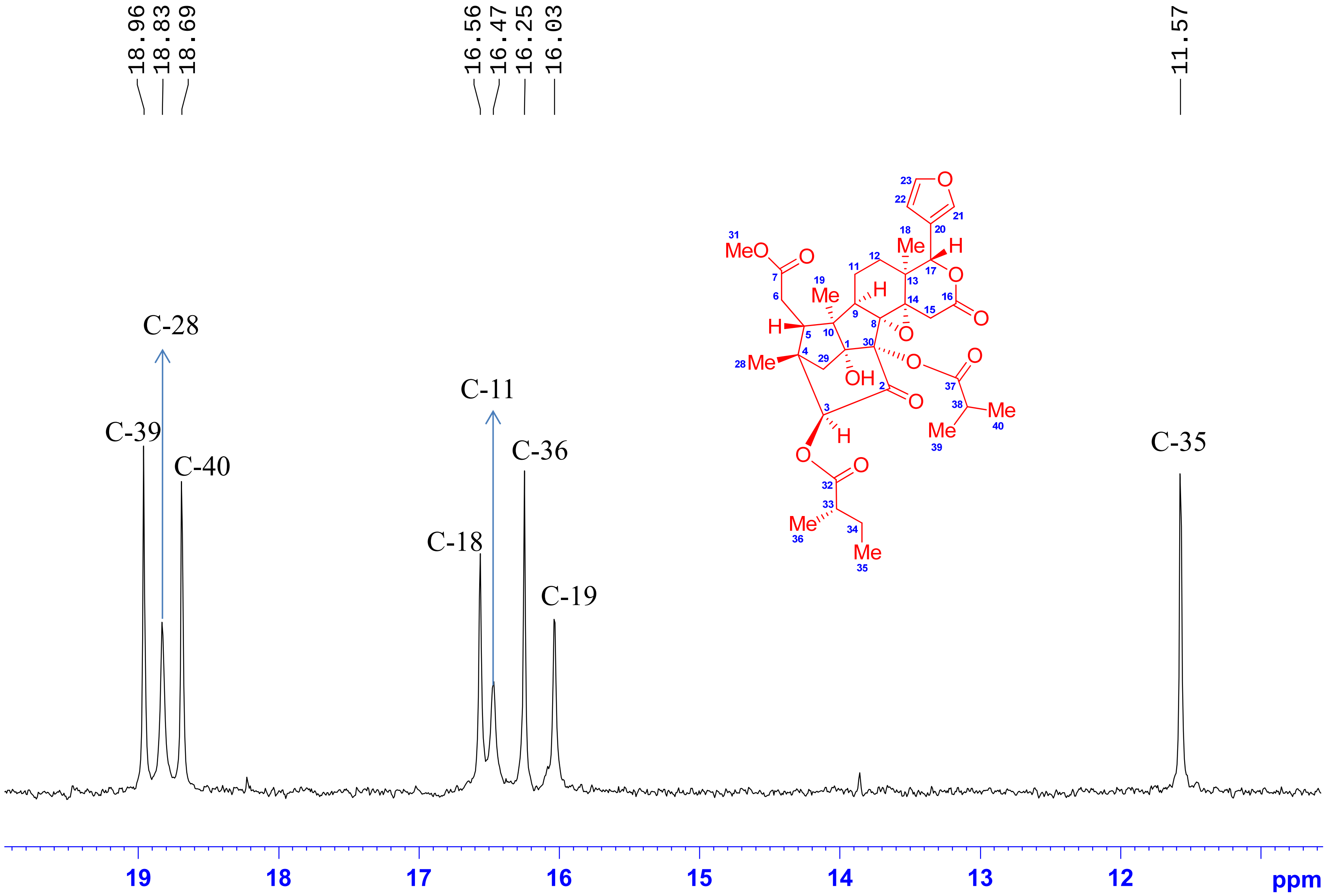

DEPT 135 spectrum of Krishnolide A (**1**) in DMSO-d_6

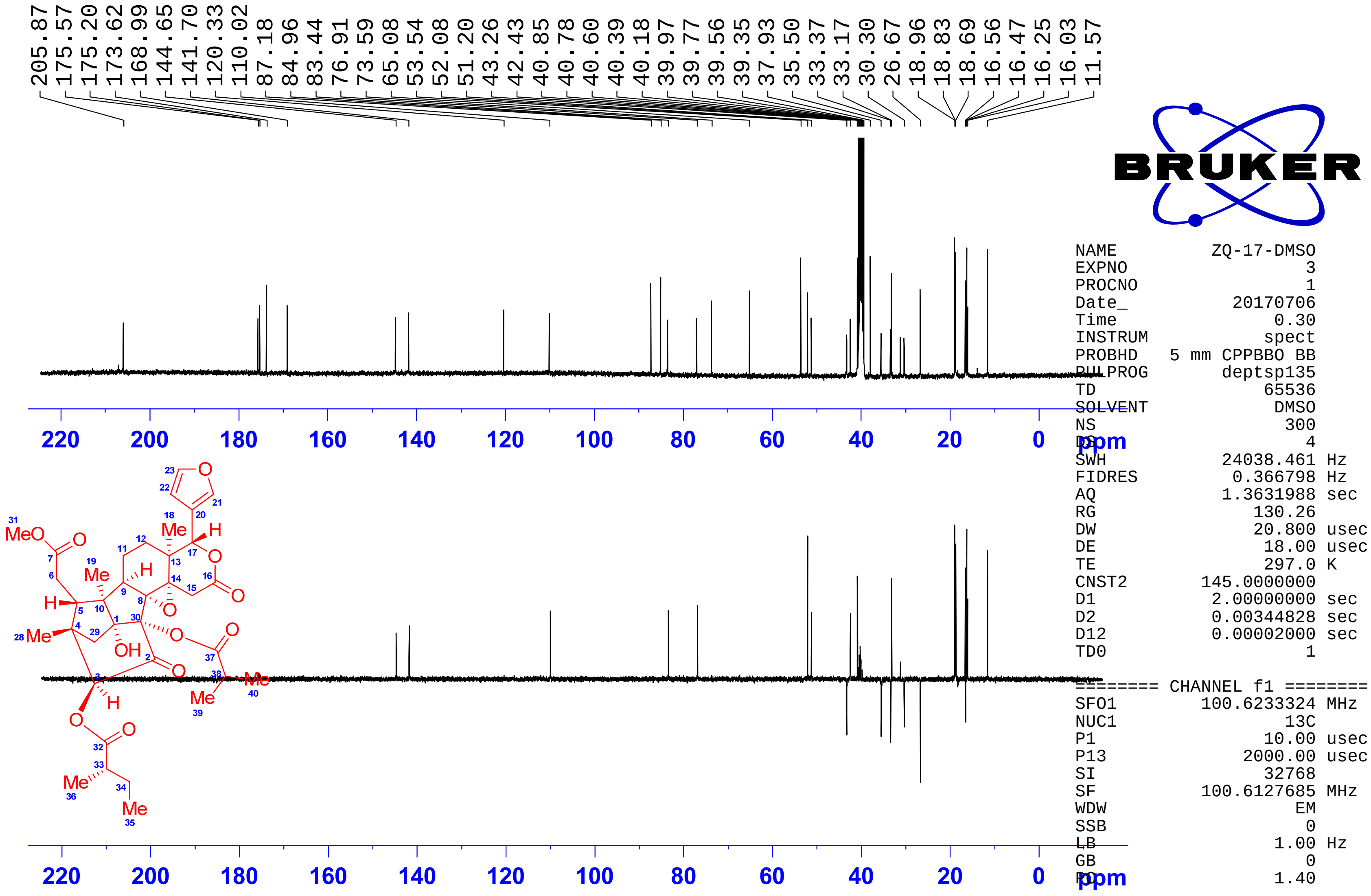

DEPT 135 spectrum of Krishnolide A (**1**) in DMSO-d_6

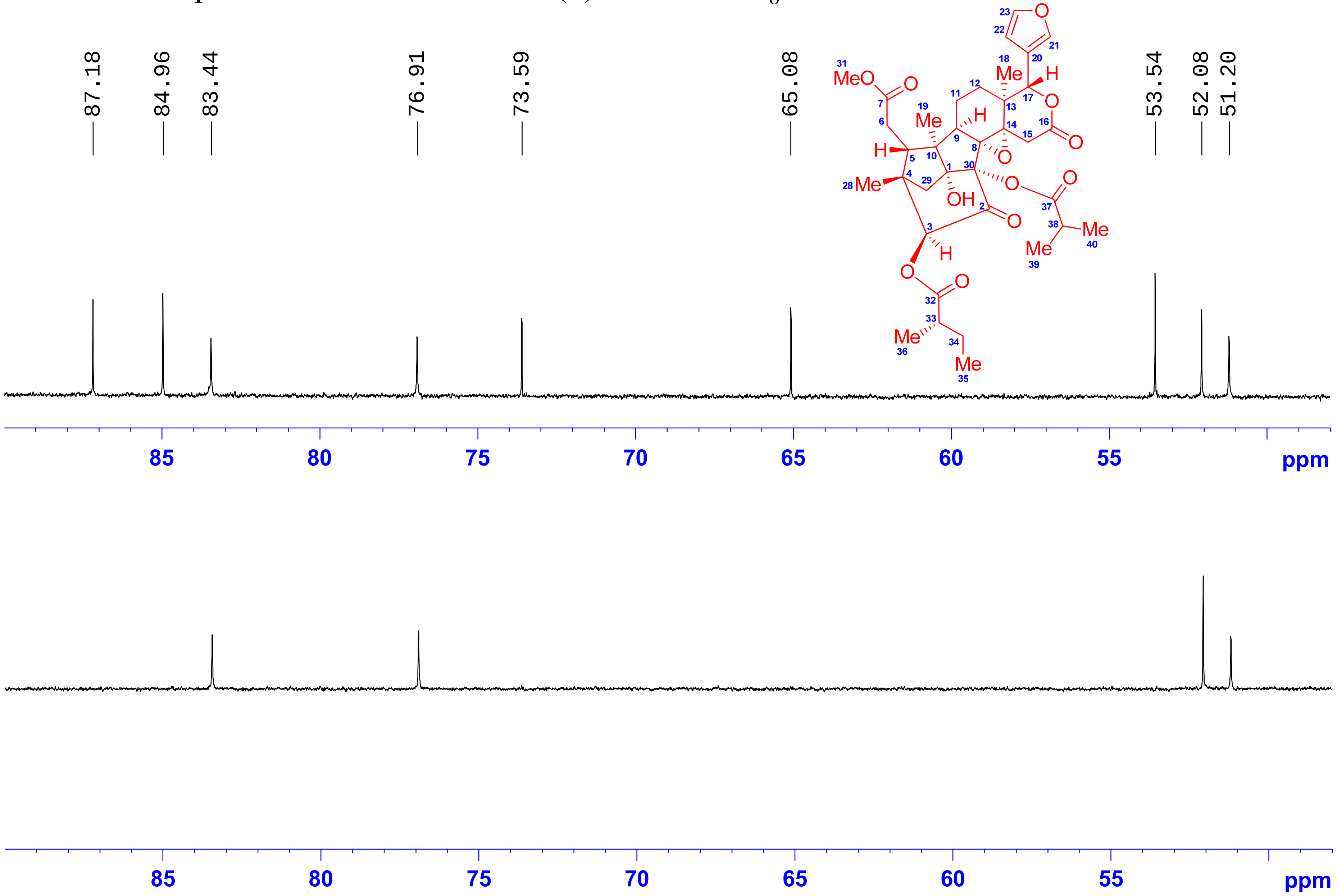

DEPT 135 spectrum of Krishnolide A (**1**) in DMSO-d_6

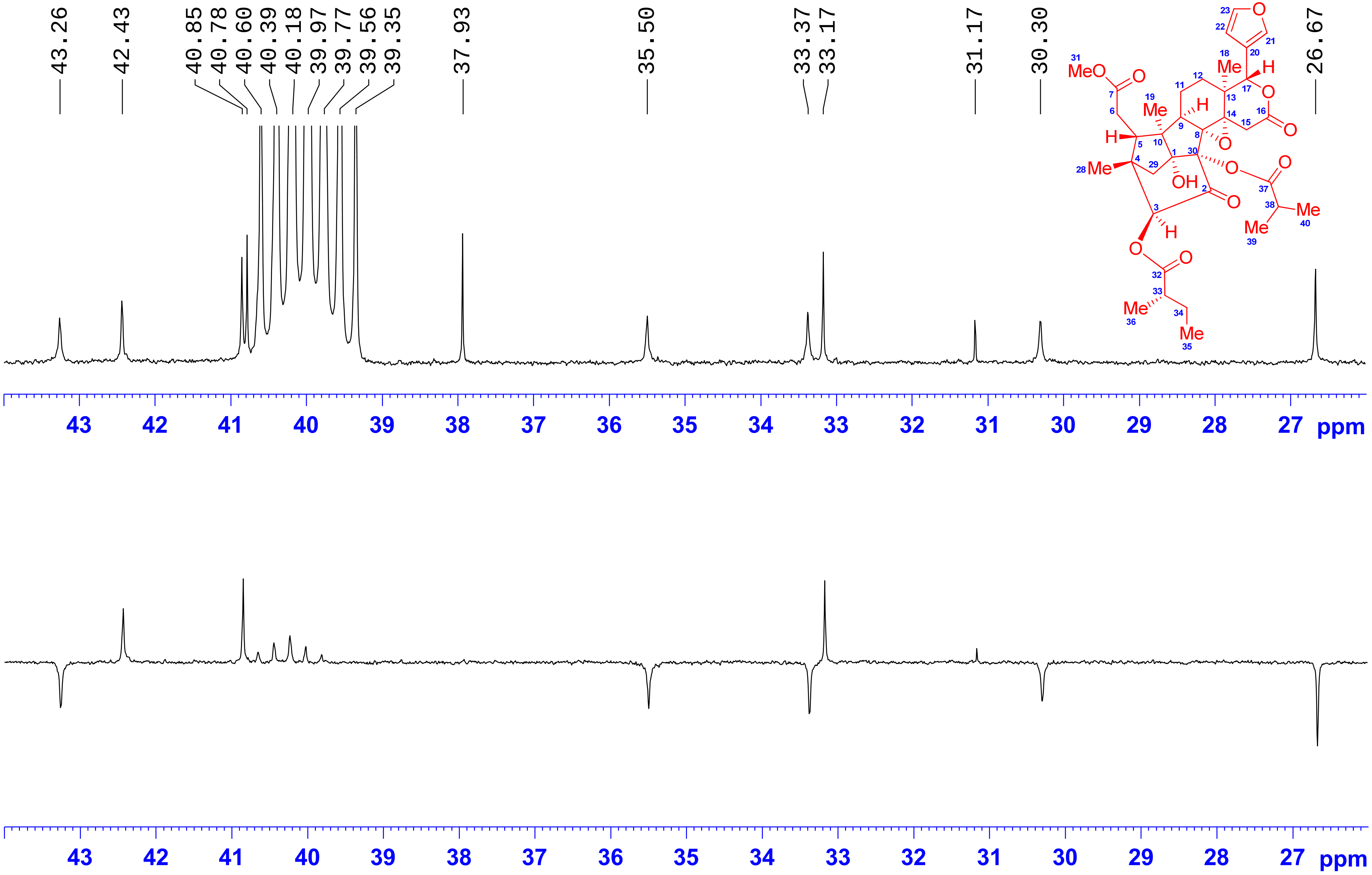

DEPT 135 spectrum of Krishnolide A (**1**) in DMSO-d_6

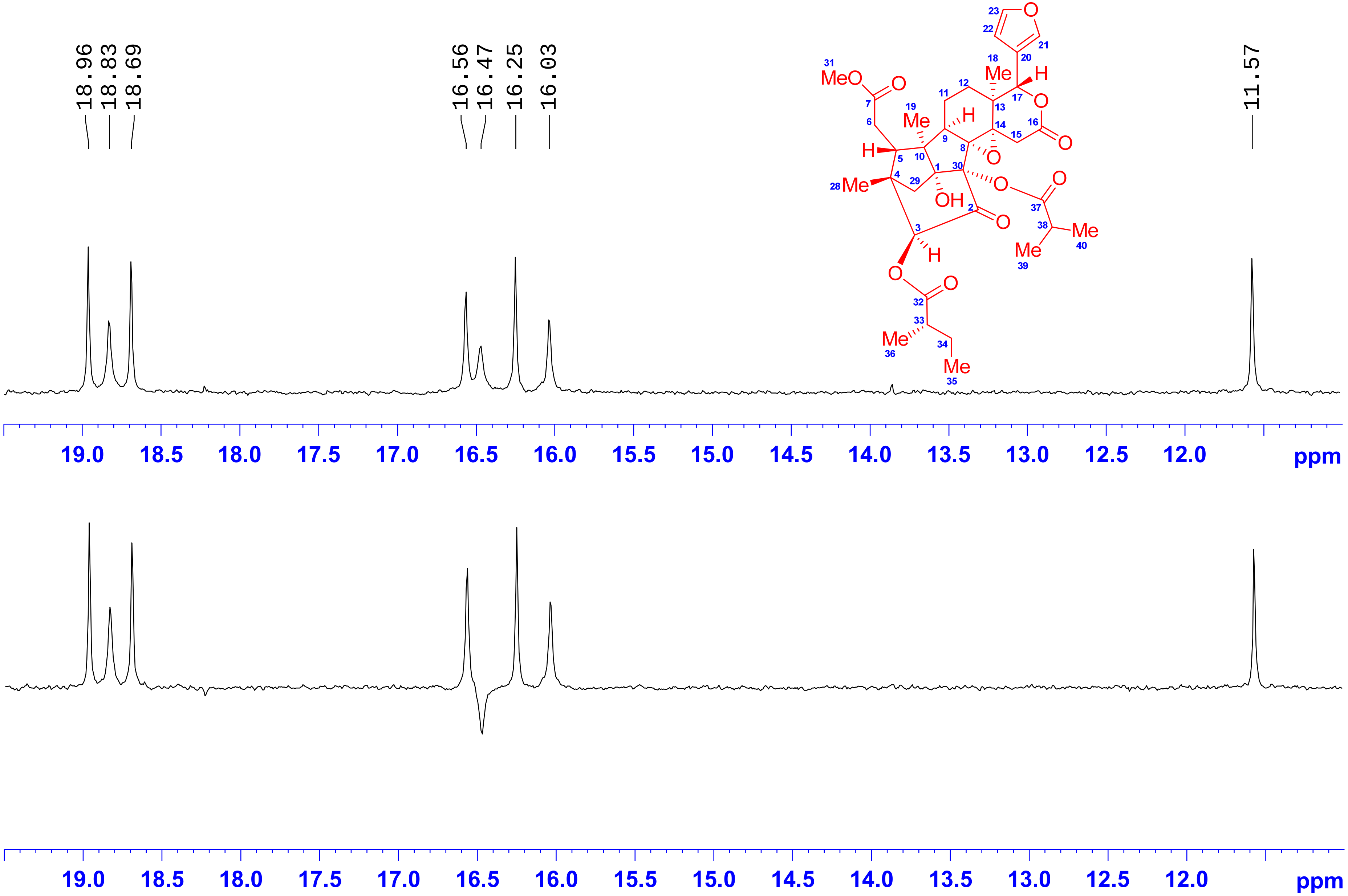

^1H-^1H COSY spectrum of Krishnolide A (**1**) in DMSO-d_6

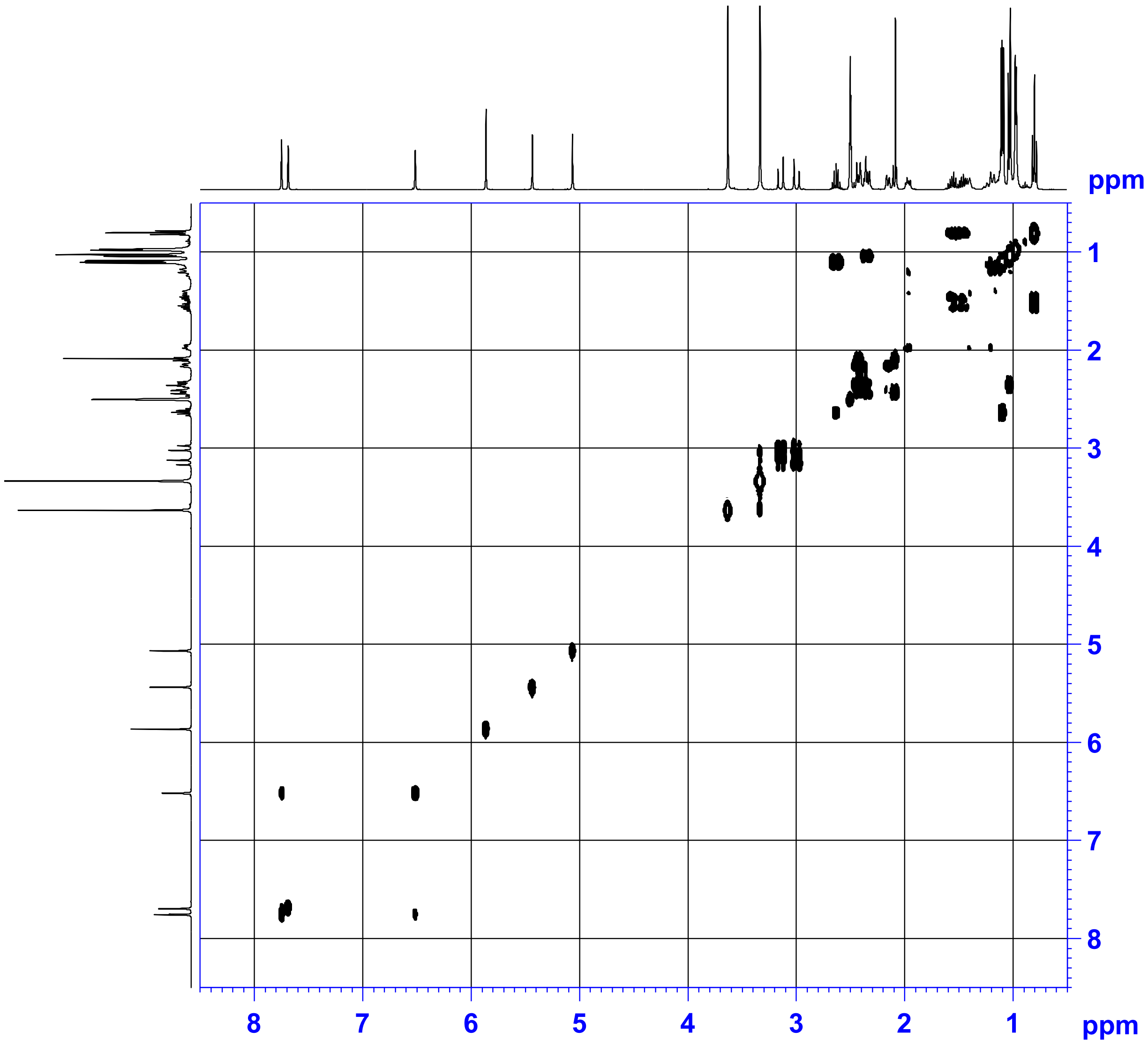

NAME	ZQ-17-DMSO
EXPNO	4
PROCNO	1
Date_	20170706
Time	0.31
INSTRUM	spect
PROBHD	5 mm CPPBBO BB
PULPROG	cosygpppqf
TD	2048
SOLVENT	DMSO
NS	8
DS	8
SWH	3906.250 Hz
FIDRES	1.907349 Hz
AQ	0.2621940 sec
RG	171.57
DW	128.000 usec
DE	10.00 usec
TE	297.0 K
D0	0.00000300 sec
D1	1.89678097 sec
D11	0.03000000 sec
D12	0.00002000 sec
D13	0.00000400 sec
D16	0.00020000 sec
IN0	0.00025600 sec
======== CHANNEL f1 ========	
SFO1	400.1318006 MHz
NUC1	1H
P0	11.50 usec
P1	11.50 usec
P17	2500.00 usec
ND0	1
TD	128
SFO1	400.1318 MHz
FIDRES	30.517578 Hz
SW	9.762 ppm
FnMODE	QF
SI	1024
SF	400.1300018 MHz
WDW	QSINE
SSB	0
LB	0.00 Hz
GB	0
PC	1.40
SI	1024
MC2	QF
SF	400.1300002 MHz
WDW	QSINE
SSB	0
LB	0.00 Hz
GB	0

^{1}H-^{1}H COSY spectrum of Krishnolide A (**1**) in DMSO-d_6

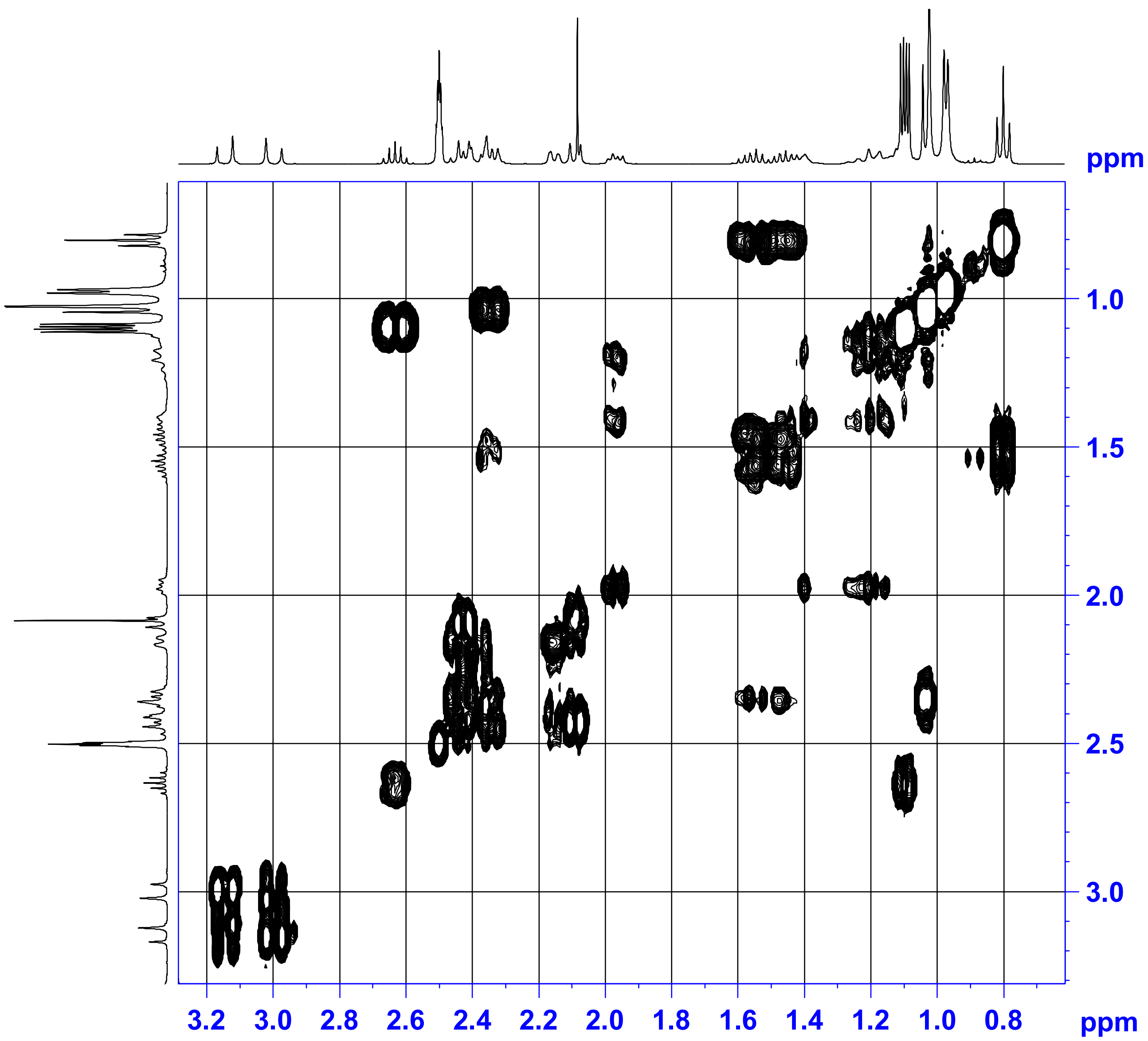

^{1}H-^{1}H COSY spectrum of Krishnolide A (**1**) in DMSO-d_6

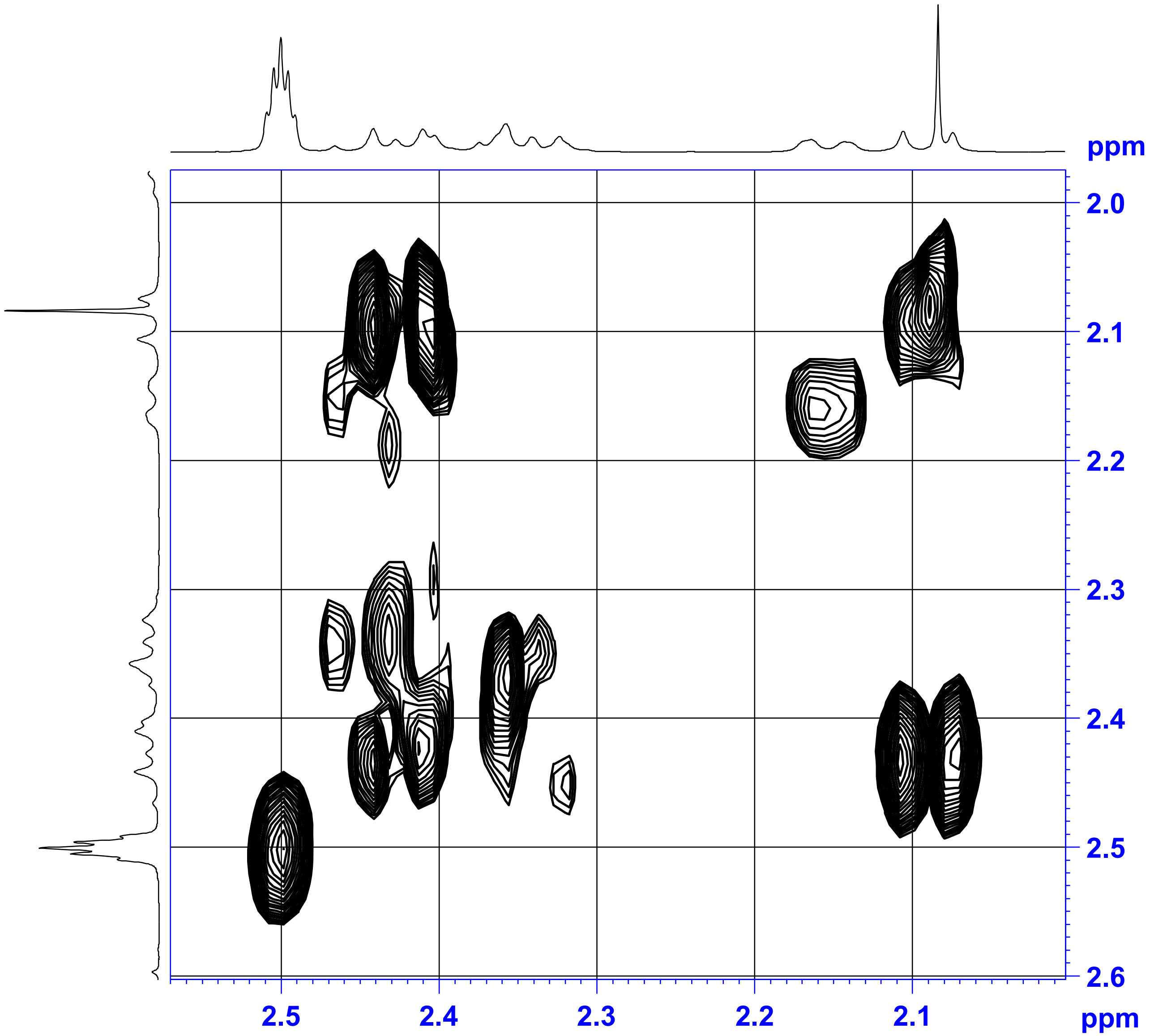

^{1}H-^{1}H COSY spectrum of Krishnolide A (**1**) in DMSO-d_6

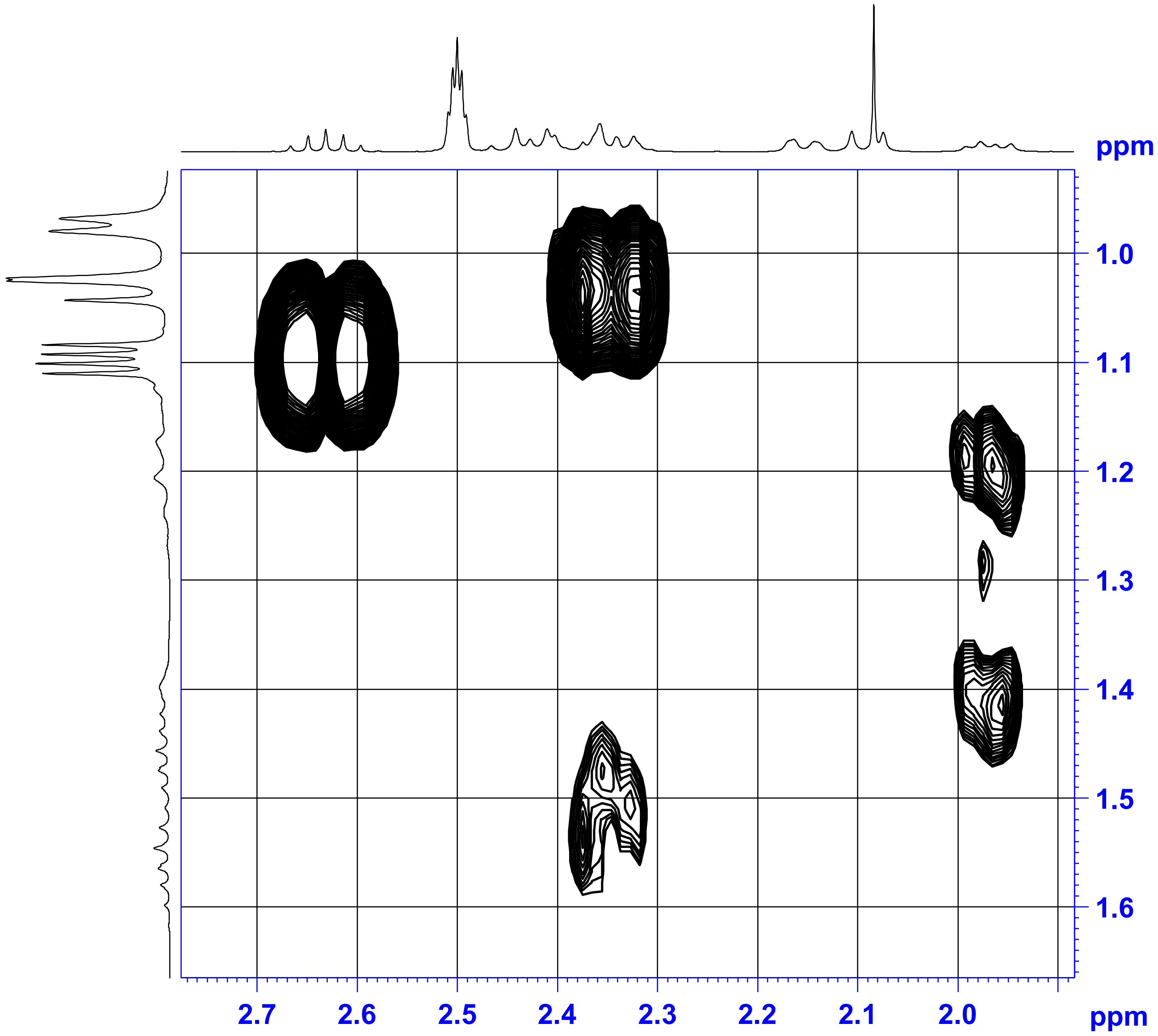

^{1}H-^{1}H COSY spectrum of Krishnolide A (**1**) in DMSO-d_6

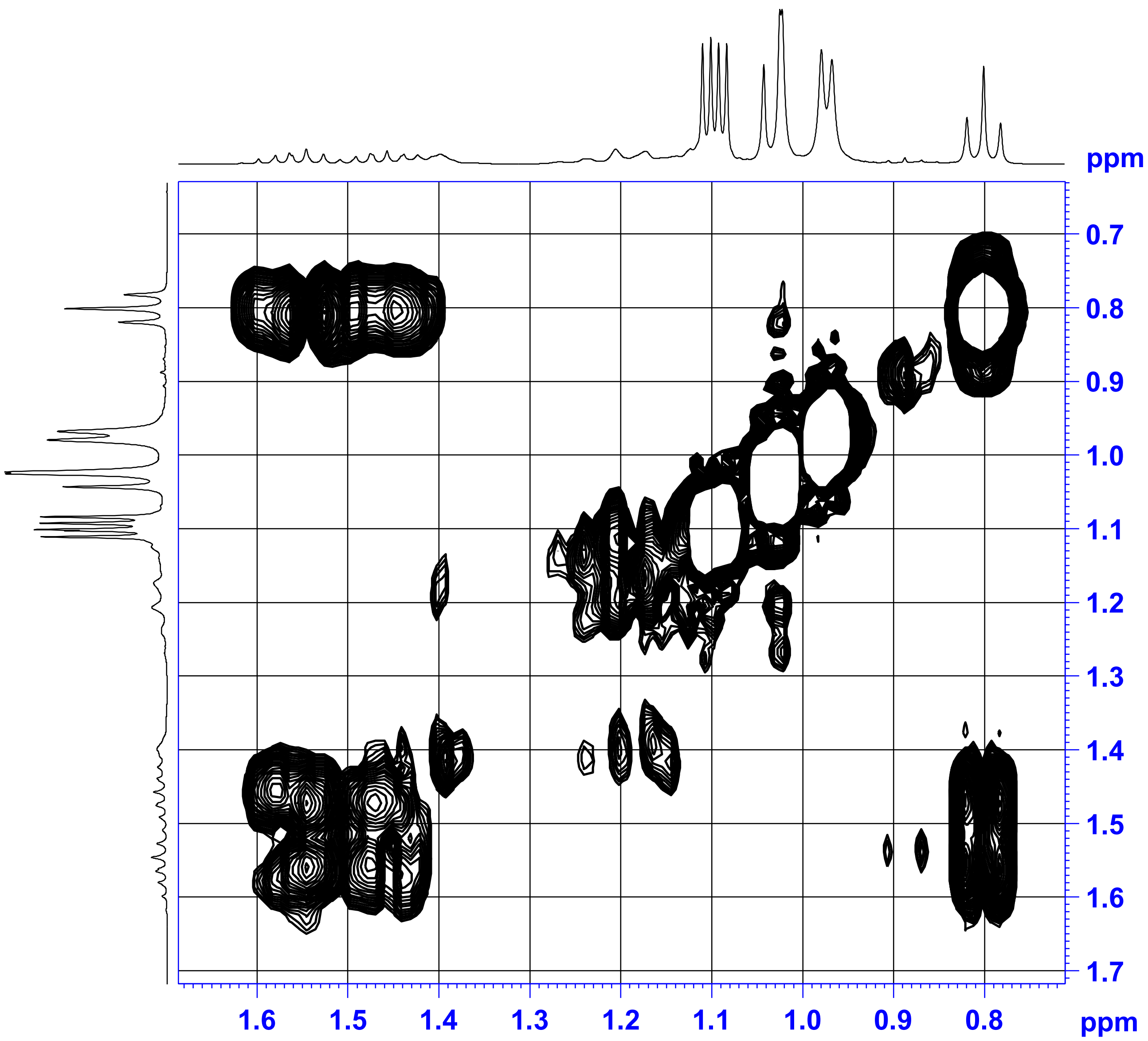

HSQC spectrum of Krishnolide A (**1**) in DMSO-d_6

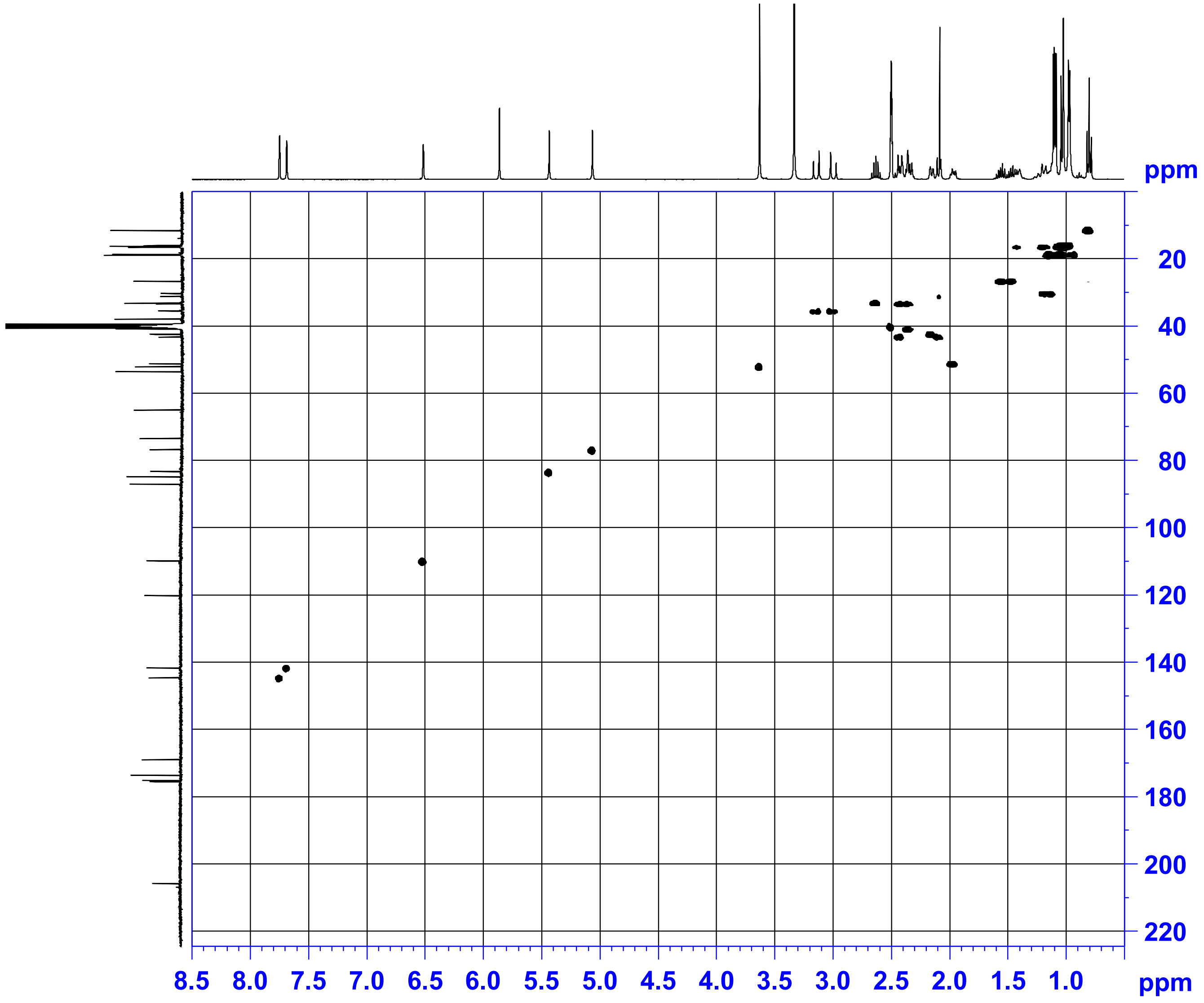

NAME	ZQ-17-DMSO
EXPNO	5
PROCNO	1
Date_	20170706
Time	1.11
INSTRUM	spect
PROBHD	5 mm CPPBBO BB
PULPROG	hsqcetgpsi2
TD	1024
SOLVENT	DMSO
NS	16
DS	16
SWH	4302.926 Hz
FIDRES	4.202076 Hz
AQ	0.1190388 sec
RG	208.5
DW	116.200 usec
DE	10.00 usec
TE	297.0 K
CNST2	145.0000000
D0	0.00000300 sec
D1	1.46497905 sec
D4	0.00172414 sec
D11	0.03000000 sec
D16	0.00020000 sec
D24	0.00086207 sec
IN0	0.00002080 sec
ZGOPTNS	

======== CHANNEL f1 ========

SFO1	400.1320007 MHz
NUC1	1H
P1	11.50 usec
P2	23.00 usec
P28	0.00 usec
ND0	2
TD	256
SFO1	100.6233 MHz
FIDRES	93.900238 Hz
SW	238.896 ppm
FnMODE	Echo-Antiecho
SI	1024
SF	400.1300015 MHz
WDW	QSINE
SSB	2
LB	0.00 Hz
GB	0
PC	1.40
SI	1024
MC2	echo-antiecho
SF	100.6127556 MHz
WDW	QSINE
SSB	2
LB	0.00 Hz
GB	0

HSQC spectrum of Krishnolide A (**1**) in DMSO-d_6

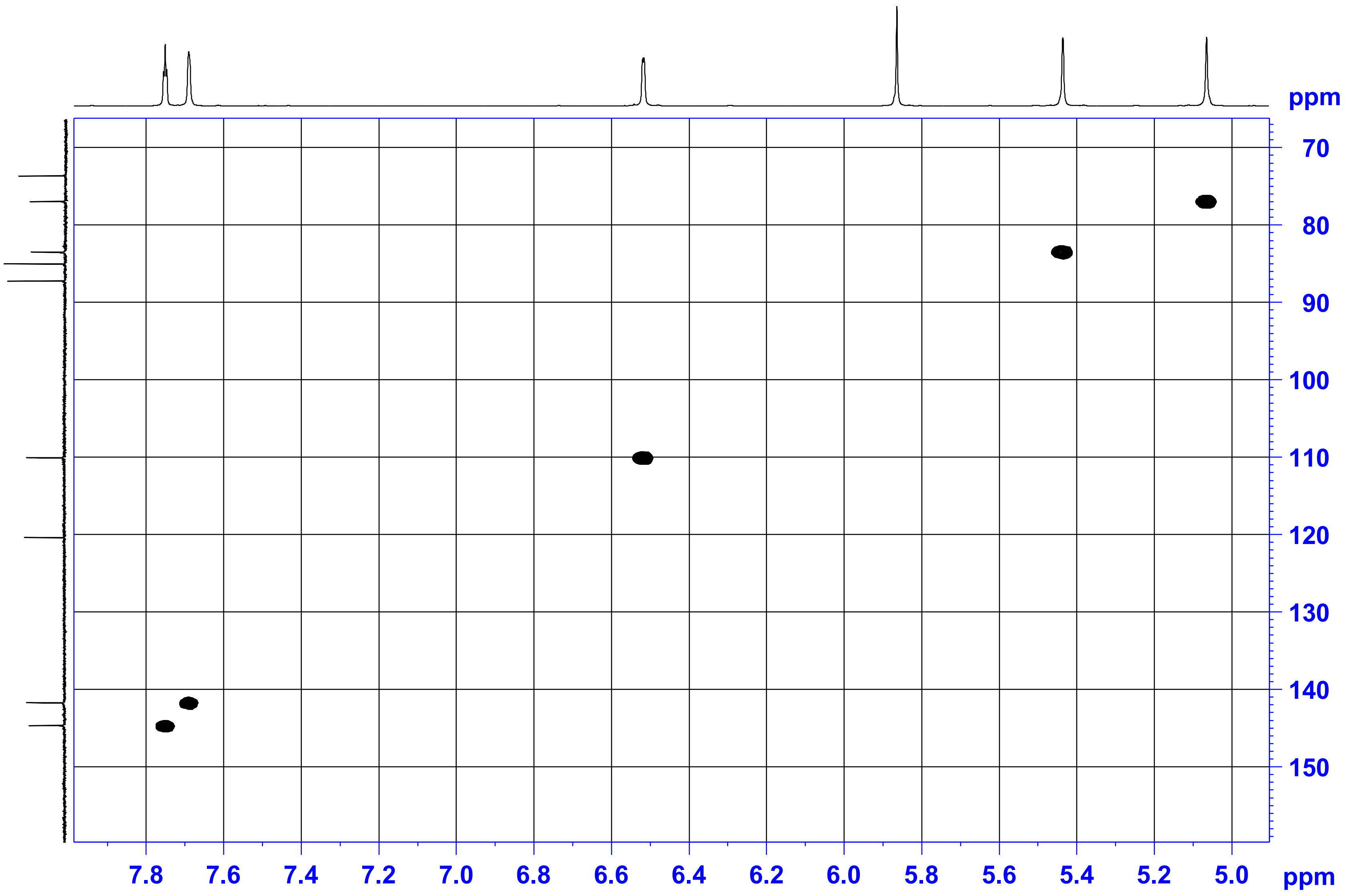

HSQC spectrum of Krishnolide A (**1**) in DMSO-d_6

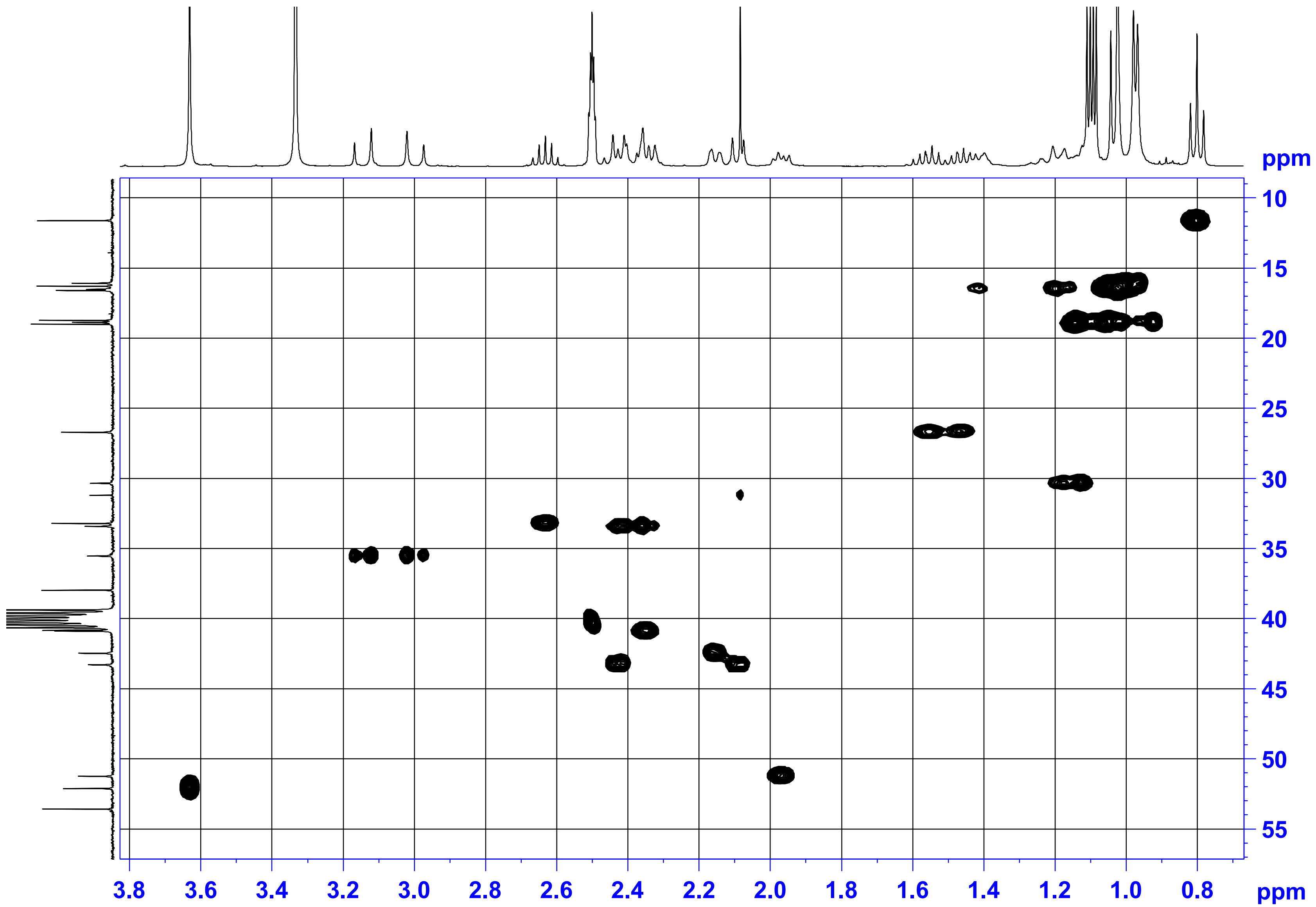

HSQC spectrum of Krishnolide A (**1**) in DMSO-d_6

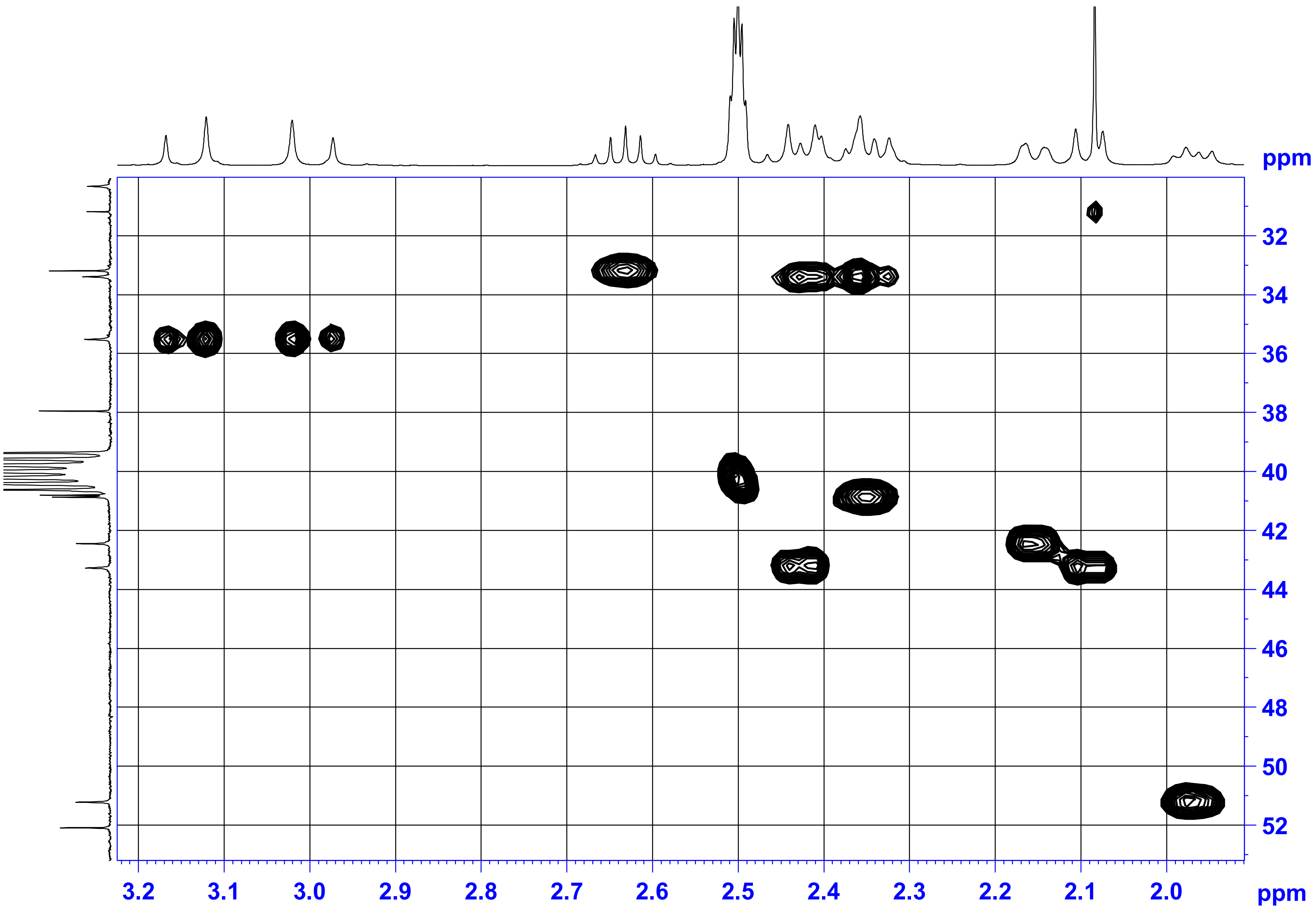

HSQC spectrum of Krishnolide A (**1**) in DMSO-d_6

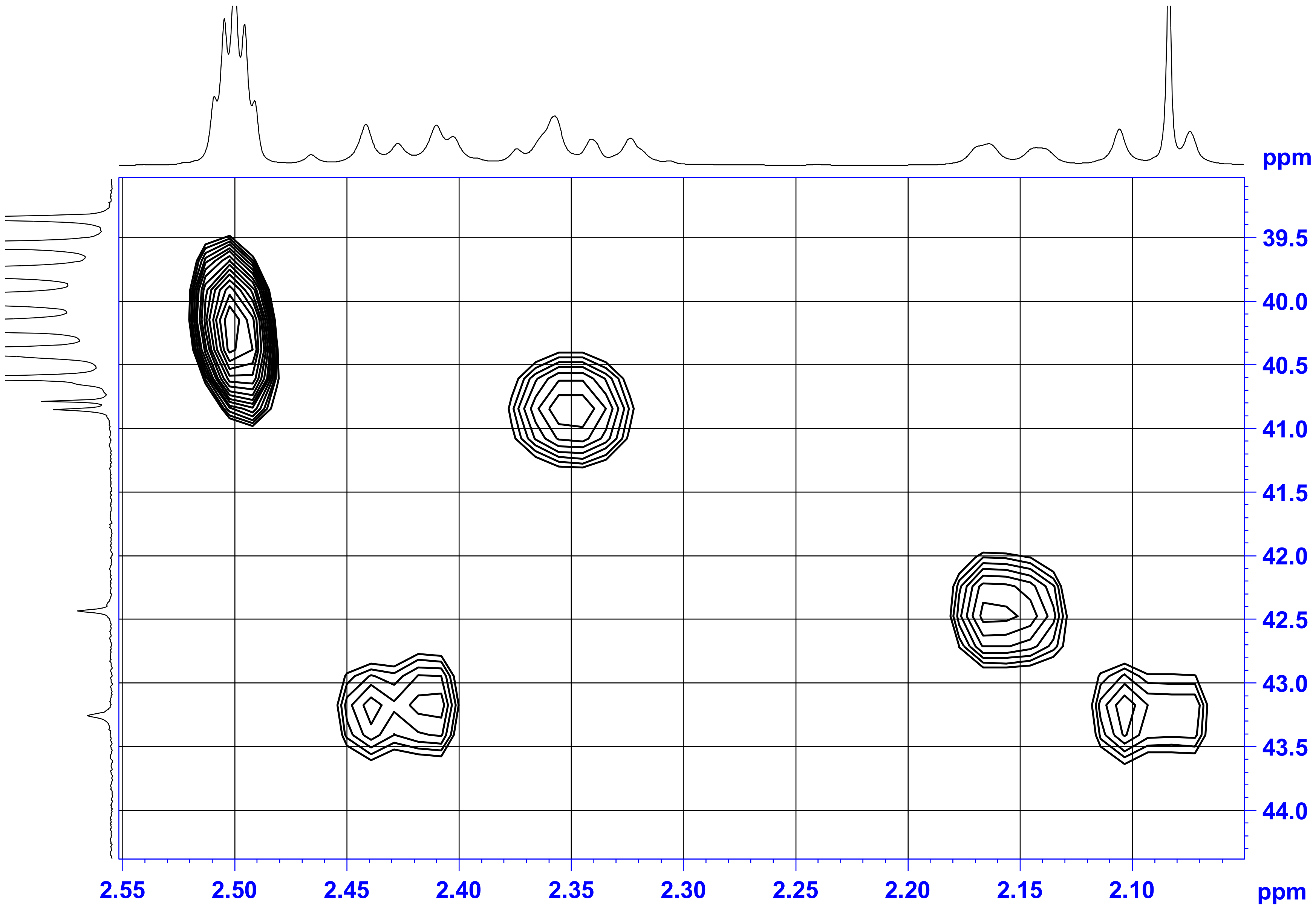

HSQC spectrum of Krishnolide A (**1**) in DMSO-d_6

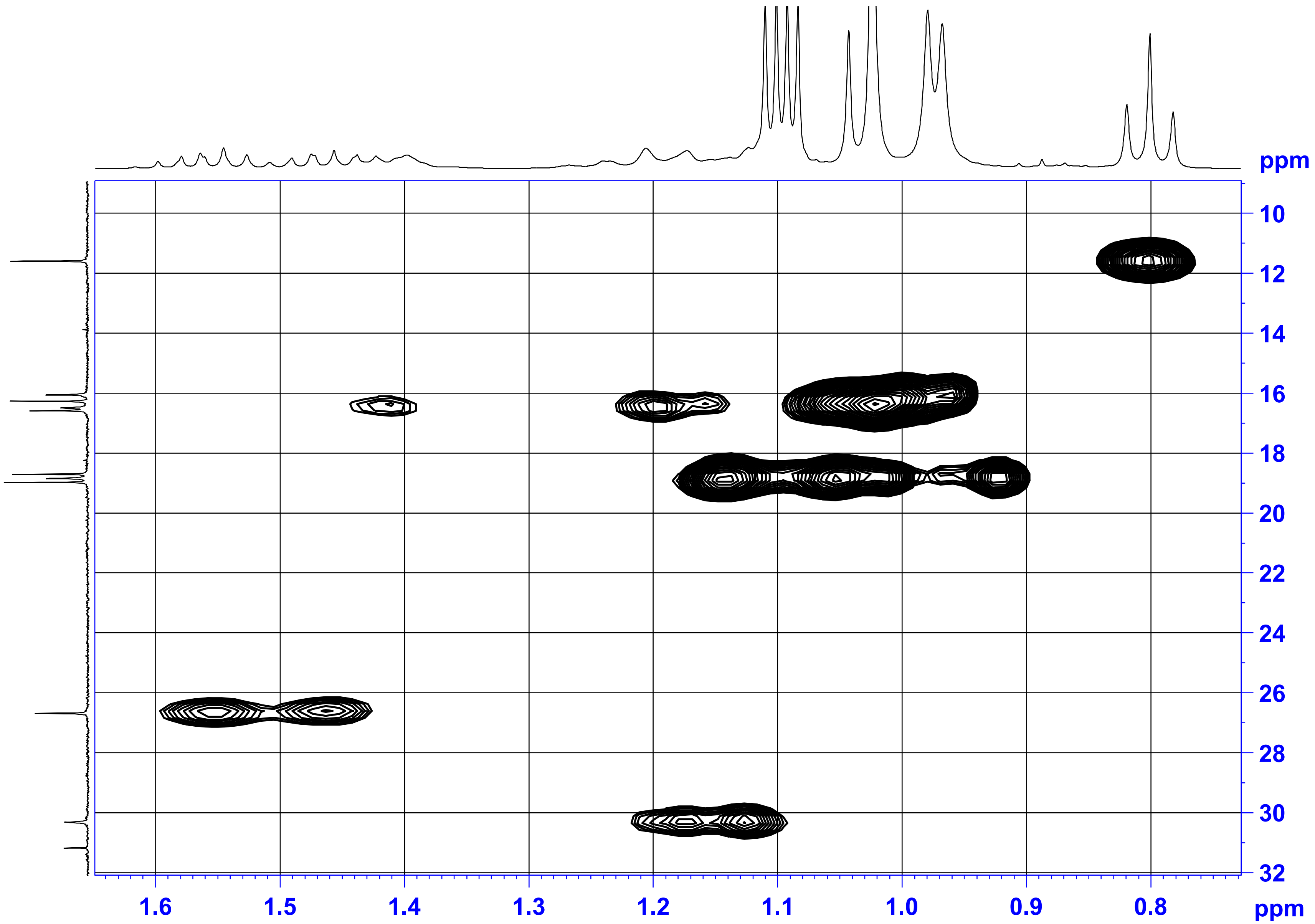

HSQC spectrum of Krishnolide A (**1**) in DMSO-d_6

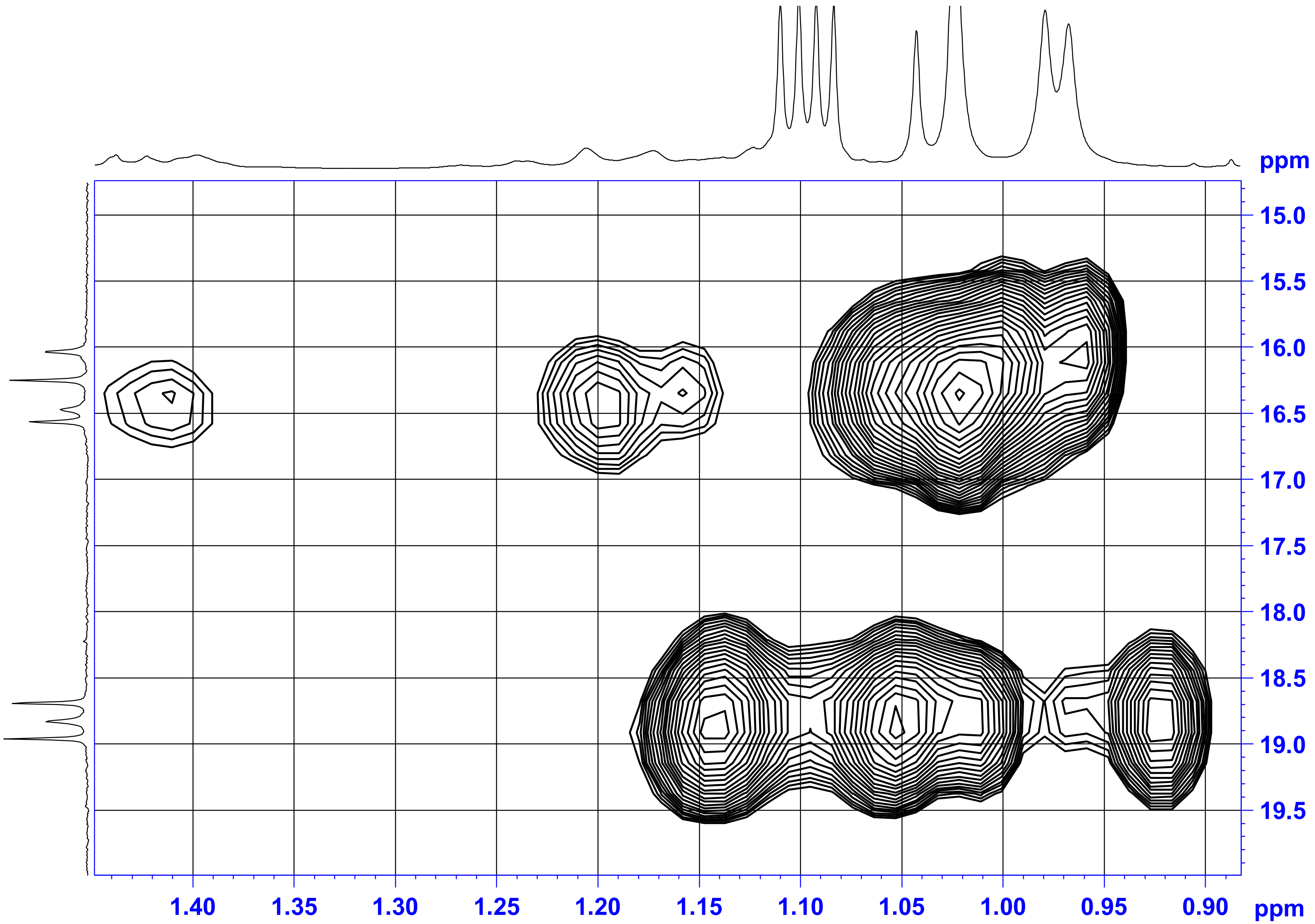

HMBC spectrum of Krishnolide A (**1**) in DMSO-d_6

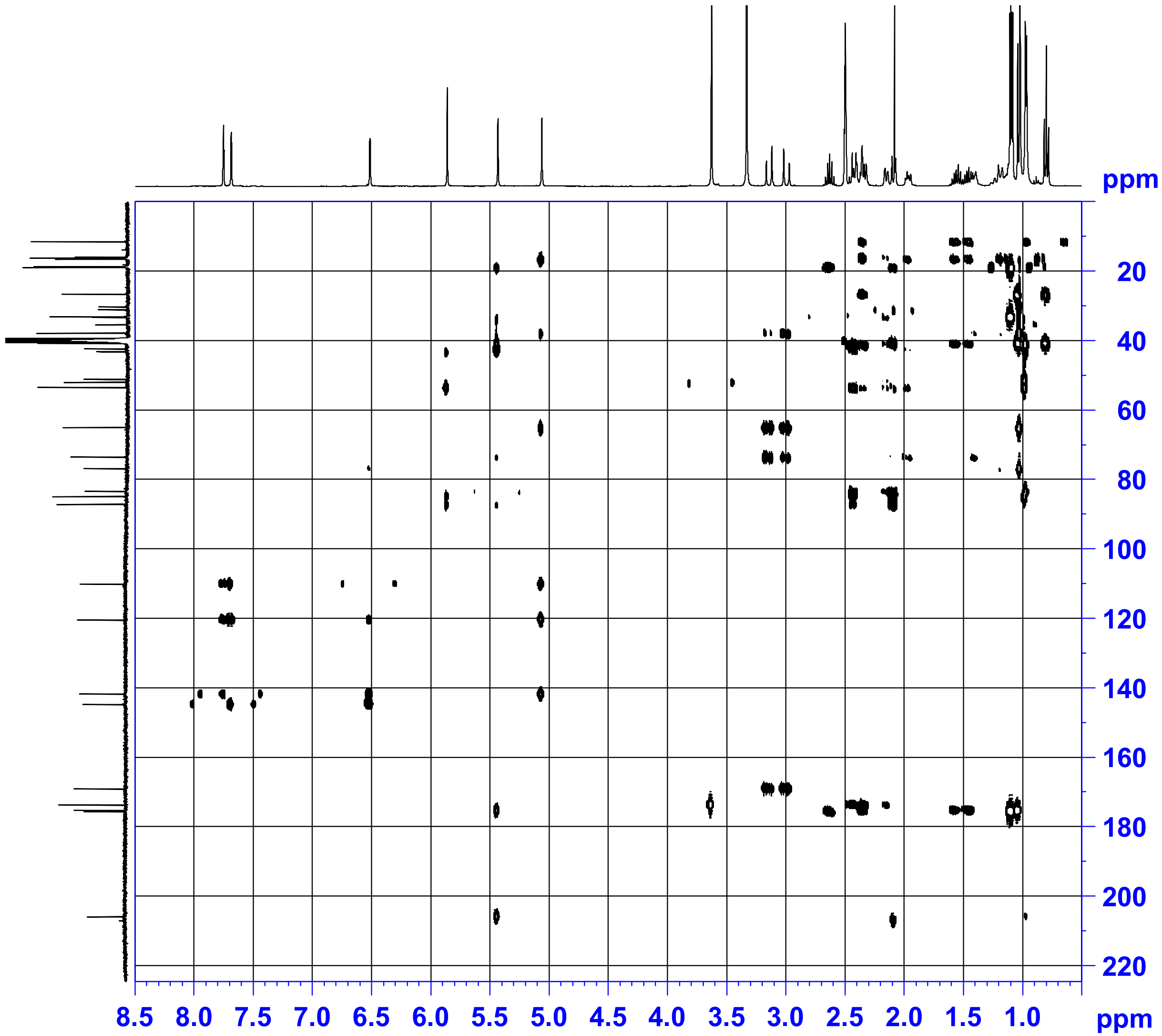

NAME	ZQ-17-DMSO	
EXPNO	6	
PROCNO	1	
Date_	20170706	
Time	3.02	
INSTRUM	spect	
PROBHD	5 mm CPPBBO BB	
PULPROG	hmbcgplpndqf	
TD	4096	
SOLVENT	DMSO	
NS	32	
DS	16	
SWH	5197.505	Hz
FIDRES	1.268922	Hz
AQ	0.3940852	sec
RG	208.5	
DW	96.200	usec
DE	10.00	usec
TE	297.0	K
CNST2	145.0000000	
CNST13	10.0000000	
D0	0.00000300	sec
D1	1.50000000	sec
D2	0.00344828	sec
D6	0.05000000	sec
D16	0.00020000	sec
IN0	0.00002080	sec
======== CHANNEL f1 ========		
SFO1	400.1323208	MHz
NUC1	1H	
P1	11.50	usec
P2	23.00	usec
ND0	2	
TD	128	
SFO1	100.6233	MHz
FIDRES	187.800476	Hz
SW	238.896	ppm
FnMODE	QF	
SI	2048	
SF	400.1300015	MHz
WDW	SINE	
SSB	0	
LB	0.00	Hz
GB	0	
PC	1.40	
SI	1024	
MC2	QF	
SF	100.6127527	MHz
WDW	SINE	
SSB	0	
LB	0.00	Hz
GB	0	

HMBC spectrum of Krishnolide A (**1**) in DMSO-d_6

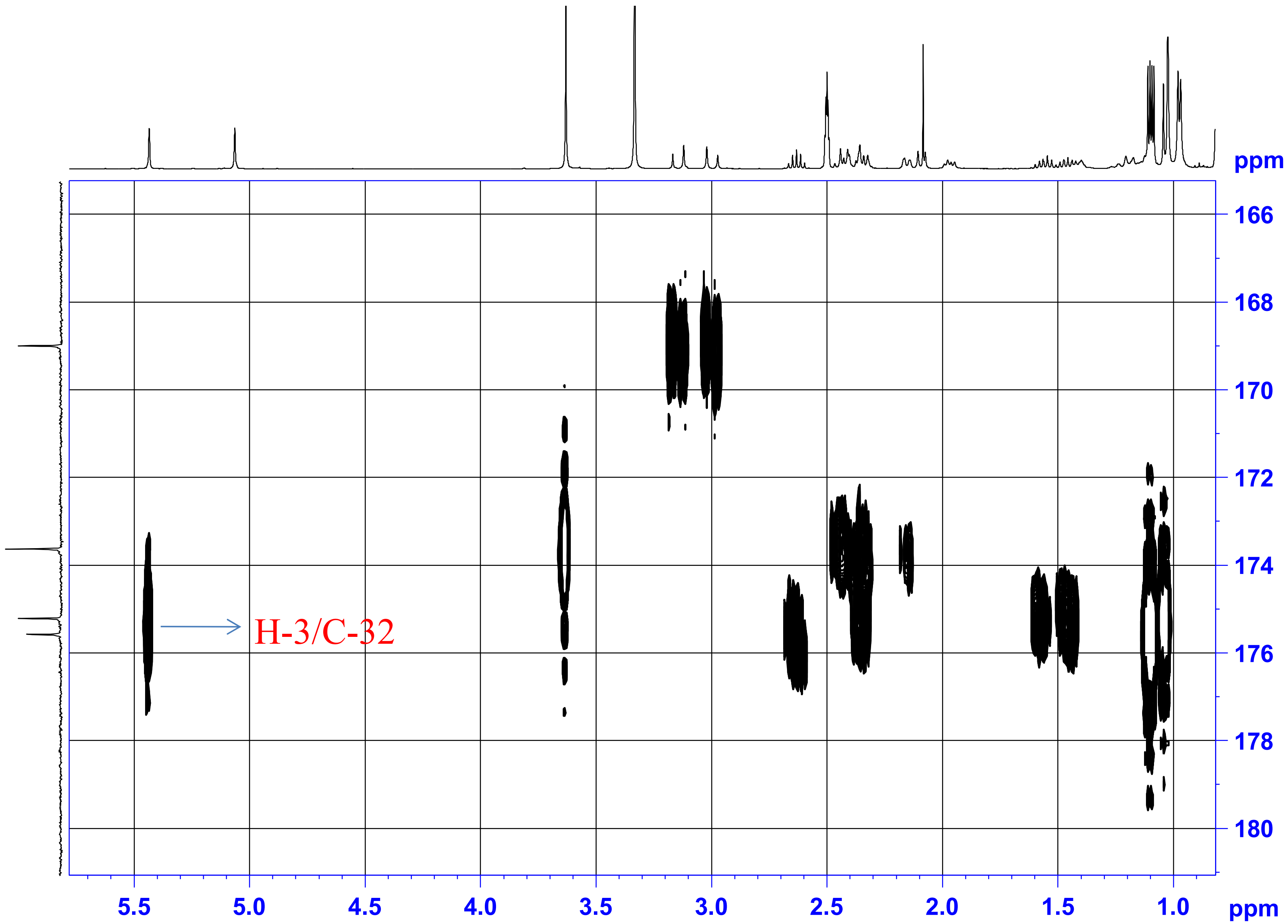

HMBC spectrum of Krishnolide A (**1**) in DMSO-d_6

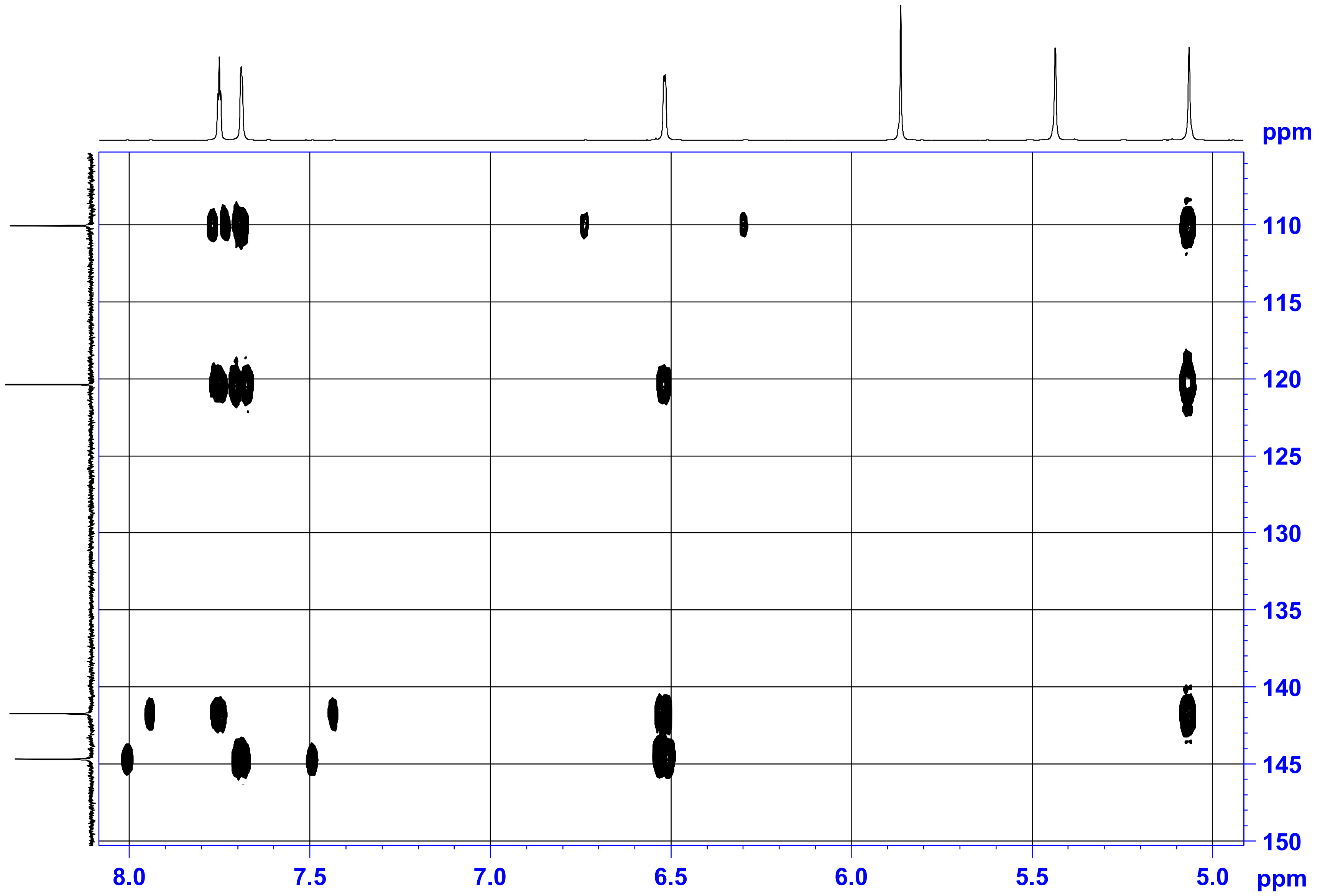

HMBC spectrum of Krishnolide A (**1**) in DMSO-d_6

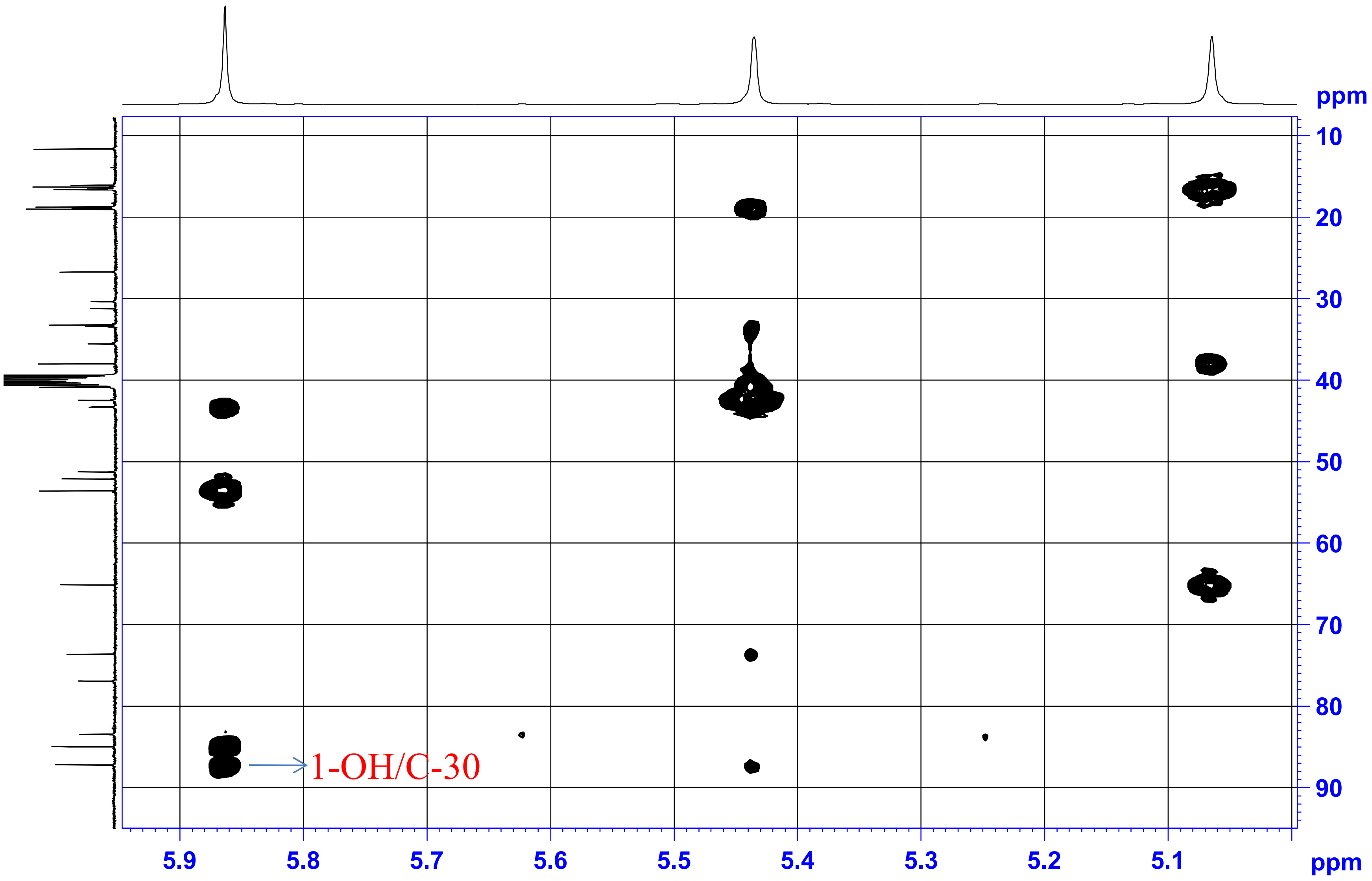

HMBC spectrum of Krishnolide A (**1**) in DMSO-d_6

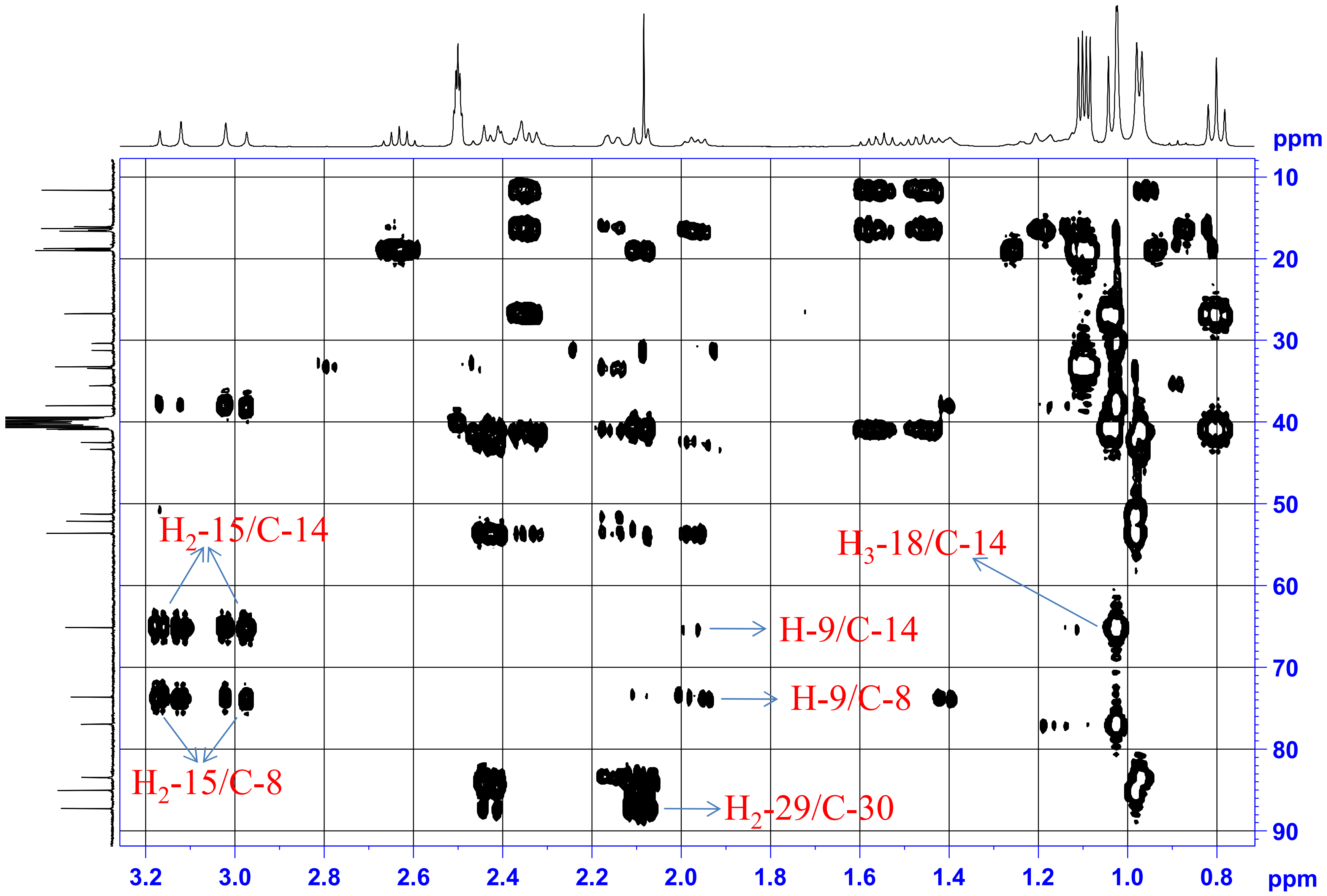

HMBC spectrum of Krishnolide A (**1**) in DMSO-d_6

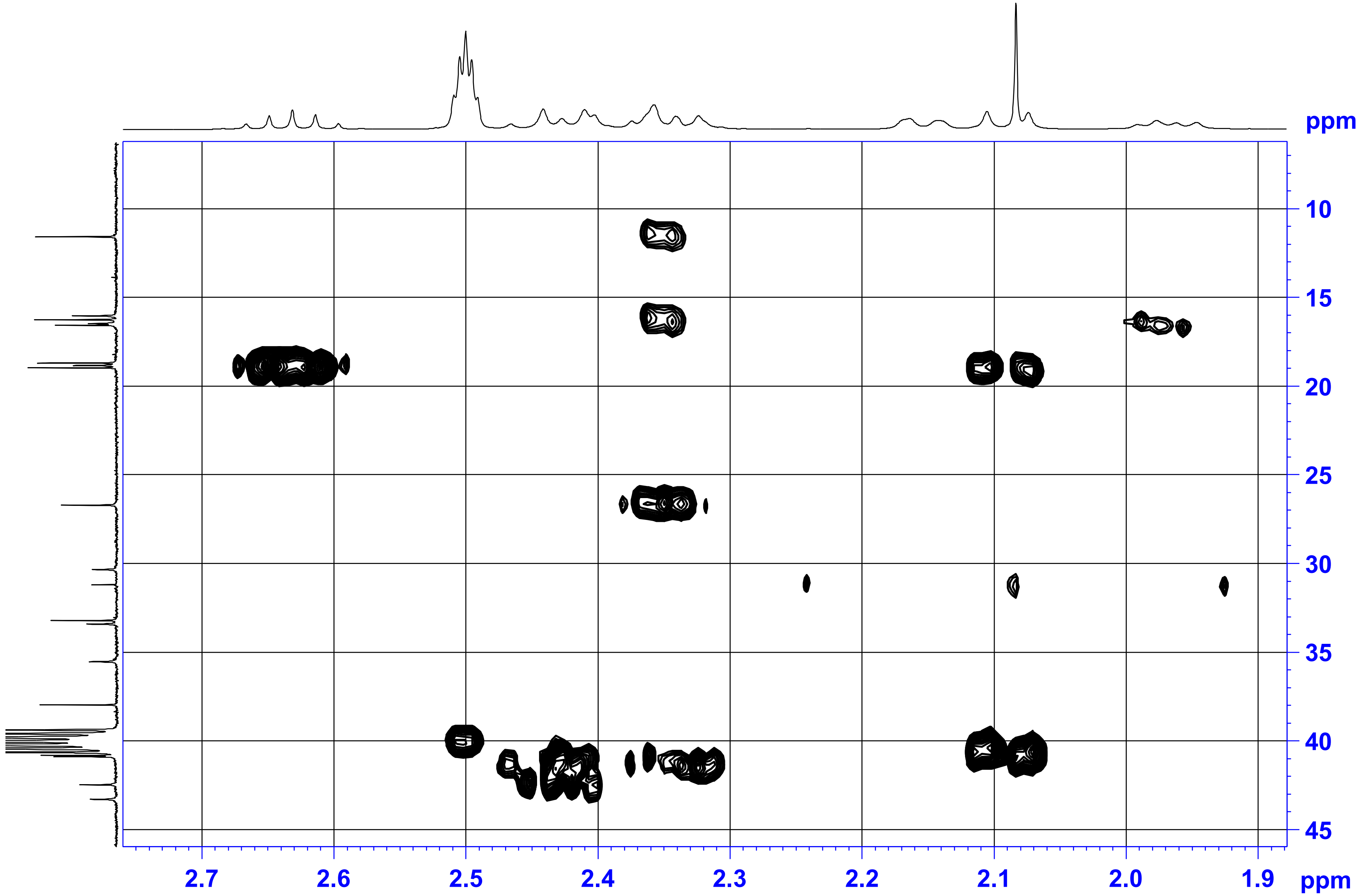

HMBC spectrum of Krishnolide A (**1**) in DMSO-d_6

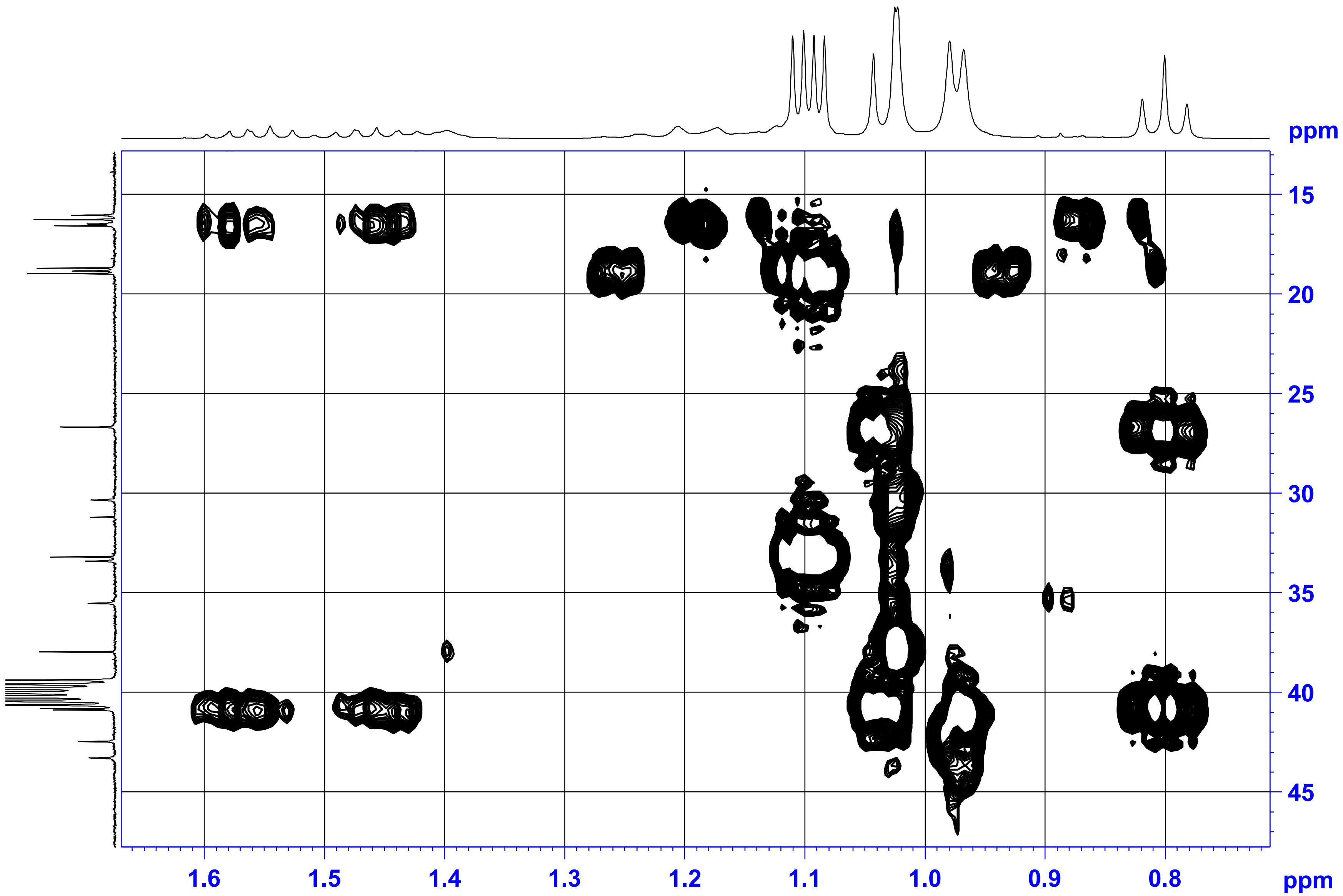

NOESY spectrum of Krishnolide A (**1**) in DMSO-d_6

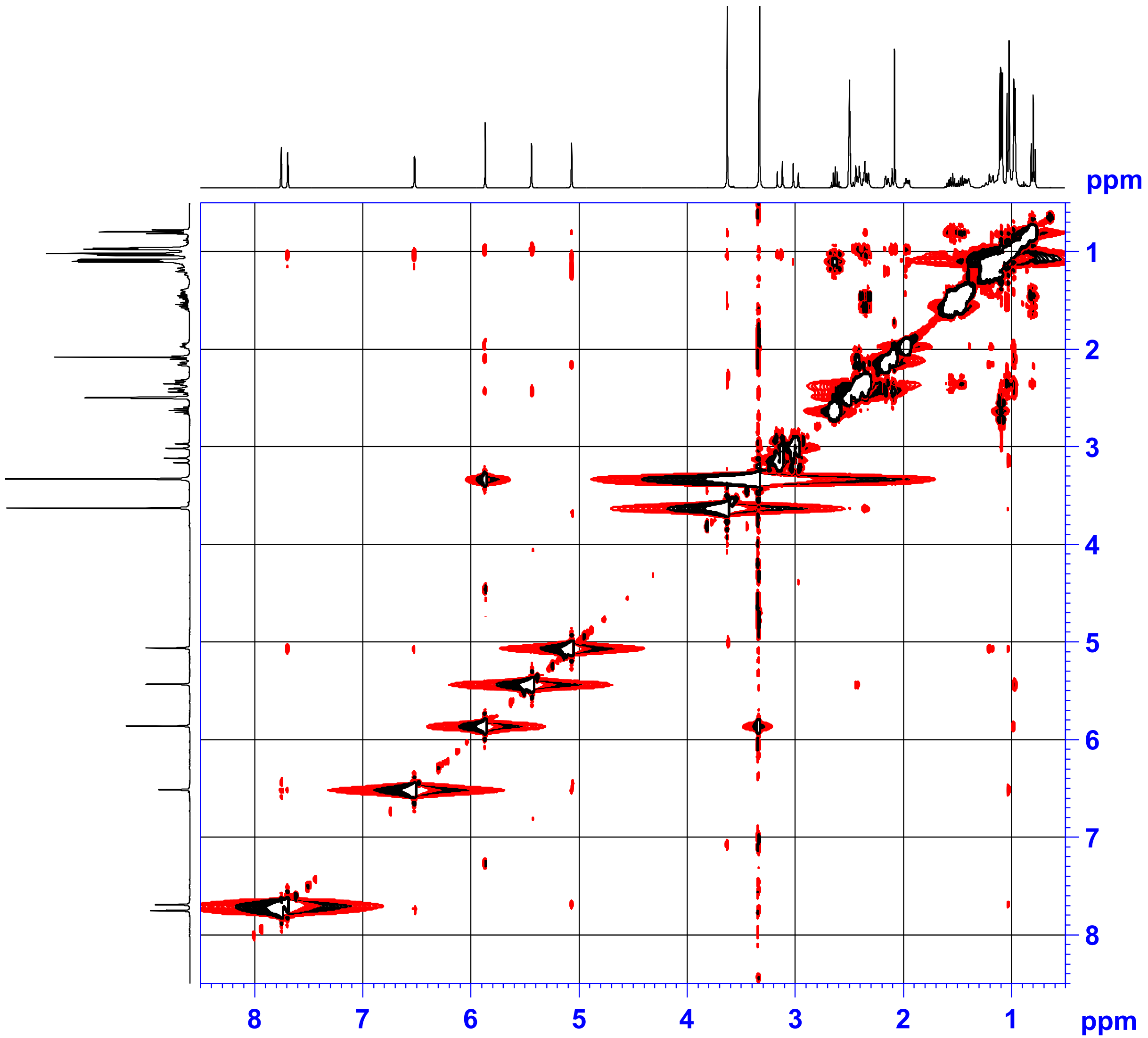

NAME	ZQ-17-DMSO
EXPNO	7
PROCNO	1
Date_	20170706
Time	5.18
INSTRUM	spect
PROBHD	5 mm CPPBBO BB
PULPROG	noesygpphpp
TD	2048
SOLVENT	DMSO
NS	16
DS	32
SWH	4000.000 Hz
FIDRES	1.953125 Hz
AQ	0.2560500 sec
RG	117.37
DW	125.000 usec
DE	10.00 usec
TE	297.0 K
D0	0.00011036 sec
D1	1.99385595 sec
D8	0.30000001 sec
D11	0.03000000 sec
D12	0.00002000 sec
D16	0.00020000 sec
IN0	0.00025000 sec
======== CHANNEL f1 ========	
SFO1	400.1318006 MHz
NUC1	1H
P1	11.50 usec
P2	23.00 usec
P17	2500.00 usec
ND0	1
TD	256
SFO1	400.1318 MHz
FIDRES	15.625000 Hz
SW	9.997 ppm
FnMODE	States-TPPI
SI	1024
SF	400.1300018 MHz
WDW	QSINE
SSB	2
LB	0.00 Hz
GB	0
PC	1.00
SI	1024
MC2	States-TPPI
SF	400.1300002 MHz
WDW	QSINE
SSB	2
LB	0.00 Hz
GB	0

NOESY spectrum of Krishnolide A (**1**) in DMSO-d_6

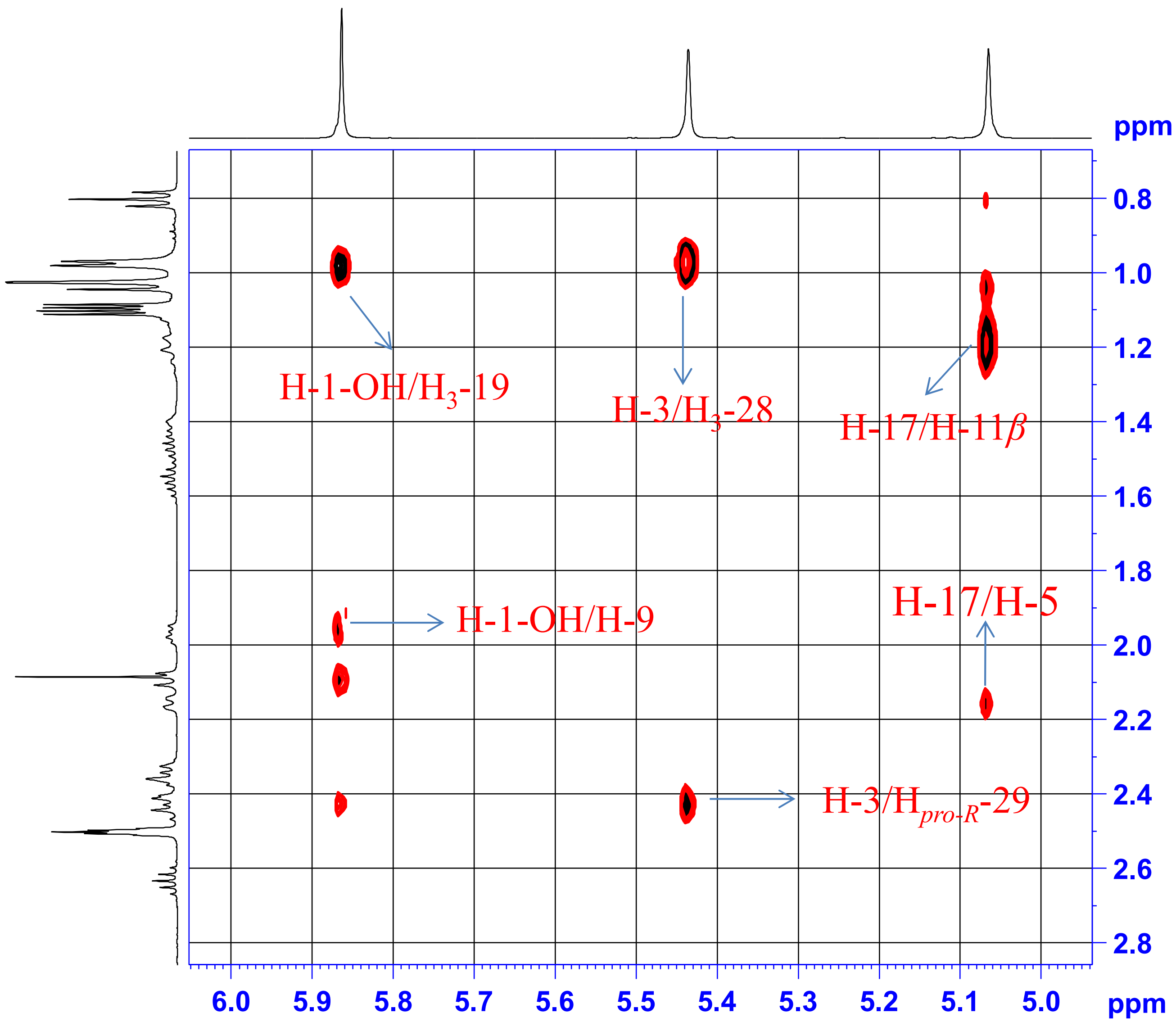

NOESY spectrum of Krishnolide A (**1**) in DMSO-d_6

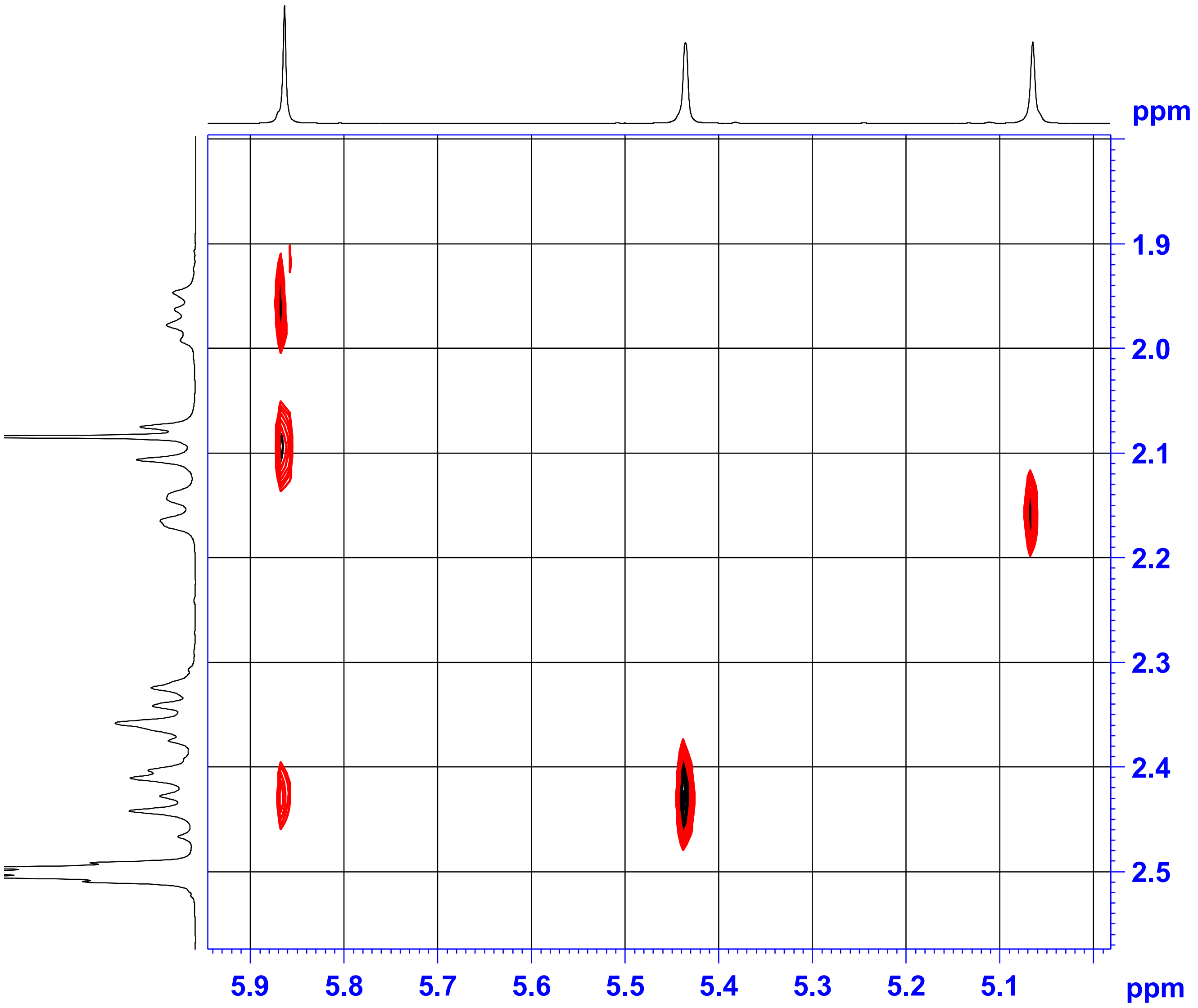

NOESY spectrum of Krishnolide A (**1**) in DMSO-d_6

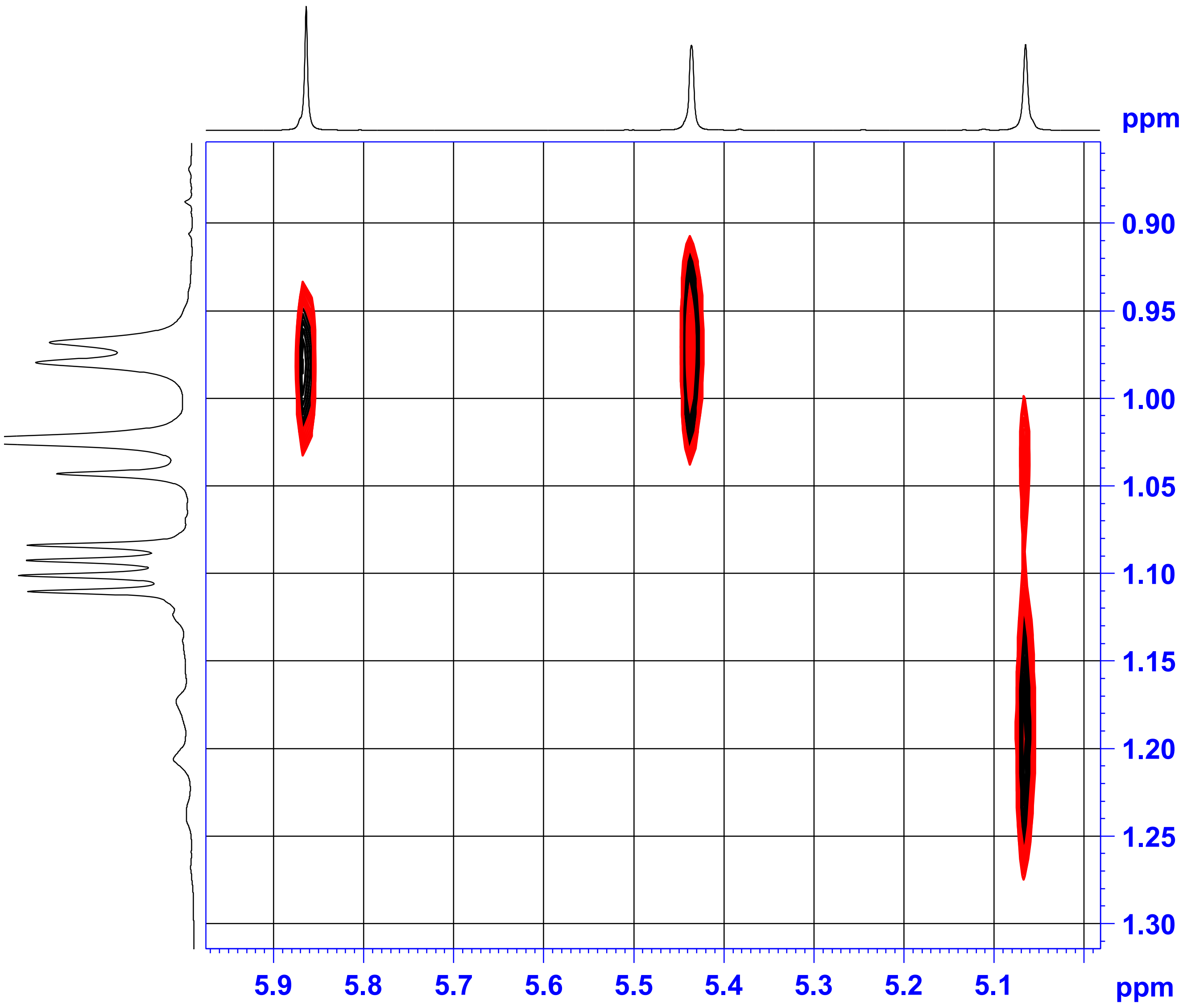

NOESY spectrum of Krishnolide A (**1**) in DMSO-d_6

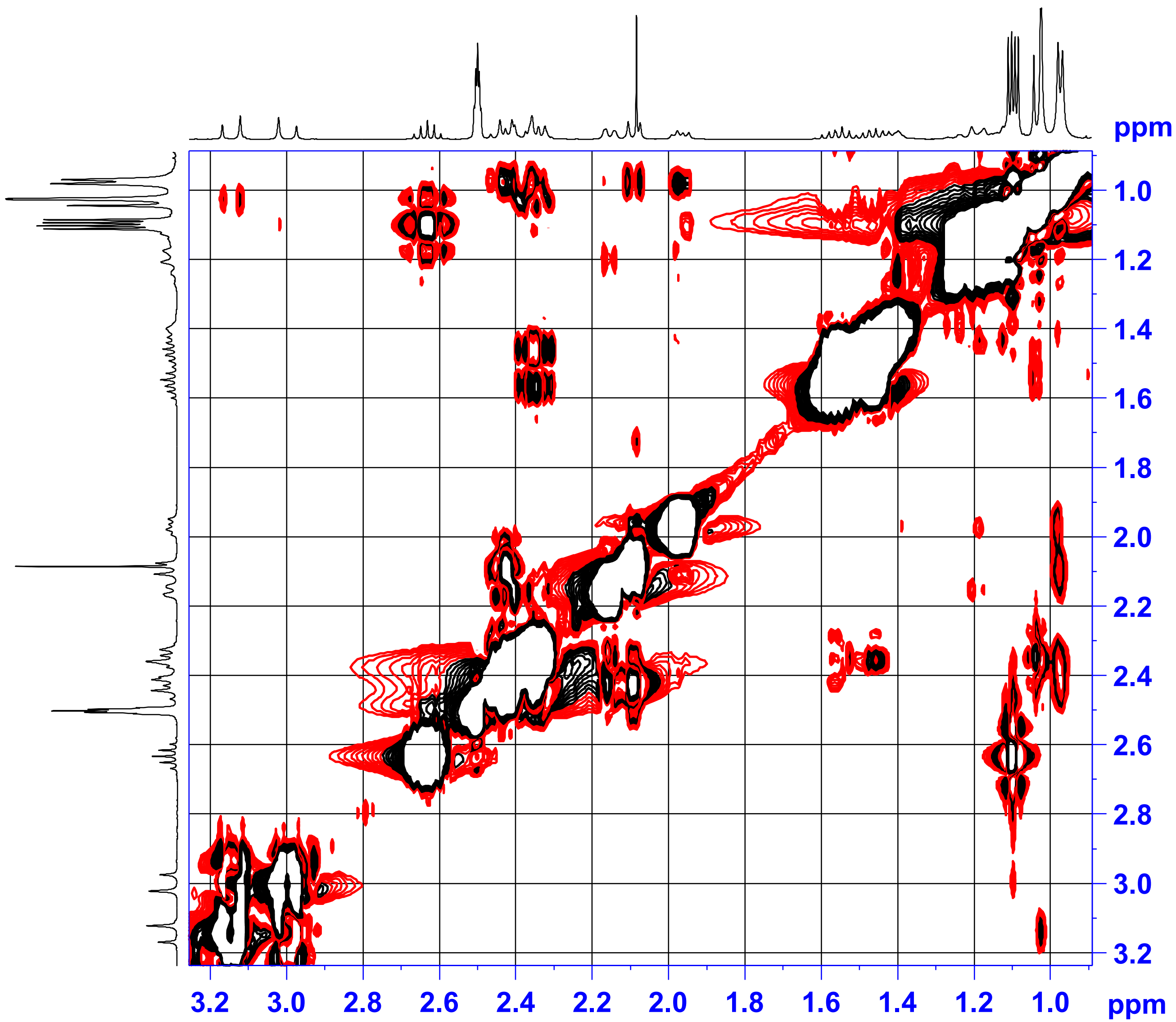

NOESY spectrum of Krishnolide A (**1**) in DMSO-d_6

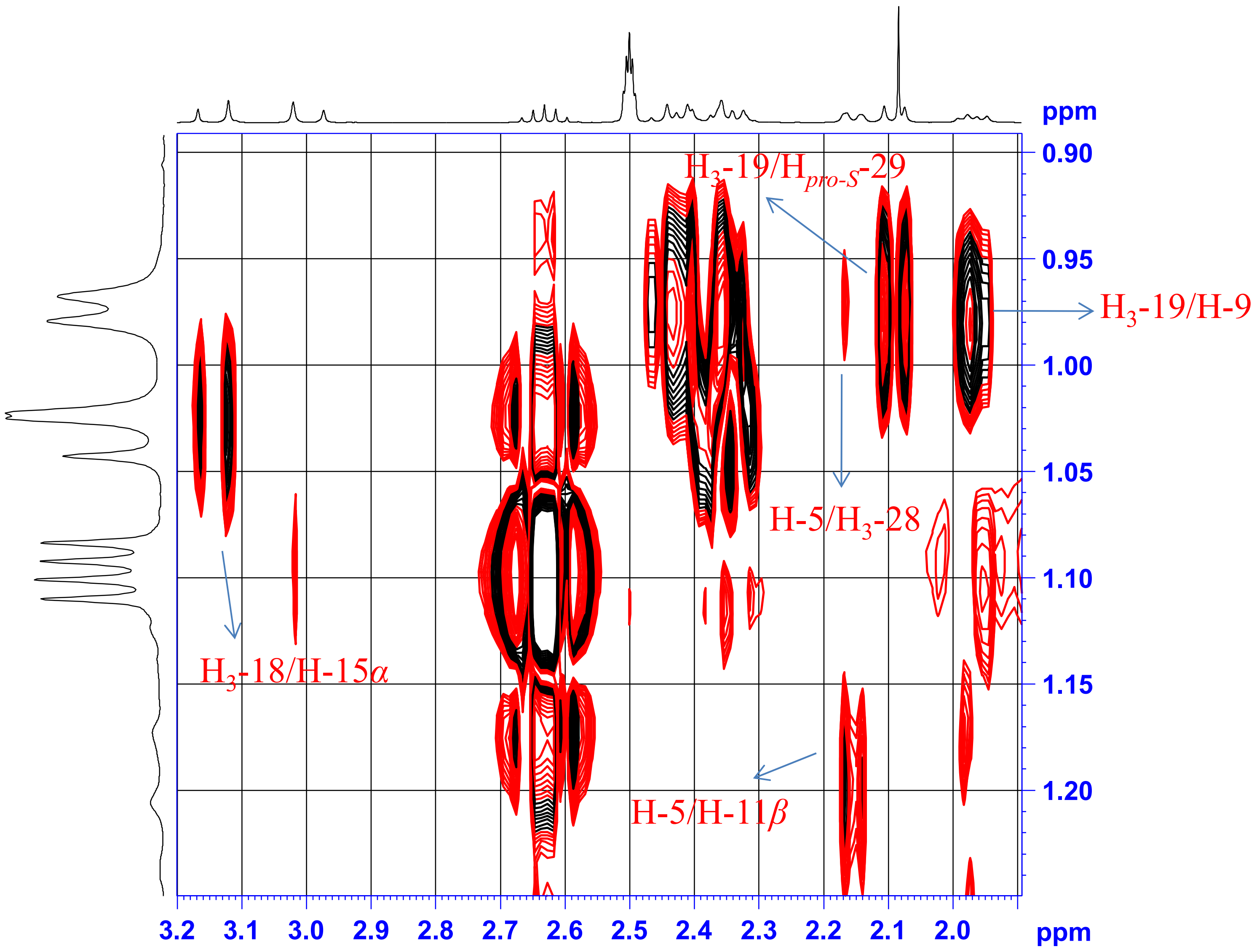

HR-ESIMS of Krishnolide B (**2**)

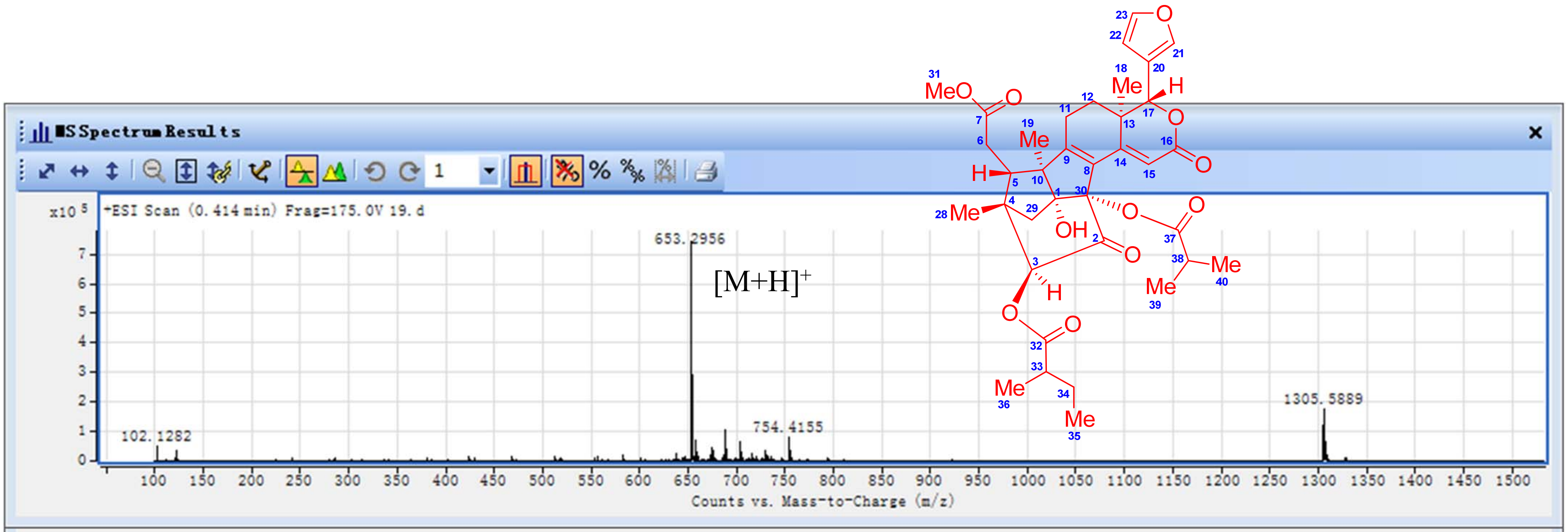

MS Formula Results: + Scan (0.414 min)

m/z	Ion	Formula	Abundance
653.2956	(M+H)+	C36 H45 O11	743096.6

Best	Formula (M)	Ion Formula	Score	Cross Score	Calc m/z	Diff (ppm)	Mass Match	Abund Match	Spacing Match
☑	C36 H44 O11	C36 H45 O11	97.6		653.2956	0.14	99.97	99.31	90.81

Isotope	Abund Sum%	Calc Abund Sum%	m/z	Calc m/z	Diff (ppm)
1	65.42	65.9	653.2956	653.2956	0.08
2	26	26.28	654.2984	654.299	1.04
3	7.02	6.59	655.299	655.3019	4.42
4	1.56	1.24	656.2997	656.3046	7.47

UV spectrum of Krishnolide B (**2**) in MeCN

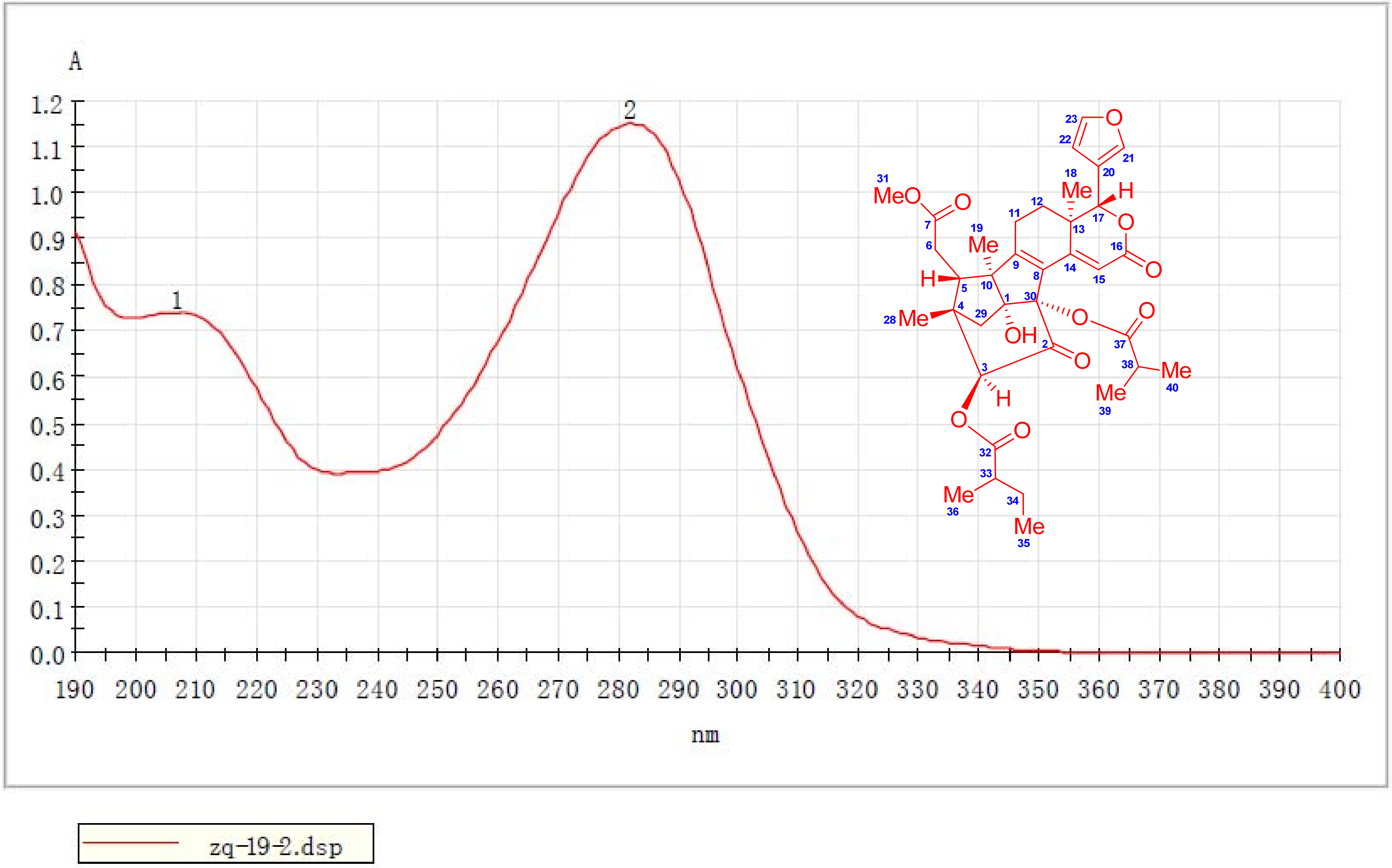

^1H NMR (400 MHz) spectrum of Krishnolide B (**2**) in $CDCl_3$

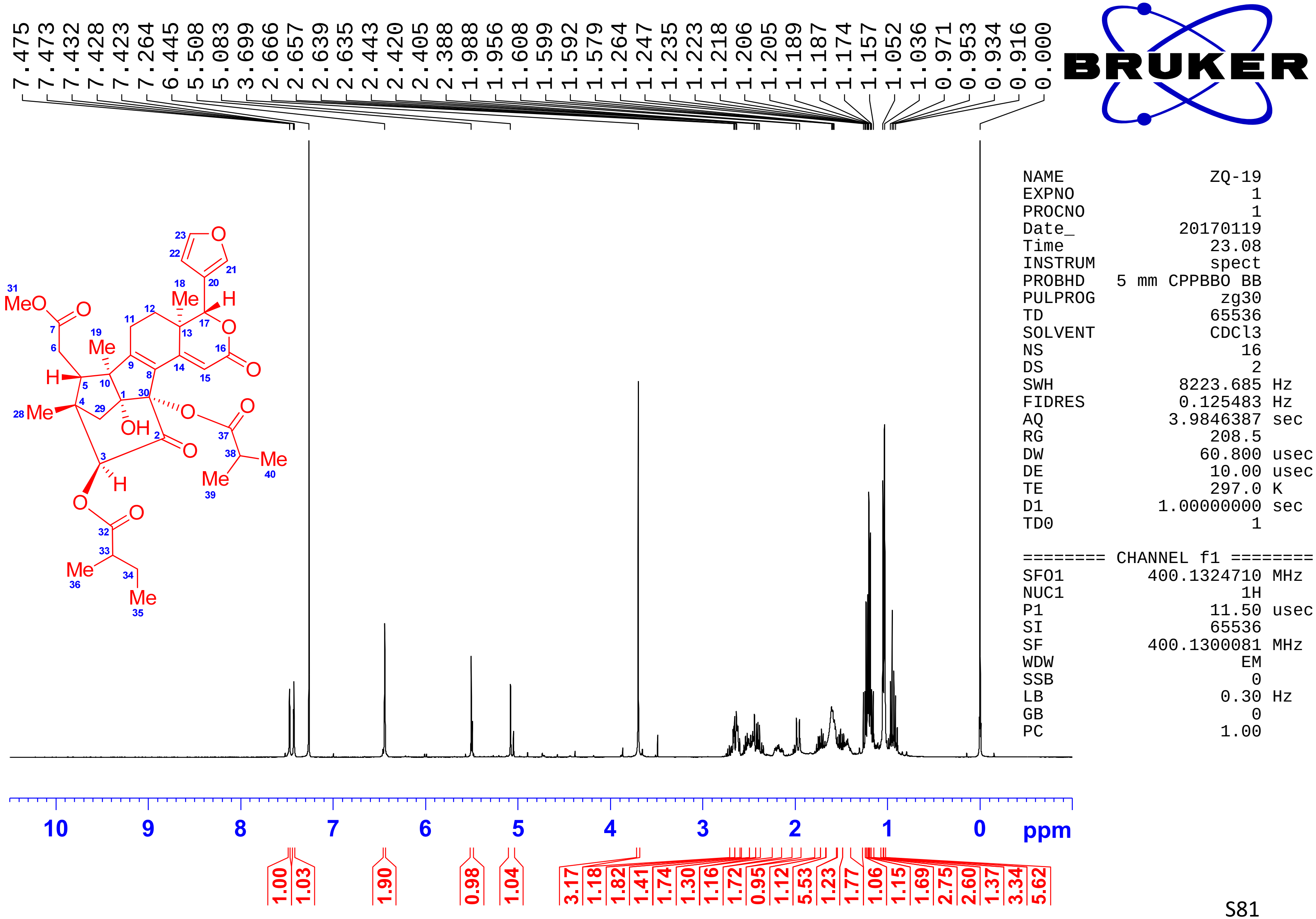

^{1}H NMR (400 MHz) spectrum of Krishnolide B (**2**) in $CDCl_3$

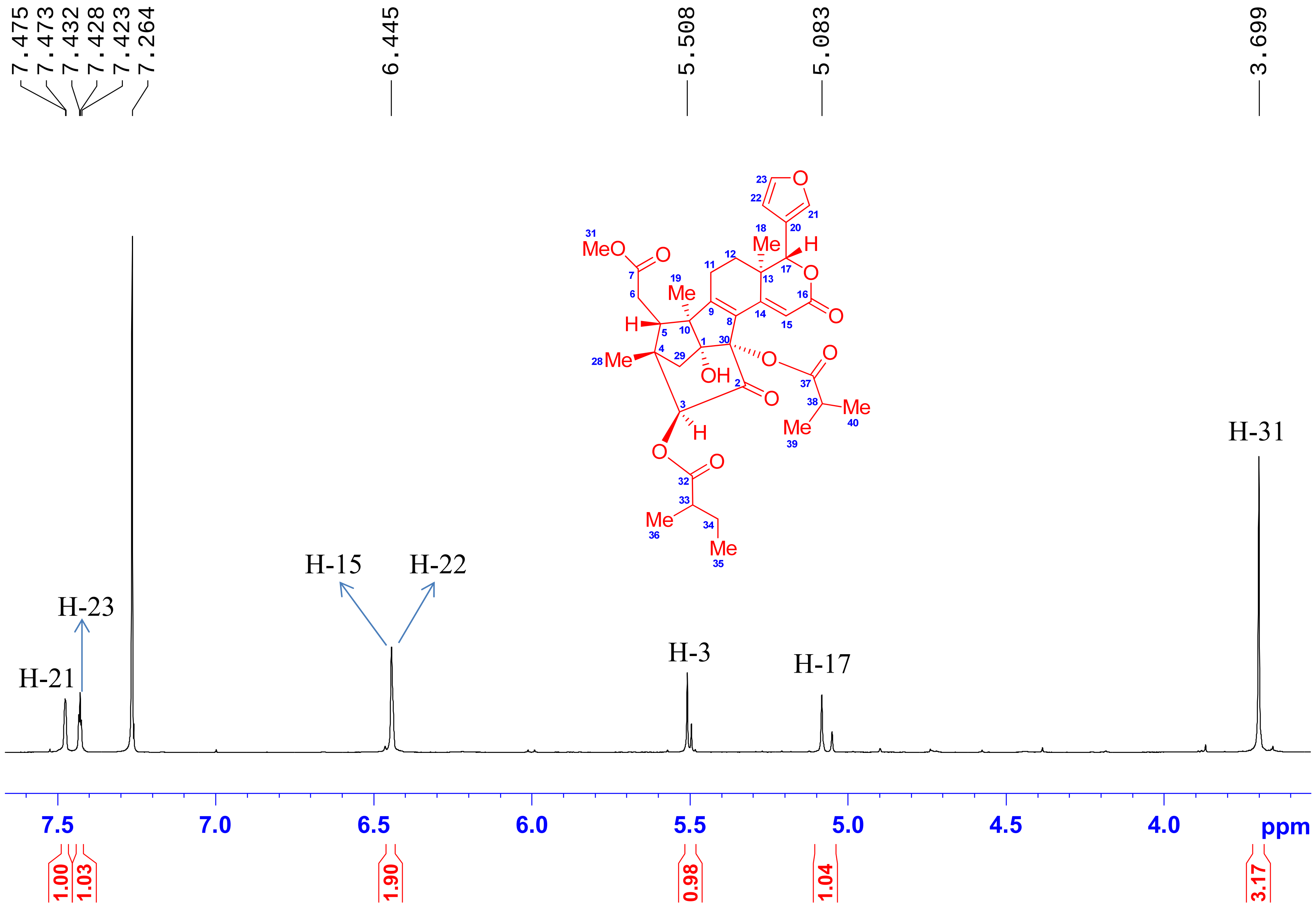

^{1}H NMR (400 MHz) spectrum of Krishnolide B (**2**) in $CDCl_3$

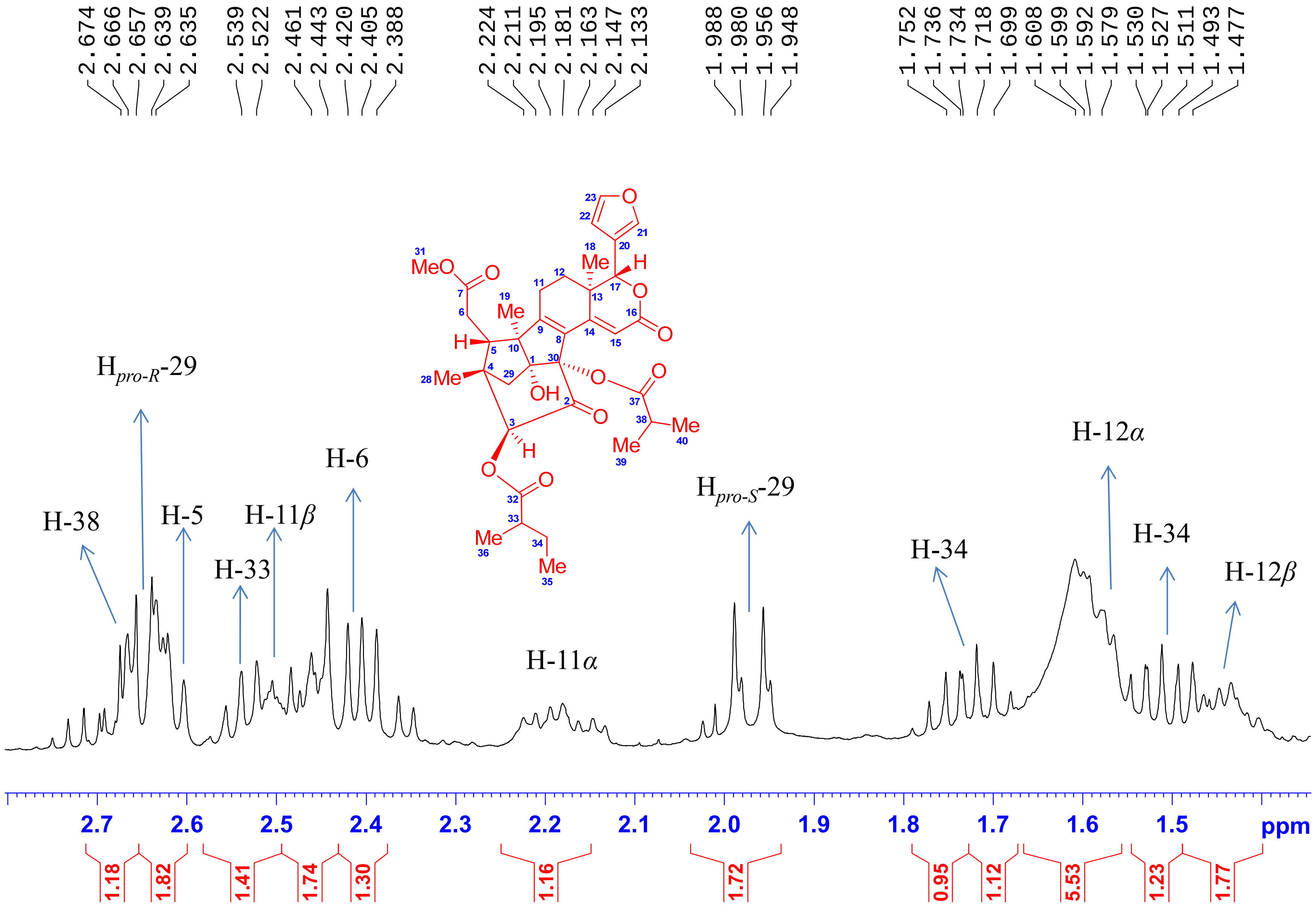

^{1}H NMR (400 MHz) spectrum of Krishnolide B (**2**) in $CDCl_3$

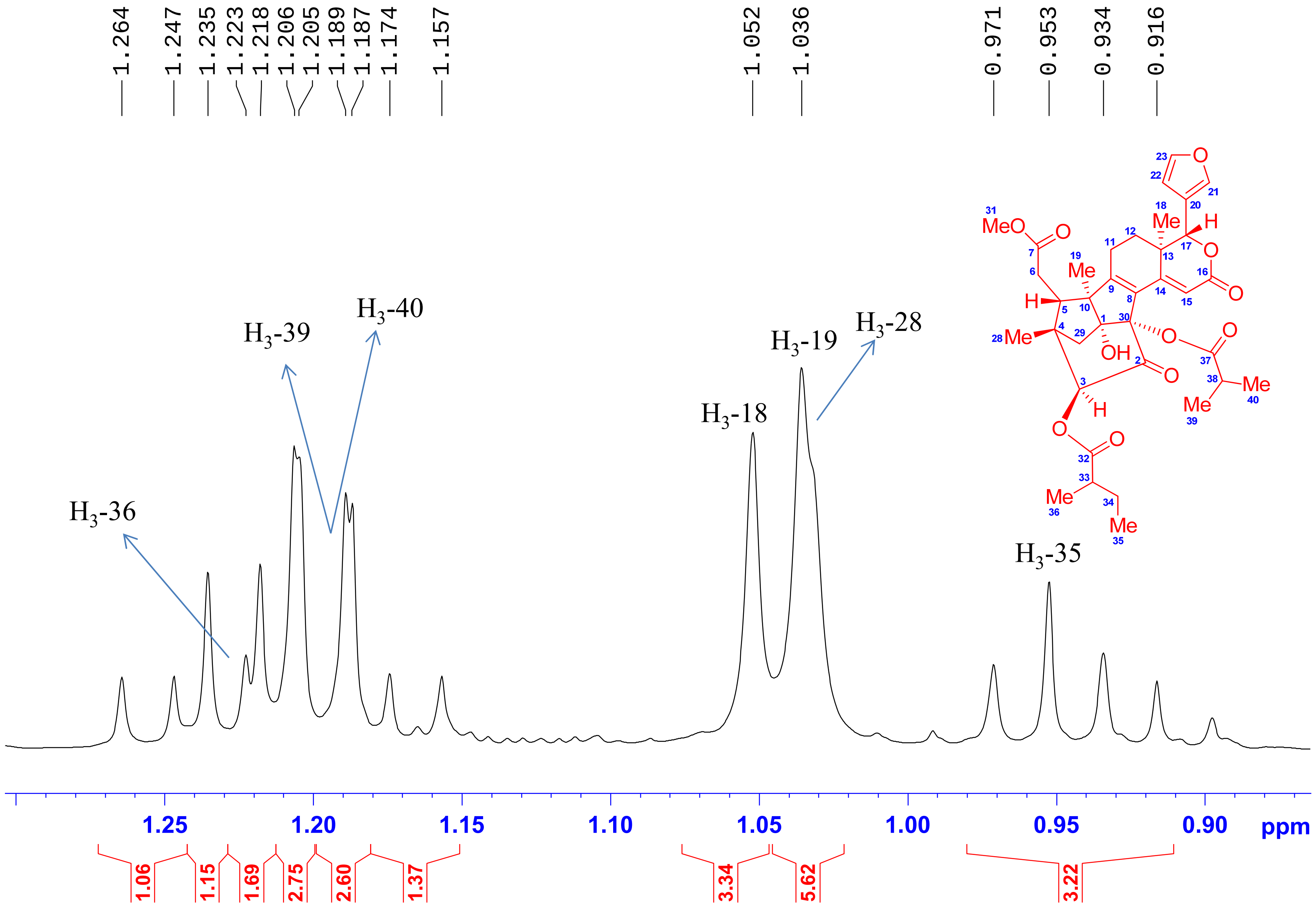

^{13}C NMR (100 MHz) spectrum of Krishnolide B (**2**) in $CDCl_3$

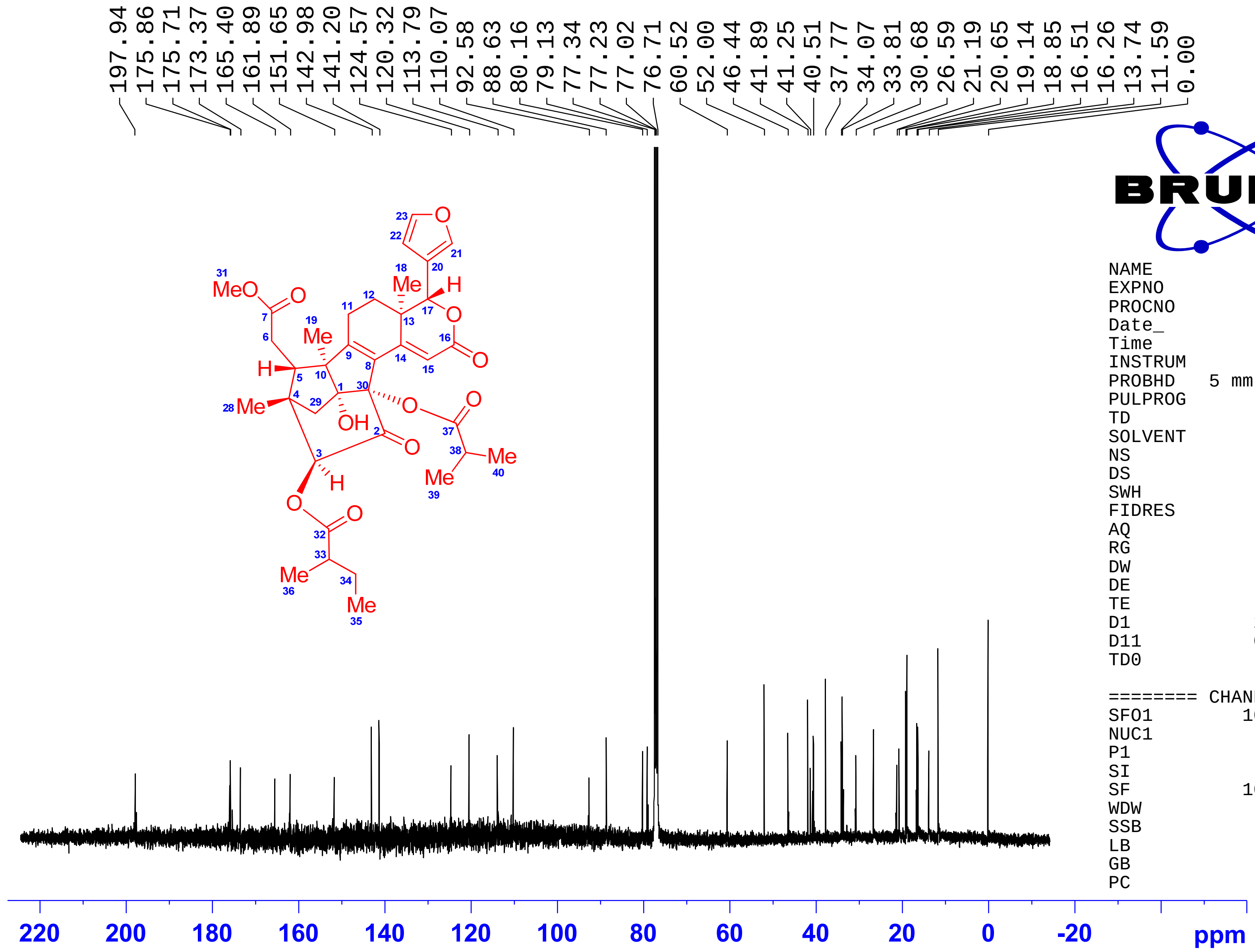

NAME ZQ-19
EXPNO 2
PROCNO 1
Date_ 20170120
Time 7.49
INSTRUM spect
PROBHD 5 mm CPPBBO BB
PULPROG zgpg30
TD 65536
SOLVENT CDCl3
NS 2500
DS 4
SWH 24038.461 Hz
FIDRES 0.366798 Hz
AQ 1.3631988 sec
RG 91.64
DW 20.800 usec
DE 18.00 usec
TE 297.0 K
D1 2.00000000 sec
D11 0.03000000 sec
TD0 1

======== CHANNEL f1 ========
SFO1 100.6233324 MHz
NUC1 13C
P1 10.00 usec
SI 32768
SF 100.6127689 MHz
WDW EM
SSB 0
LB 1.00 Hz
GB 0
PC 1.40

^{13}C NMR (100 MHz) spectrum of Krishnolide B (**2**) in $CDCl_3$

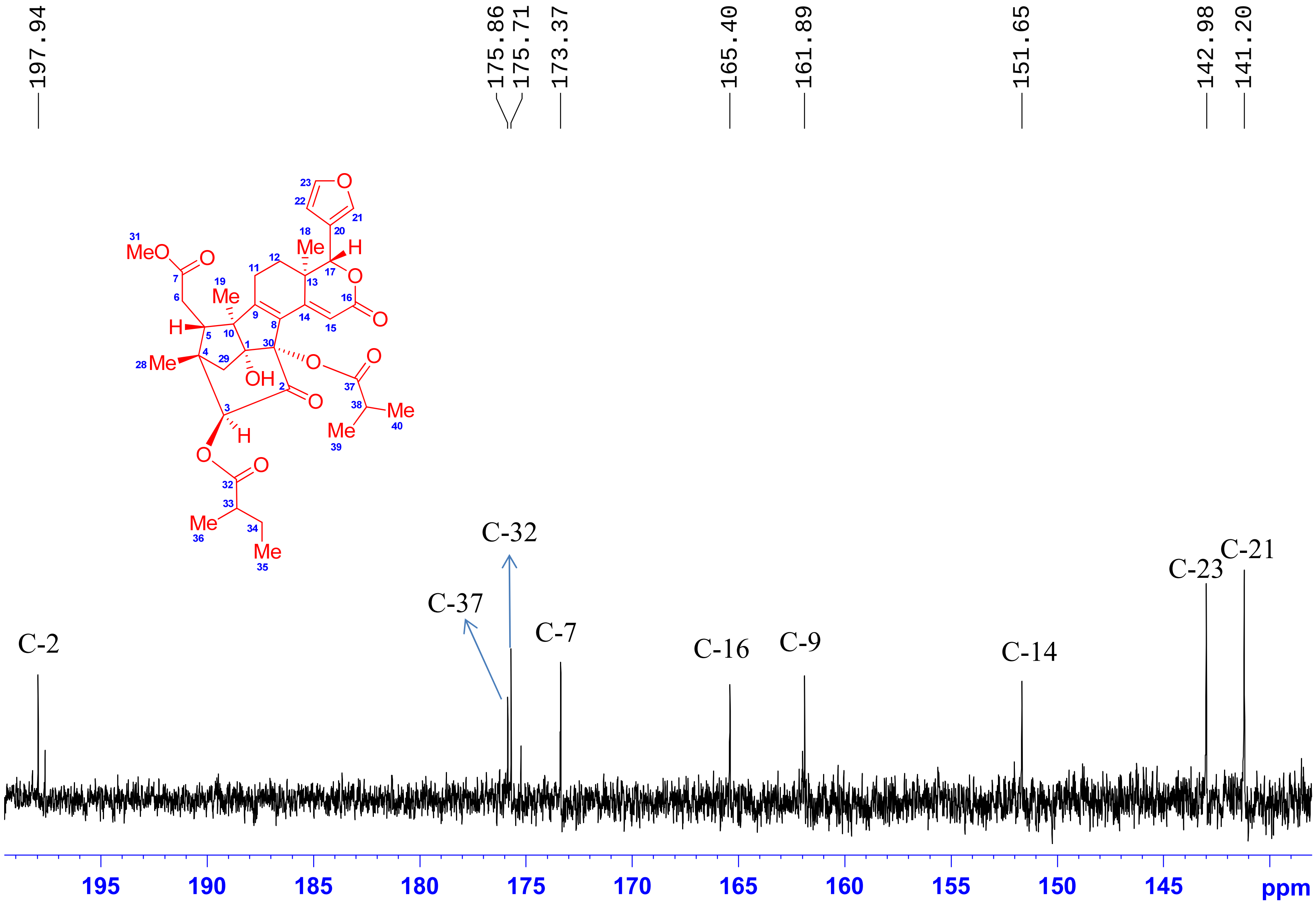

^{13}C NMR (100 MHz) spectrum of Krishnolide B (**2**) in $CDCl_3$

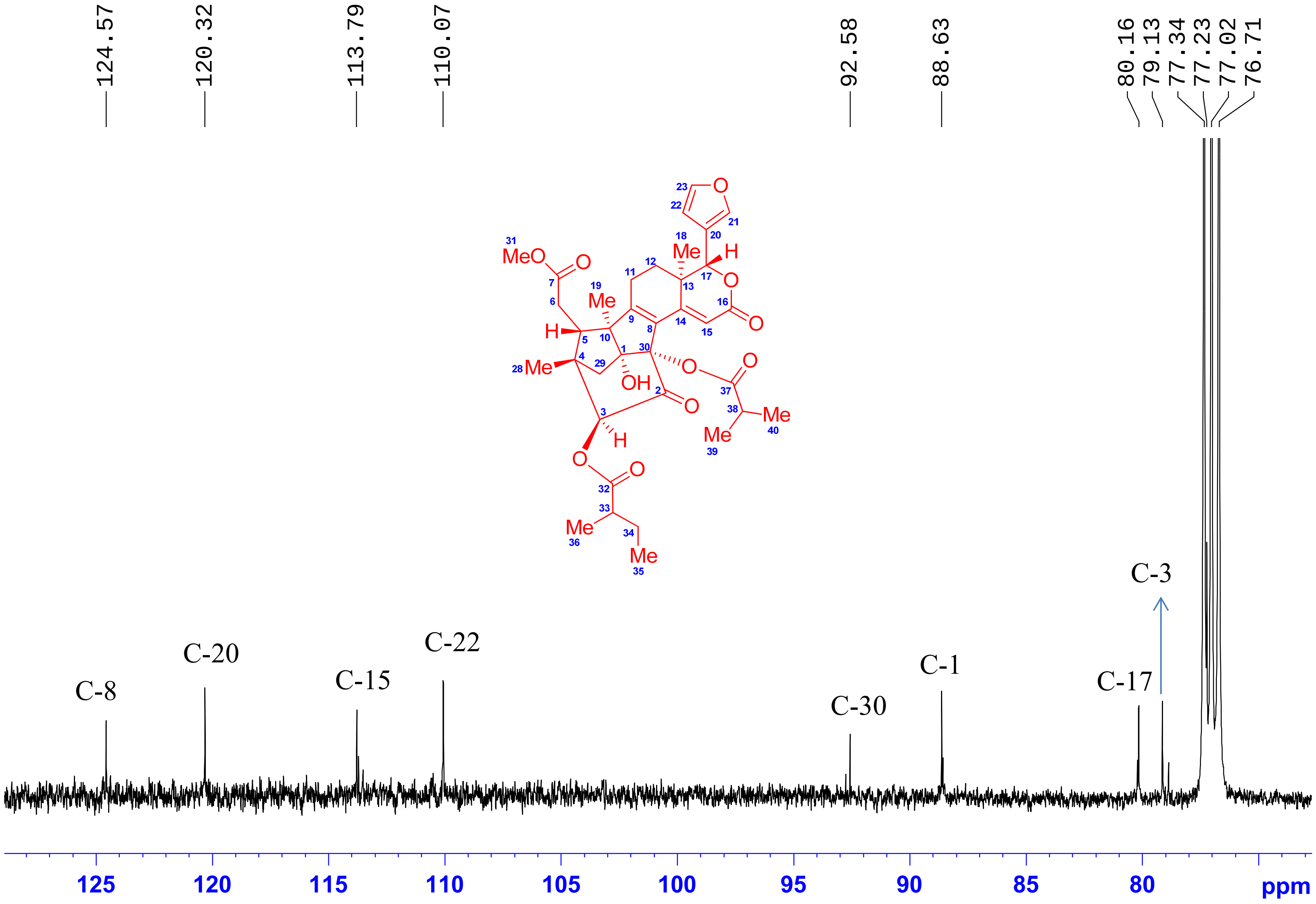

^{13}C NMR (100 MHz) spectrum of Krishnolide B (**2**) in $CDCl_3$

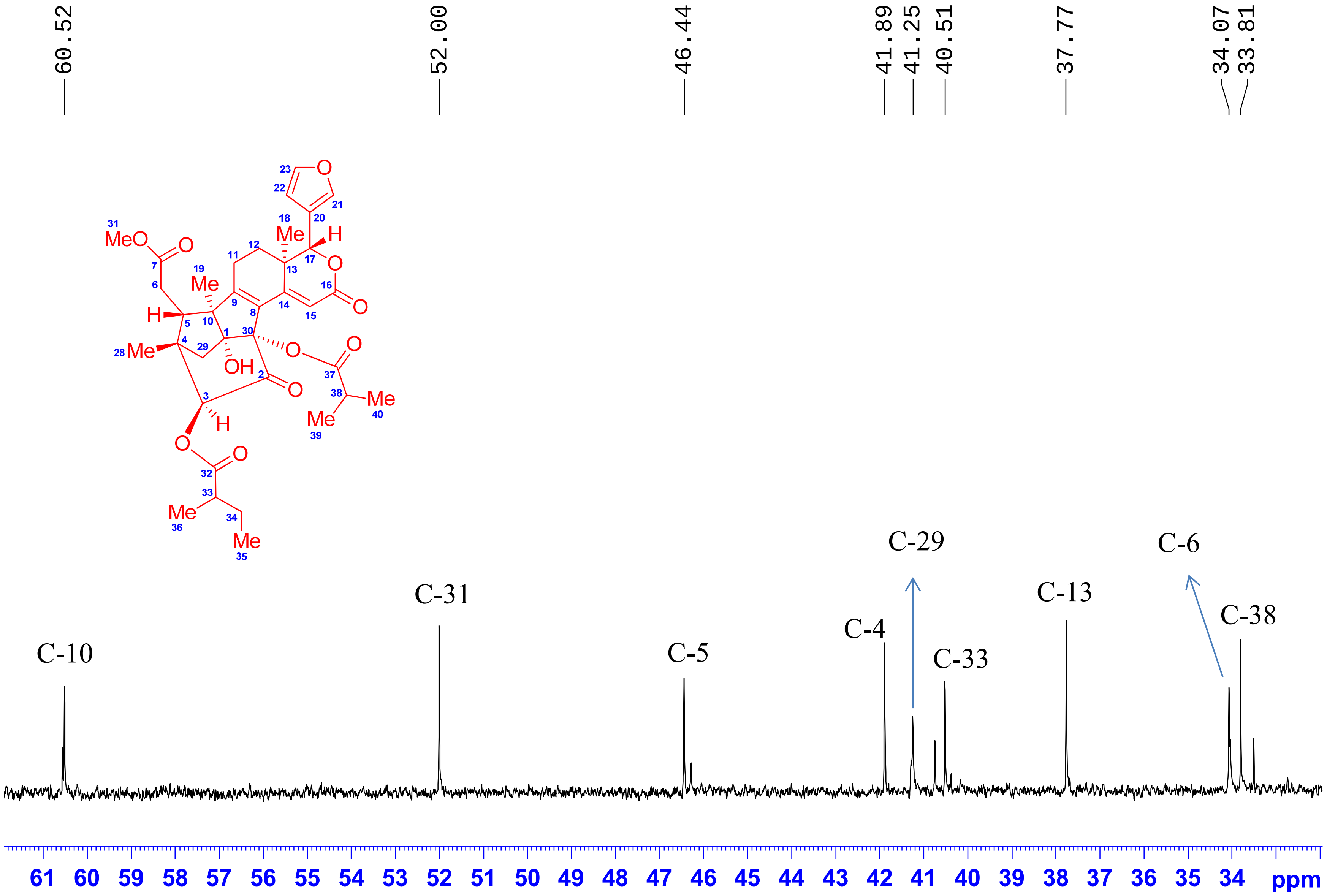

^{13}C NMR (100 MHz) spectrum of Krishnolide B (**2**) in $CDCl_3$

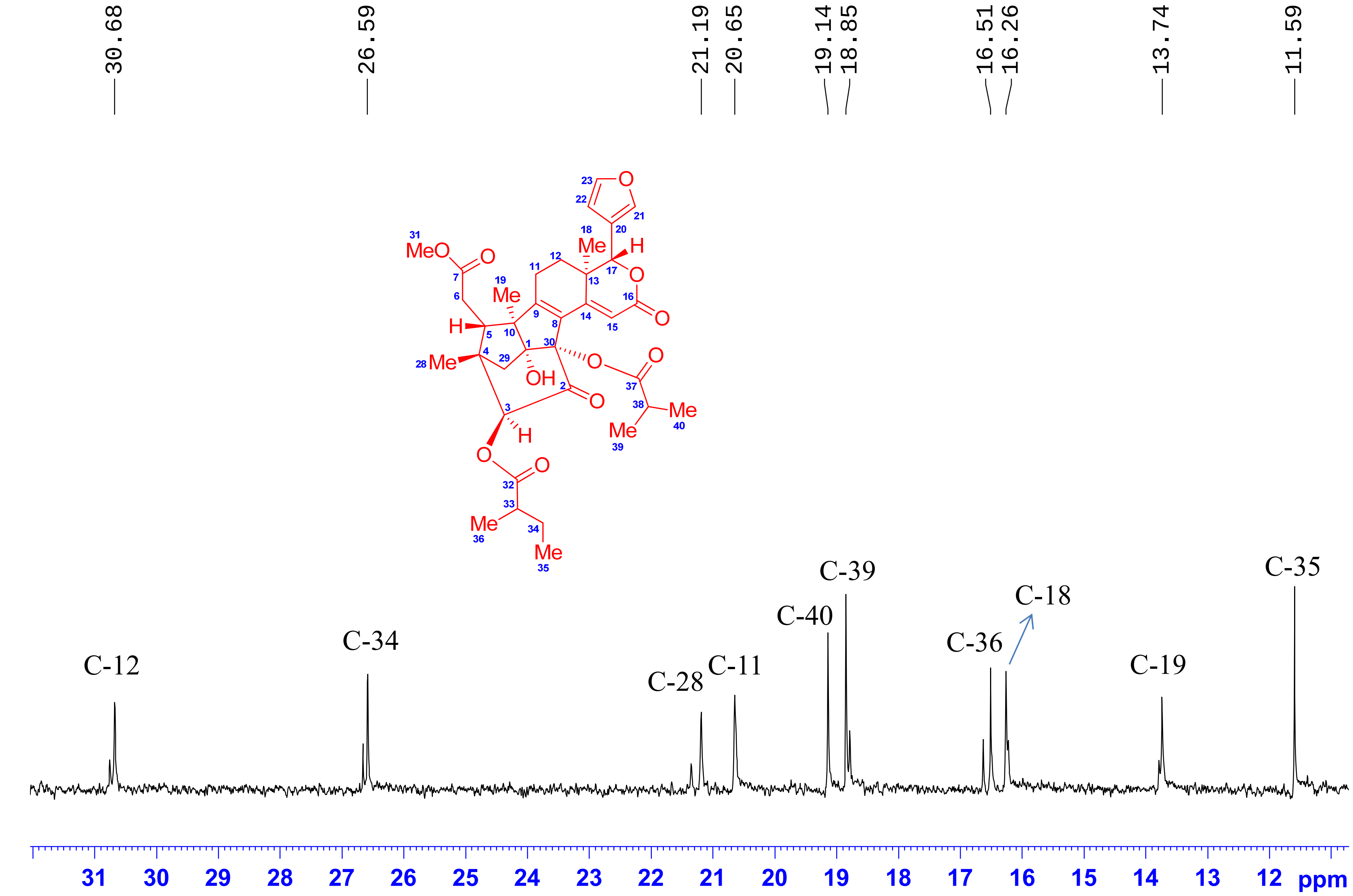

DEPT 135 spectrum of Krishnolide B (**2**) in $CDCl_3$

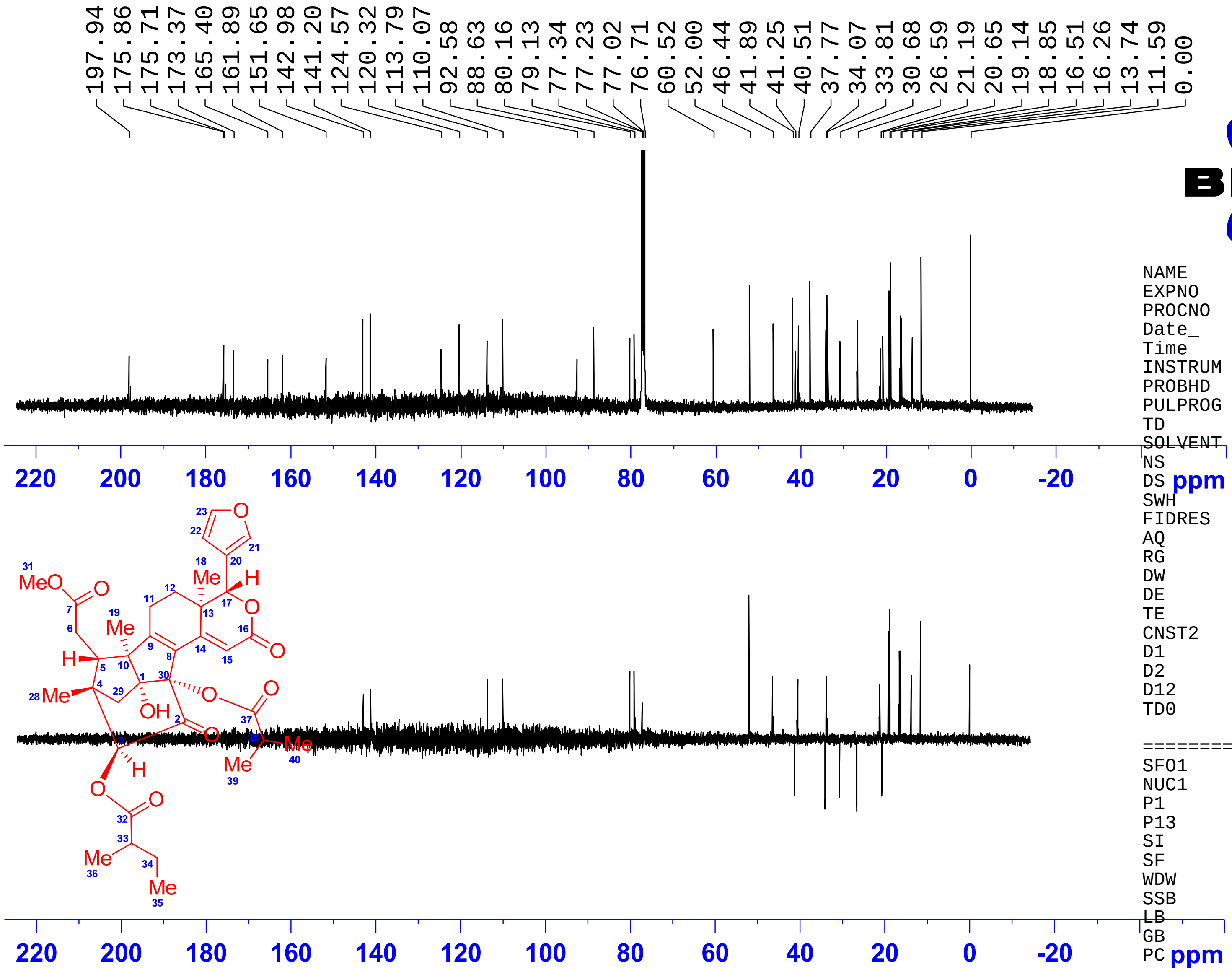

NAME	ZQ-19
EXPNO	3
PROCNO	1
Date_	20170120
Time	8.24
INSTRUM	spect
PROBHD	5 mm CPPBBO BB
PULPROG	deptsp135
TD	65536
SOLVENT	CDCl3
NS	600
DS	4
SWH	24038.461 Hz
FIDRES	0.366798 Hz
AQ	1.3631988 sec
RG	91.64
DW	20.800 usec
DE	18.00 usec
TE	297.0 K
CNST2	145.0000000
D1	2.00000000 sec
D2	0.00344828 sec
D12	0.00002000 sec
TD0	1

======== CHANNEL f1 ========

SFO1	100.6233324 MHz
NUC1	13C
P1	10.00 usec
P13	2000.00 usec
SI	32768
SF	100.6127689 MHz
WDW	EM
SSB	0
LB	1.00 Hz
GB	0
PC	1.40

DEPT 135 spectrum of Krishnolide B (**2**) in $CDCl_3$

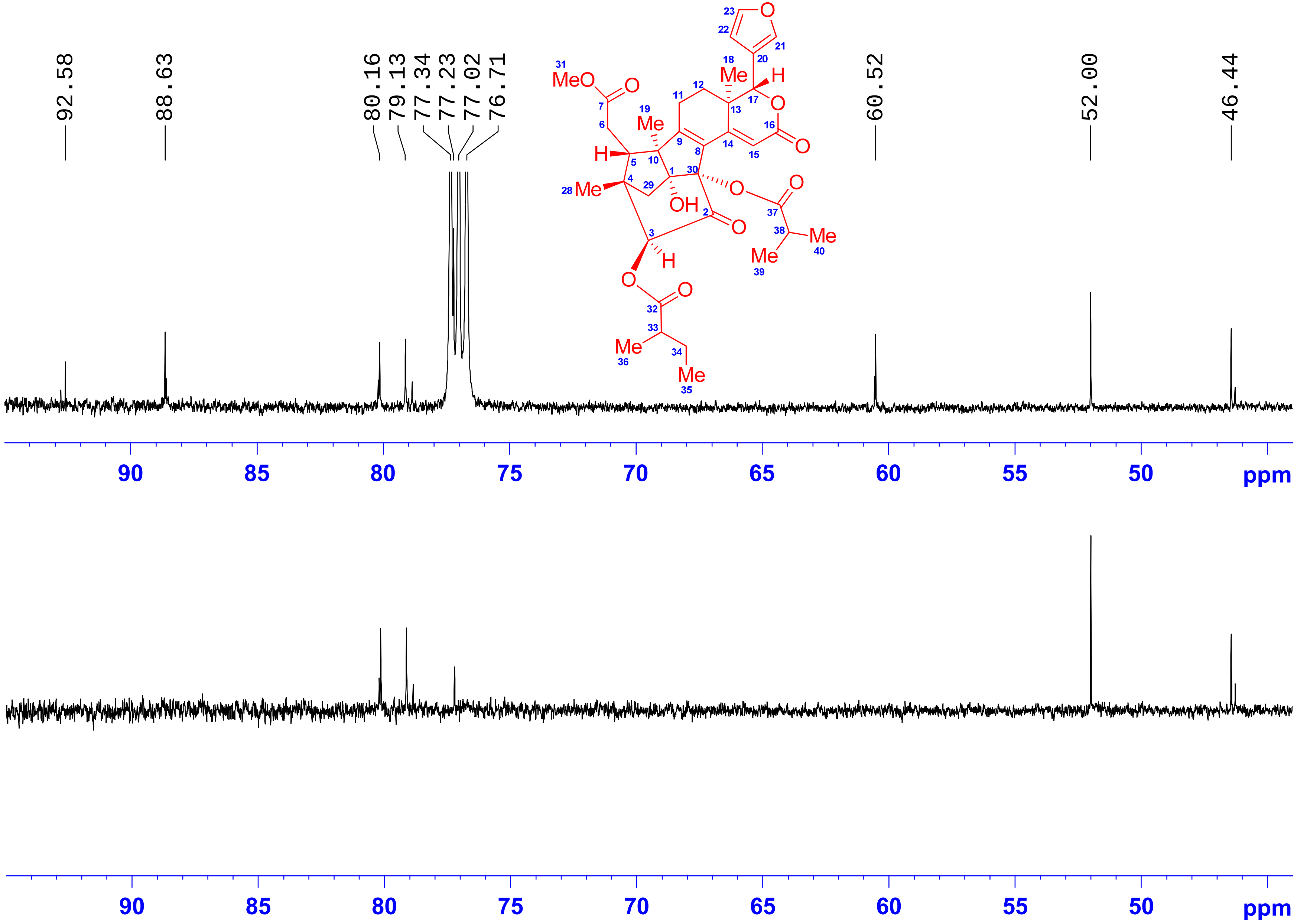

DEPT 135 spectrum of Krishnolide B (**2**) in $CDCl_3$

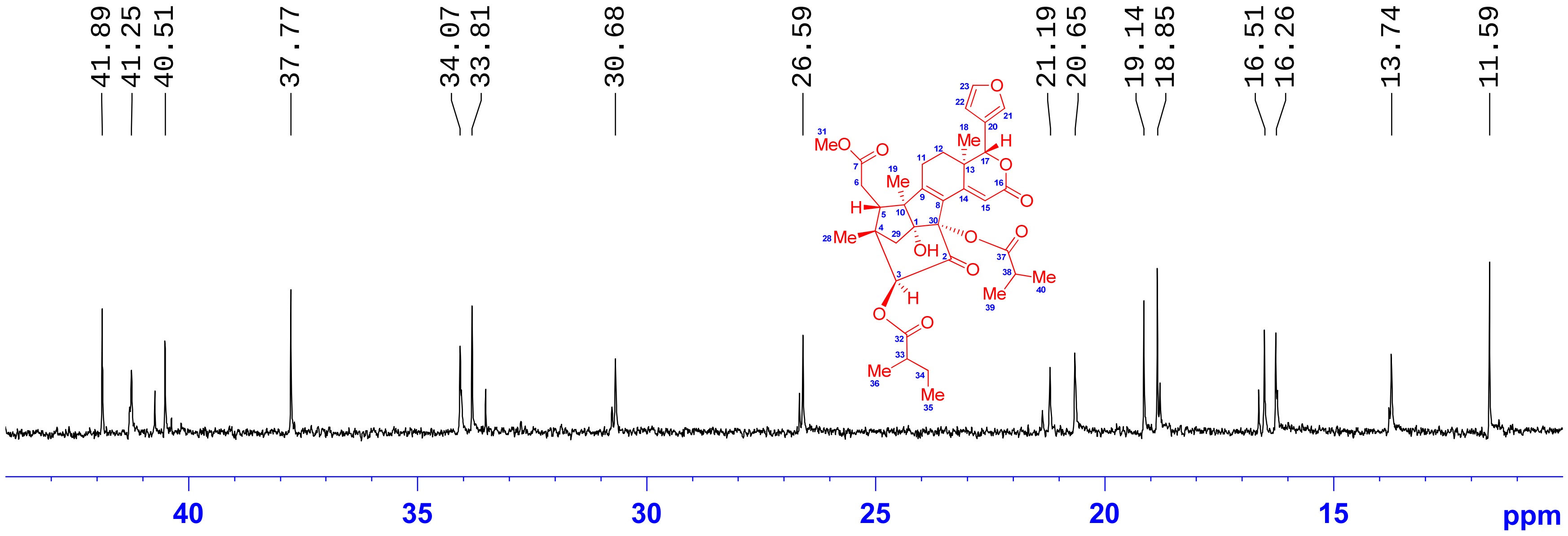

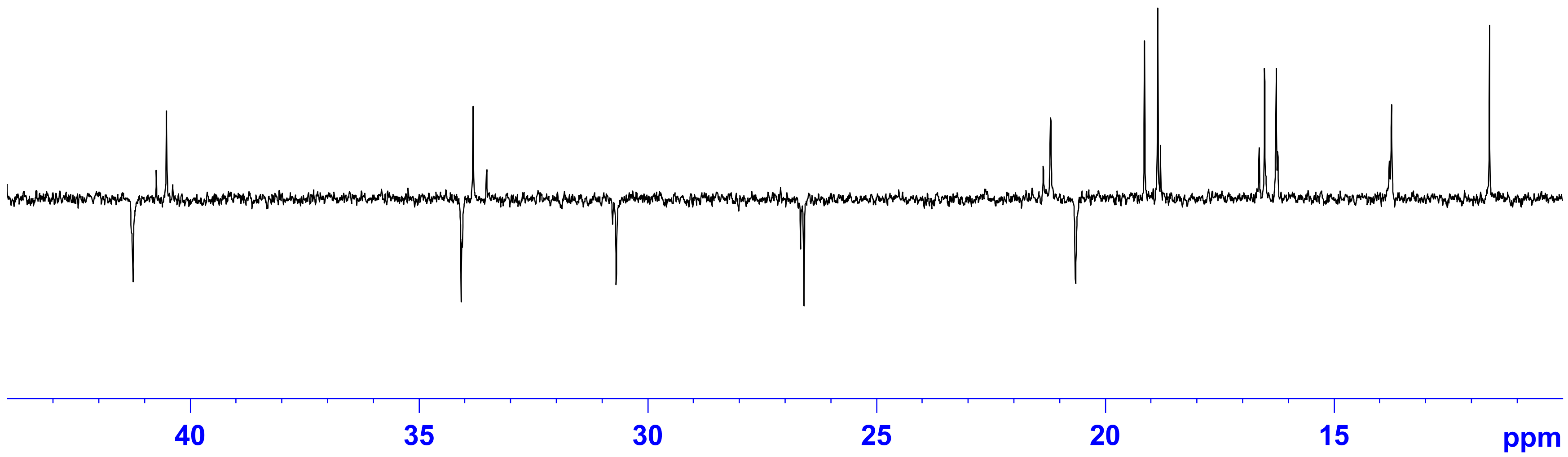

^{1}H-^{1}H COSY spectrum of Krishnolide B (**2**) in $CDCl_3$

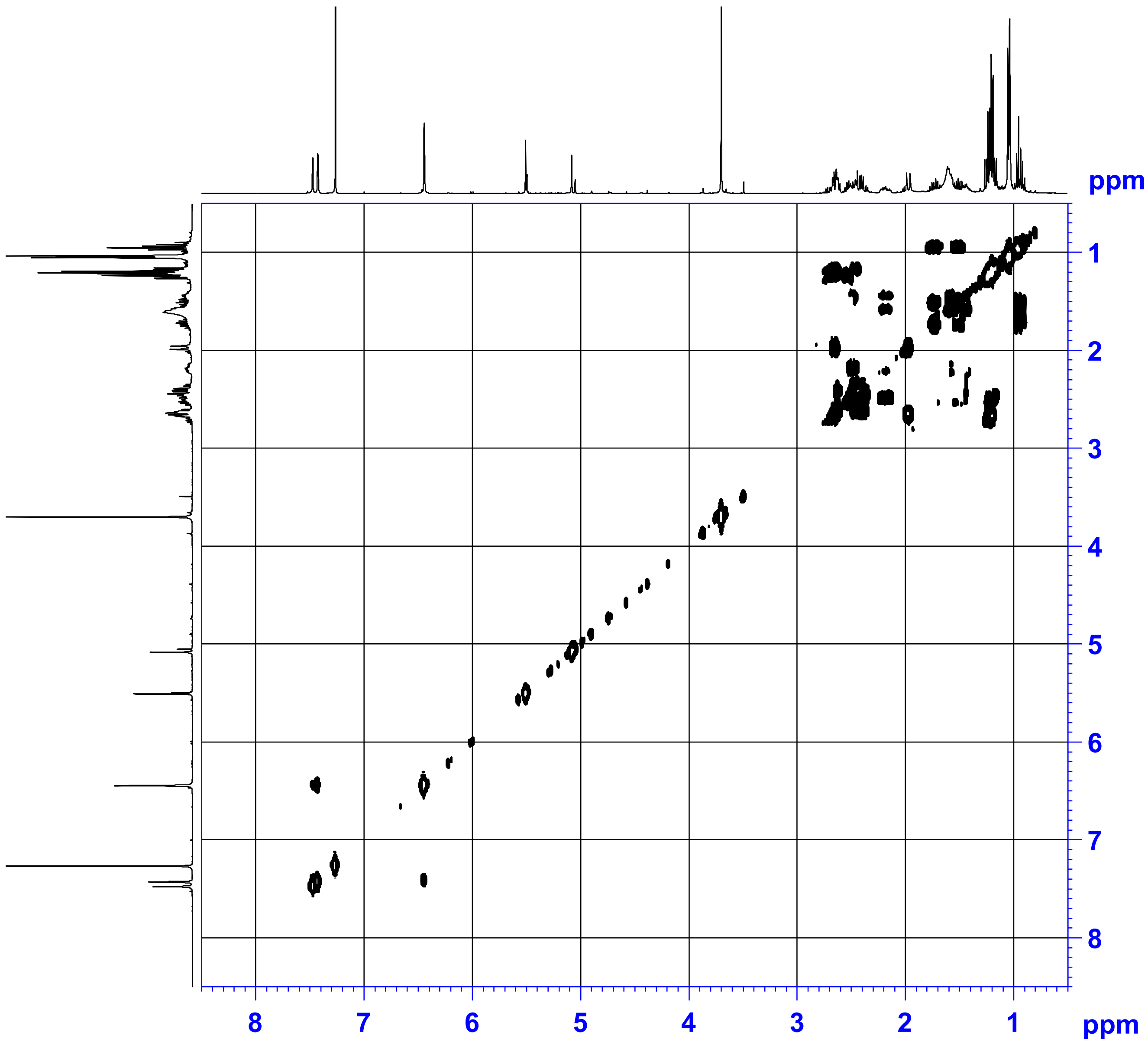

NAME	ZQ-19
EXPNO	4
PROCNO	1
Date_	20170121
Time	15.30
INSTRUM	spect
PROBHD	5 mm CPPBBO BB
PULPROG	cosygpppqf
TD	2048
SOLVENT	CDCl3
NS	8
DS	8
SWH	3906.250 Hz
FIDRES	1.907349 Hz
AQ	0.2621940 sec
RG	208.5
DW	128.000 usec
DE	10.00 usec
TE	297.0 K
D0	0.00000300 sec
D1	1.89678097 sec
D11	0.03000000 sec
D12	0.00002000 sec
D13	0.00000400 sec
D16	0.00020000 sec
IN0	0.00025600 sec

======== CHANNEL f1 ========

SFO1	400.1318006 MHz
NUC1	1H
P0	11.50 usec
P1	11.50 usec
P17	2500.00 usec
ND0	1
TD	128
SFO1	400.1318 MHz
FIDRES	30.517578 Hz
SW	9.762 ppm
FnMODE	QF
SI	1024
SF	400.1300061 MHz
WDW	QSINE
SSB	0
LB	0.00 Hz
GB	0
PC	1.40
SI	1024
MC2	QF
SF	400.1300061 MHz
WDW	QSINE
SSB	0
LB	0.00 Hz
GB	0

^{1}H-^{1}H COSY spectrum of Krishnolide B (**2**) in $CDCl_3$

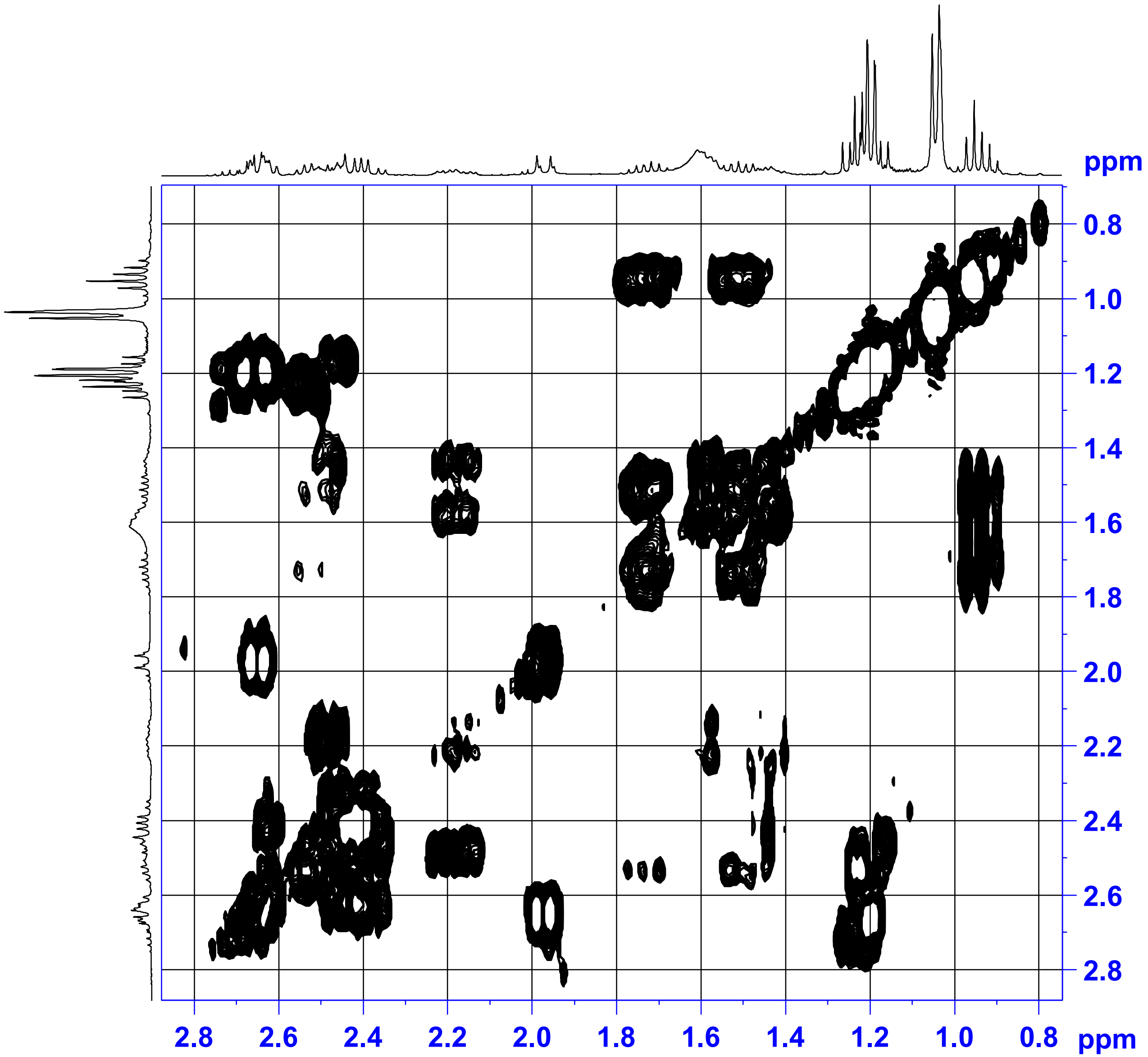

^{1}H-^{1}H COSY spectrum of Krishnolide B (**2**) in $CDCl_3$

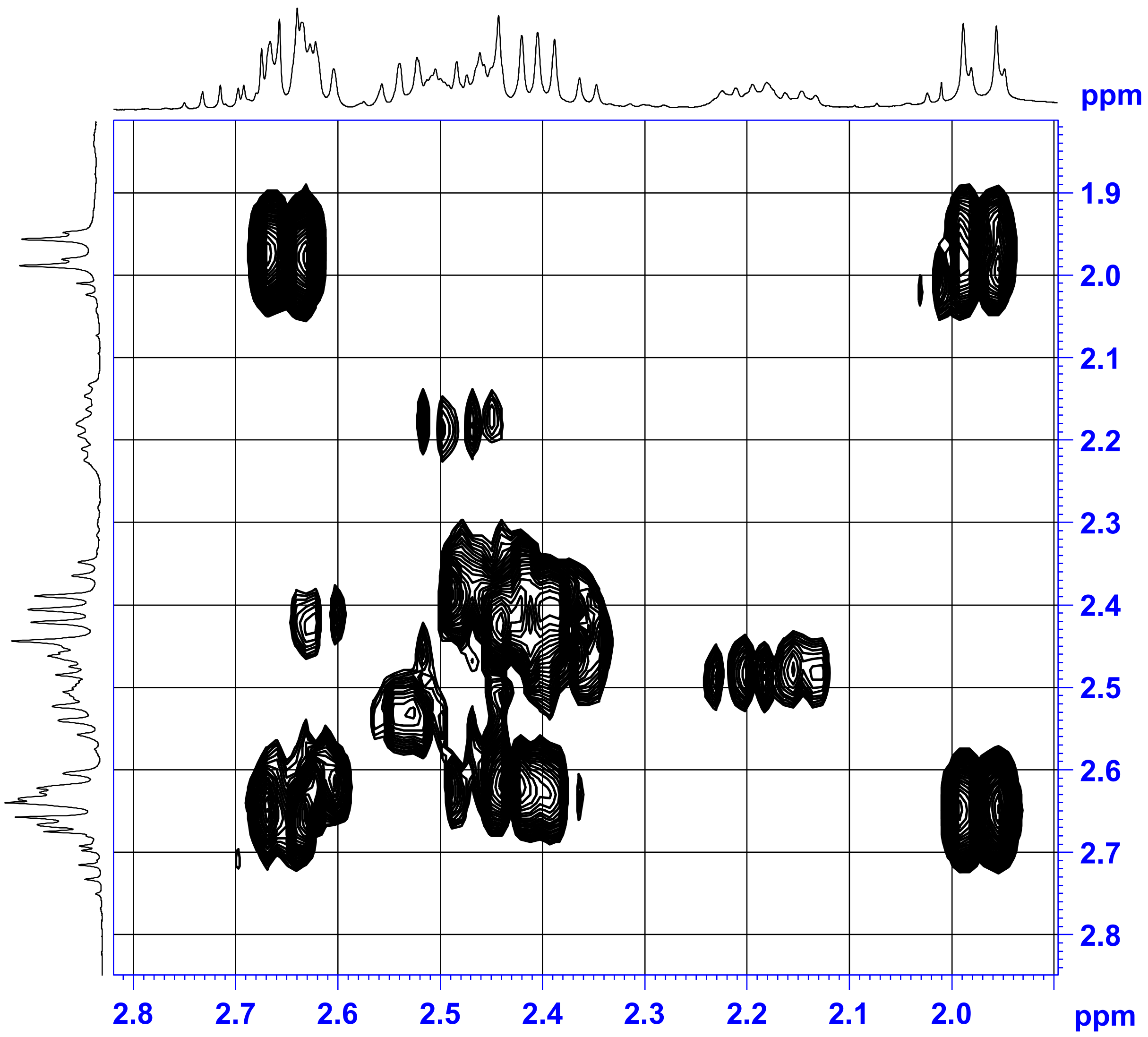

^{1}H-^{1}H COSY spectrum of Krishnolide B (**2**) in $CDCl_3$

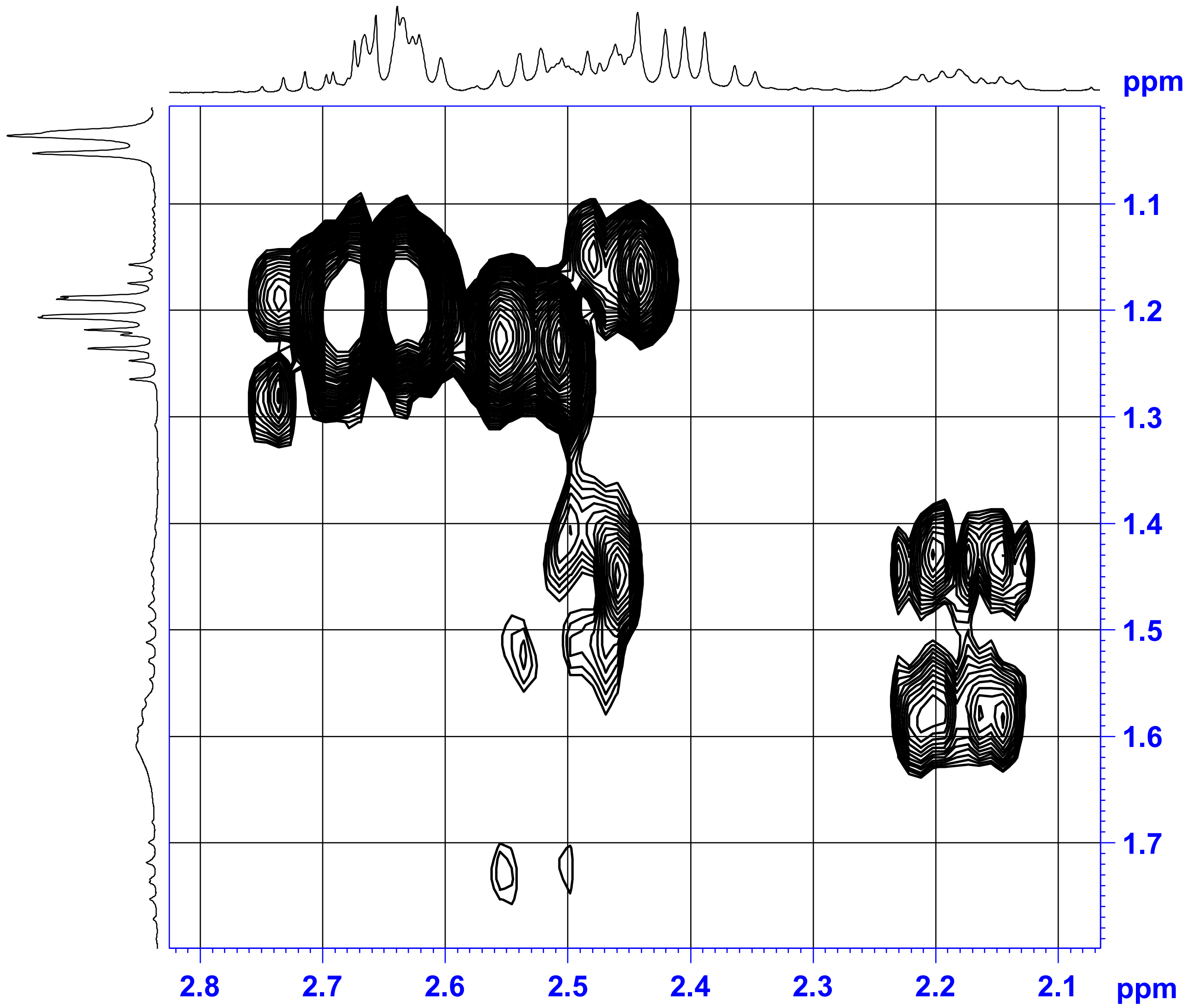

^{1}H-^{1}H COSY spectrum of Krishnolide B (**2**) in $CDCl_3$

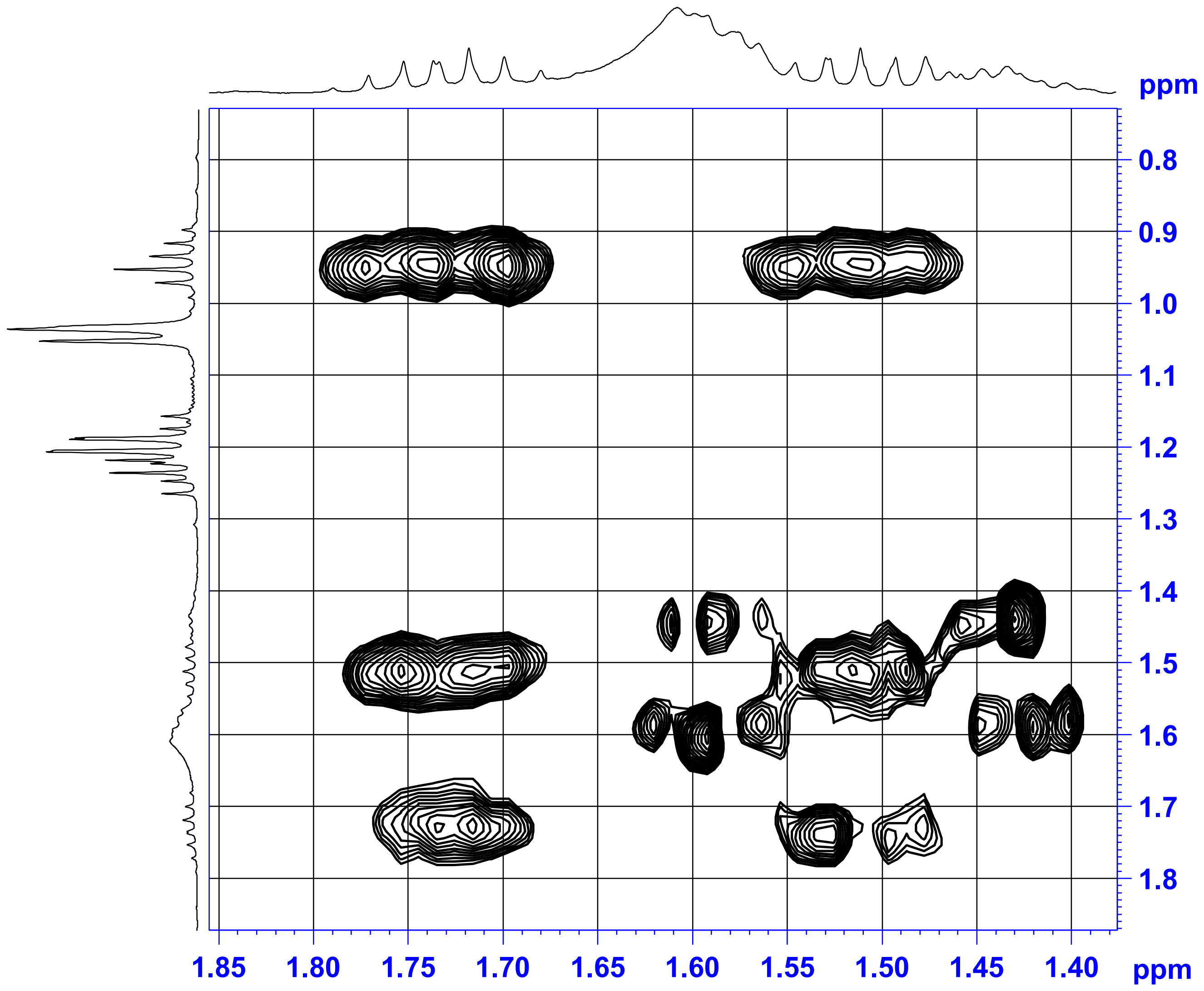

HSQC spectrum of Krishnolide B (**2**) in $CDCl_3$

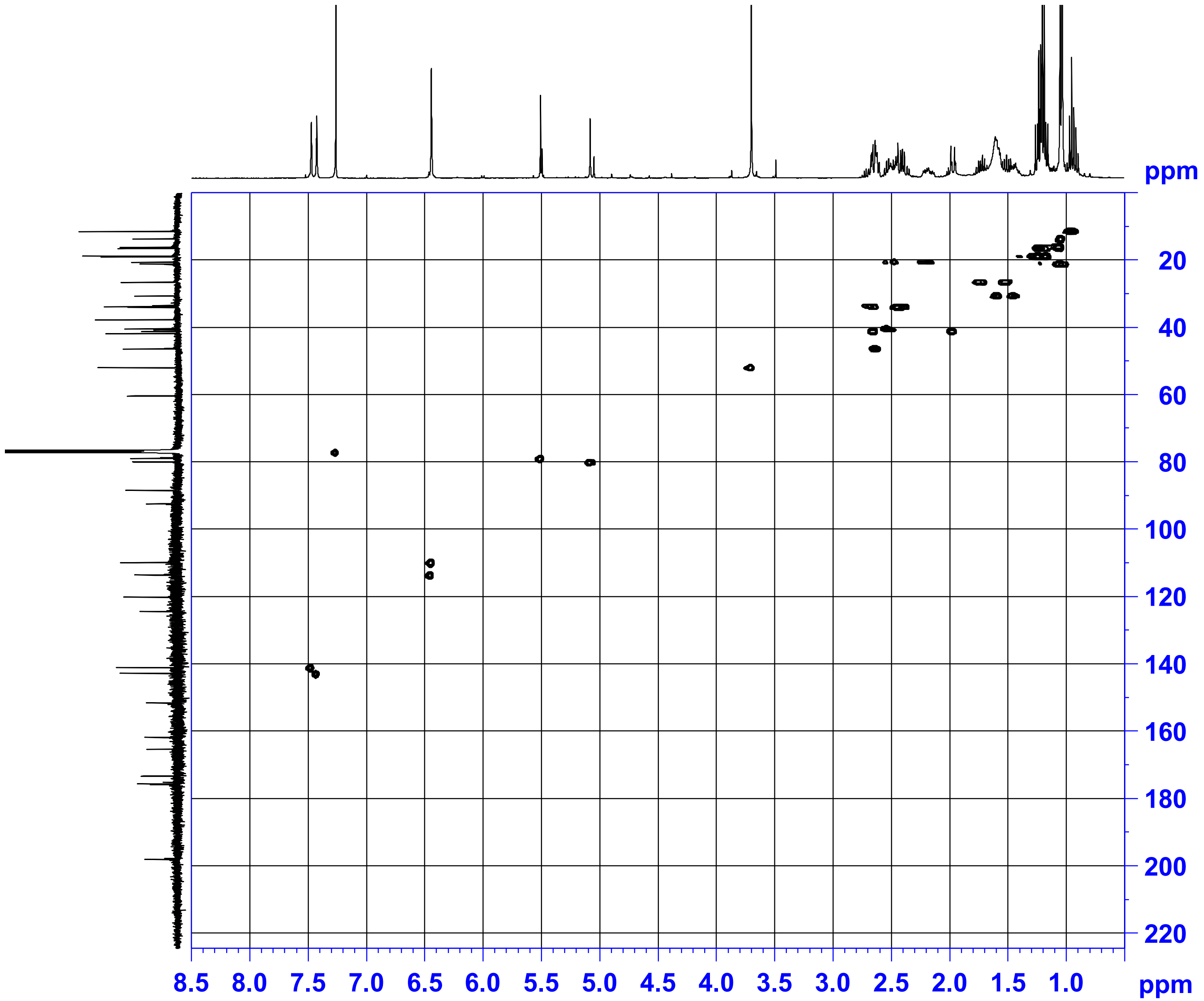

NAME	ZQ-19
EXPNO	5
PROCNO	1
Date_	20170121
Time	16.09
INSTRUM	spect
PROBHD	5 mm CPPBBO BB
PULPROG	hsqcetgpsi2
TD	1024
SOLVENT	CDCl3
NS	16
DS	16
SWH	4302.926 Hz
FIDRES	4.202076 Hz
AQ	0.1190388 sec
RG	208.5
DW	116.200 usec
DE	10.00 usec
TE	297.0 K
CNST2	145.0000000
D0	0.00000300 sec
D1	1.46497905 sec
D4	0.00172414 sec
D11	0.03000000 sec
D16	0.00020000 sec
D24	0.00086207 sec
IN0	0.00002080 sec
ZGOPTNS	

======== CHANNEL f1 ========

SFO1	400.1320007 MHz
NUC1	1H
P1	11.50 usec
P2	23.00 usec
P28	0.00 usec
ND0	2
TD	256
SFO1	100.6233 MHz
FIDRES	93.900238 Hz
SW	238.896 ppm
FnMODE	Echo-Antiecho
SI	1024
SF	400.1300061 MHz
WDW	QSINE
SSB	2
LB	0.00 Hz
GB	0
PC	1.40
SI	1024
MC2	echo-antiecho
SF	100.6127562 MHz
WDW	QSINE
SSB	2
LB	0.00 Hz
GB	0

HSQC spectrum of Krishnolide B (**2**) in $CDCl_3$

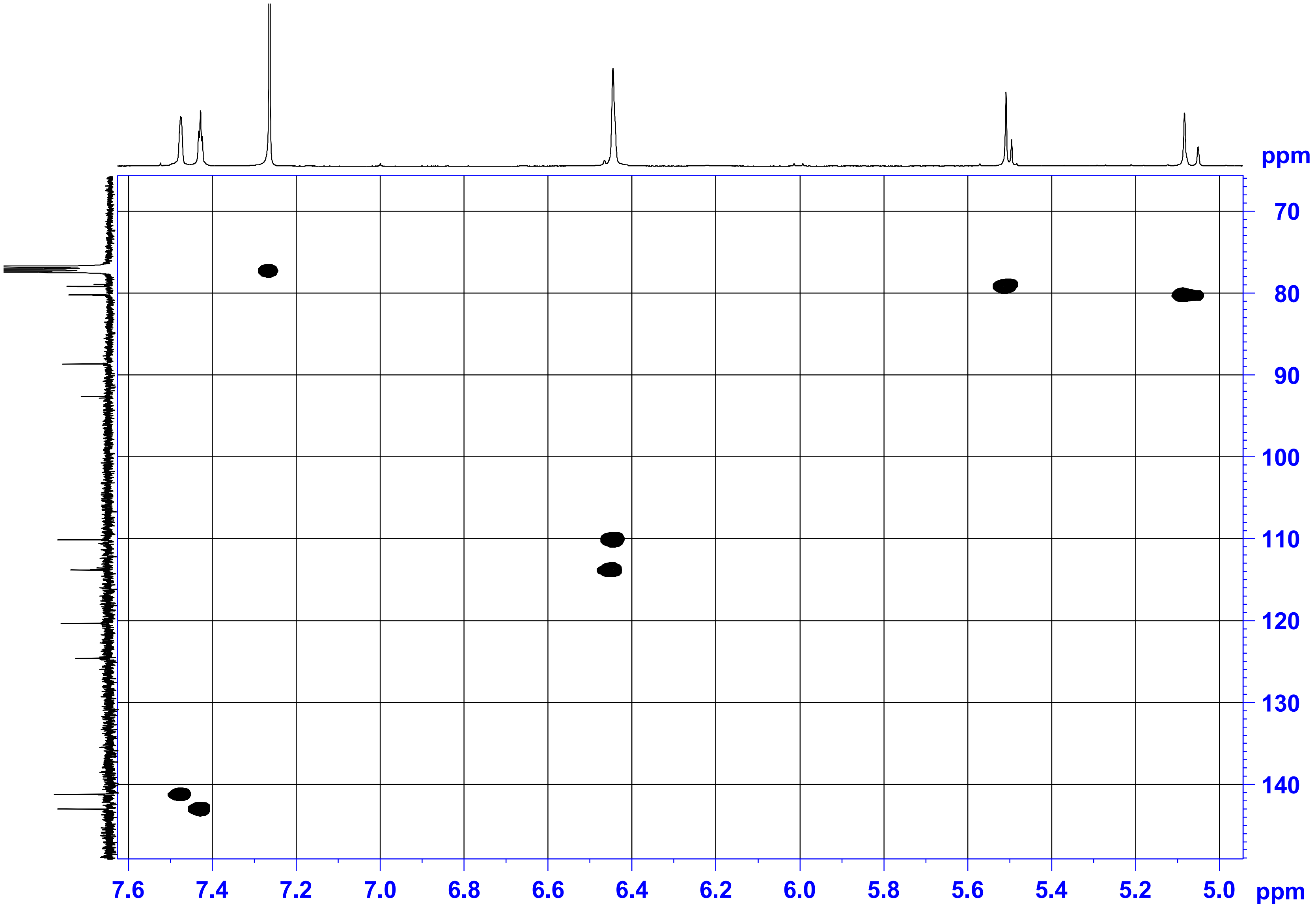

HSQC spectrum of Krishnolide B (**2**) in $CDCl_3$

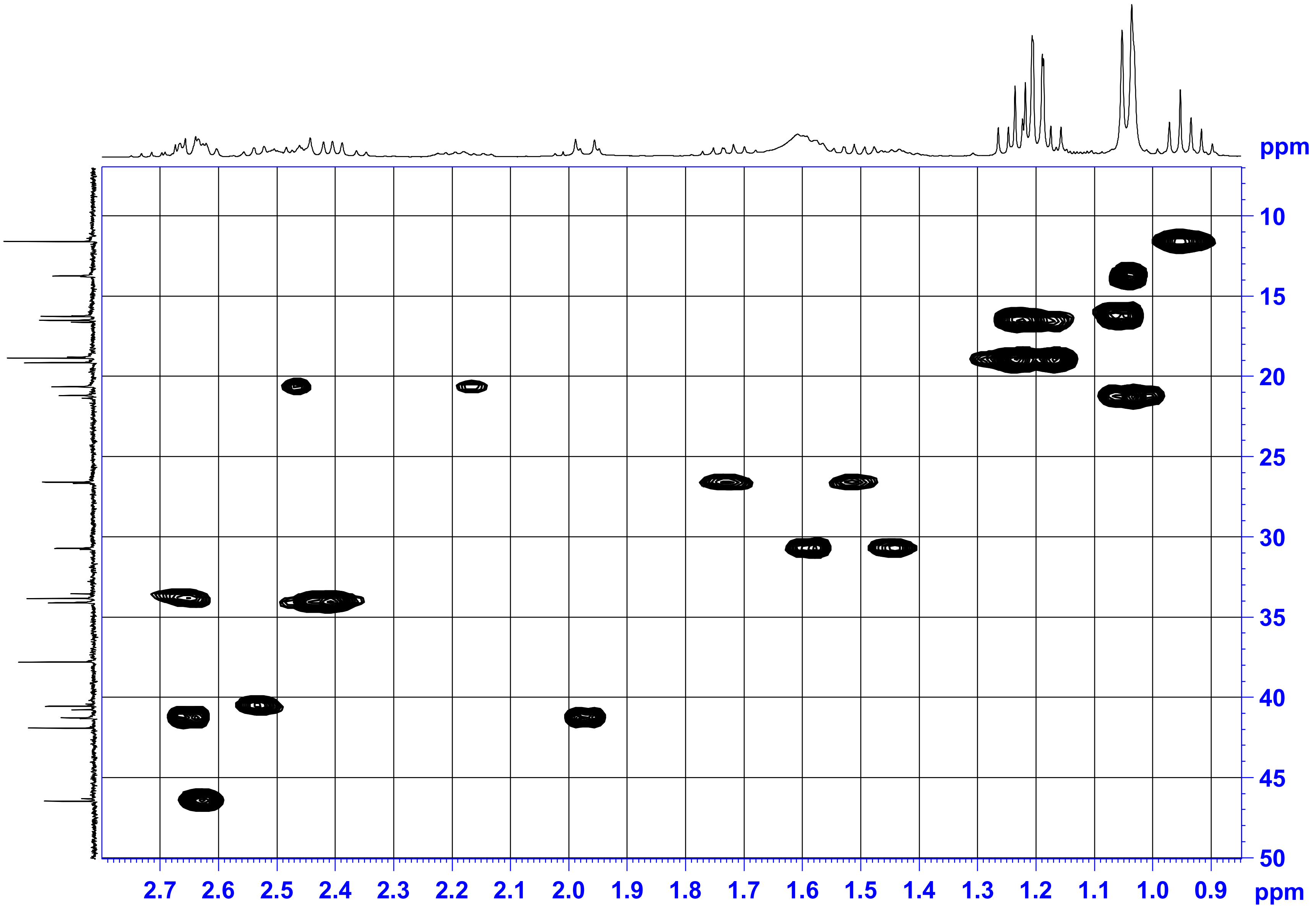

HSQC spectrum of Krishnolide B (**2**) in $CDCl_3$

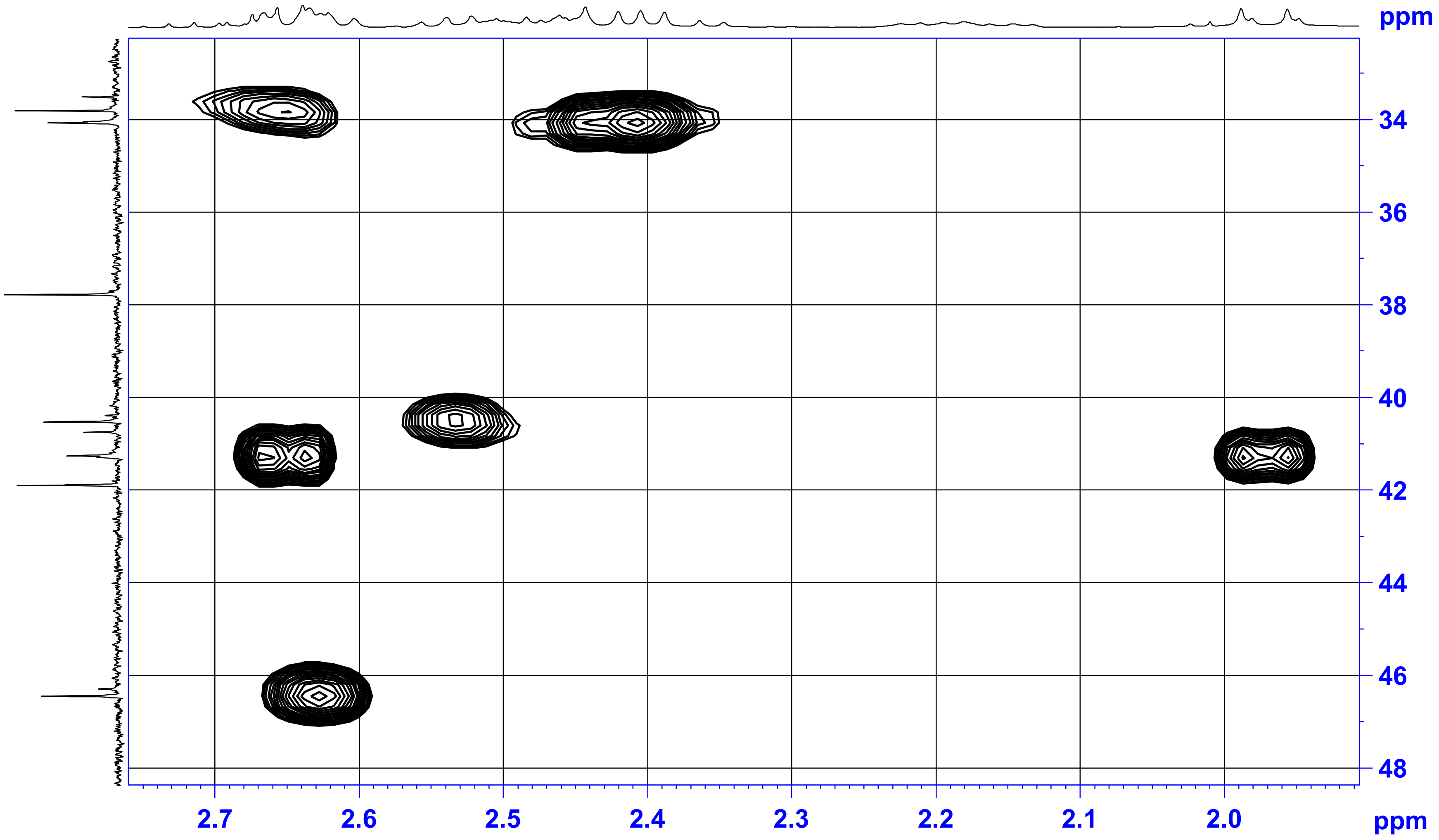

HSQC spectrum of Krishnolide B (**2**) in $CDCl_3$

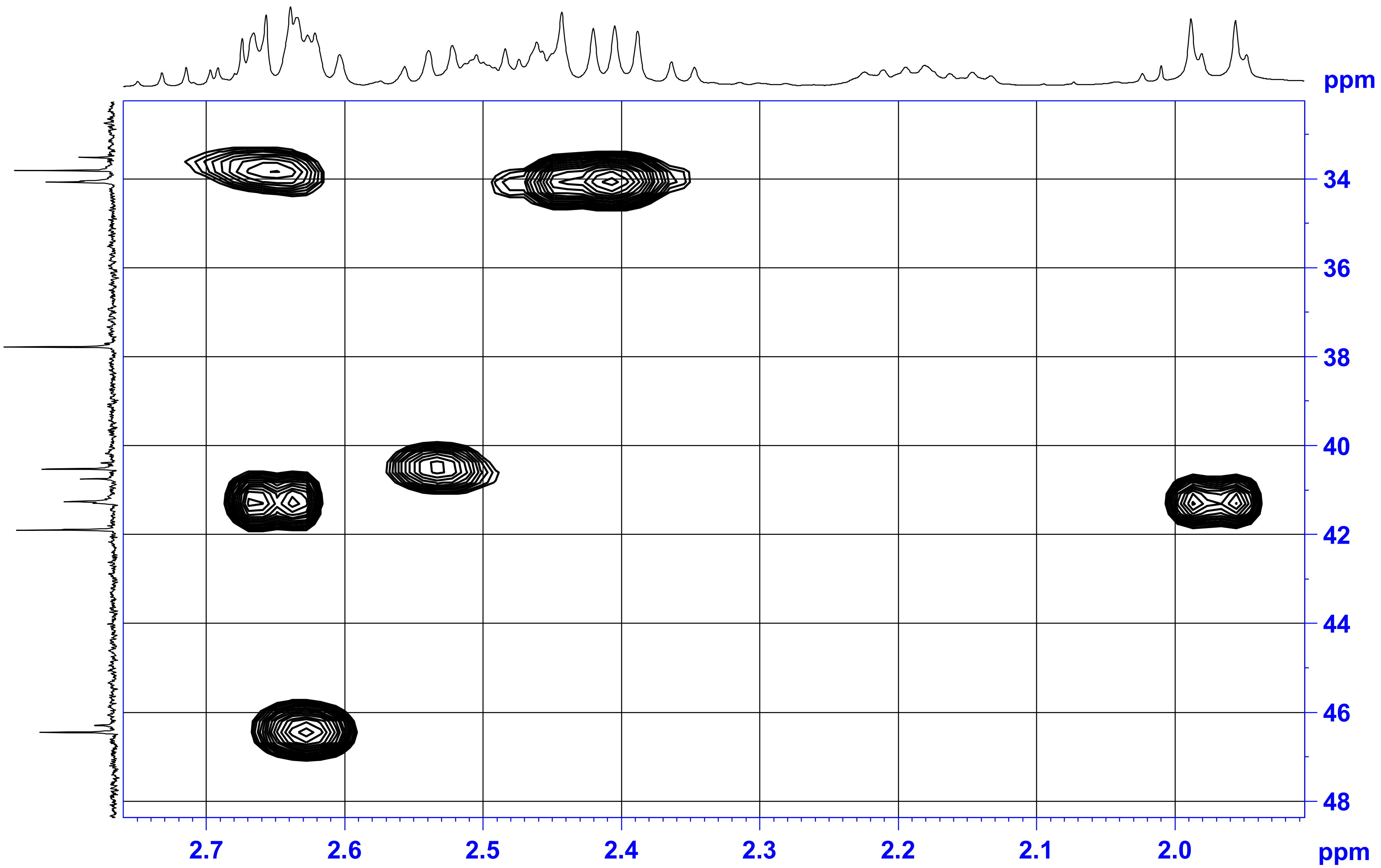

HSQC spectrum of Krishnolide B (**2**) in $CDCl_3$

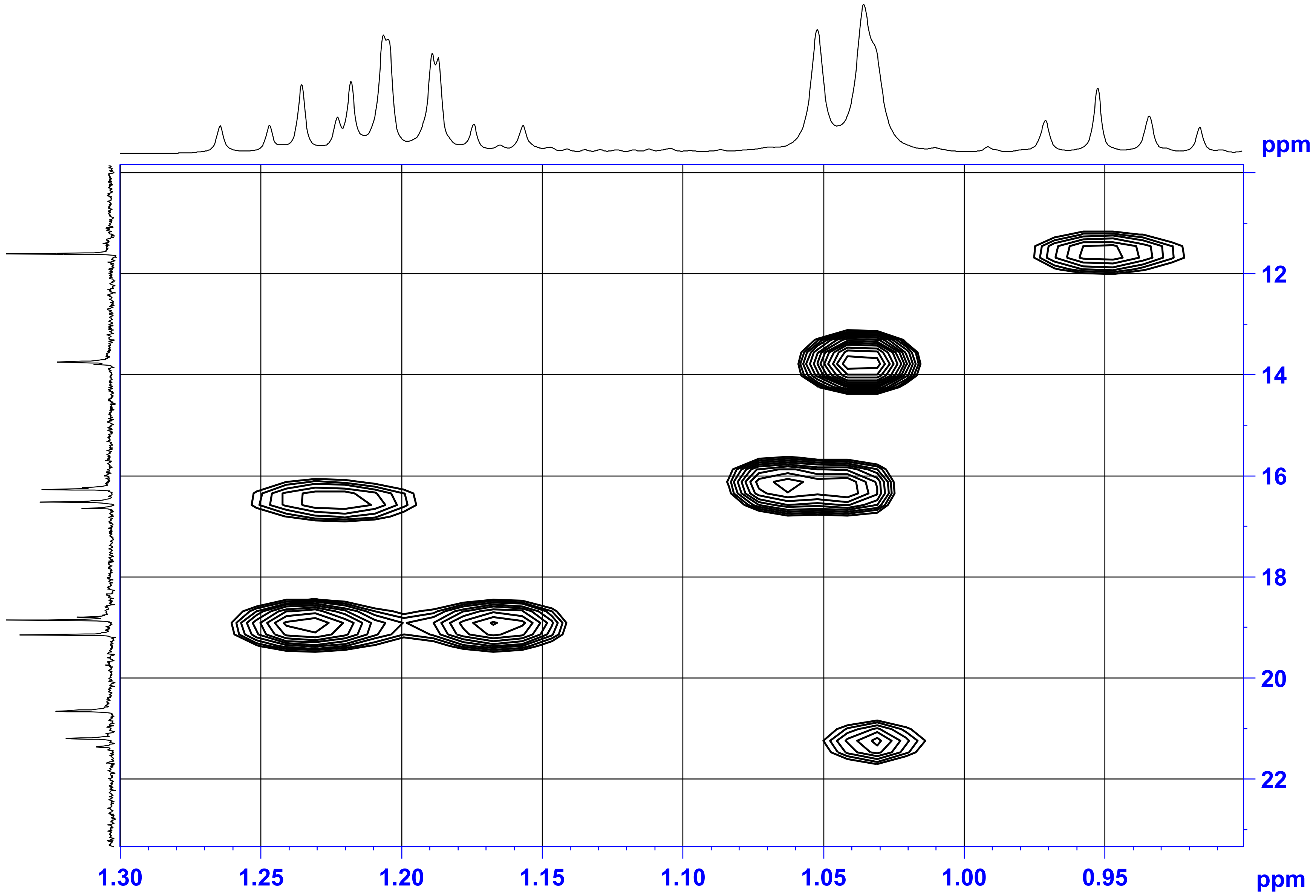

HMBC spectrum of Krishnolide B (**2**) in $CDCl_3$

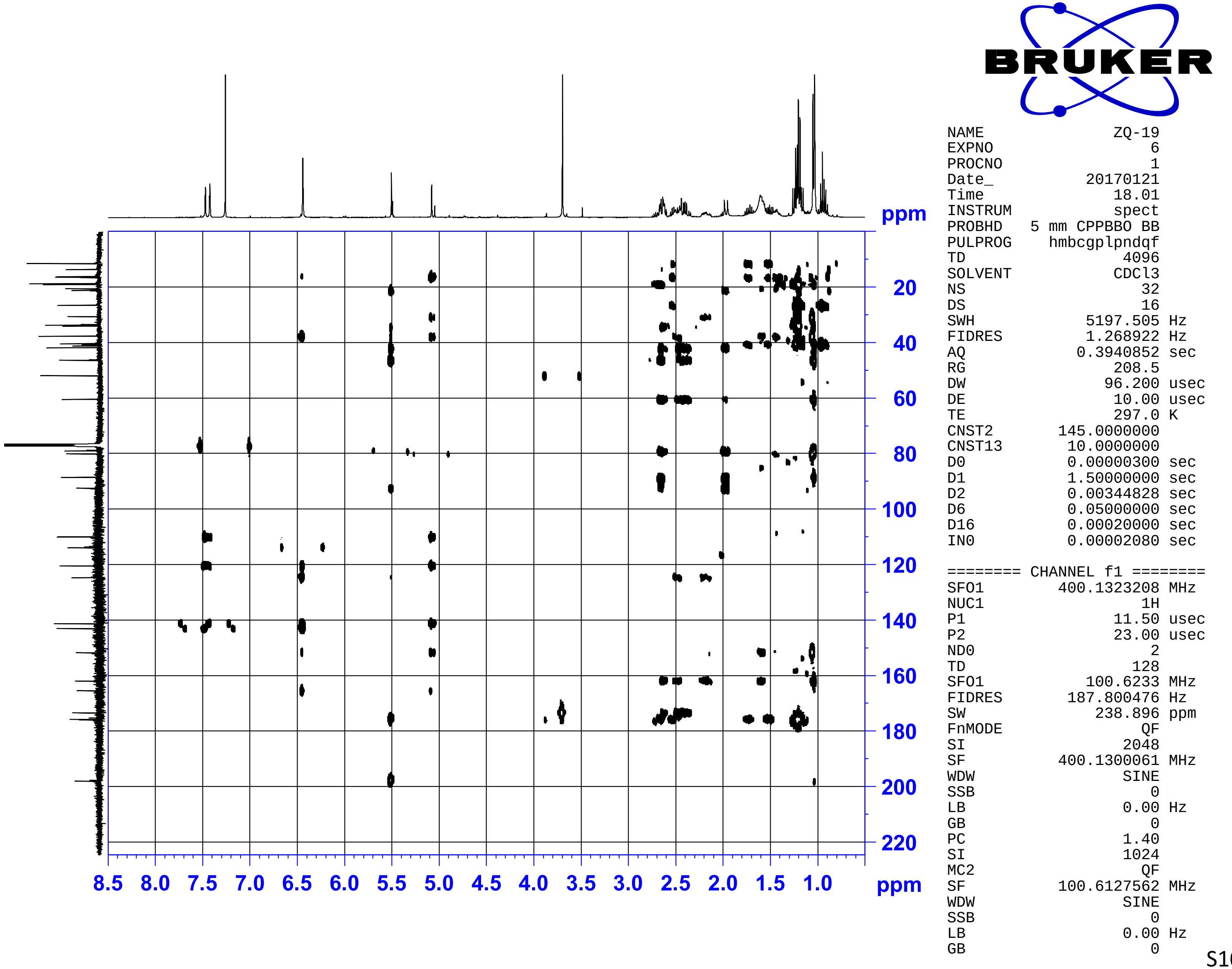

HMBC spectrum of Krishnolide B (**2**) in $CDCl_3$

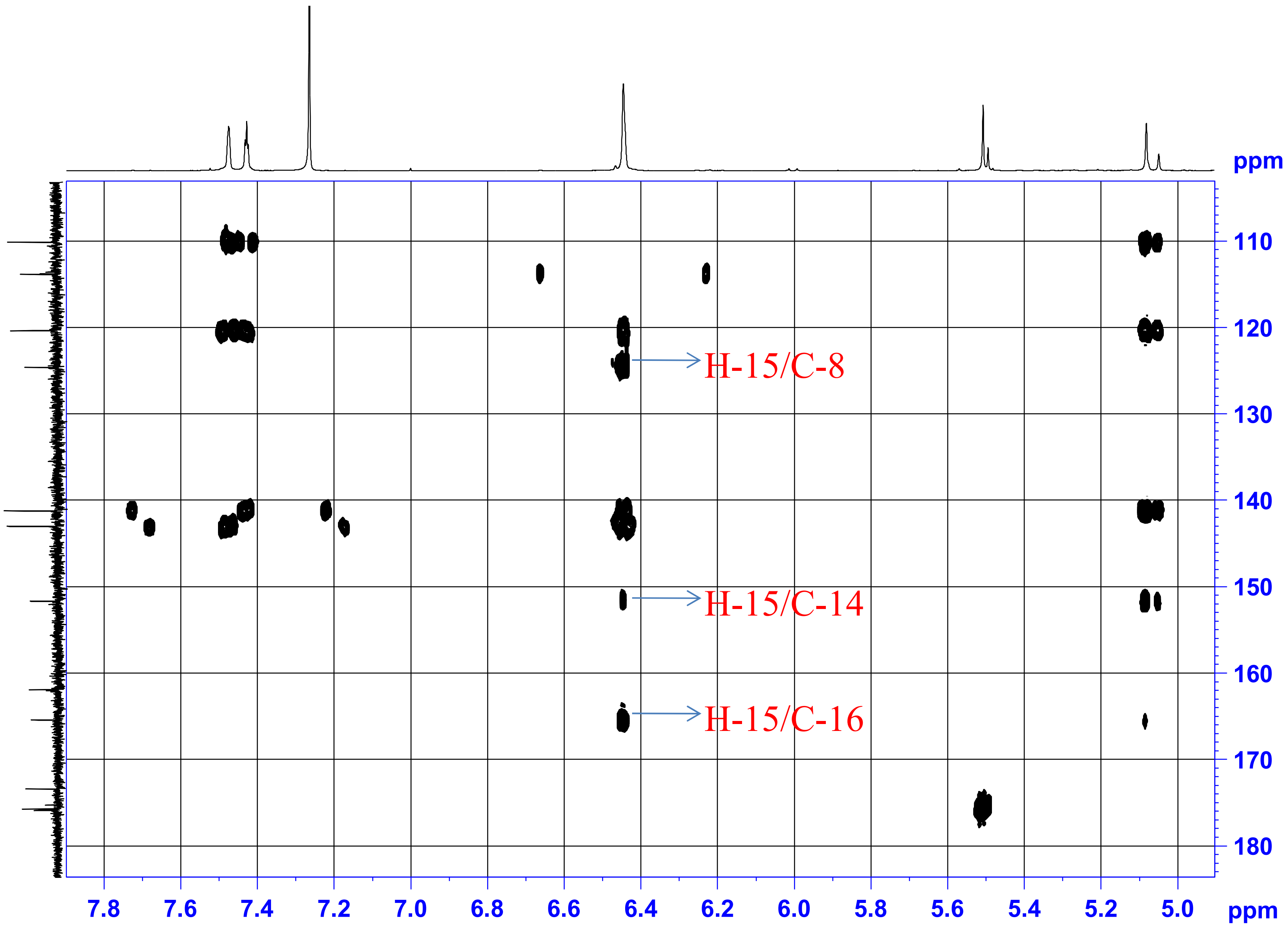

HMBC spectrum of Krishnolide B (**2**) in $CDCl_3$

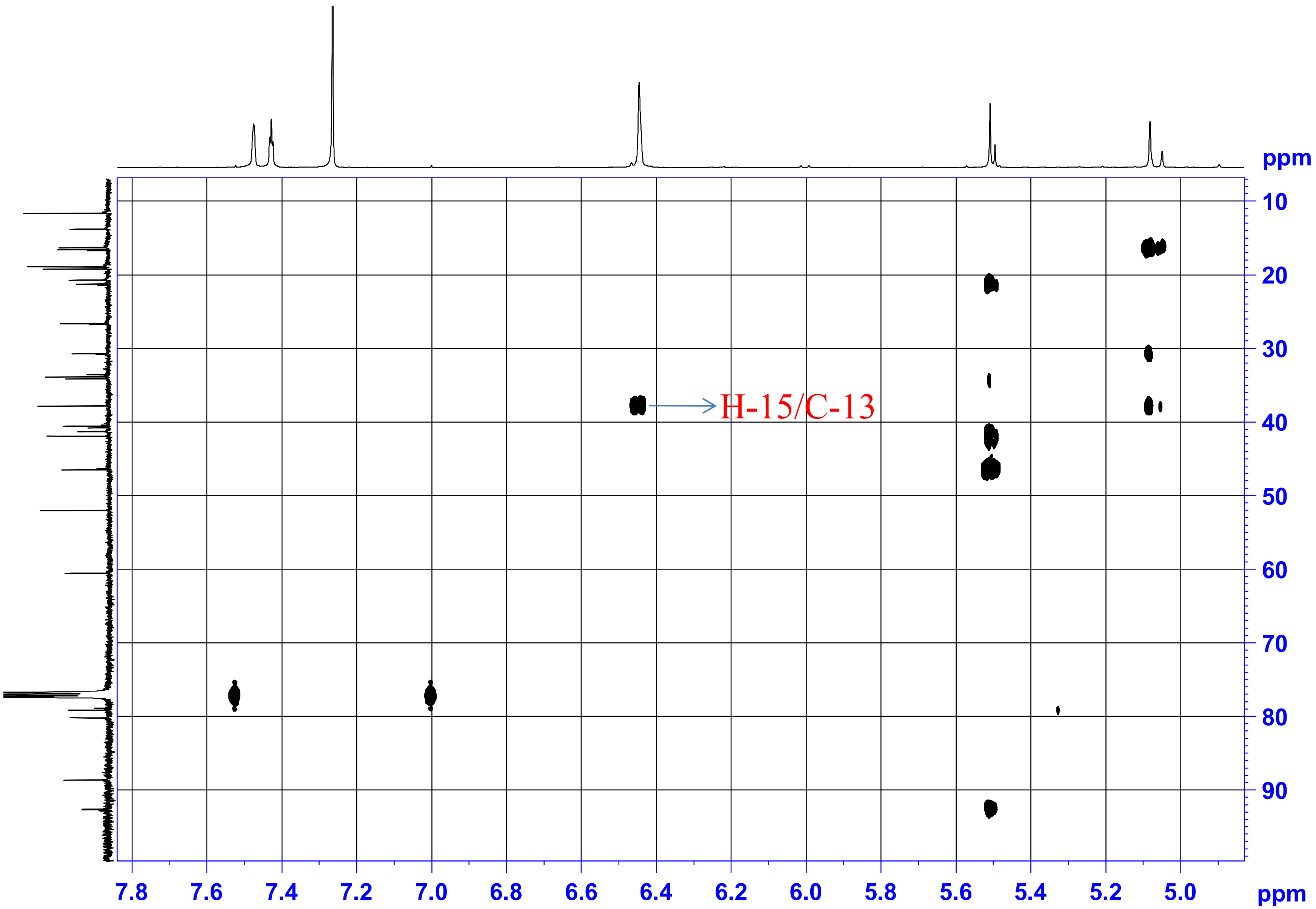

HMBC spectrum of Krishnolide B (**2**) in $CDCl_3$

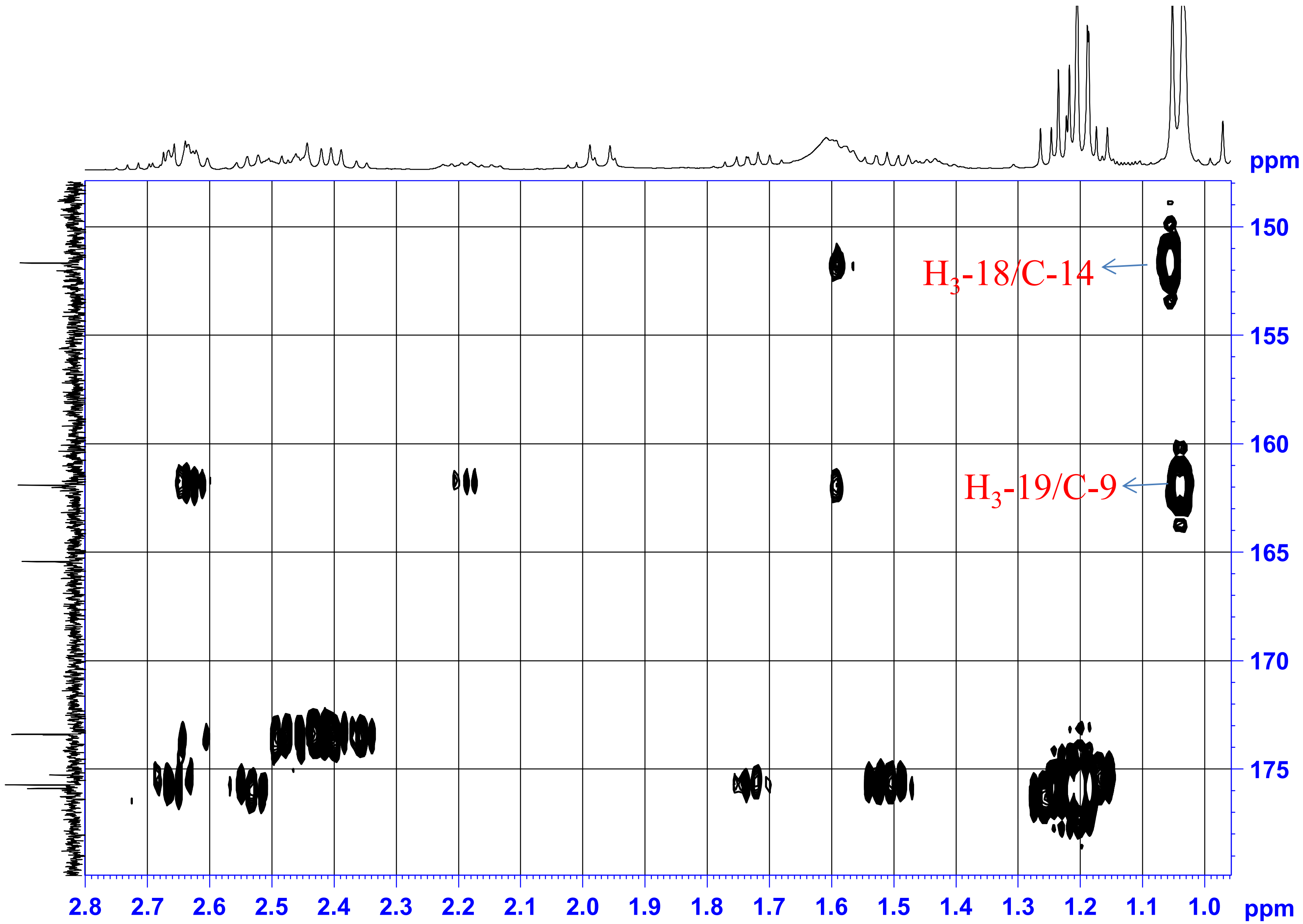

HMBC spectrum of Krishnolide B (**2**) in $CDCl_3$

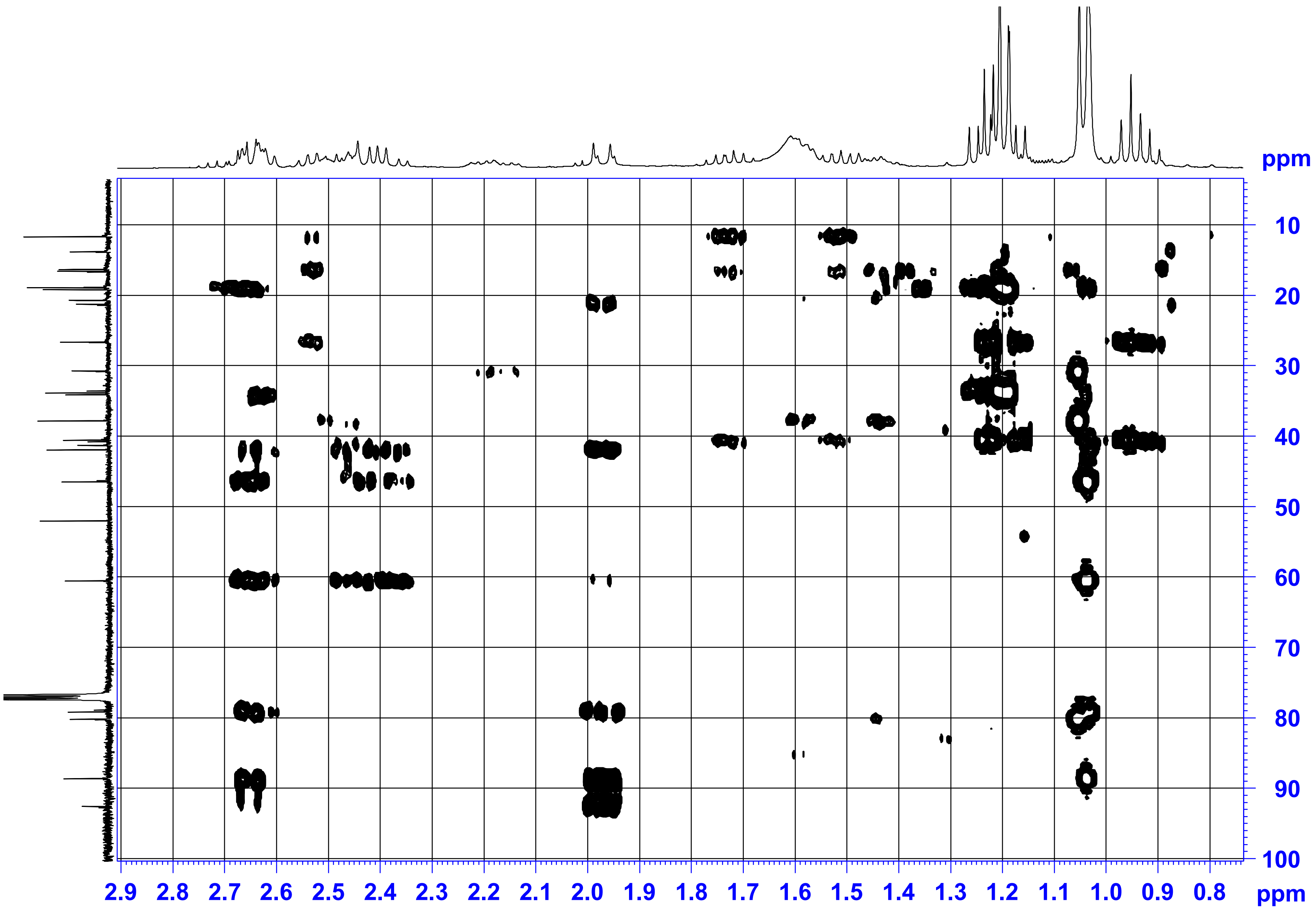

HMBC spectrum of Krishnolide B (**2**) in $CDCl_3$

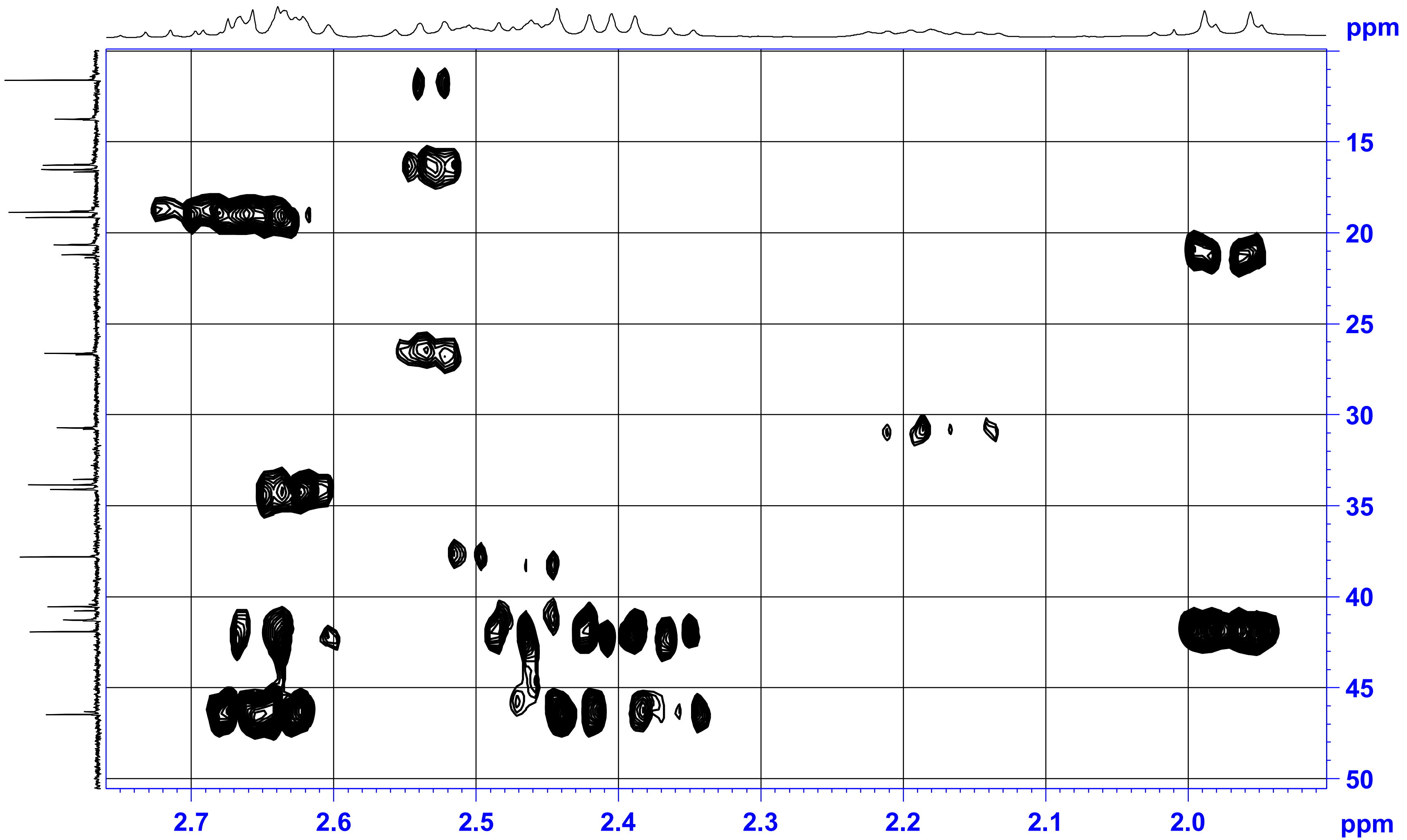

HMBC spectrum of Krishnolide B (**2**) in $CDCl_3$

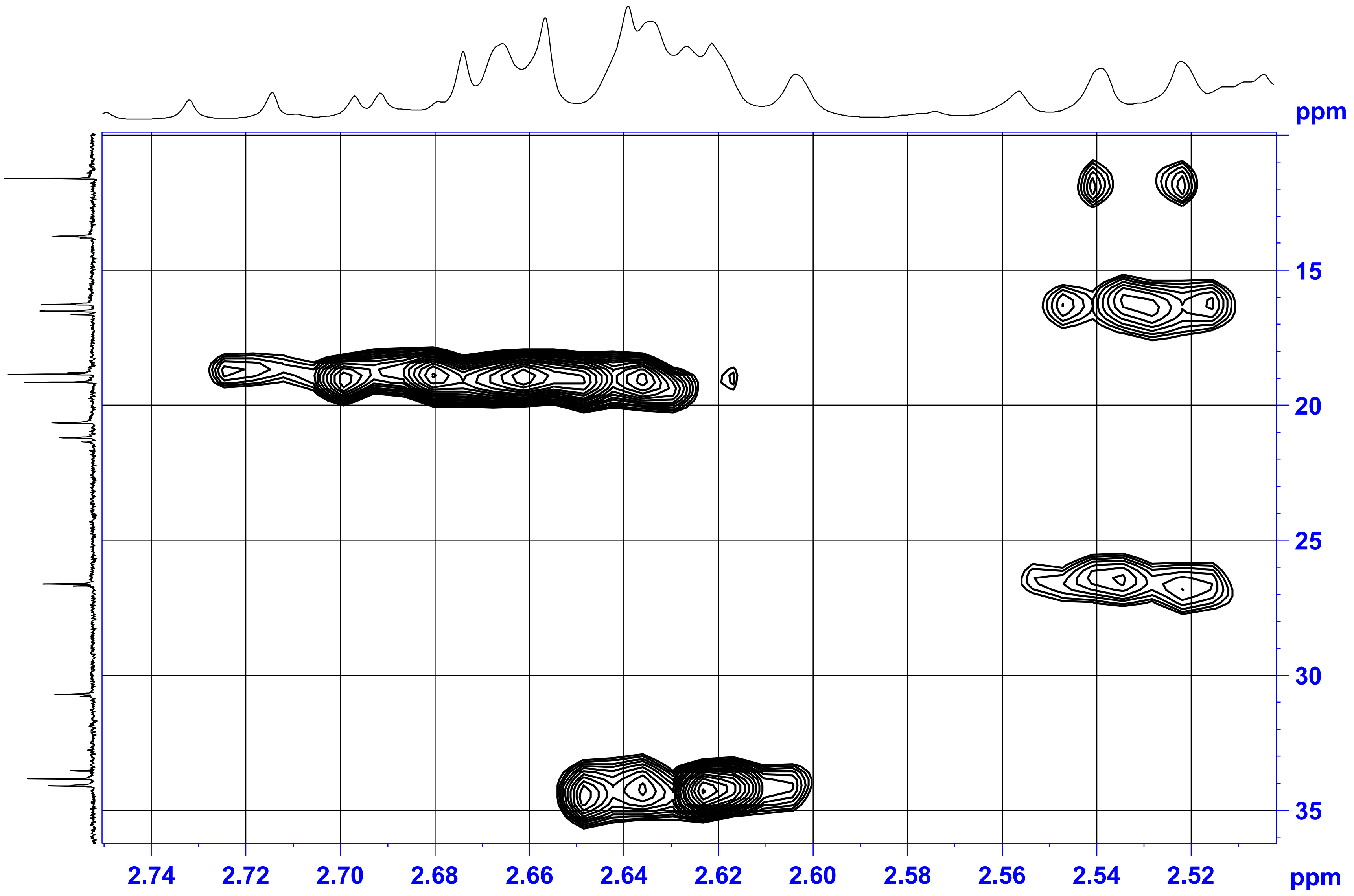

HMBC spectrum of Krishnolide B (**2**) in $CDCl_3$

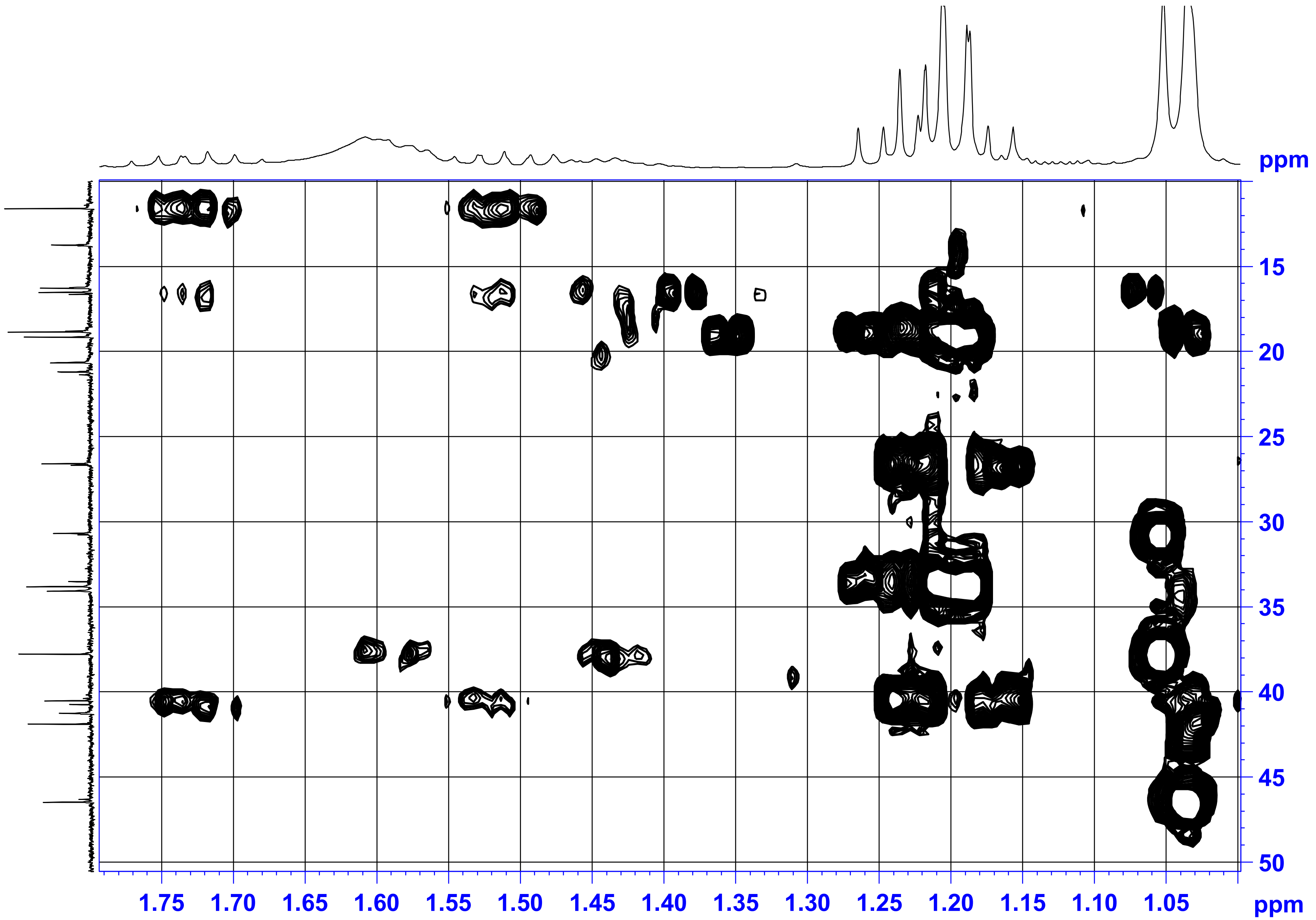

HMBC spectrum of Krishnolide B (**2**) in $CDCl_3$

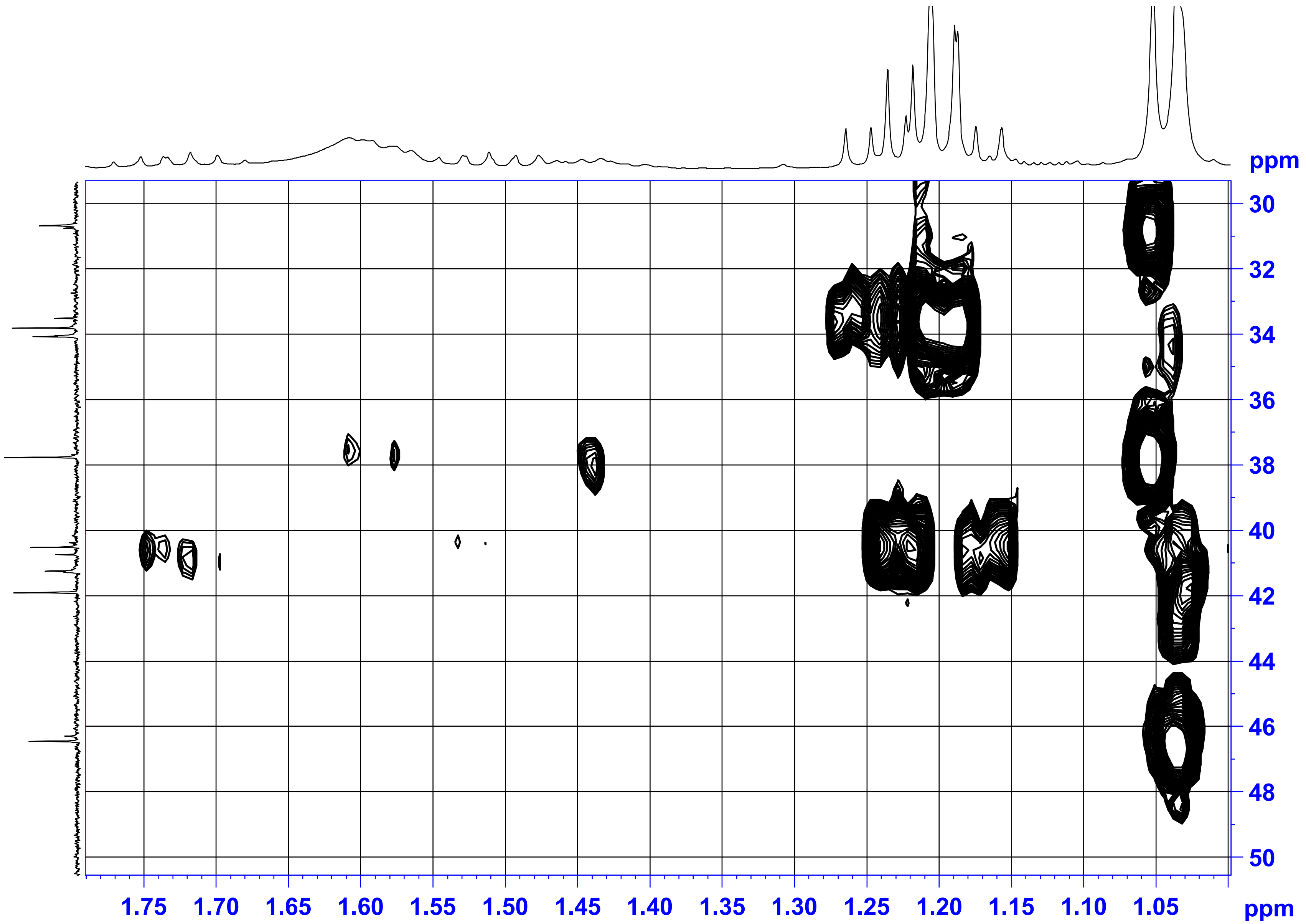

HMBC spectrum of Krishnolide B (**2**) in $CDCl_3$

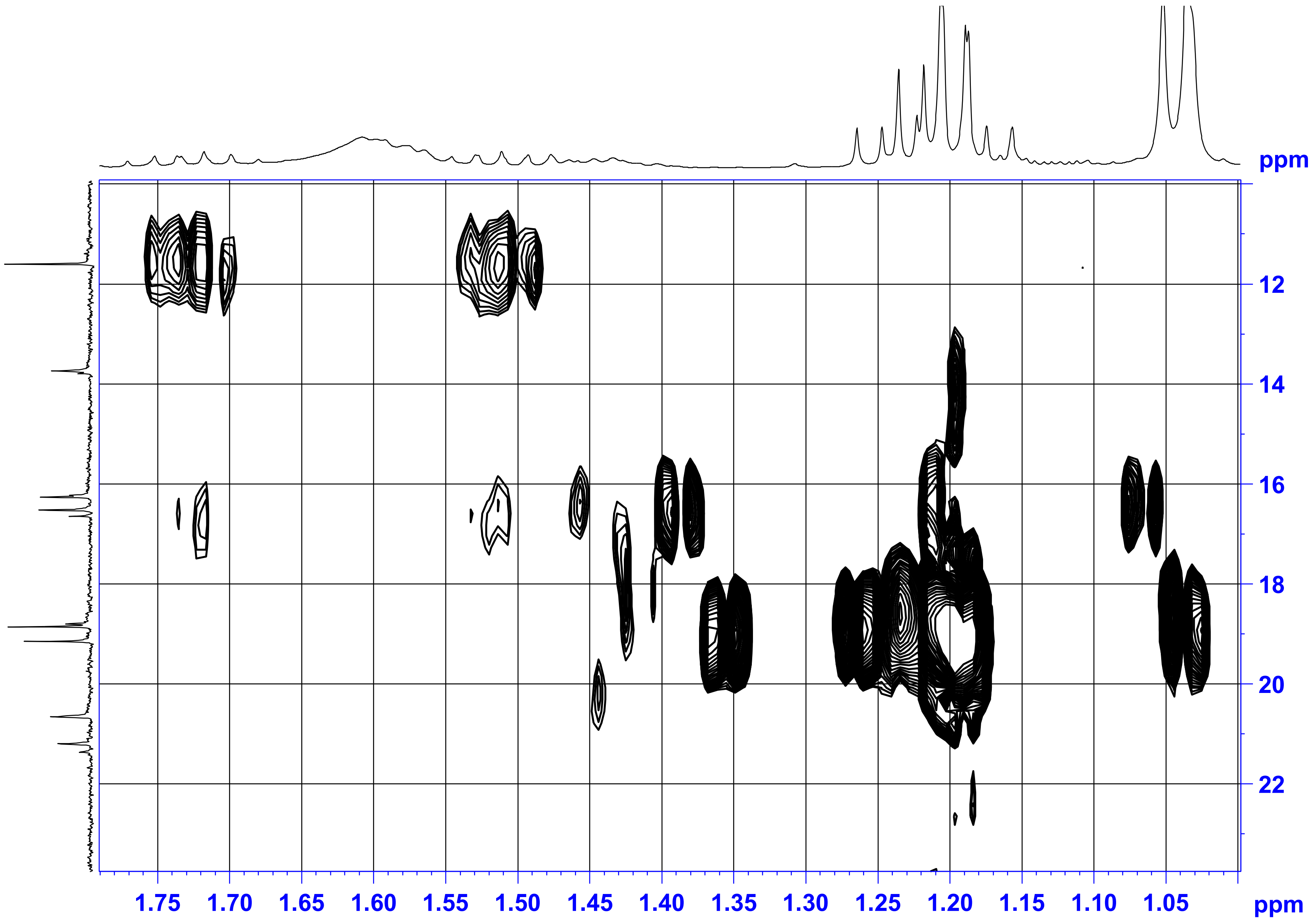

NOESY spectrum of Krishnolide B (**2**) in $CDCl_3$

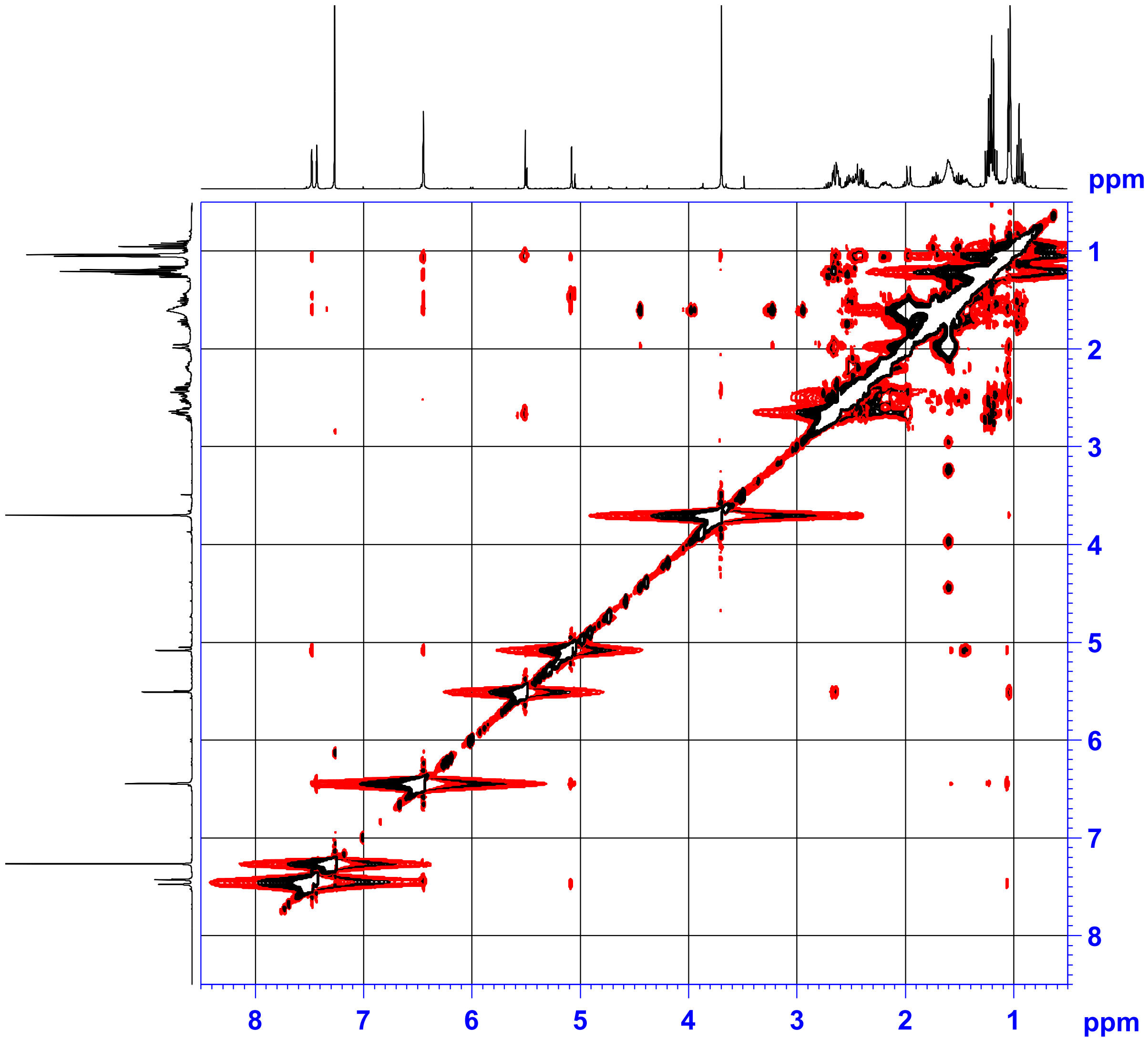

BRUKER

NAME	ZQ-19	
EXPNO	7	
PROCNO	1	
Date_	20170121	
Time	20.16	
INSTRUM	spect	
PROBHD	5 mm CPPBBO BB	
PULPROG	noesygpphpp	
TD	2048	
SOLVENT	CDCl3	
NS	16	
DS	32	
SWH	4000.000	Hz
FIDRES	1.953125	Hz
AQ	0.2560500	sec
RG	208.5	
DW	125.000	usec
DE	10.00	usec
TE	297.0	K
D0	0.00011036	sec
D1	1.99385595	sec
D8	0.30000001	sec
D11	0.03000000	sec
D12	0.00002000	sec
D16	0.00020000	sec
IN0	0.00025000	sec
======== CHANNEL f1 ========		
SFO1	400.1318006	MHz
NUC1	1H	
P1	11.50	usec
P2	23.00	usec
P17	2500.00	usec
ND0	1	
TD	256	
SFO1	400.1318	MHz
FIDRES	15.625000	Hz
SW	9.997	ppm
FnMODE	States-TPPI	
SI	1024	
SF	400.1300061	MHz
WDW	QSINE	
SSB	2	
LB	0.00	Hz
GB	0	
PC	1.00	
SI	1024	
MC2	States-TPPI	
SF	400.1300061	MHz
WDW	QSINE	
SSB	2	
LB	0.00	Hz
GB	0	

NOESY spectrum of Krishnolide B (**2**) in $CDCl_3$

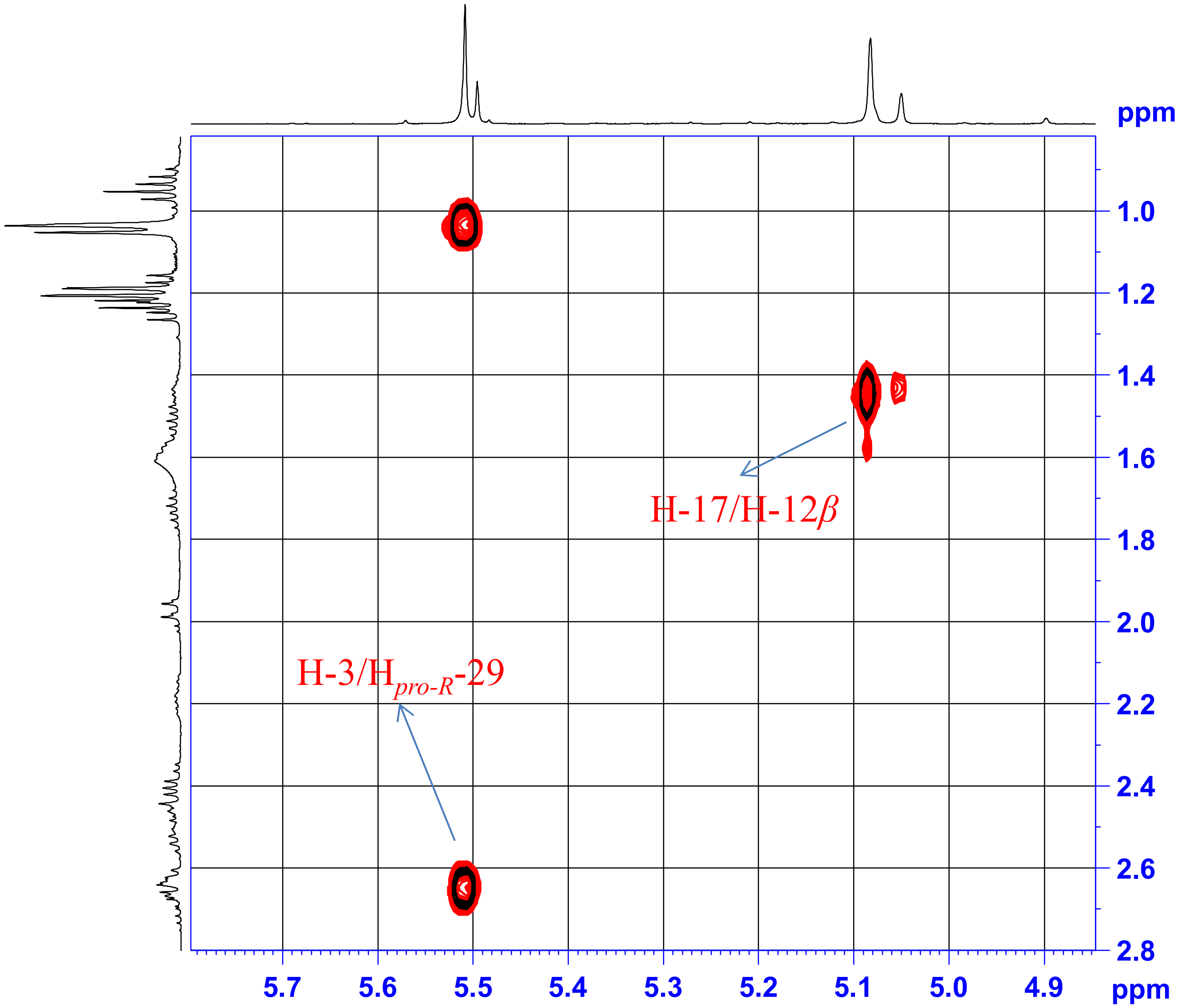

NOESY spectrum of Krishnolide B (**2**) in $CDCl_3$

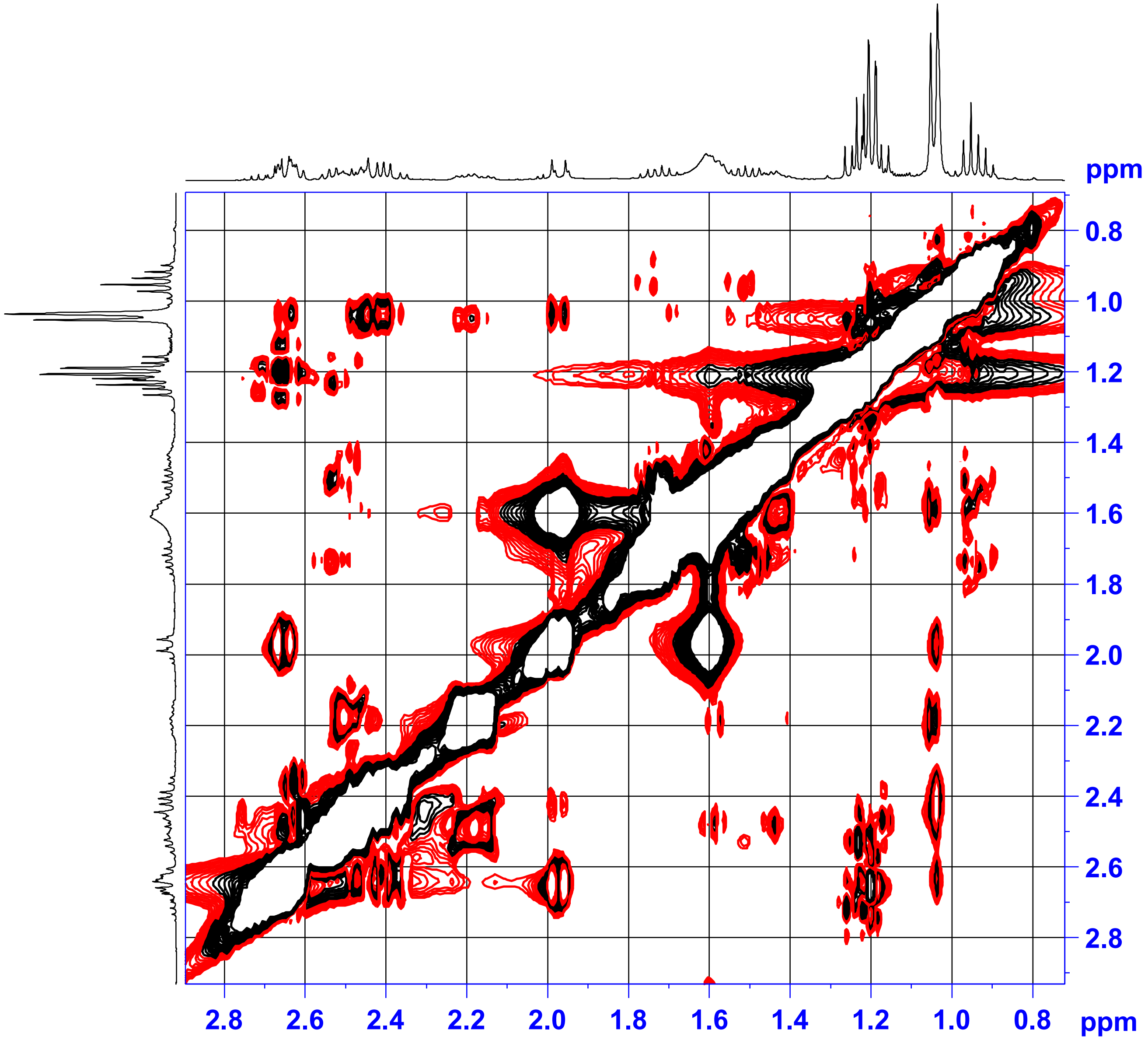

NOESY spectrum of Krishnolide B (**2**) in $CDCl_3$

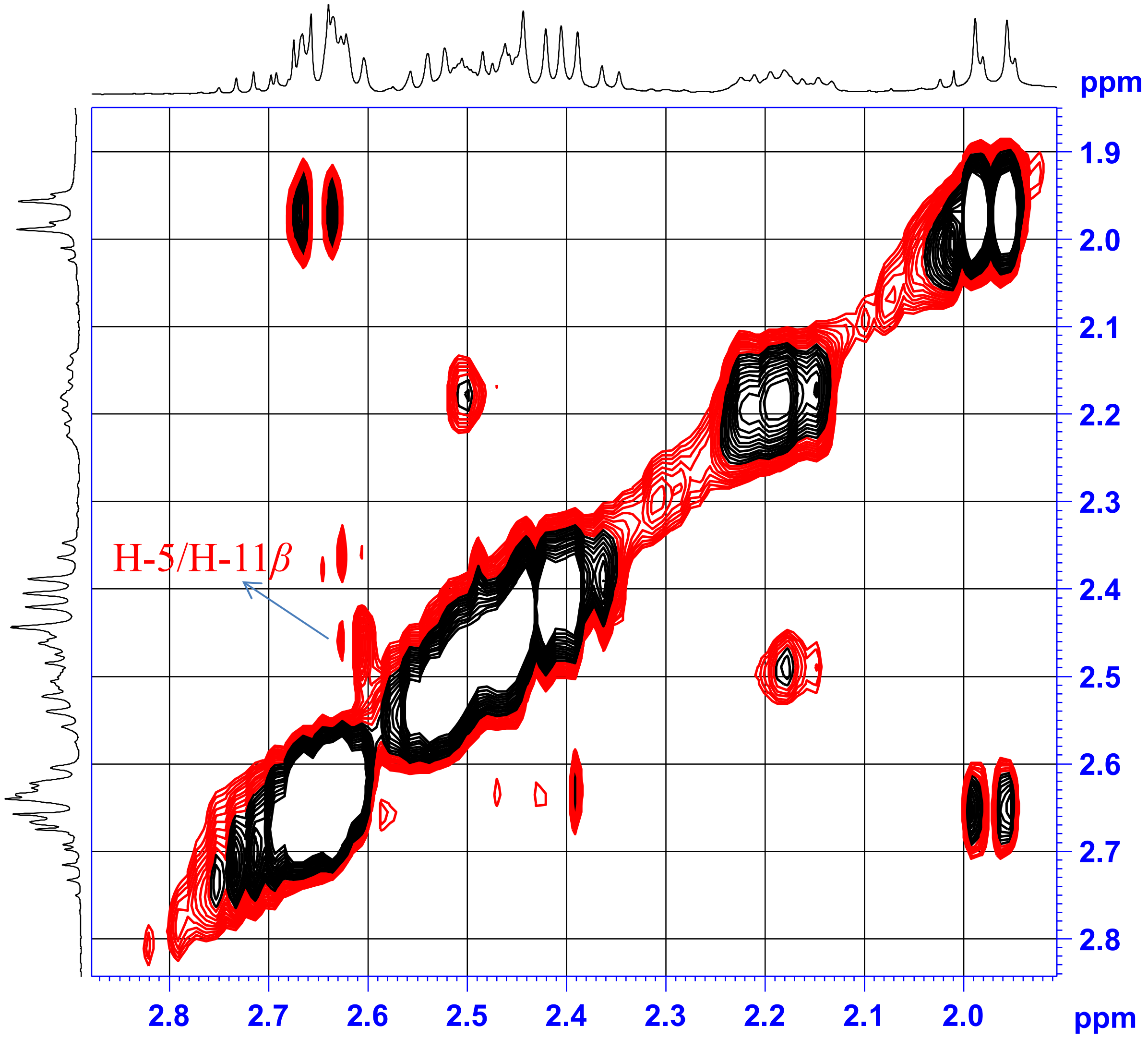

NOESY spectrum of Krishnolide B (**2**) in $CDCl_3$

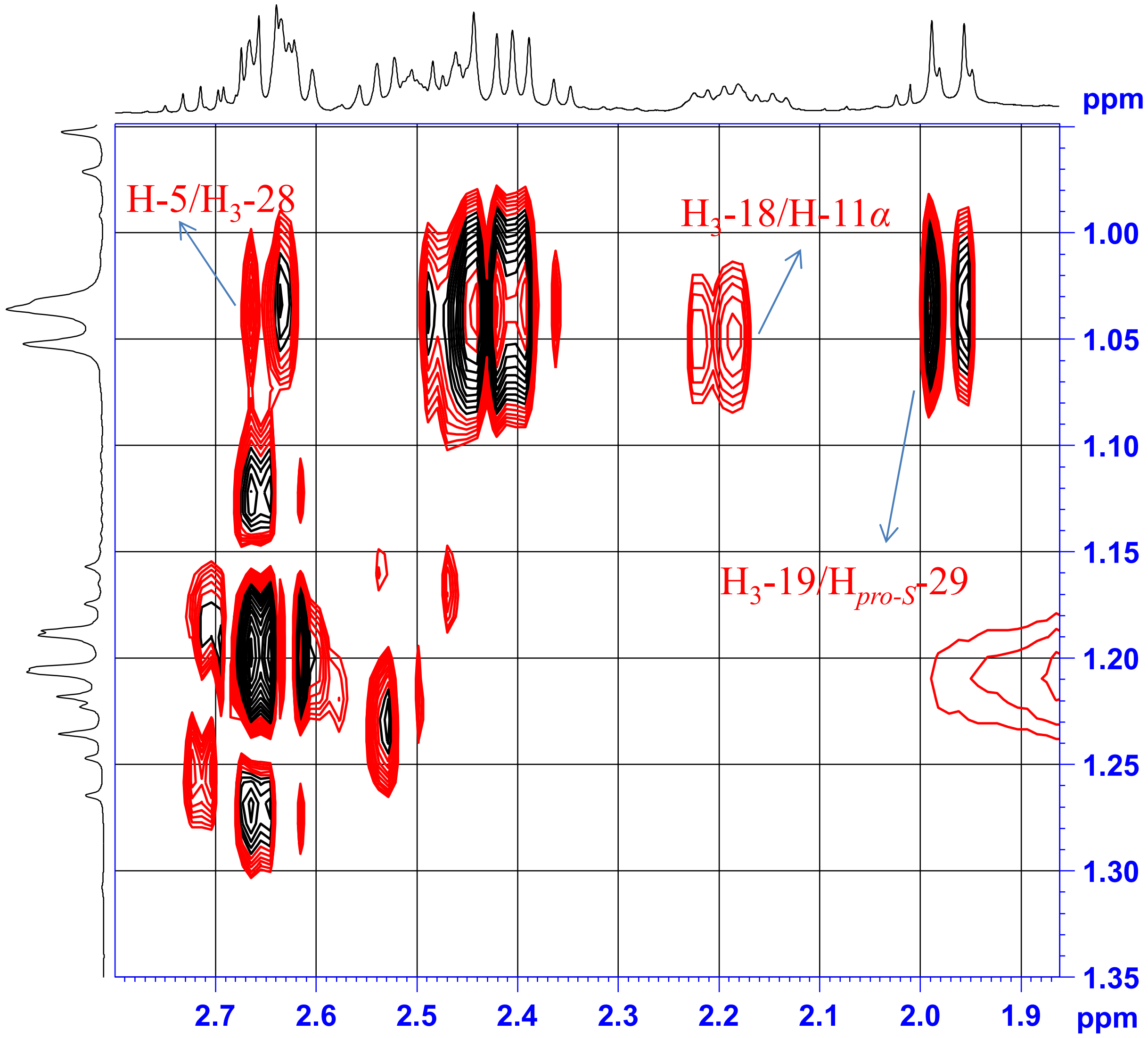

HR-ESIMS of Krishnolide C (**3**)

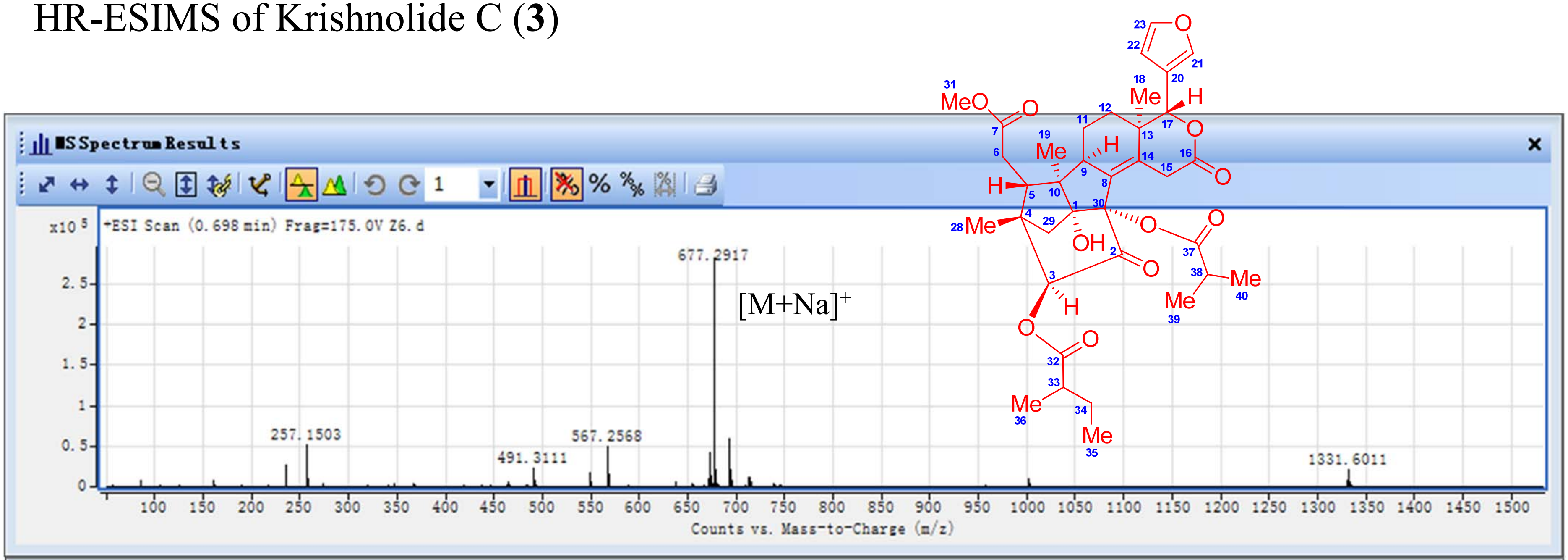

MS Formula Results: + Scan (0.698 min)

m/z	Ion	Formula	Abundance
677.2917	(M+Na)+	C36 H46 Na O11	281805.7

Best	Formula (M)	Ion Formula	Score	Cross Score	Calc m/z	Diff (ppm)	Mass Match	Abund Match	Spacing Match
☑	C36 H46 O11	C36 H46 Na O11	92.5		677.2932	2.34	93.28	86.08	98.64

Isotope	Abund Sum%	Calc Abund Sum%	m/z	Calc m/z	Diff (ppm)
1	70.12	65.89	677.2917	677.2932	2.22
2	23.63	26.28	678.2945	678.2966	3.15
3	5.34	6.59	679.2988	679.2995	1.05
4	0.91	1.24	680.3038	680.3022	-2.3

UV spectrum of Krishnolide C (**3**) in MeCN

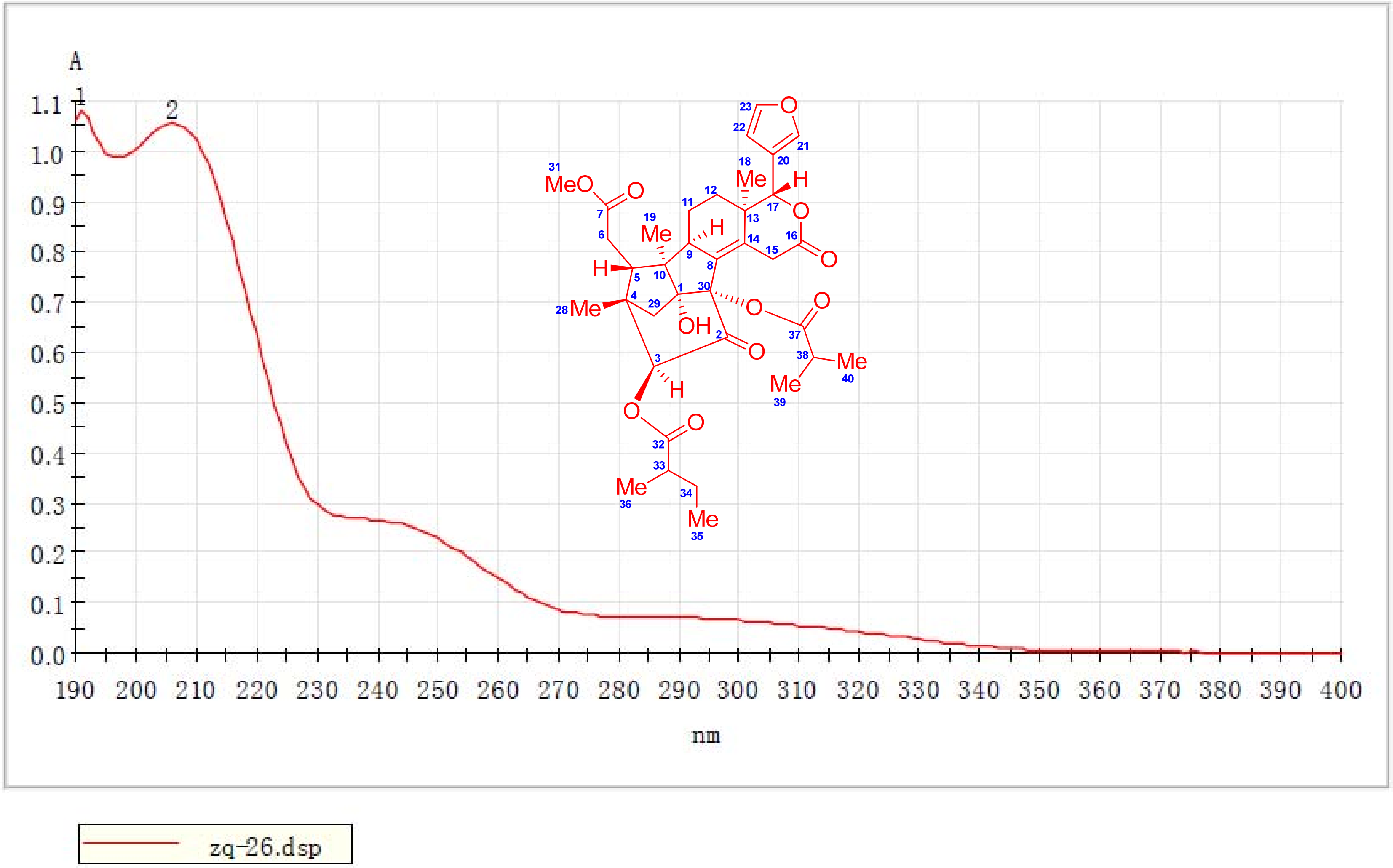

^1H NMR (400 MHz) spectrum of Krishnolide C (**3**) in $CDCl_3$

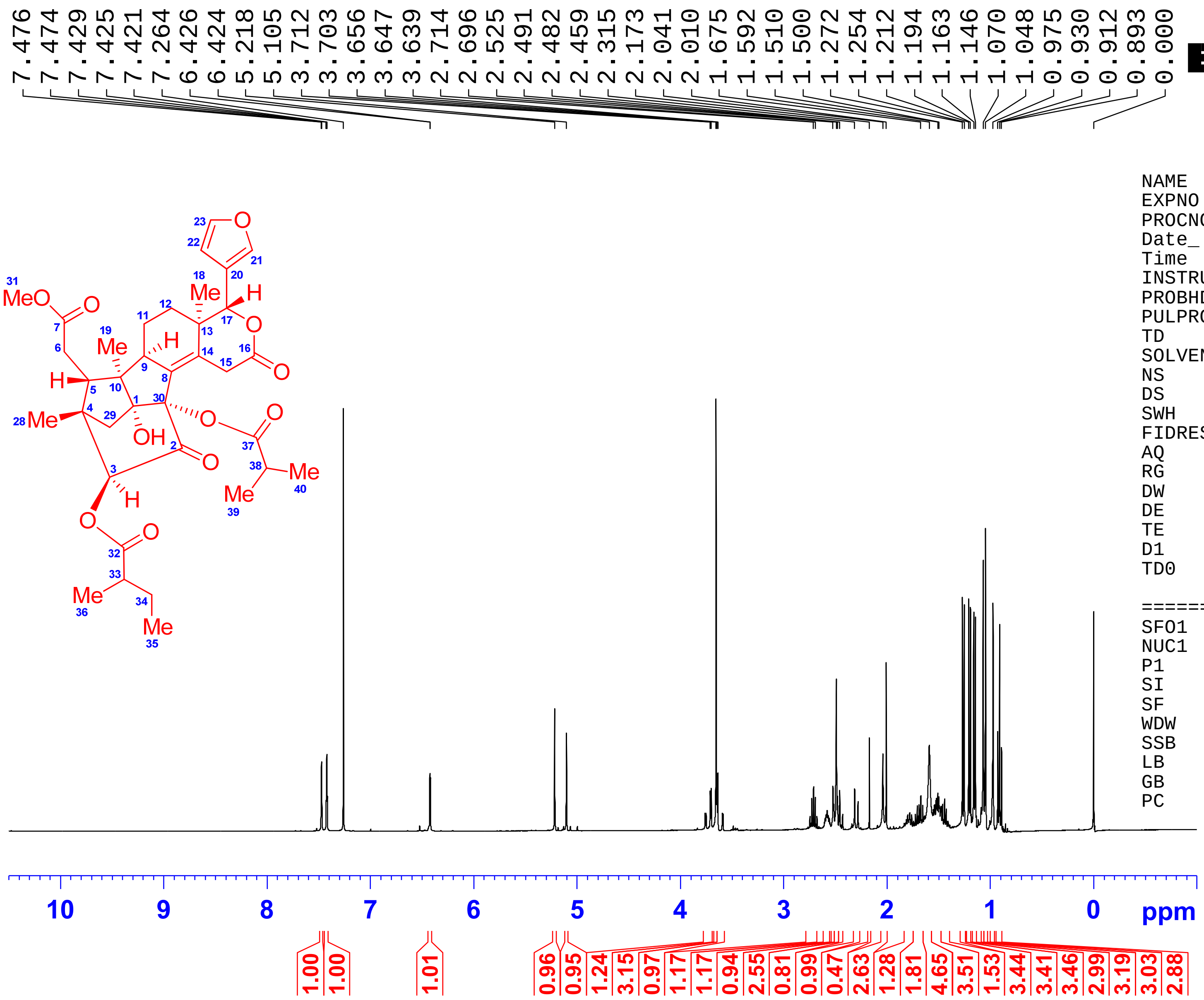

NAME	zq-26
EXPNO	1
PROCNO	1
Date_	20170629
Time	22.26
INSTRUM	spect
PROBHD	5 mm CPPBBO BB
PULPROG	zg30
TD	65536
SOLVENT	CDCl3
NS	16
DS	2
SWH	8223.685 Hz
FIDRES	0.125483 Hz
AQ	3.9846387 sec
RG	208.5
DW	60.800 usec
DE	10.00 usec
TE	297.0 K
D1	1.00000000 sec
TD0	1

======== CHANNEL f1 ========

SFO1	400.1324710 MHz
NUC1	1H
P1	11.50 usec
SI	65536
SF	400.1300083 MHz
WDW	EM
SSB	0
LB	0.30 Hz
GB	0
PC	1.00

^{1}H NMR (400 MHz) spectrum of Krishnolide C (**3**) in $CDCl_3$

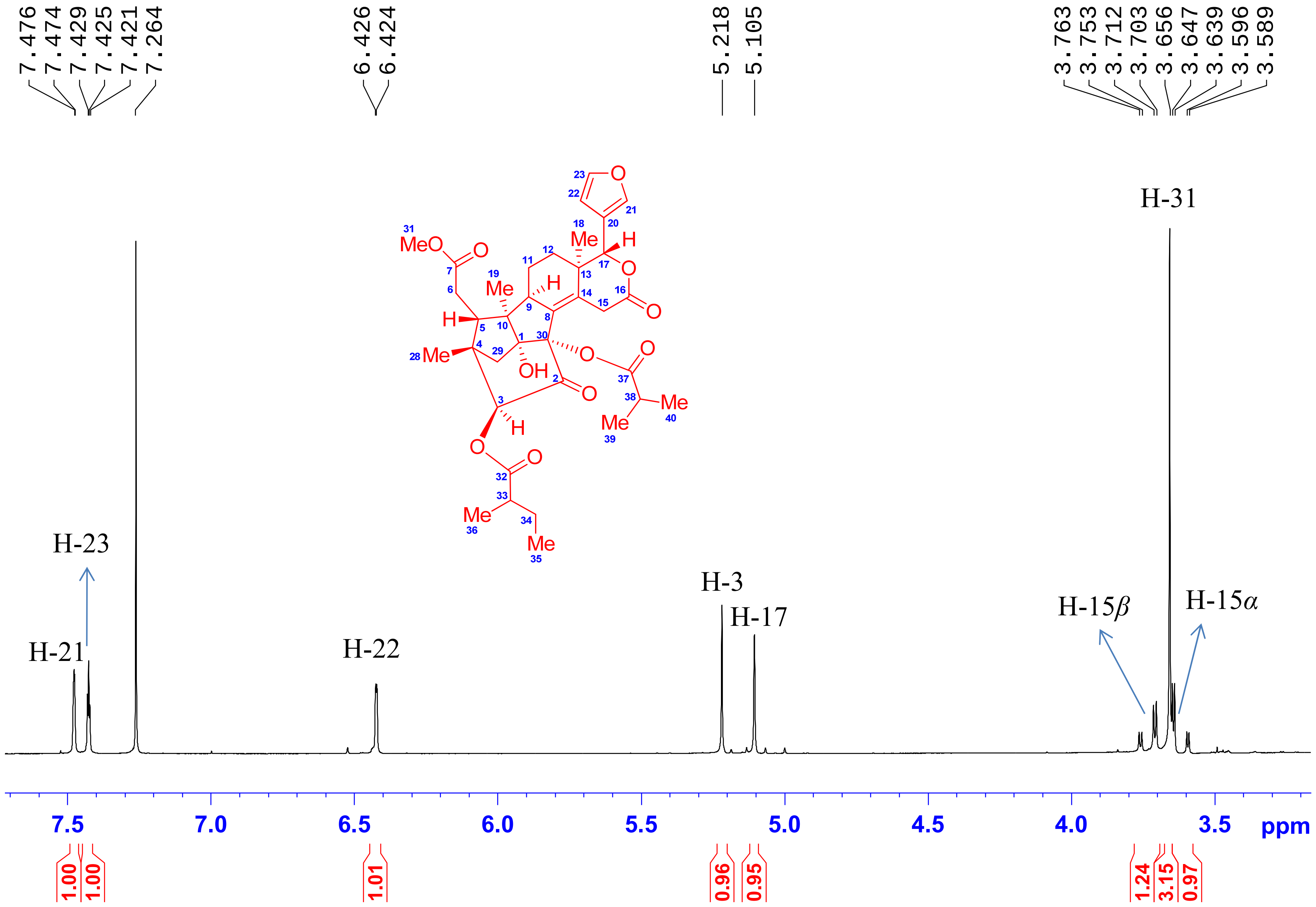

^{1}H NMR (400 MHz) spectrum of Krishnolide C (**3**) in $CDCl_3$

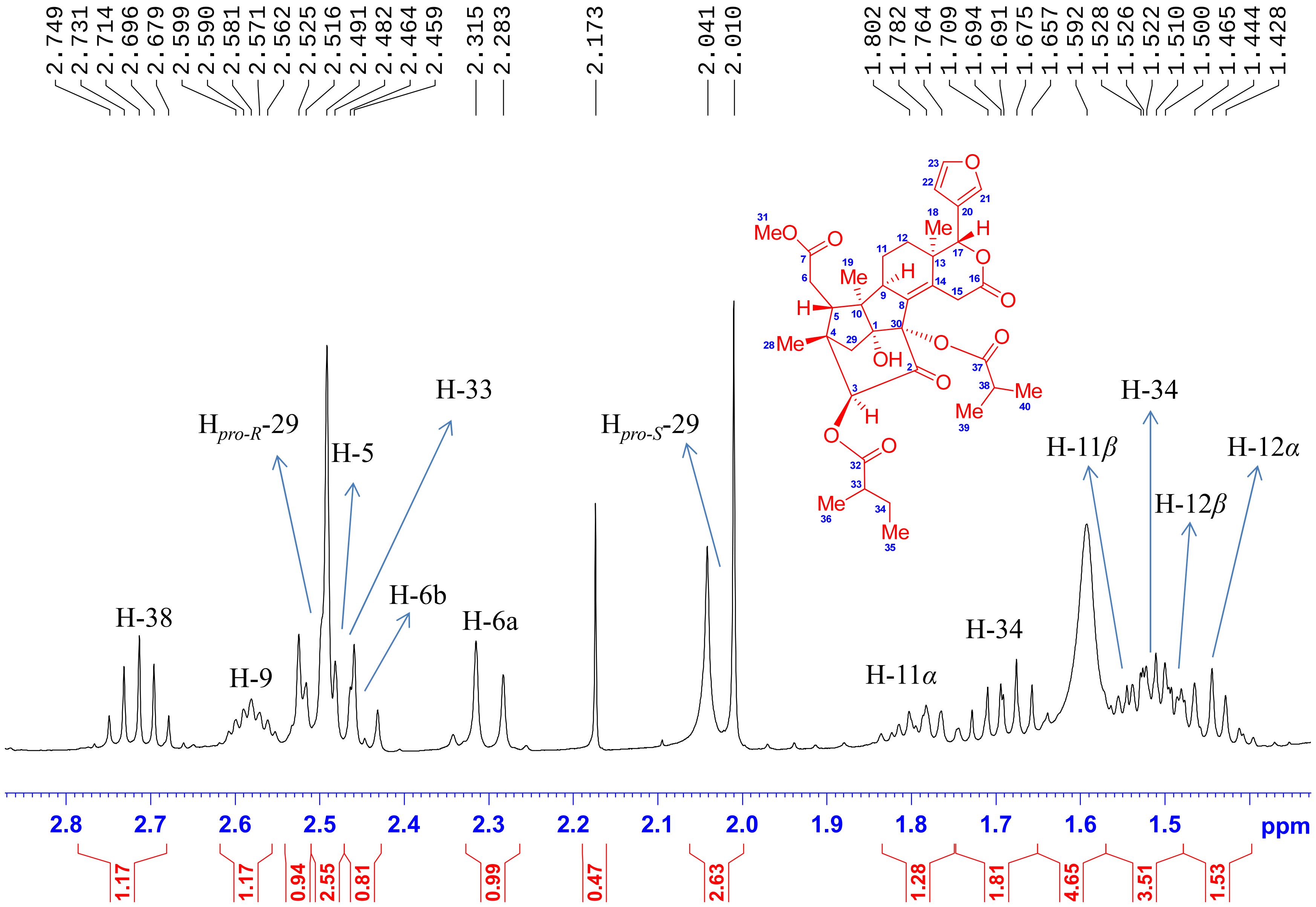

^{1}H NMR (400 MHz) spectrum of Krishnolide C (**3**) in $CDCl_3$

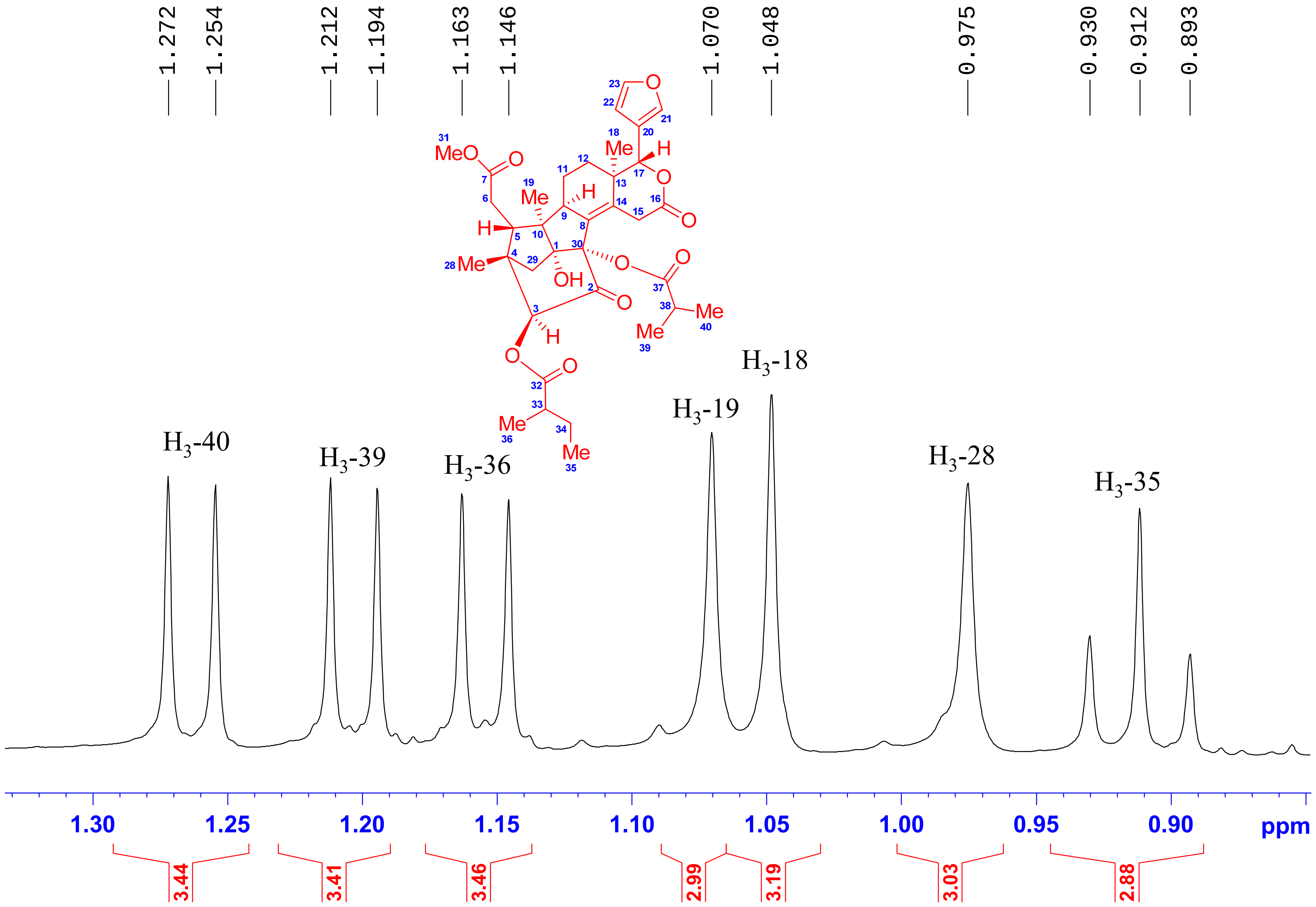

^{13}C NMR (100 MHz) spectrum of Krishnolide C (**3**) in $CDCl_3$

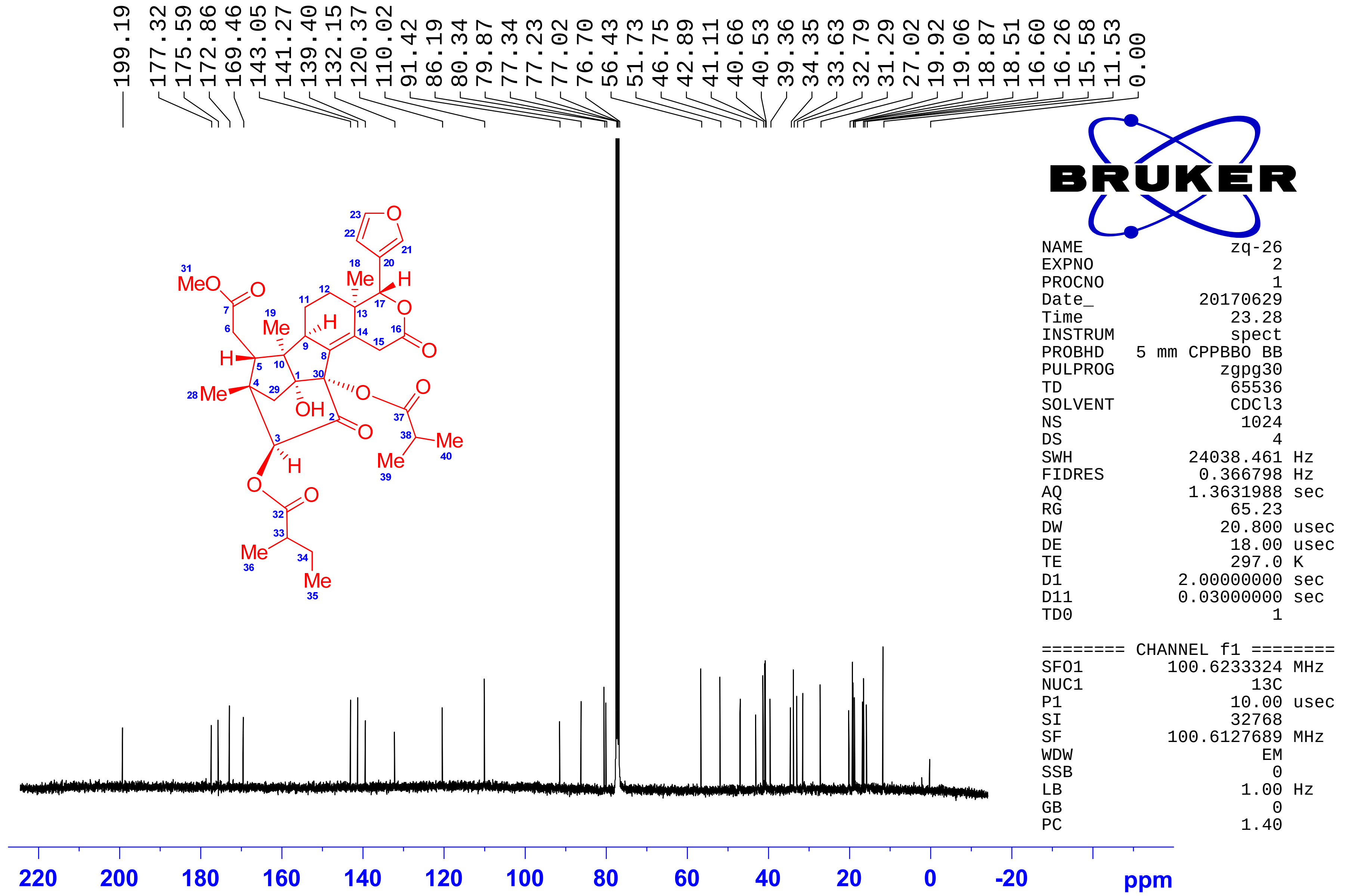

^{13}C NMR (100 MHz) spectrum of Krishnolide C (**3**) in $CDCl_3$

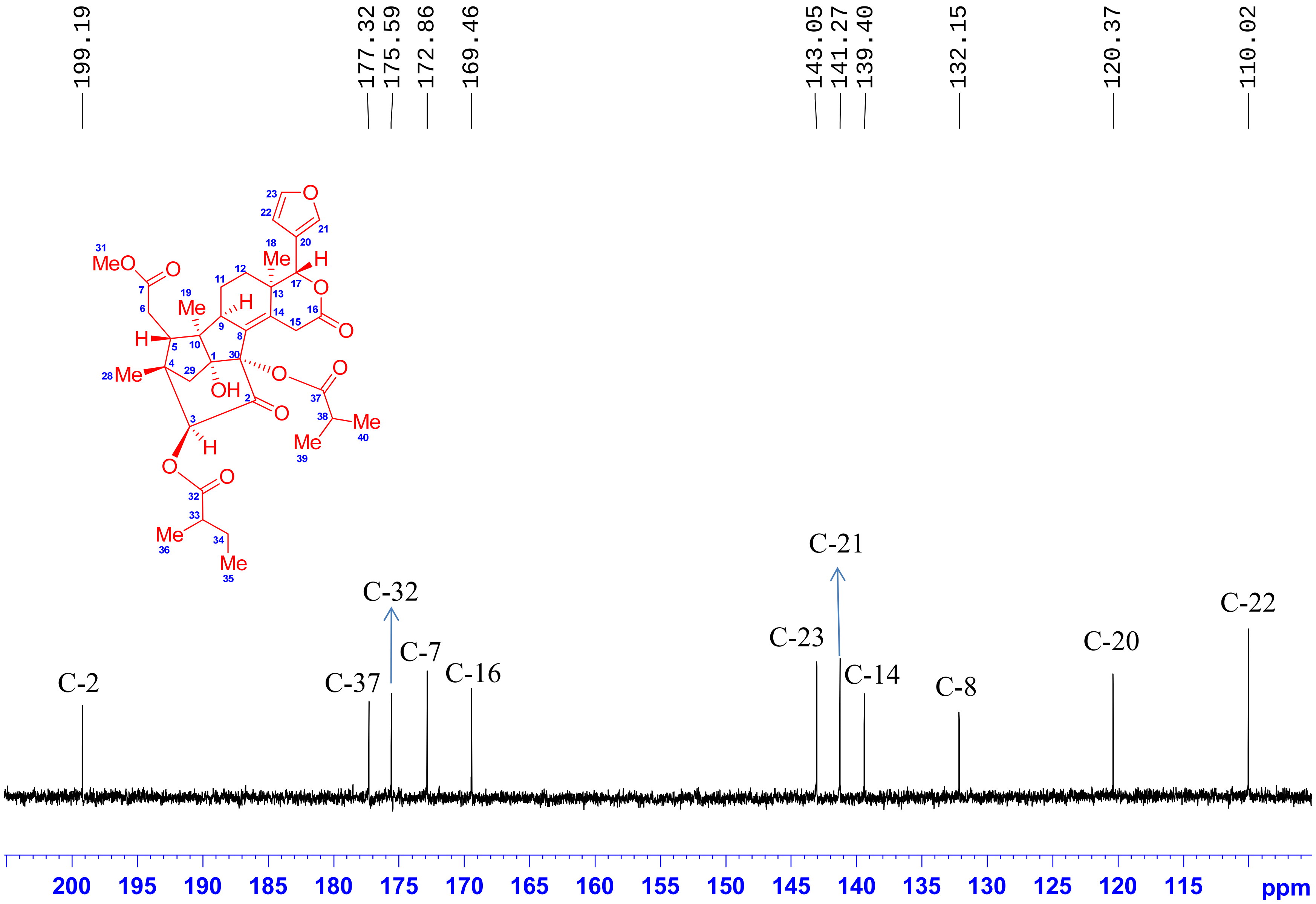

^{13}C NMR (100 MHz) spectrum of Krishnolide C (**3**) in $CDCl_3$

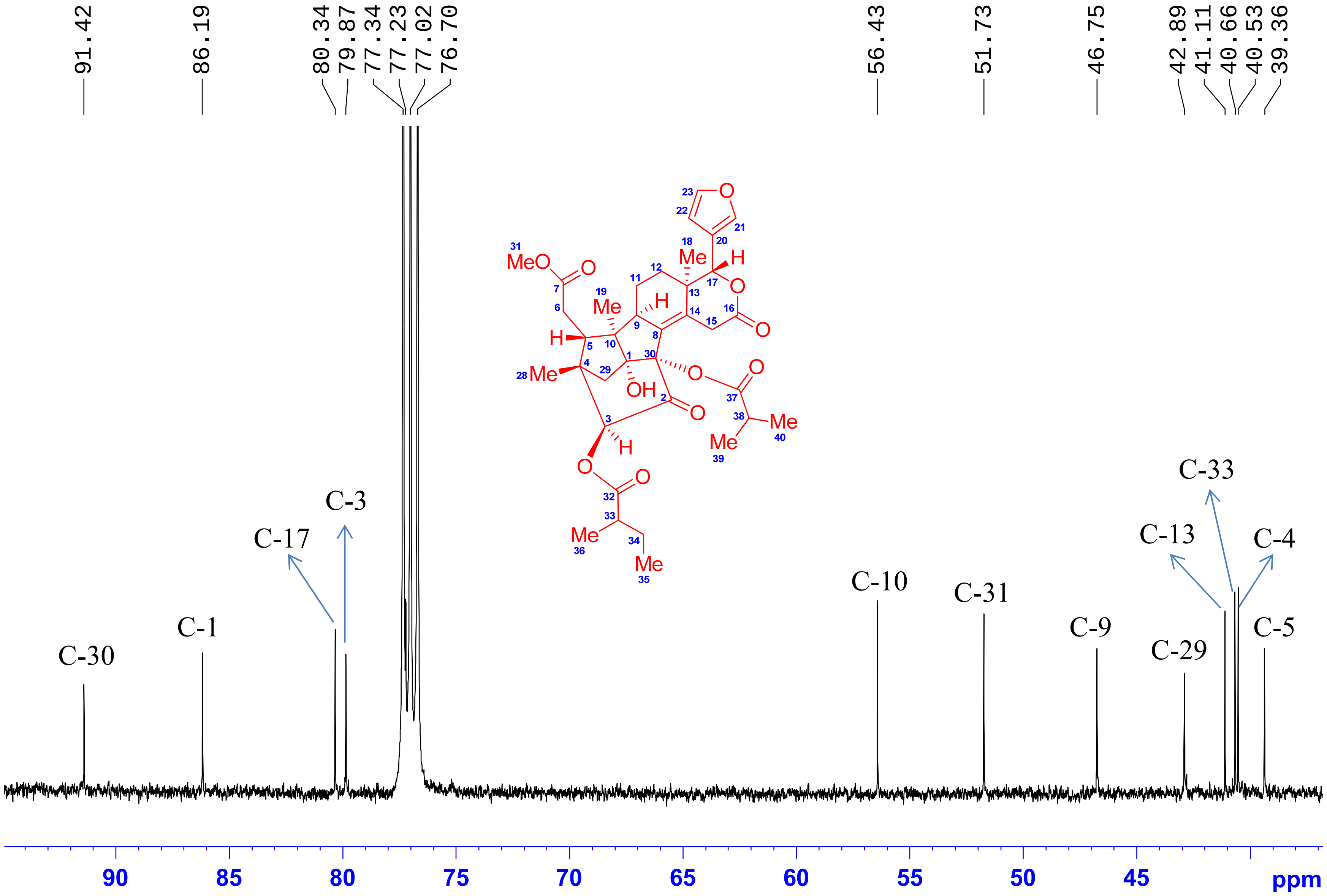

^{13}C NMR (100 MHz) spectrum of Krishnolide C (**3**) in $CDCl_3$

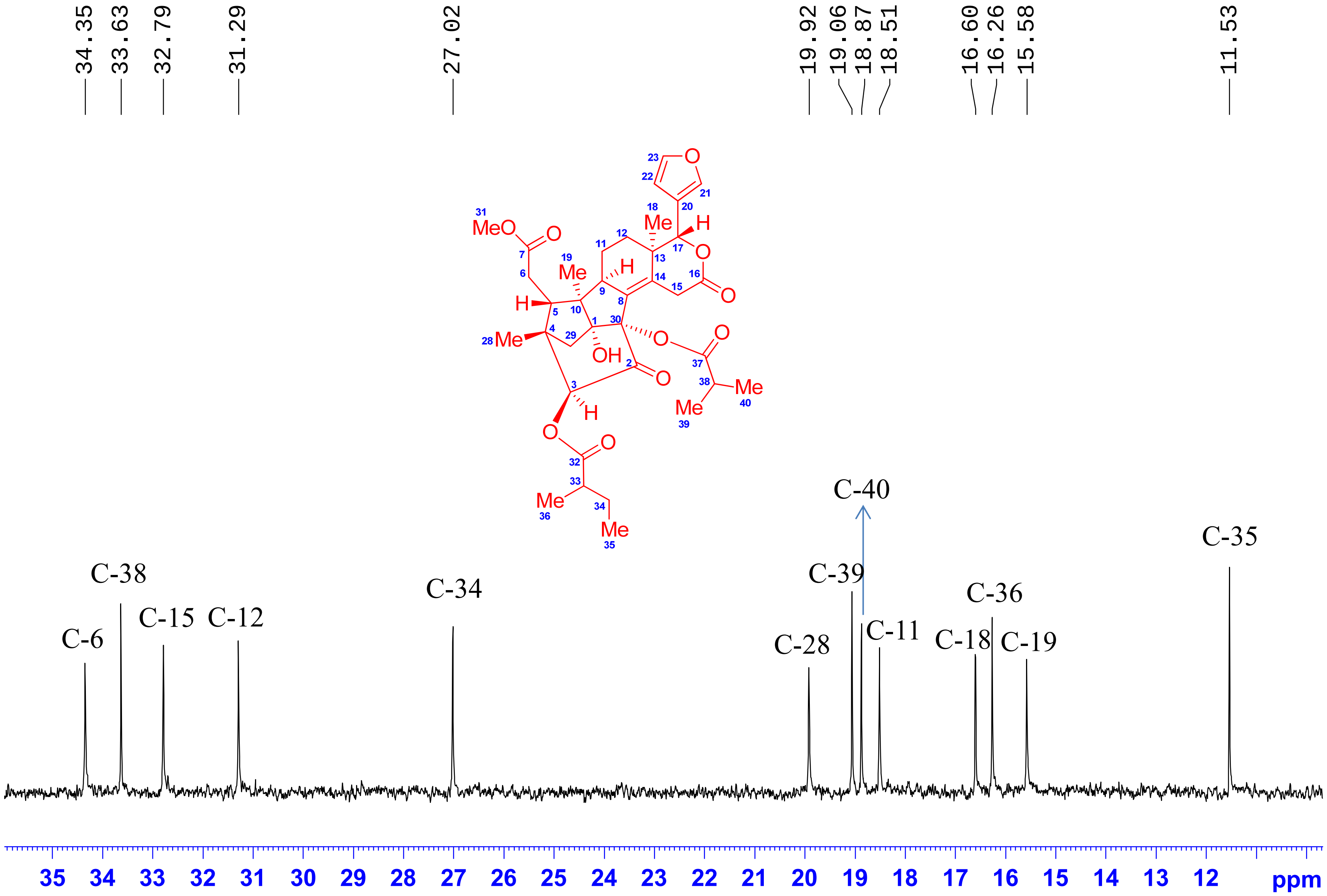

DEPT 135 spectrum of Krishnolide C (**3**) in $CDCl_3$

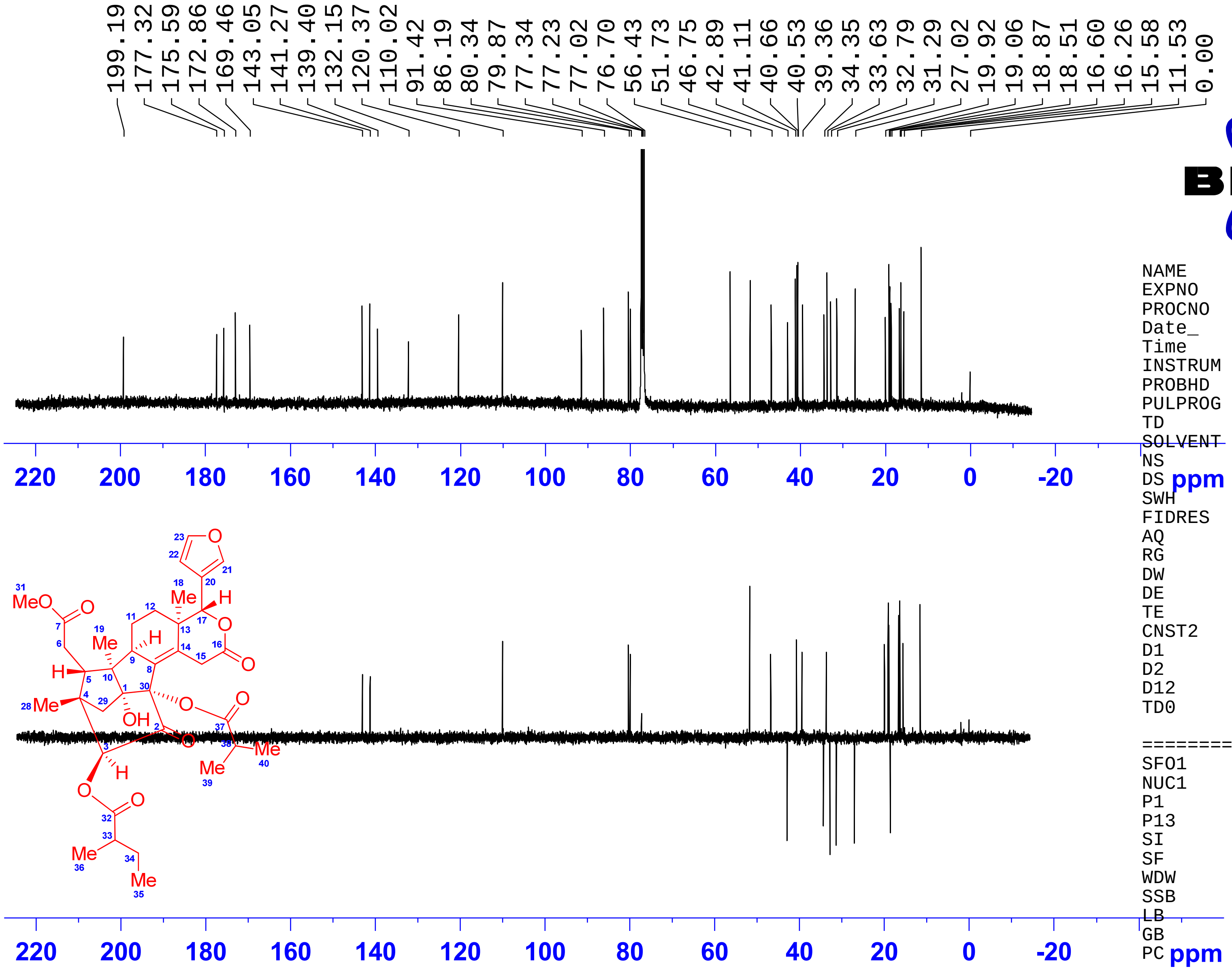


```
NAME                zq-26
EXPNO                   3
PROCNO                  1
Date_            20170629
Time                23.47
INSTRUM             spect
PROBHD   5 mm CPPBBO BB
PULPROG        deptsp135
TD                  65536
SOLVENT             CDCl3
NS                    300
DS                      4
SWH          24038.461 Hz
FIDRES        0.366798 Hz
AQ           1.3631988 sec
RG                 130.26
DW             20.800 usec
DE              18.00 usec
TE               297.0 K
CNST2         145.0000000
D1         2.00000000 sec
D2         0.00344828 sec
D12        0.00002000 sec
TD0                     1

======== CHANNEL f1 ========
SFO1       100.6233324 MHz
NUC1                  13C
P1              10.00 usec
P13           2000.00 usec
SI                  32768
SF         100.6127689 MHz
WDW                    EM
SSB                     0
LB                1.00 Hz
GB                      0
PC                   1.40
```

DEPT 135 spectrum of Krishnolide C (**3**) in $CDCl_3$

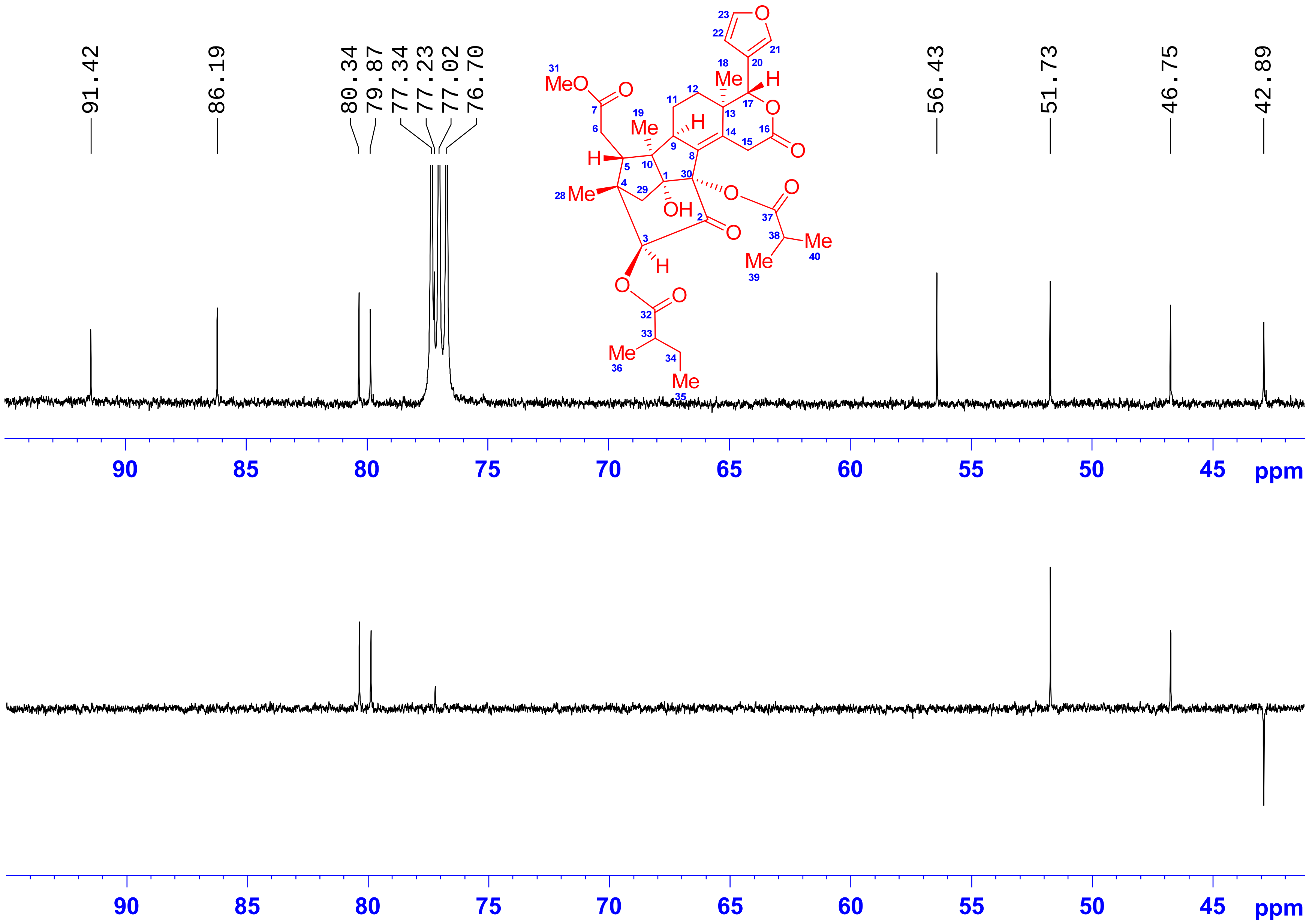

DEPT 135 spectrum of Krishnolide C (**3**) in $CDCl_3$

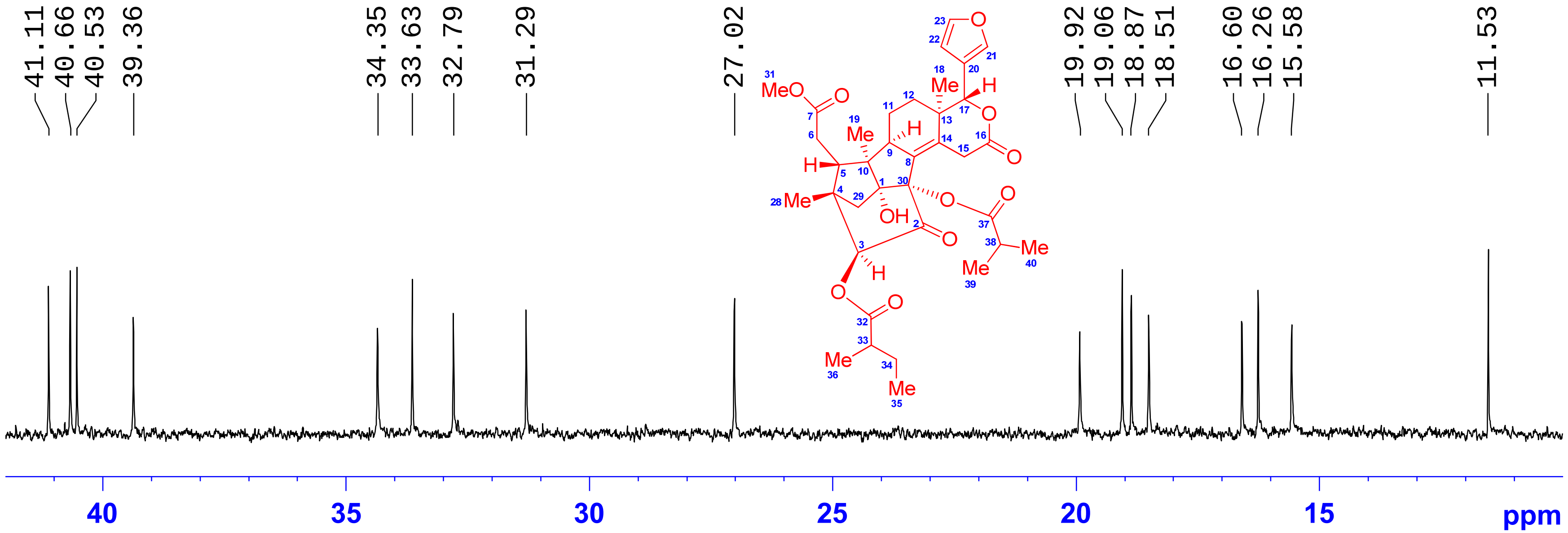

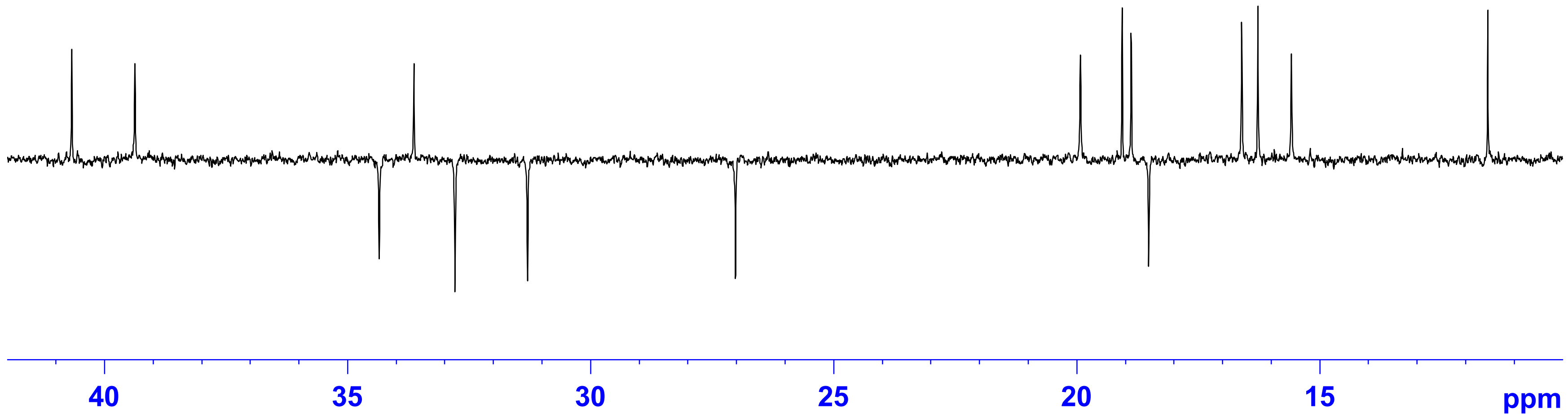

^{1}H-^{1}H COSY spectrum of Krishnolide C (**3**) in $CDCl_3$

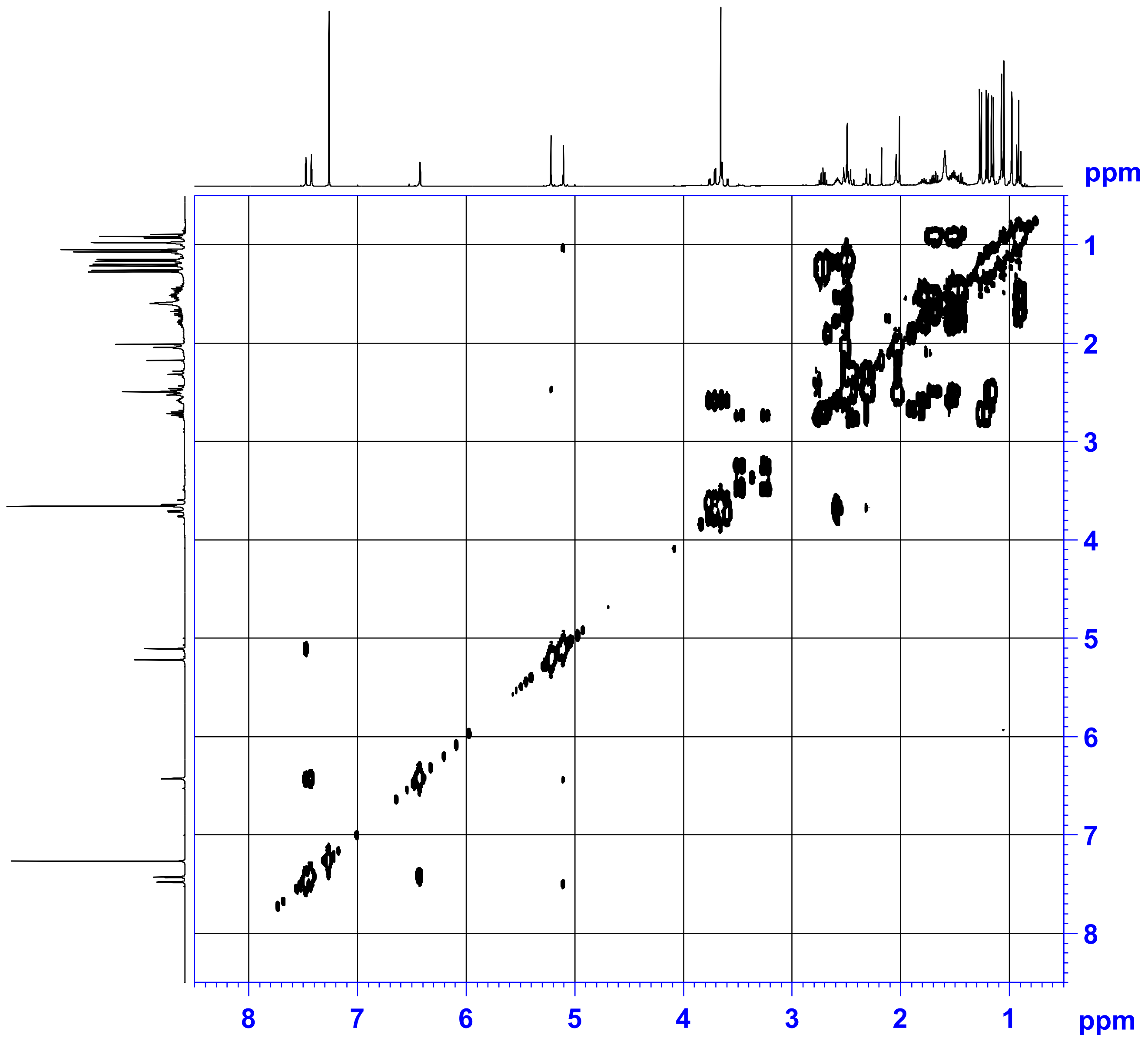

NAME	zq-26
EXPNO	4
PROCNO	1
Date_	20170707
Time	4.58
INSTRUM	spect
PROBHD	5 mm CPPBBO BB
PULPROG	cosygpppqf
TD	2048
SOLVENT	CDCl3
NS	8
DS	8
SWH	3906.250 Hz
FIDRES	1.907349 Hz
AQ	0.2621940 sec
RG	208.5
DW	128.000 usec
DE	10.00 usec
TE	297.0 K
D0	0.00000300 sec
D1	1.89678097 sec
D11	0.03000000 sec
D12	0.00002000 sec
D13	0.00000400 sec
D16	0.00020000 sec
IN0	0.00025600 sec

======== CHANNEL f1 ========

SFO1	400.1318006 MHz
NUC1	1H
P0	11.50 usec
P1	11.50 usec
P17	2500.00 usec
ND0	1
TD	128
SFO1	400.1318 MHz
FIDRES	30.517578 Hz
SW	9.762 ppm
FnMODE	QF
SI	1024
SF	400.1300063 MHz
WDW	QSINE
SSB	0
LB	0.00 Hz
GB	0
PC	1.40
SI	1024
MC2	QF
SF	400.1300063 MHz
WDW	QSINE
SSB	0
LB	0.00 Hz
GB	0

^1H-^1H COSY spectrum of Krishnolide C (**3**) in $CDCl_3$

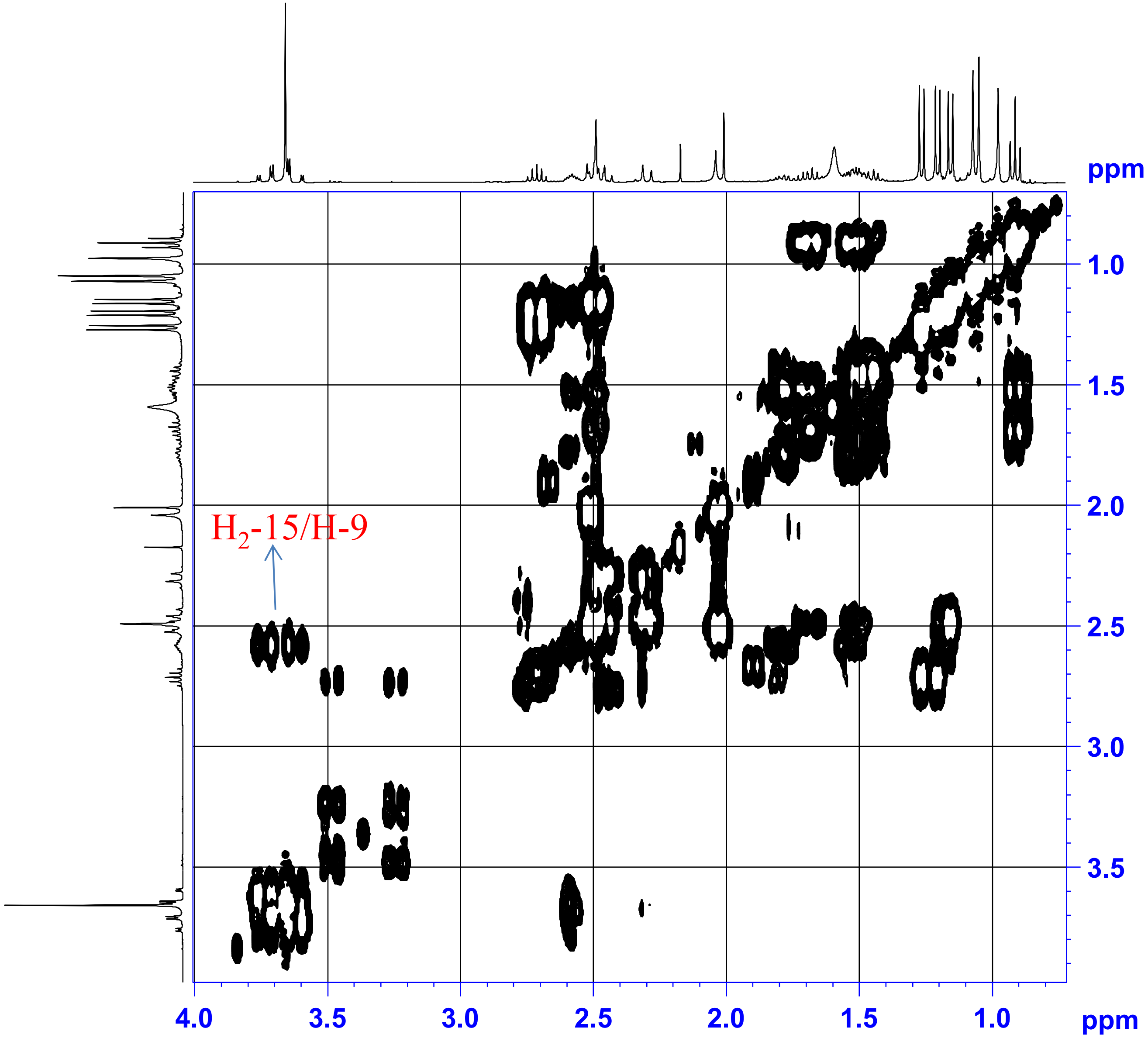

^{1}H-^{1}H COSY spectrum of Krishnolide C (**3**) in $CDCl_3$

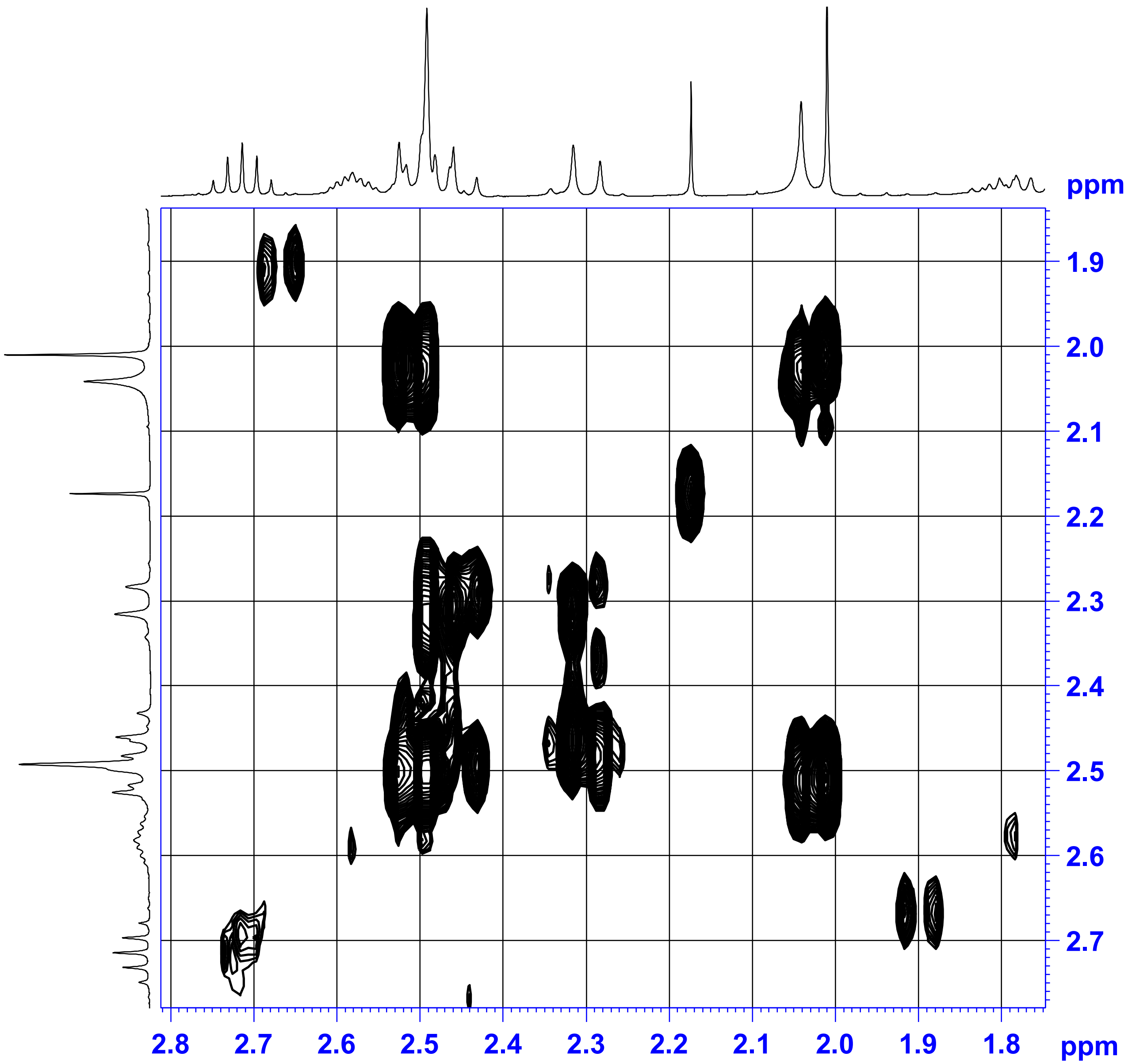

^1H-^1H COSY spectrum of Krishnolide C (**3**) in $CDCl_3$

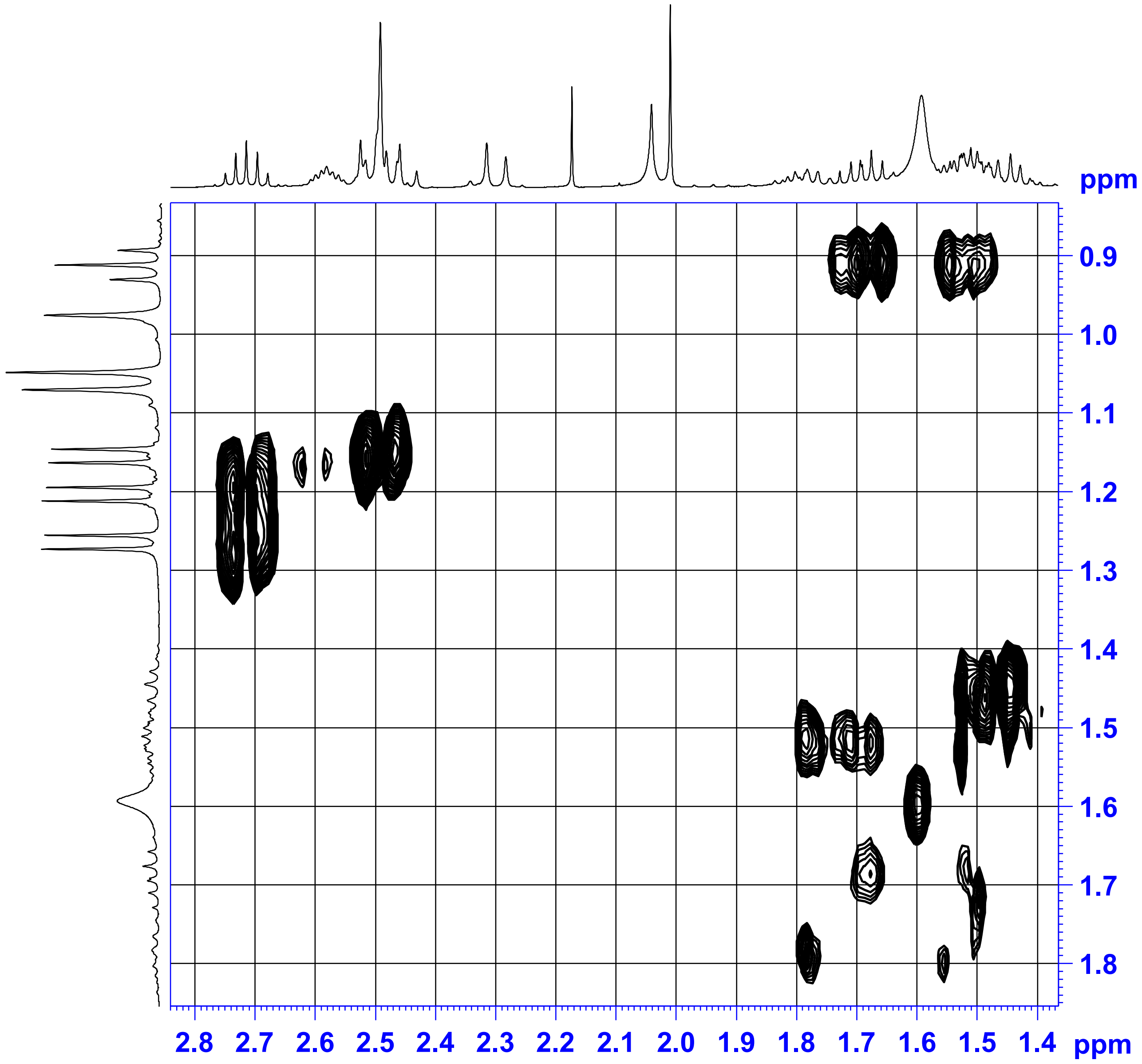

^{1}H-^{1}H COSY spectrum of Krishnolide C (**3**) in $CDCl_3$

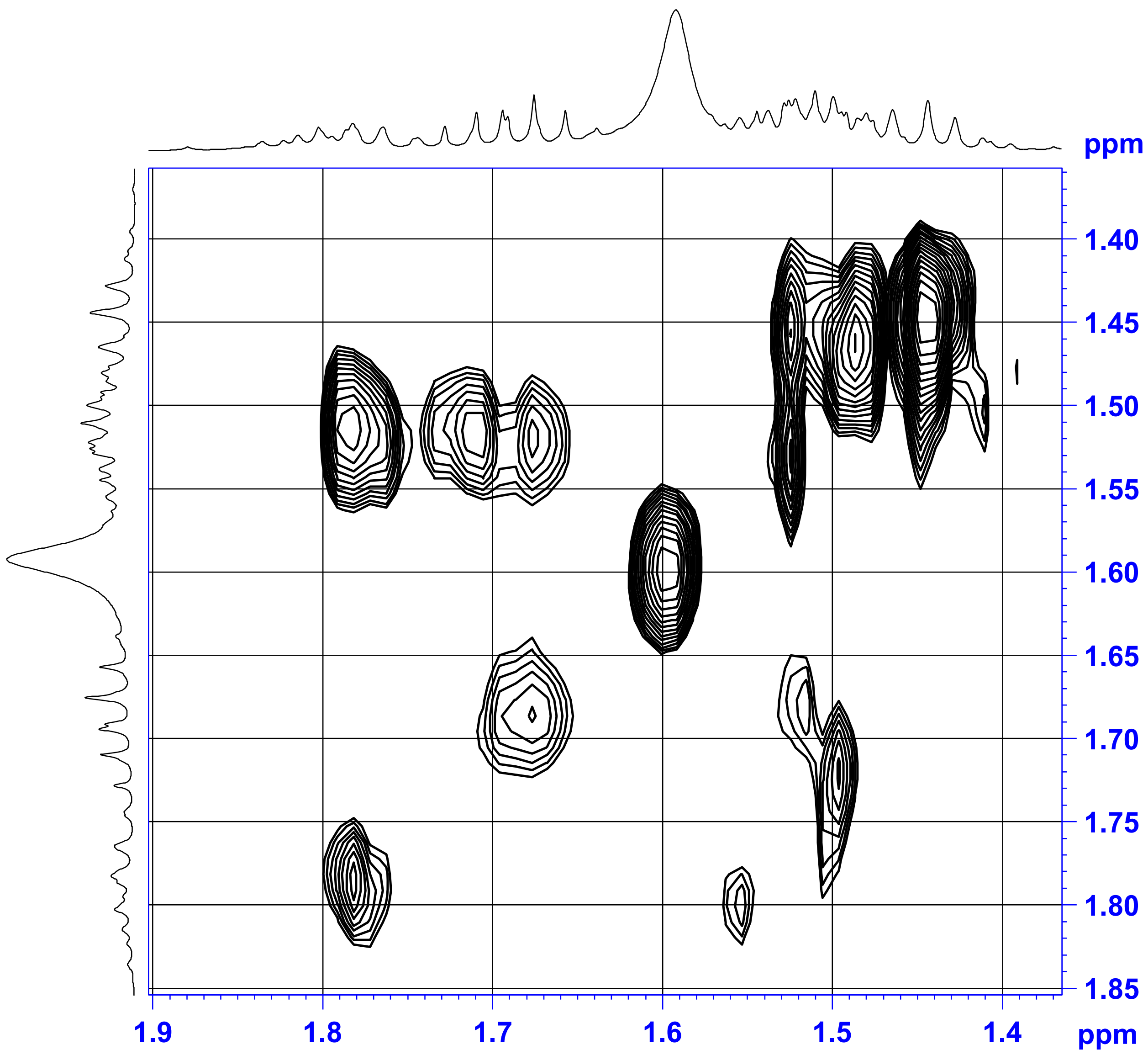

HSQC spectrum of Krishnolide C (**3**) in $CDCl_3$

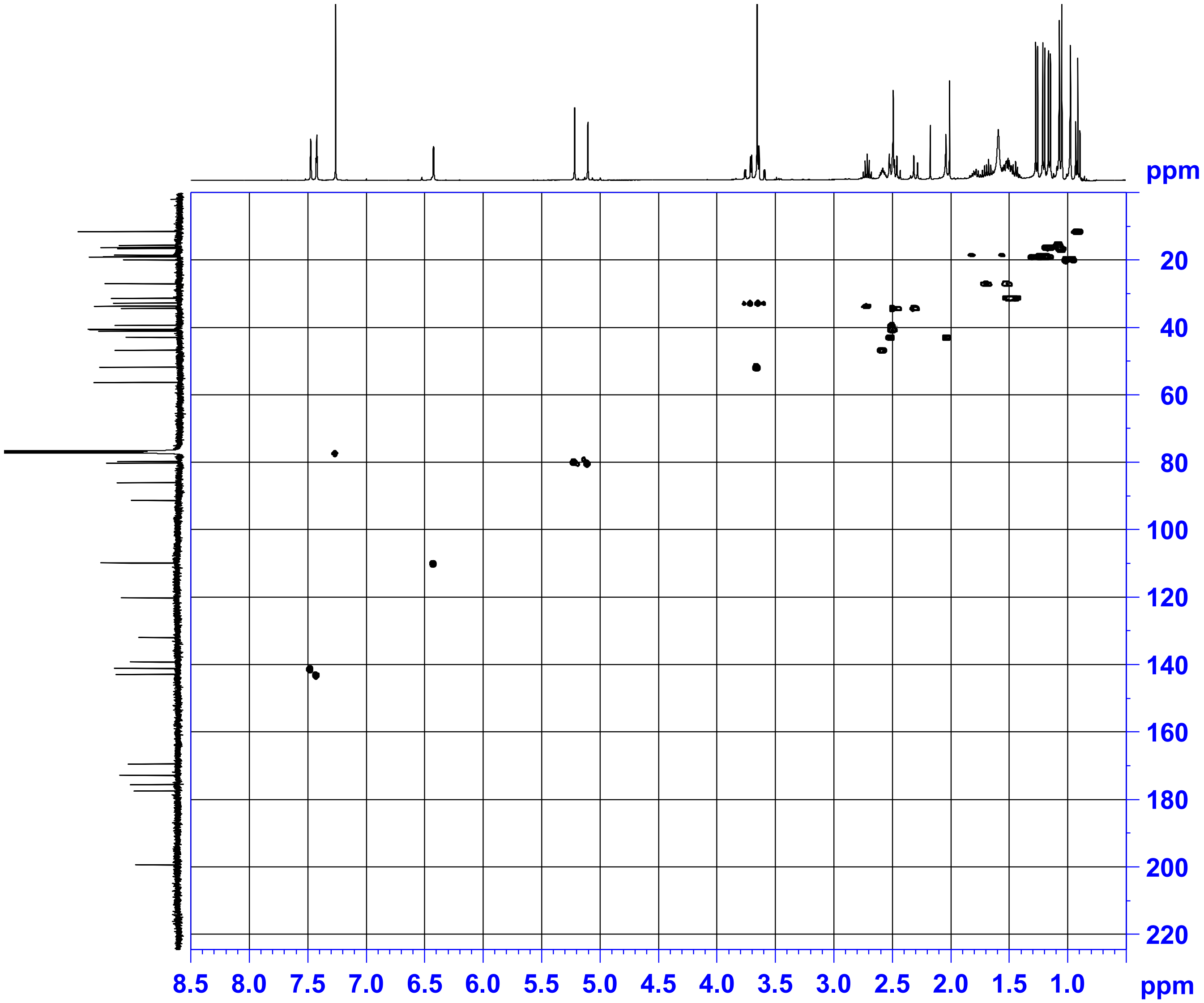

NAME	zq-26
EXPNO	5
PROCNO	1
Date_	20170707
Time	5.37
INSTRUM	spect
PROBHD	5 mm CPPBBO BB
PULPROG	hsqcetgpsi2
TD	1024
SOLVENT	CDCl3
NS	16
DS	16
SWH	4302.926 Hz
FIDRES	4.202076 Hz
AQ	0.1190388 sec
RG	208.5
DW	116.200 usec
DE	10.00 usec
TE	297.0 K
CNST2	145.0000000
D0	0.00000300 sec
D1	1.46497905 sec
D4	0.00172414 sec
D11	0.03000000 sec
D16	0.00020000 sec
D24	0.00086207 sec
IN0	0.00002080 sec
ZGOPTNS	
======== CHANNEL f1 ========	
SFO1	400.1320007 MHz
NUC1	1H
P1	11.50 usec
P2	23.00 usec
P28	0.00 usec
ND0	2
TD	256
SFO1	100.6233 MHz
FIDRES	93.900238 Hz
SW	238.896 ppm
FnMODE	Echo-Antiecho
SI	1024
SF	400.1300063 MHz
WDW	QSINE
SSB	2
LB	0.00 Hz
GB	0
PC	1.40
SI	1024
MC2	echo-antiecho
SF	100.6127593 MHz
WDW	QSINE
SSB	2
LB	0.00 Hz
GB	0

HSQC spectrum of Krishnolide C (**3**) in $CDCl_3$

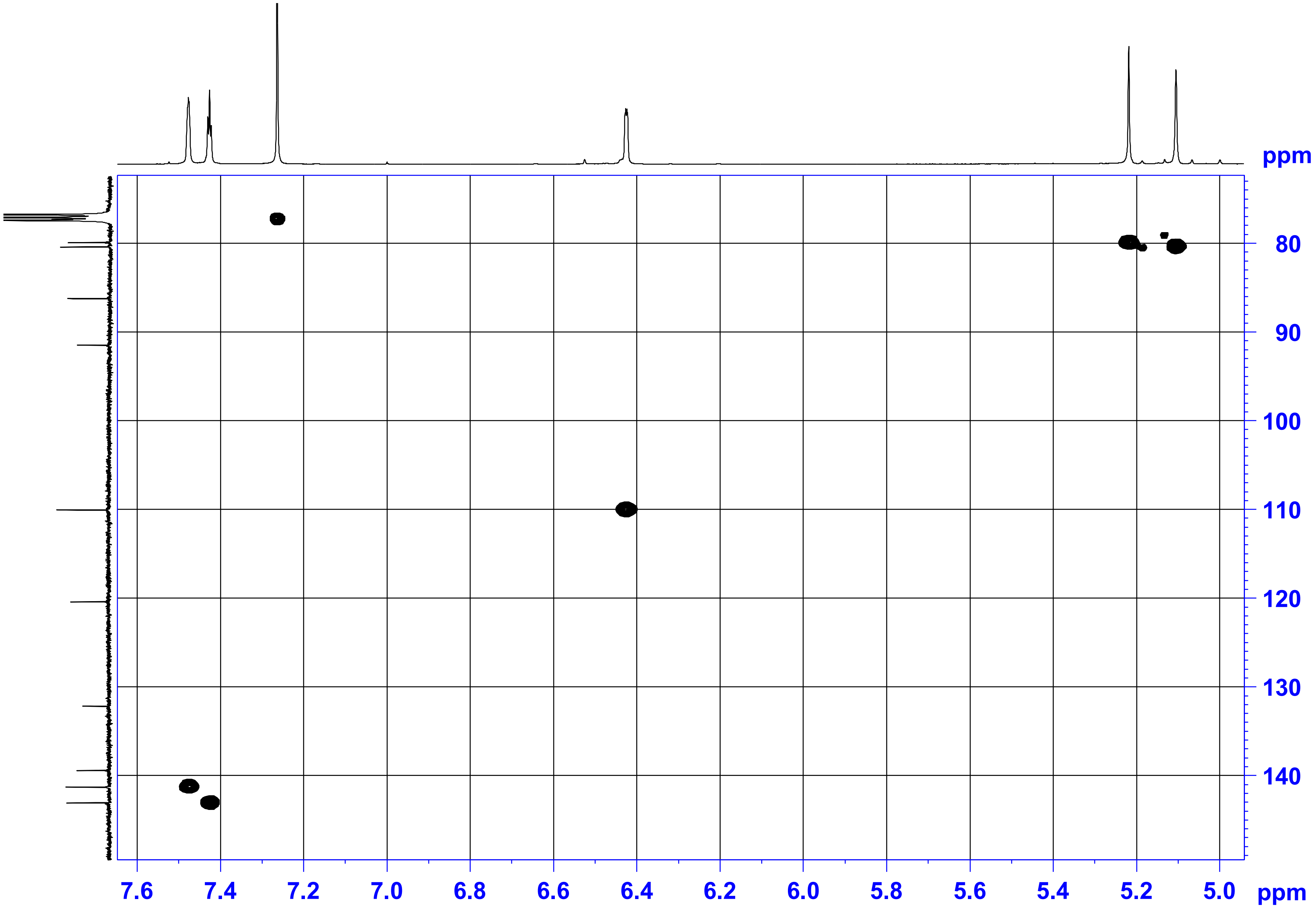

HSQC spectrum of Krishnolide C (**3**) in $CDCl_3$

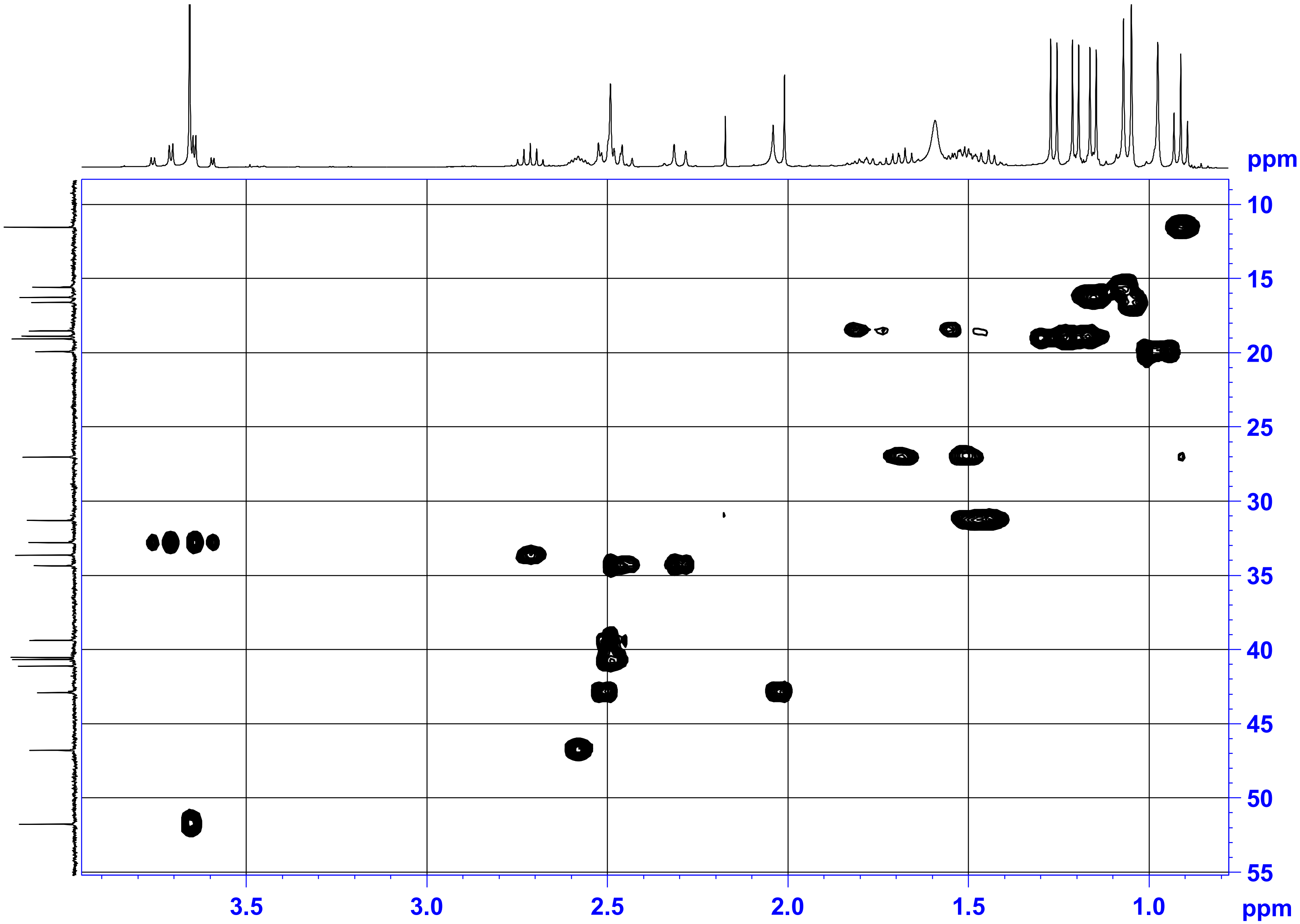

HSQC spectrum of Krishnolide C (**3**) in $CDCl_3$

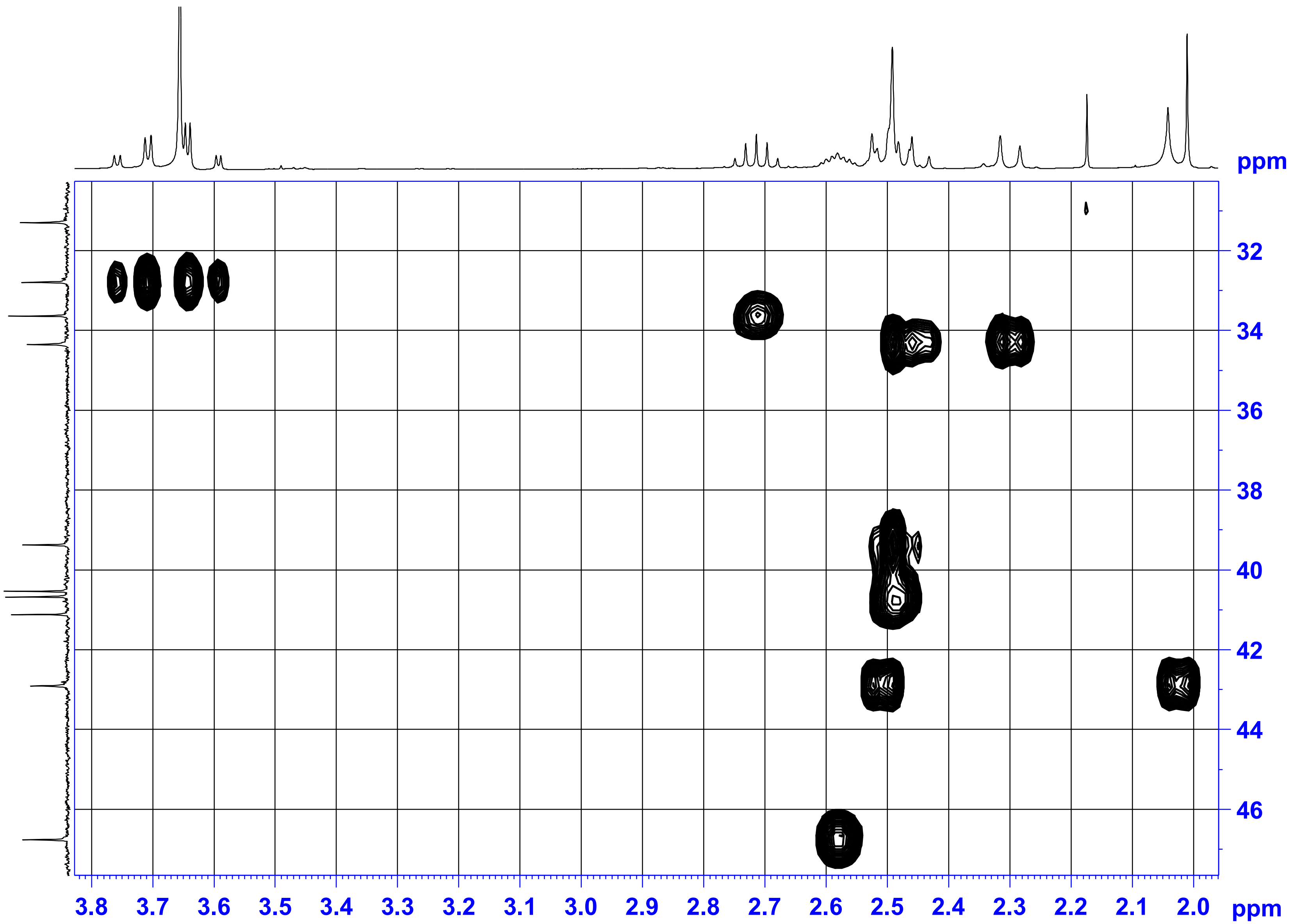

HSQC spectrum of Krishnolide C (**3**) in $CDCl_3$

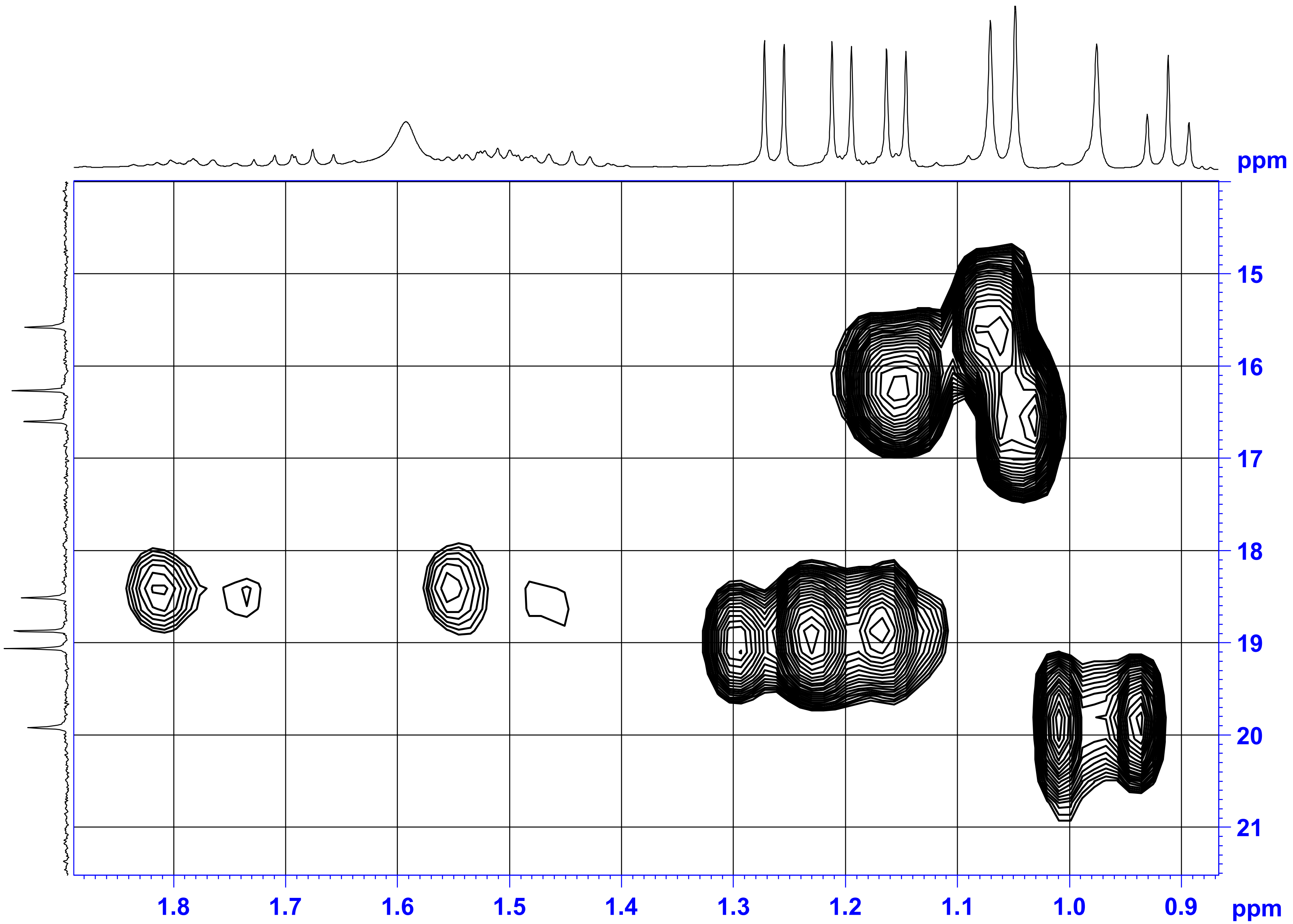

HMBC spectrum of Krishnolide C (**3**) in $CDCl_3$

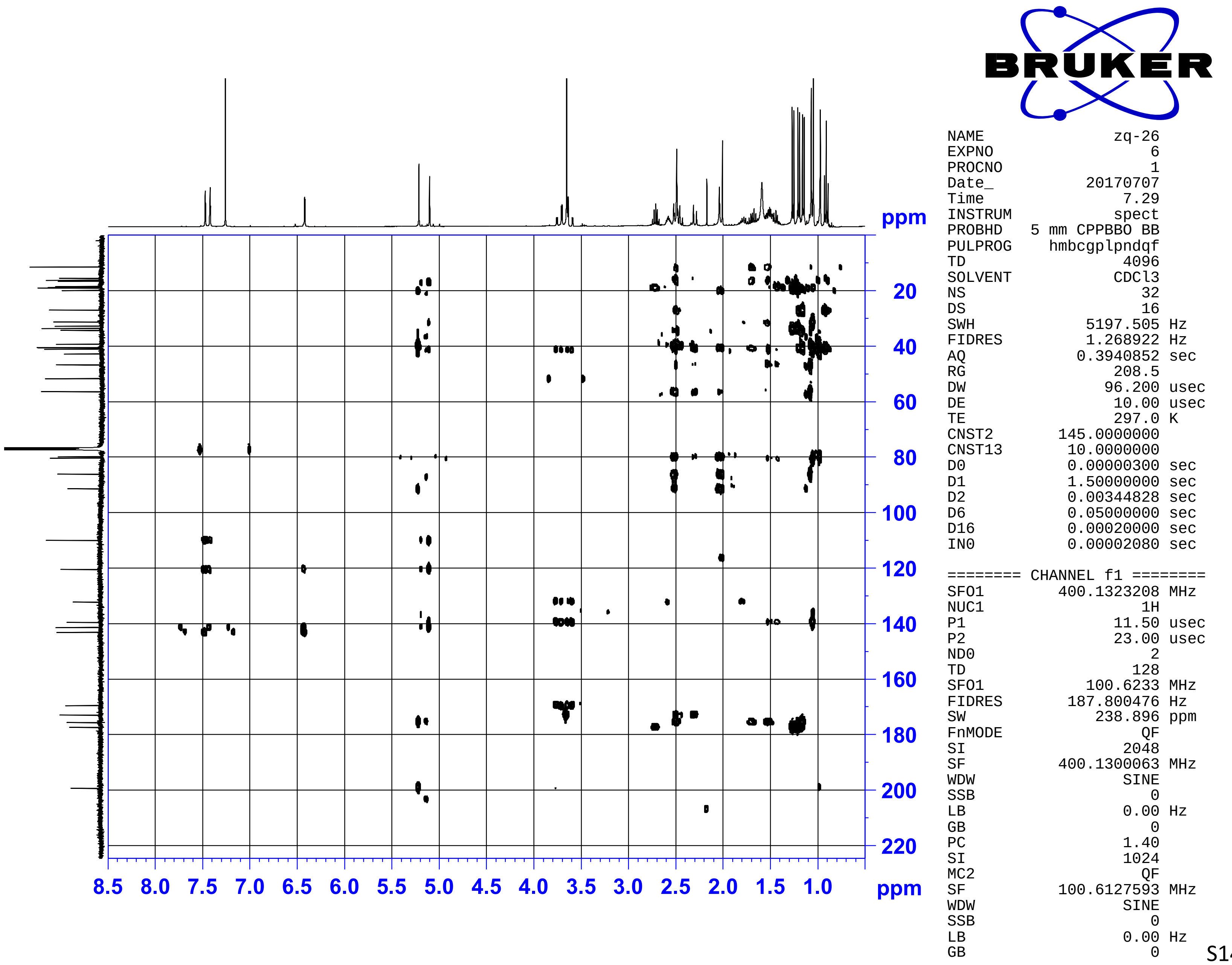

HMBC spectrum of Krishnolide C (**3**) in $CDCl_3$

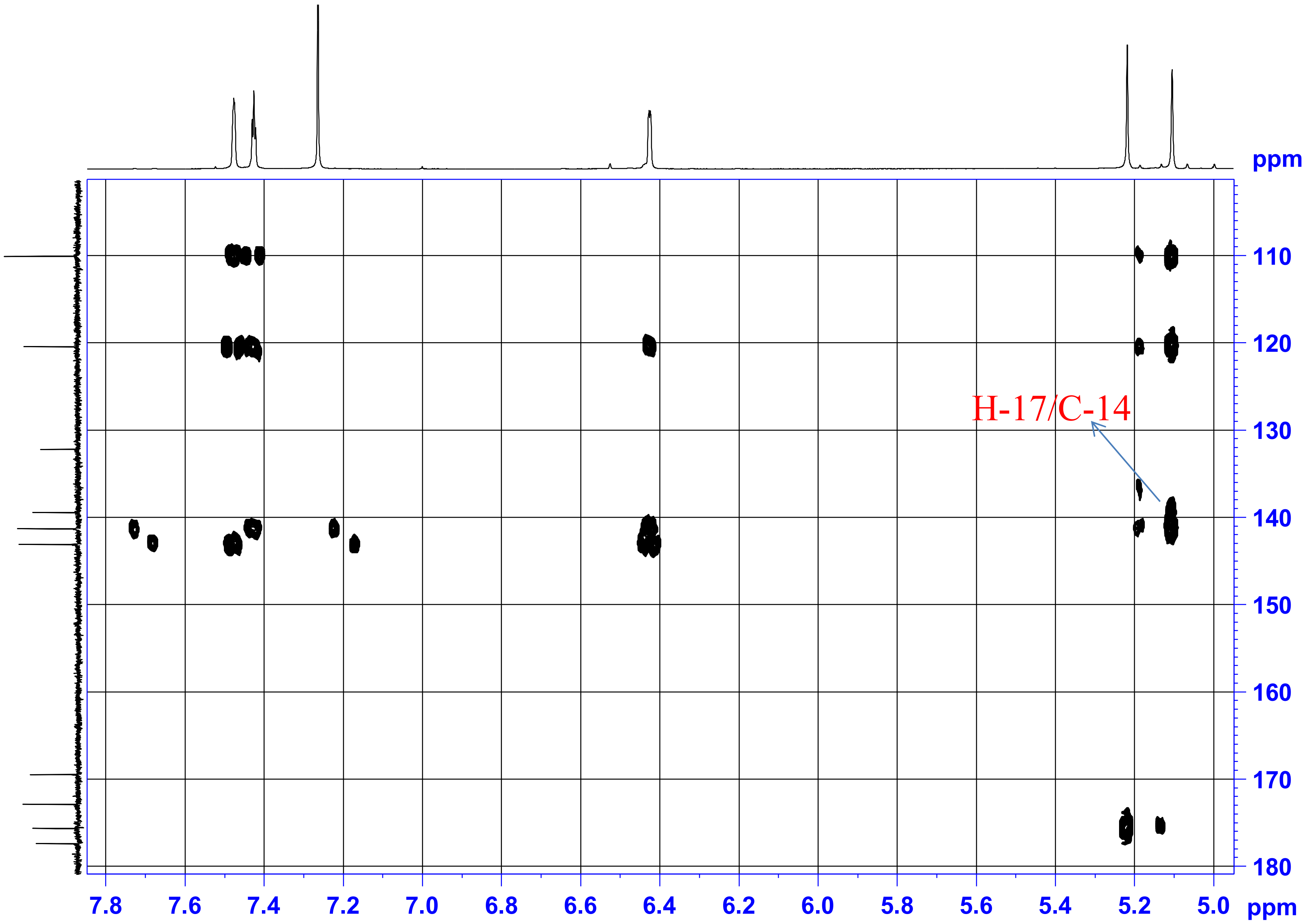

HMBC spectrum of Krishnolide C (**3**) in $CDCl_3$

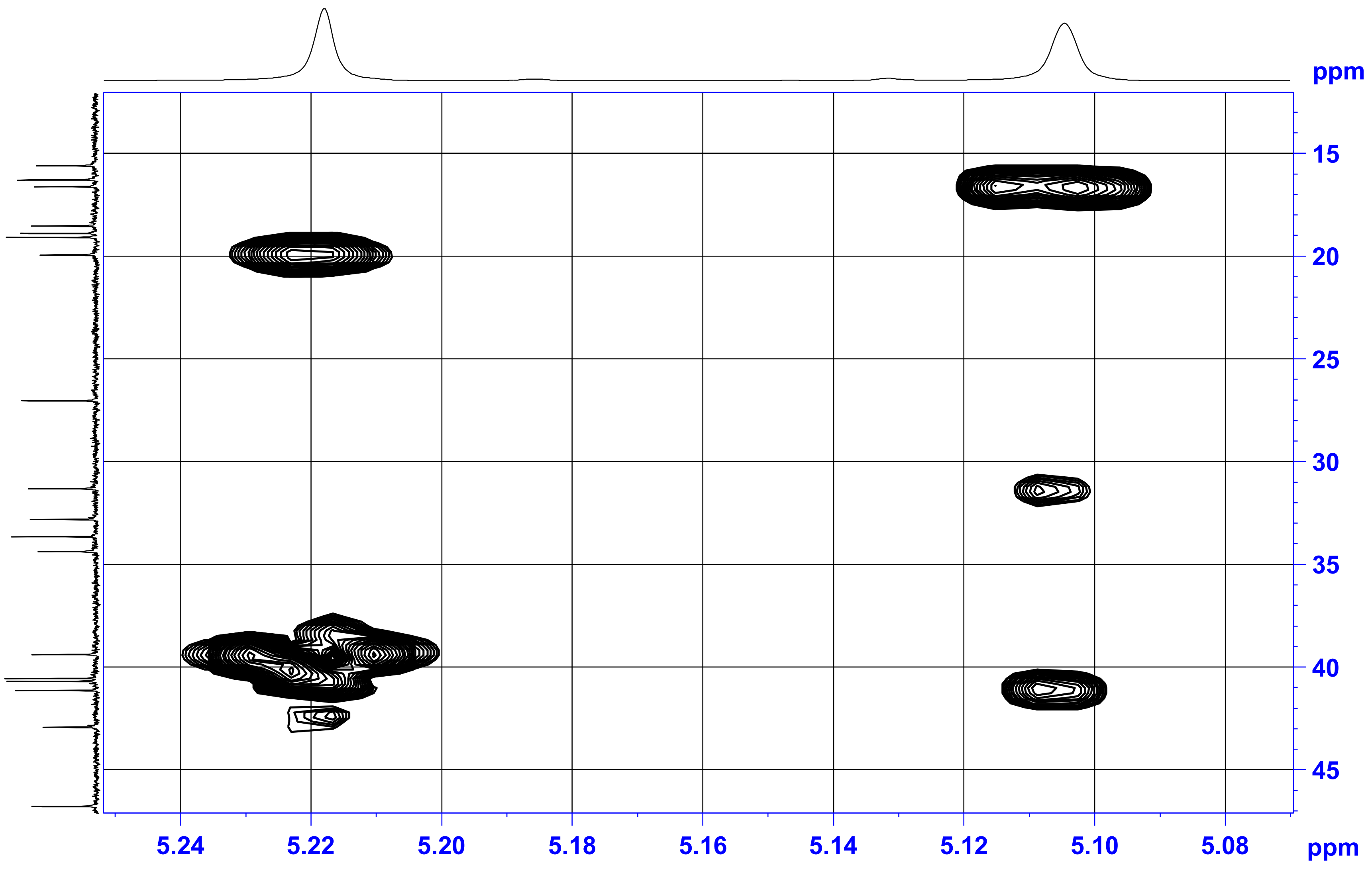

HMBC spectrum of Krishnolide C (**3**) in $CDCl_3$

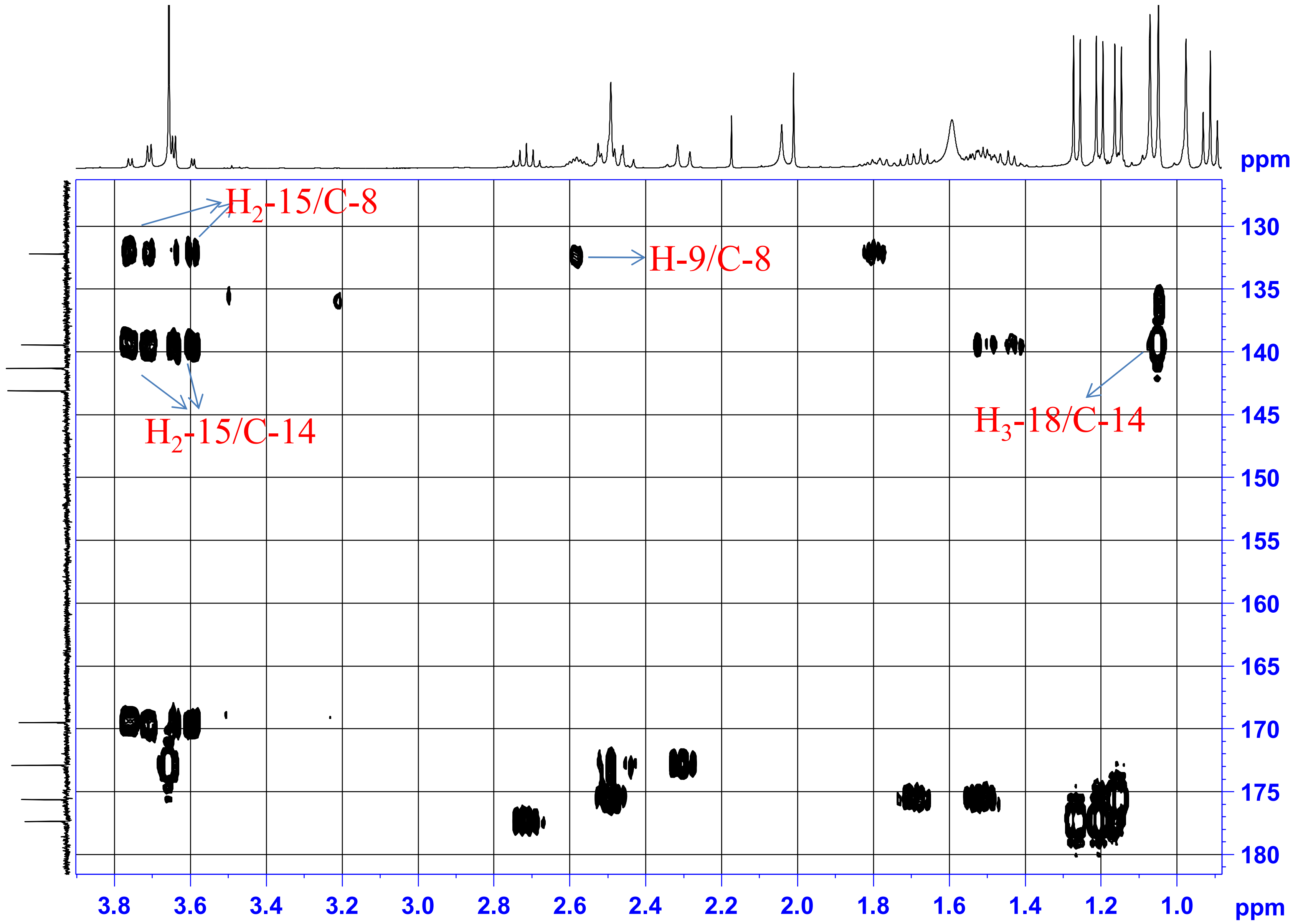

HMBC spectrum of Krishnolide C (**3**) in $CDCl_3$

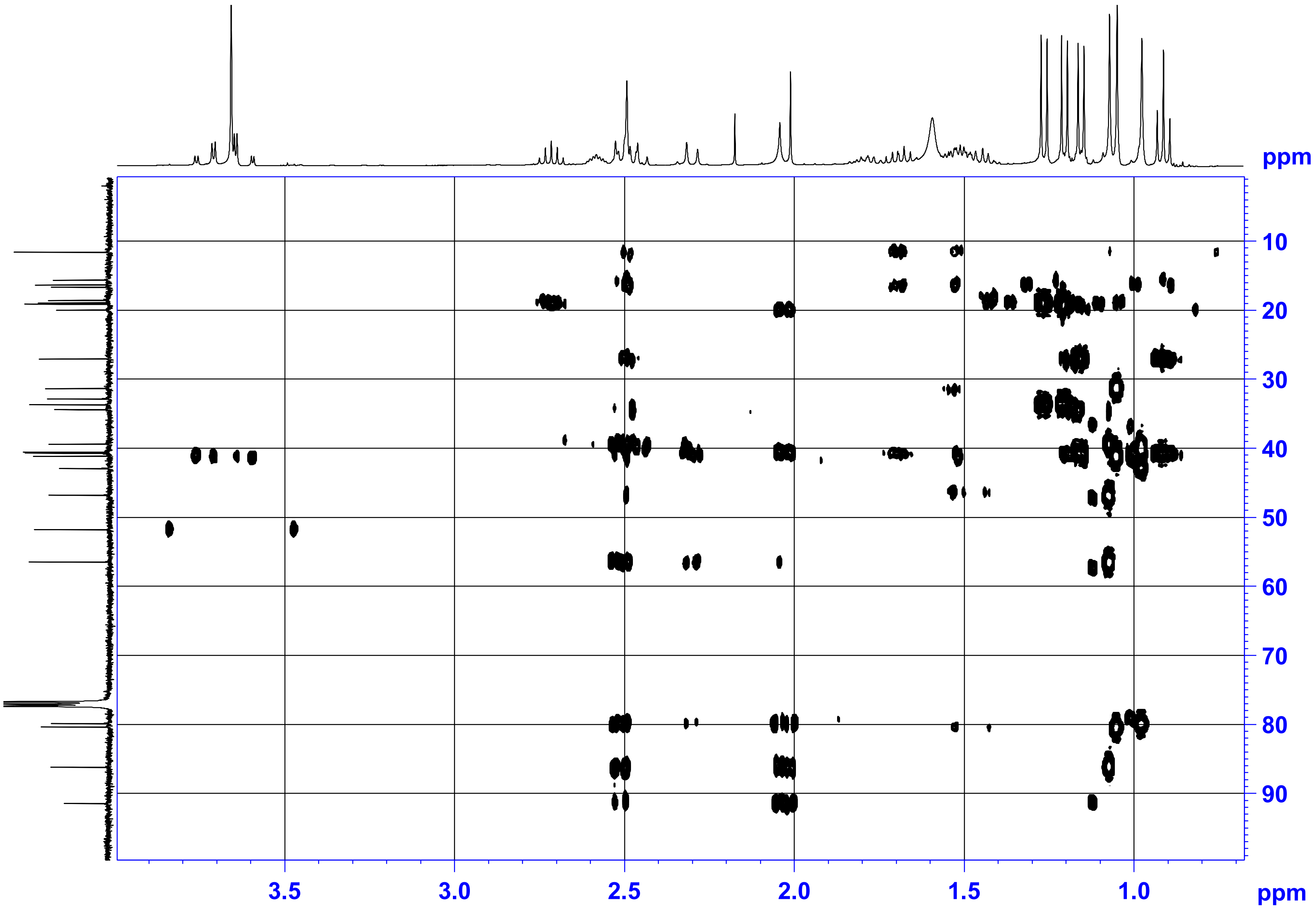

HMBC spectrum of Krishnolide C (**3**) in $CDCl_3$

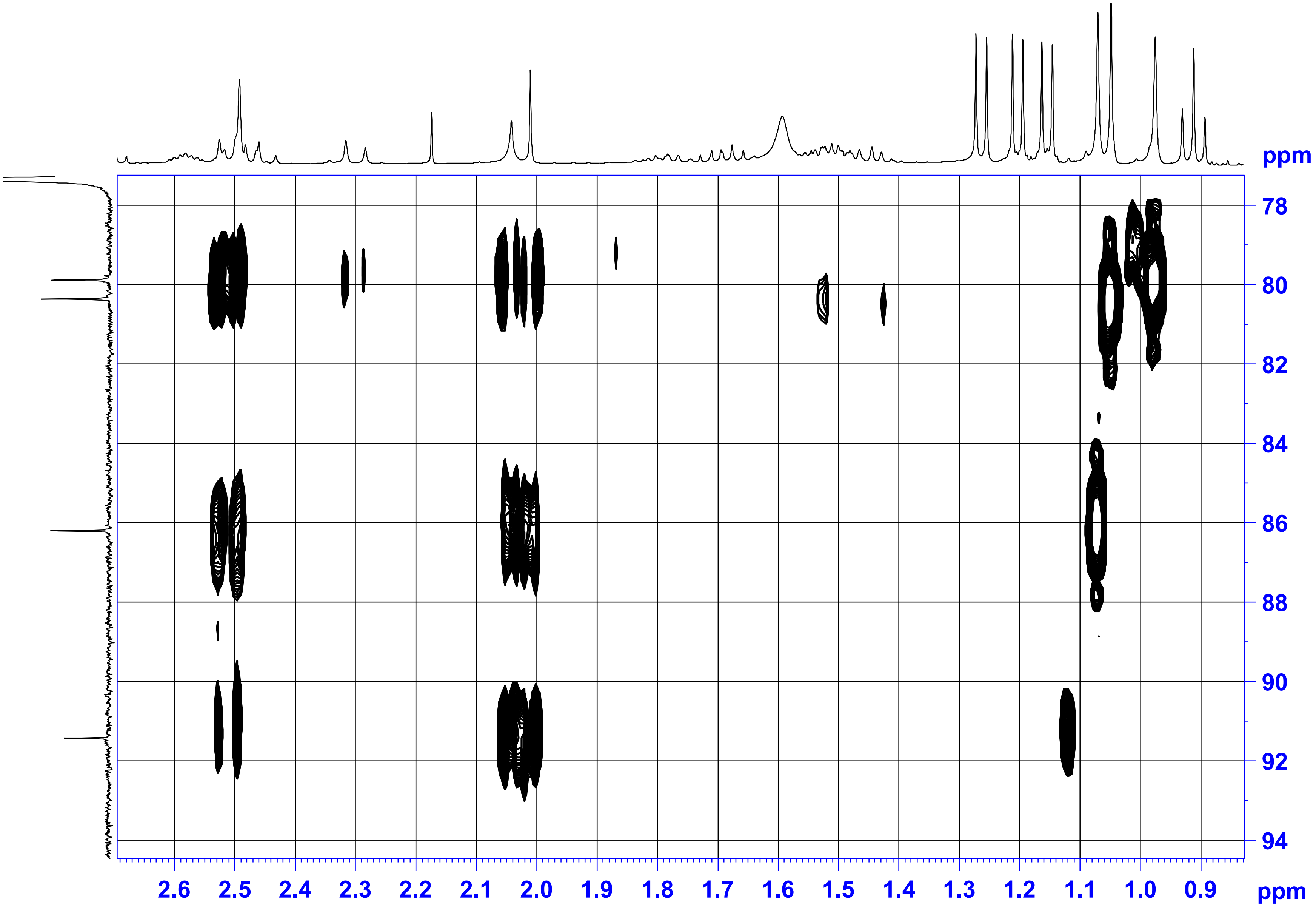

HMBC spectrum of Krishnolide C (**3**) in $CDCl_3$

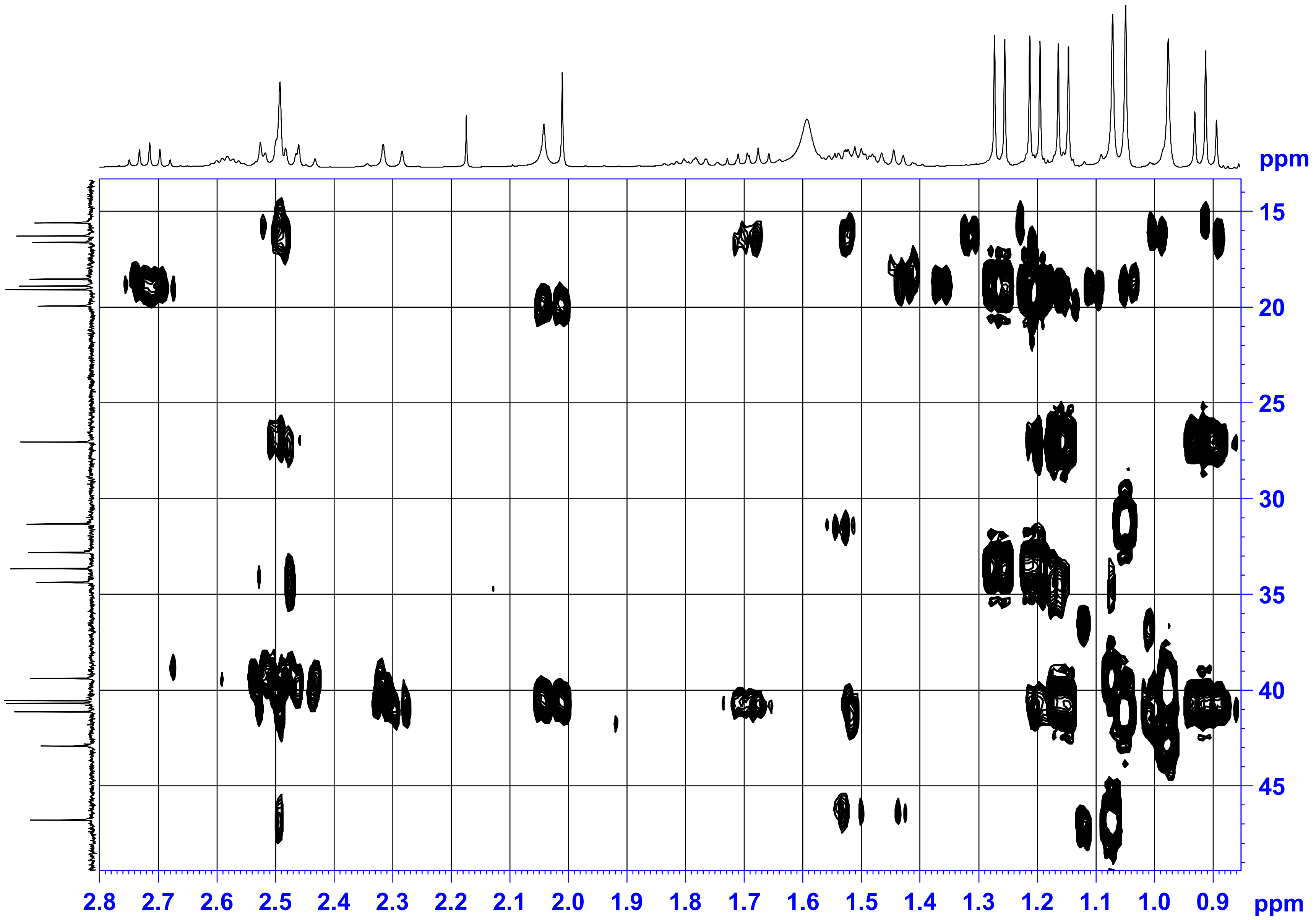

HMBC spectrum of Krishnolide C (**3**) in $CDCl_3$

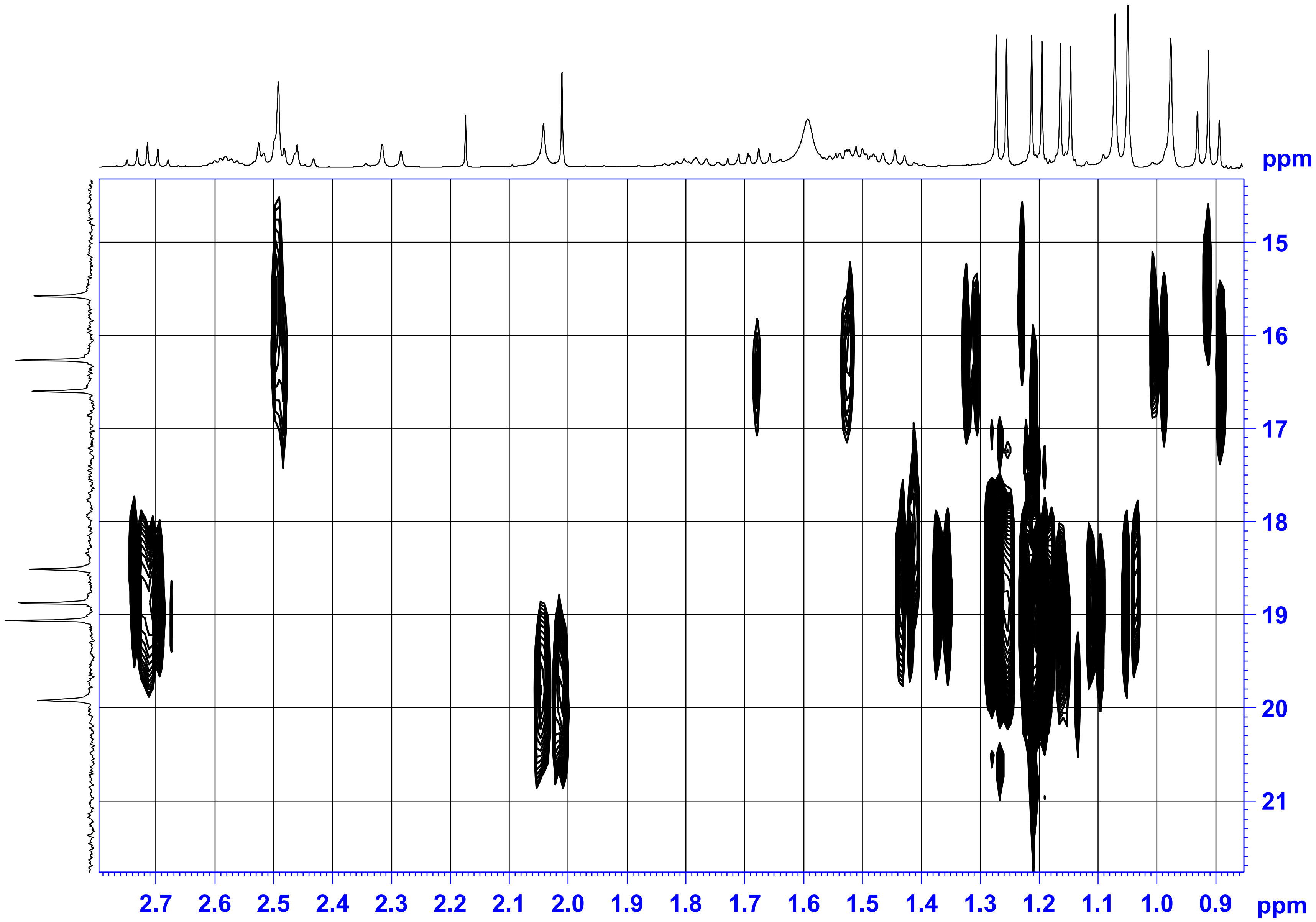

HMBC spectrum of Krishnolide C (**3**) in $CDCl_3$

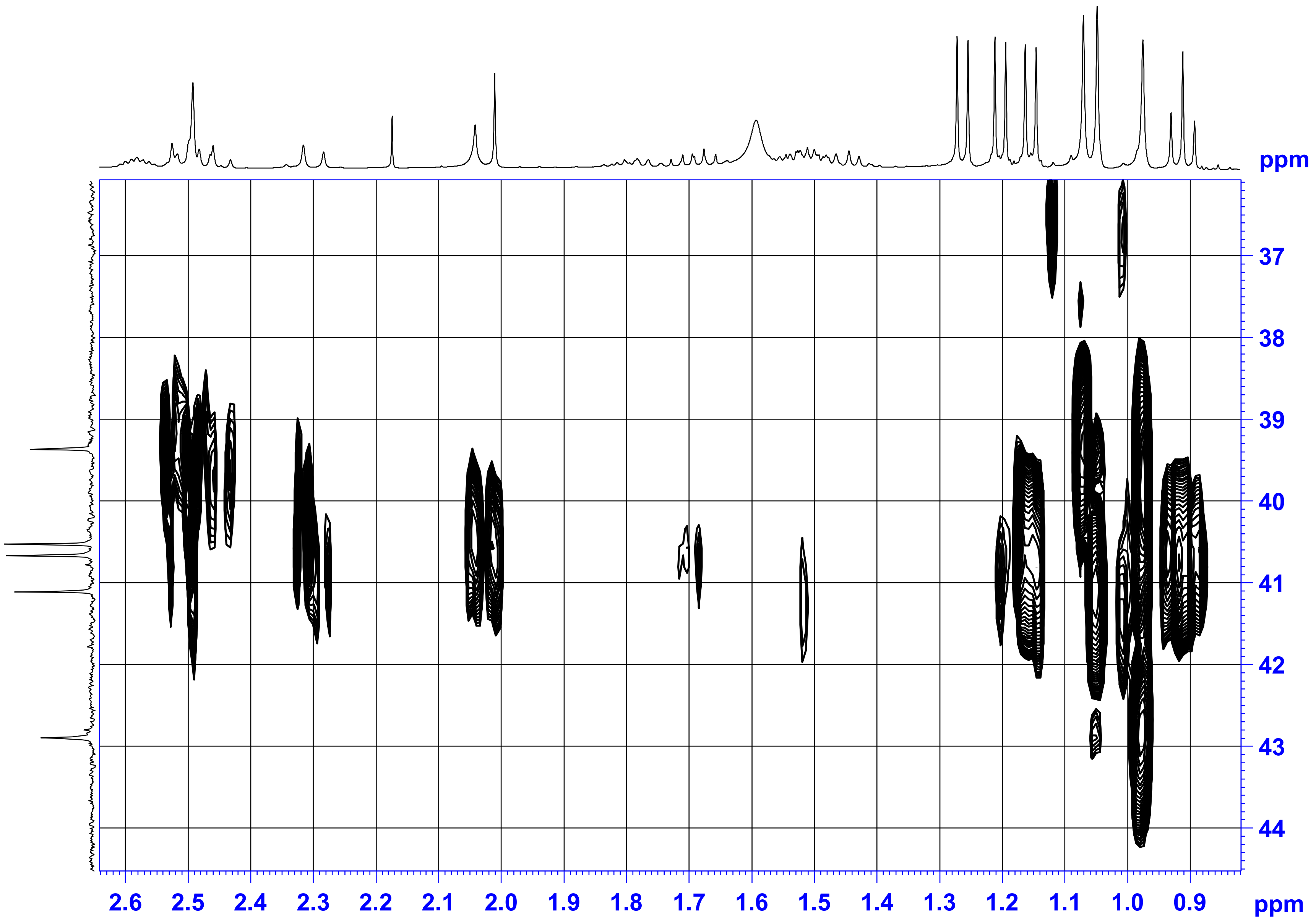

NOESY spectrum of Krishnolide C (**3**) in $CDCl_3$

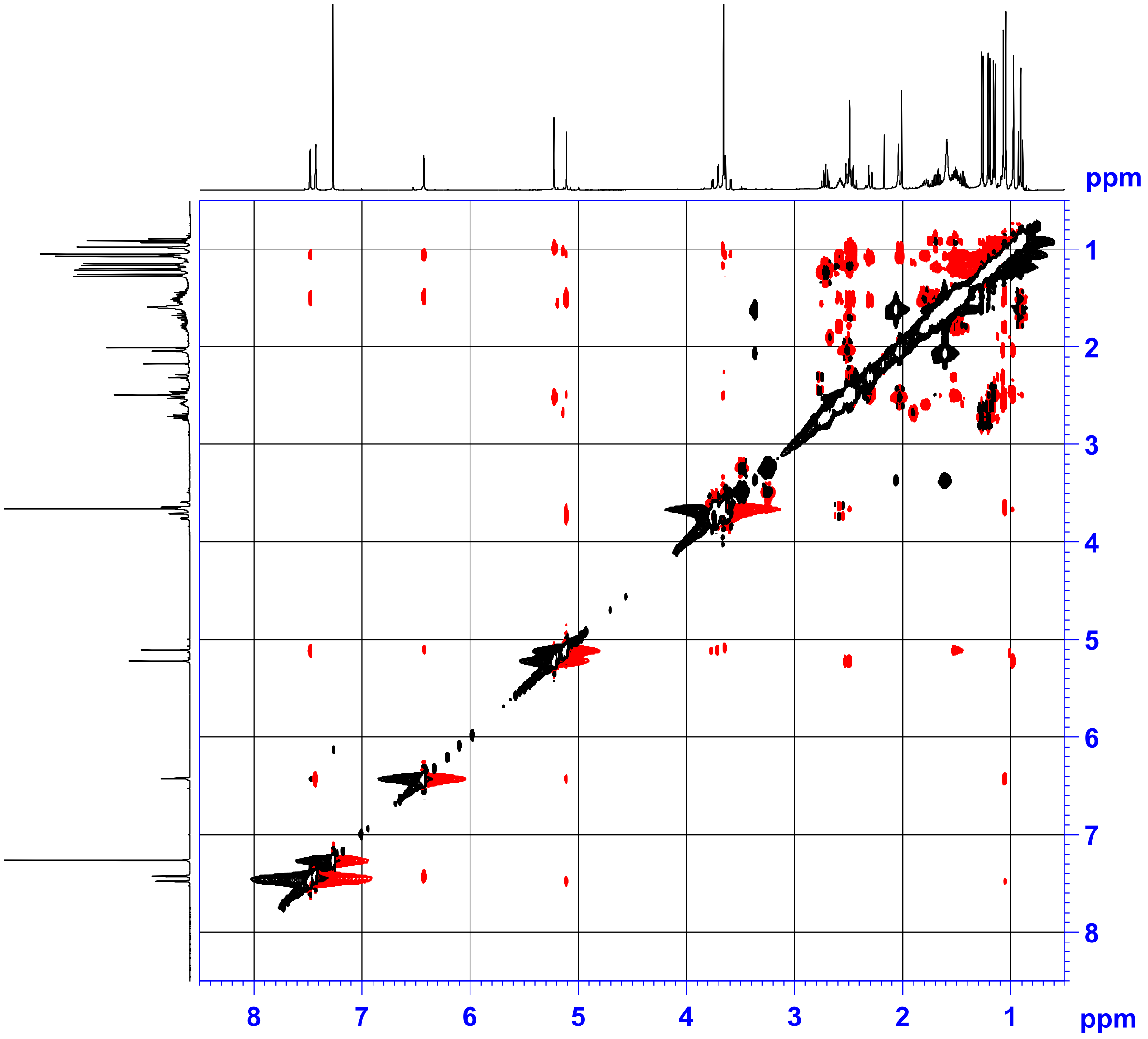

NAME	zq-26
EXPNO	7
PROCNO	1
Date_	20170710
Time	4.49
INSTRUM	spect
PROBHD	5 mm CPPBBO BB
PULPROG	noesygpphpp
TD	2048
SOLVENT	CDCl3
NS	16
DS	32
SWH	4000.000 Hz
FIDRES	1.953125 Hz
AQ	0.2560500 sec
RG	208.5
DW	125.000 usec
DE	10.00 usec
TE	297.0 K
D0	0.00011036 sec
D1	1.99385595 sec
D8	0.30000001 sec
D11	0.03000000 sec
D12	0.00002000 sec
D16	0.00020000 sec
IN0	0.00025000 sec
======== CHANNEL f1 ========	
SFO1	400.1318006 MHz
NUC1	1H
P1	11.50 usec
P2	23.00 usec
P17	2500.00 usec
ND0	1
TD	256
SFO1	400.1318 MHz
FIDRES	15.625000 Hz
SW	9.997 ppm
FnMODE	States-TPPI
SI	1024
SF	400.1300063 MHz
WDW	QSINE
SSB	2
LB	0.00 Hz
GB	0
PC	1.00
SI	1024
MC2	States-TPPI
SF	400.1300063 MHz
WDW	QSINE
SSB	2
LB	0.00 Hz
GB	0

NOESY spectrum of Krishnolide C (**3**) in $CDCl_3$

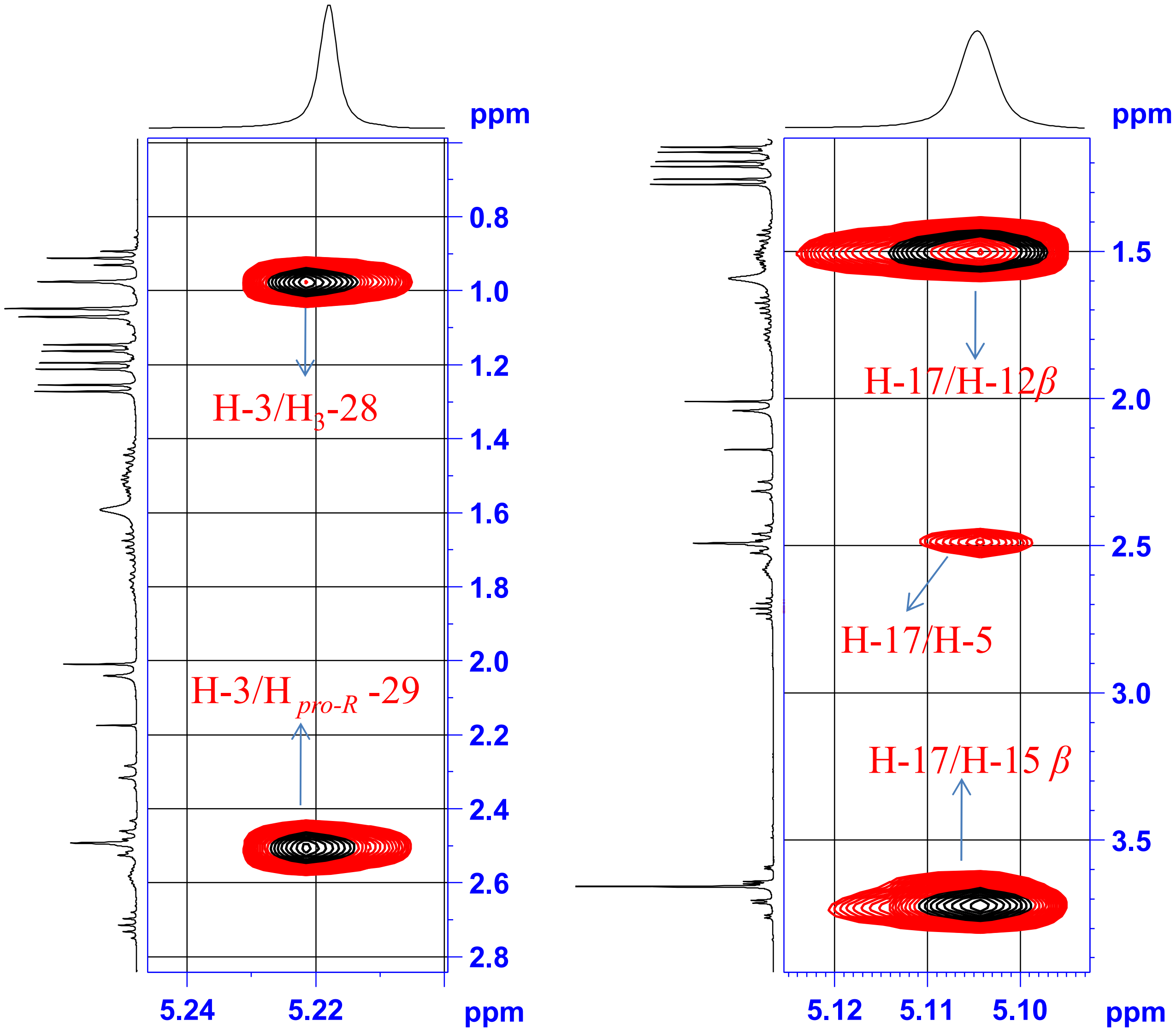

NOESY spectrum of Krishnolide C (**3**) in $CDCl_3$

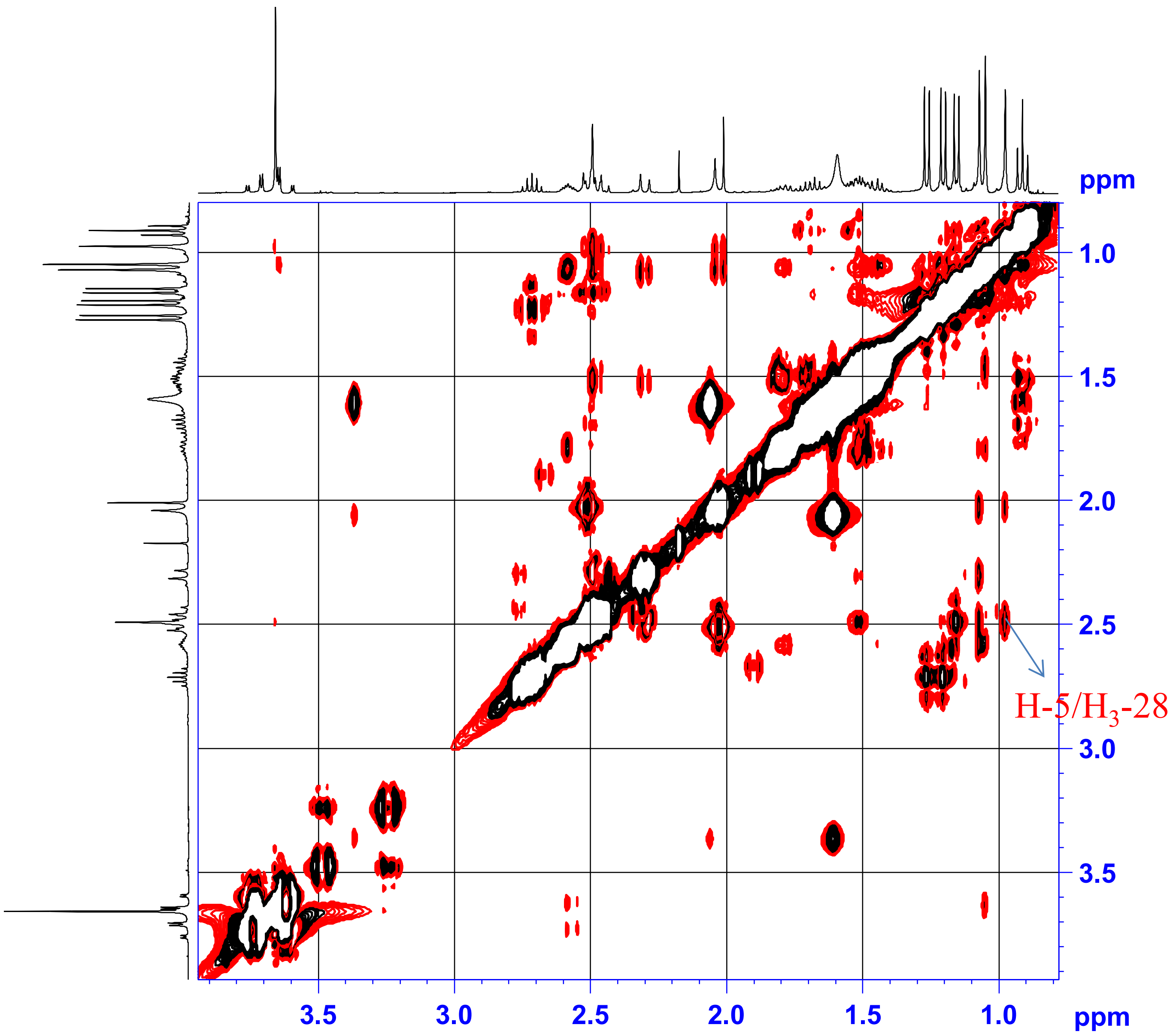

NOESY spectrum of Krishnolide C (**3**) in $CDCl_3$

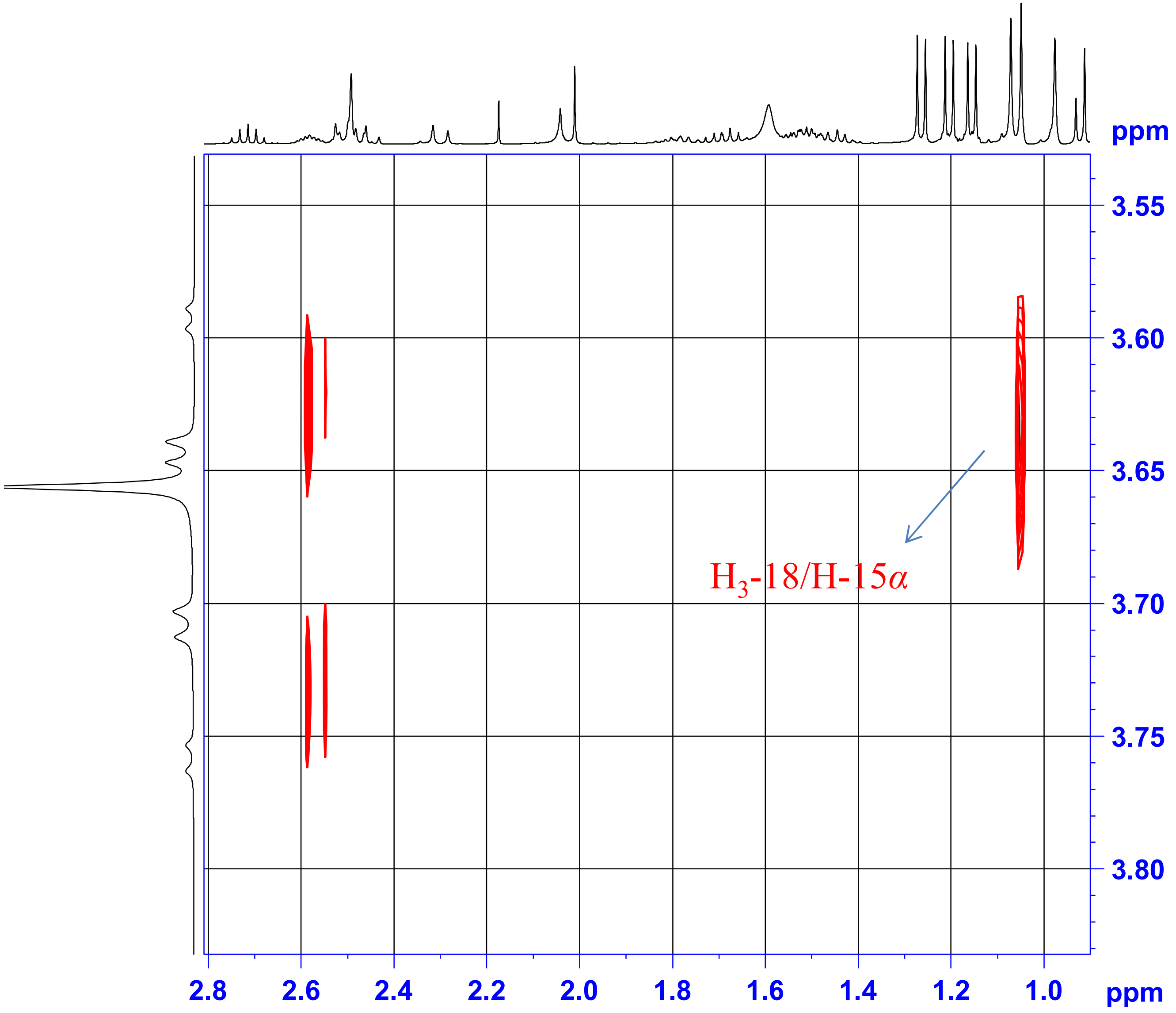

NOESY spectrum of Krishnolide C (**3**) in $CDCl_3$

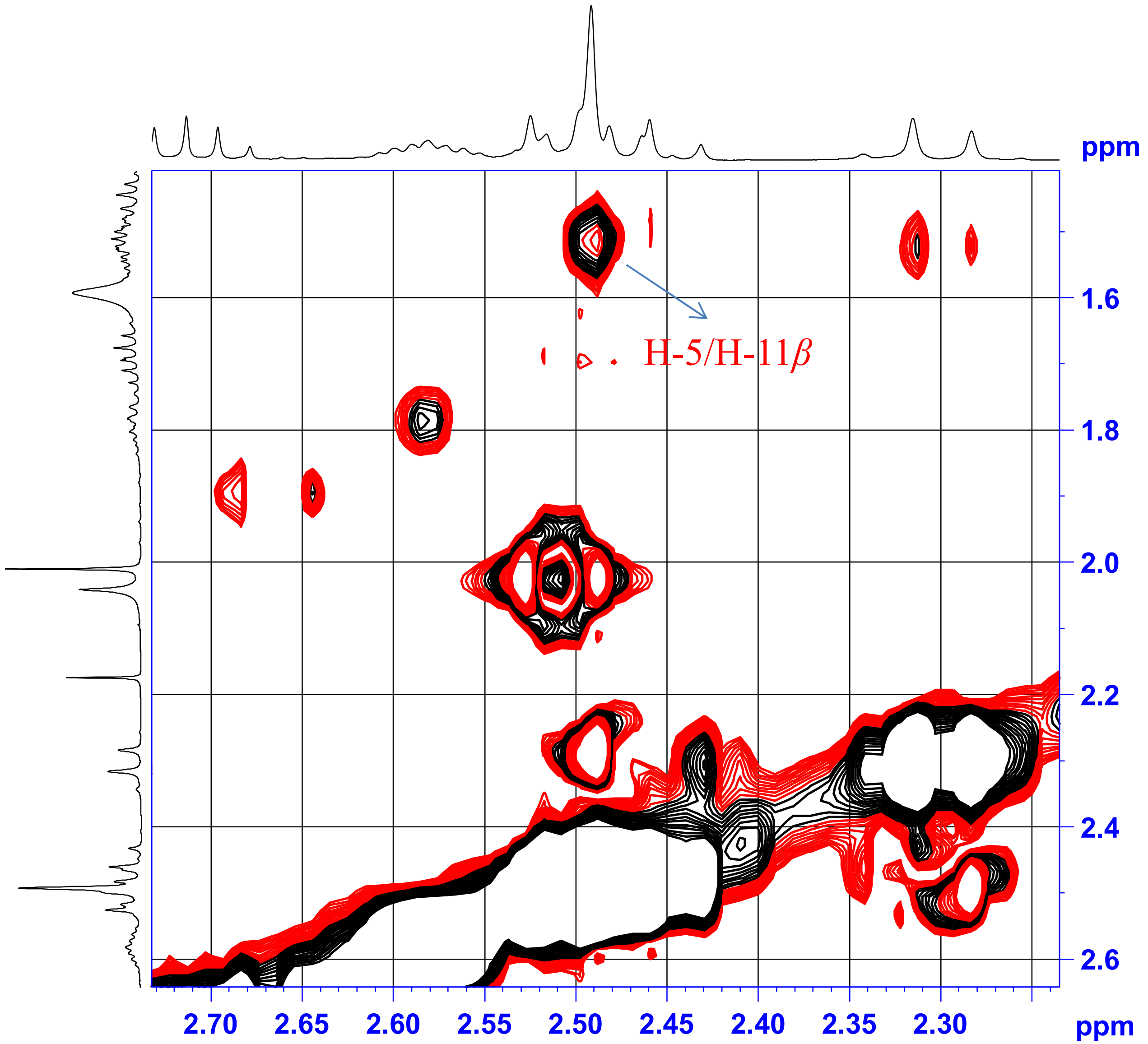

NOESY spectrum of Krishnolide C (**3**) in $CDCl_3$

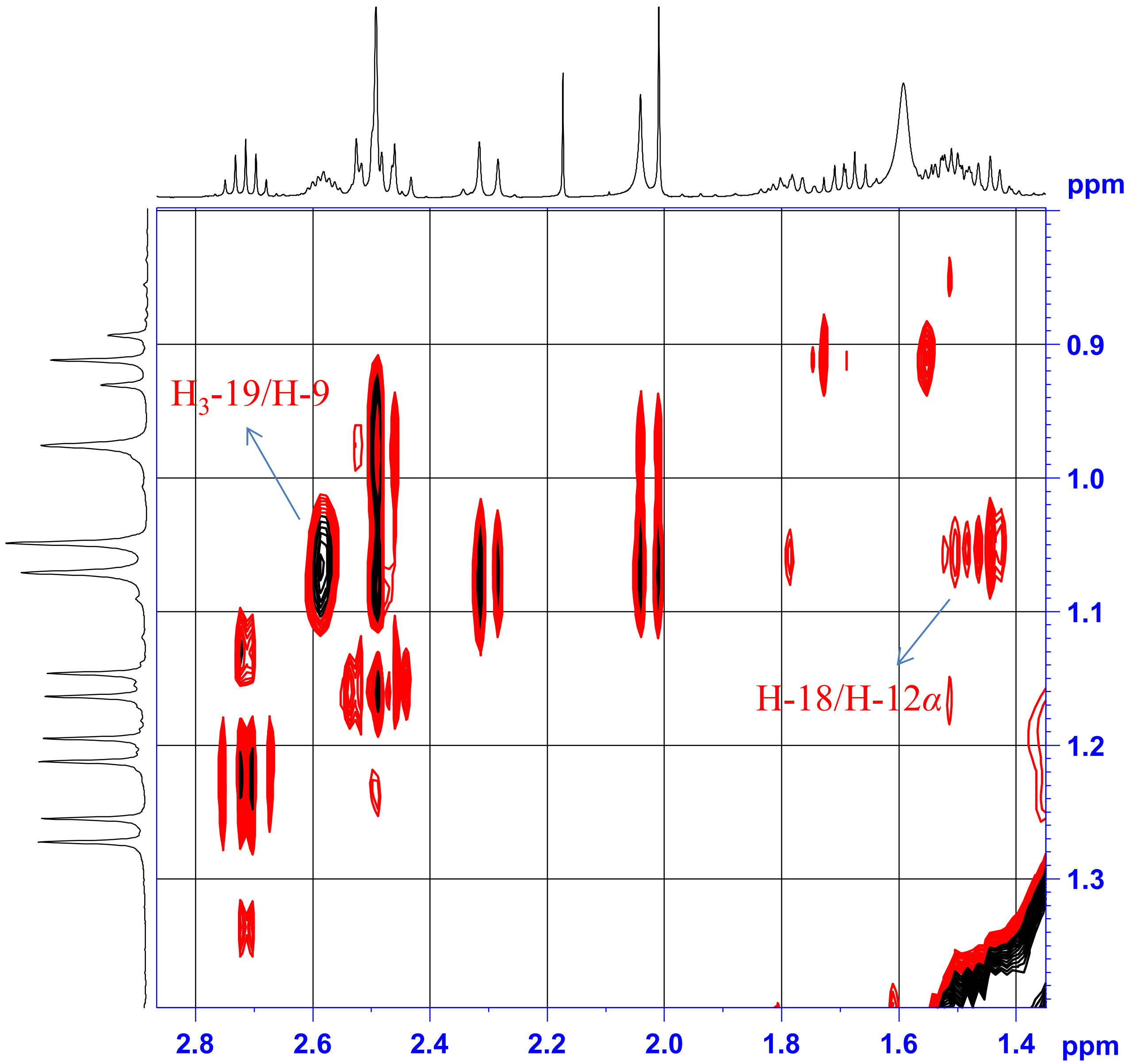

HR-ESIMS of Krishnolide D (4)

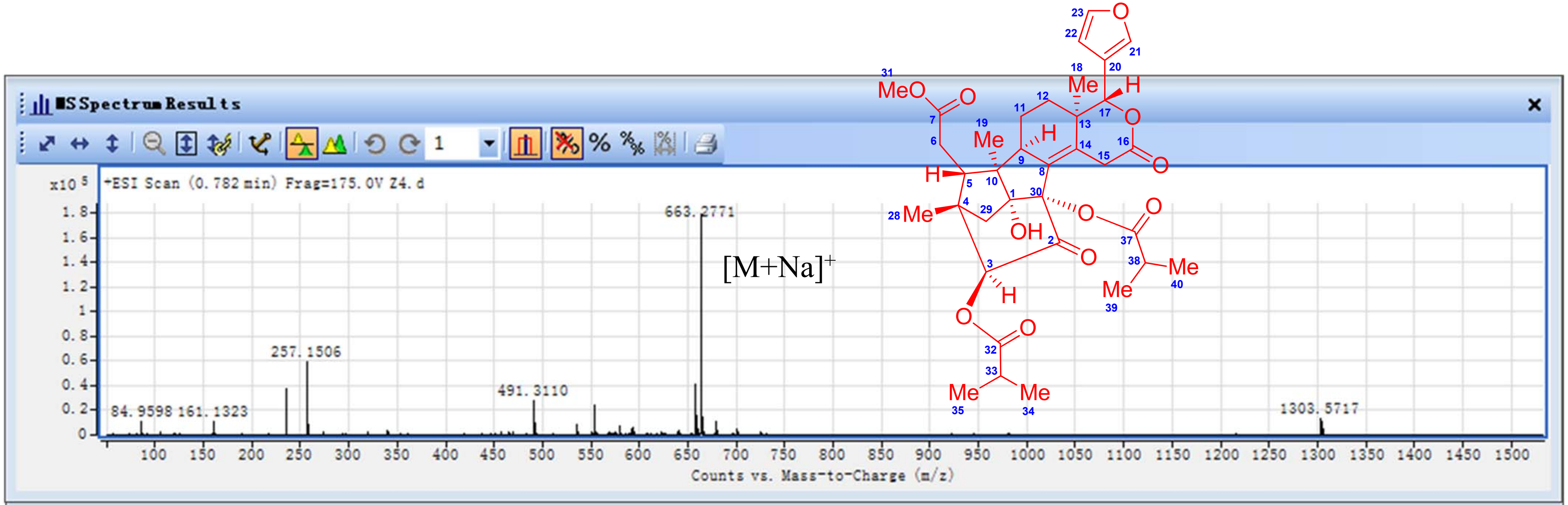

MS Formula Results: + Scan (0.782 min)

m/z	Ion	Formula	Abundance
663.2771	(M+Na)+	C35 H44 Na O11	179689.7

Best	Formula (M)	Ion Formula	Score	Cross Score	Calc m/z	Diff (ppm)	Mass Match	Abund Match	Spacing Match
☑	C35 H44 O11	C35 H44 Na O11	96.3		663.2776	0.82	99.16	89.96	98.19

Isotope	Abund Sum%	Calc Abund Sum%	m/z	Calc m/z	Diff (ppm)
1	69.94	66.61	663.2771	663.2776	0.76
2	23.49	25.83	664.2799	664.281	1.64
3	5.58	6.38	665.2821	665.2838	2.53
4	0.98	1.18	666.2862	666.2865	0.52

Best	Formula (M)	Ion Formula	Score	Cross Score	Calc m/z	Diff (ppm)	Mass Match	Abund Match	Spacing Match
☐	C42 H40 O6	C42 H40 Na O6	59.52		663.2717	-8.35	41.66	57.51	97.66

UV spectrum of Krishnolide D (**4**) in MeCN

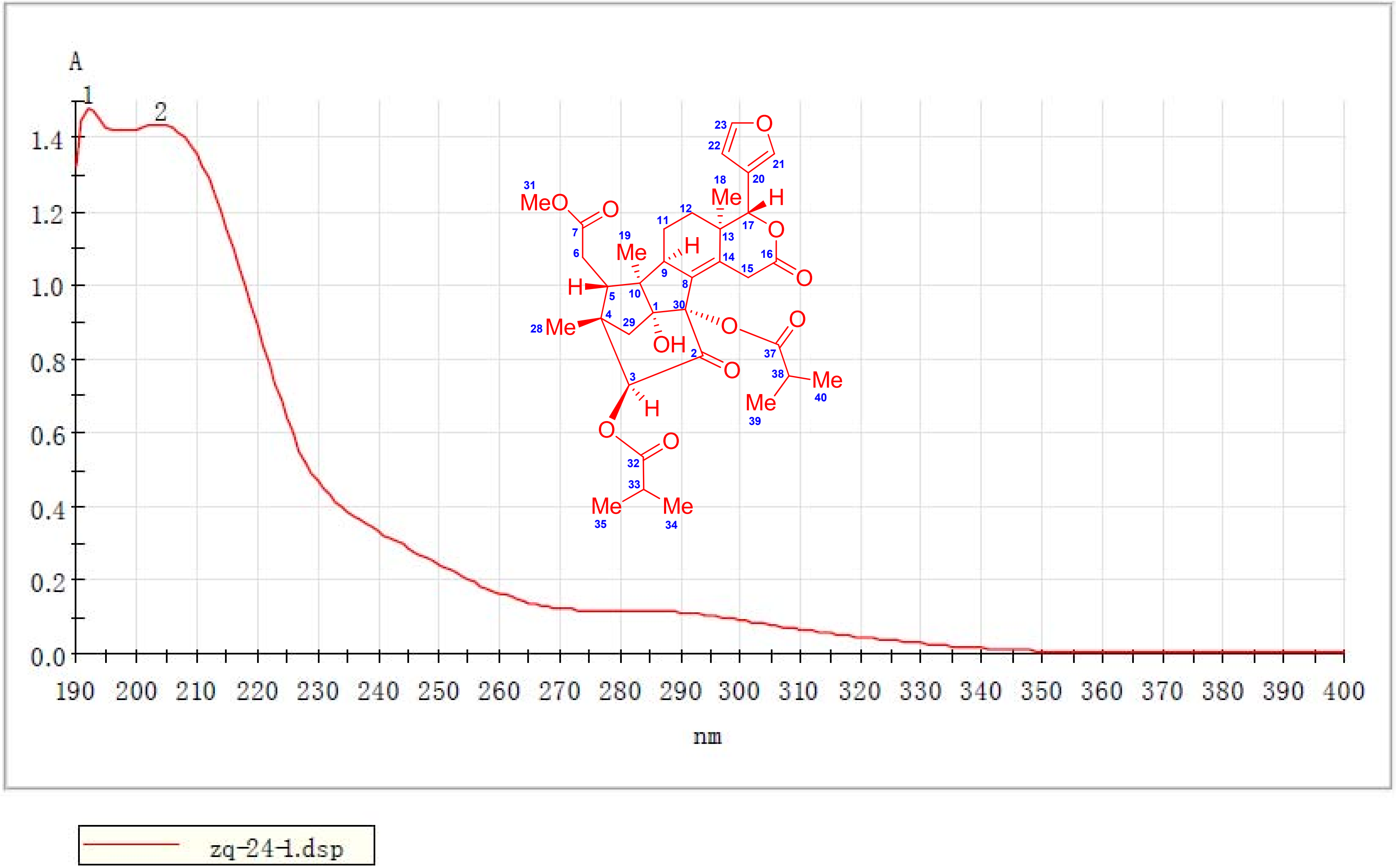

^{1}H NMR (400 MHz) spectrum of Krishnolide D (**4**) in $CDCl_3$

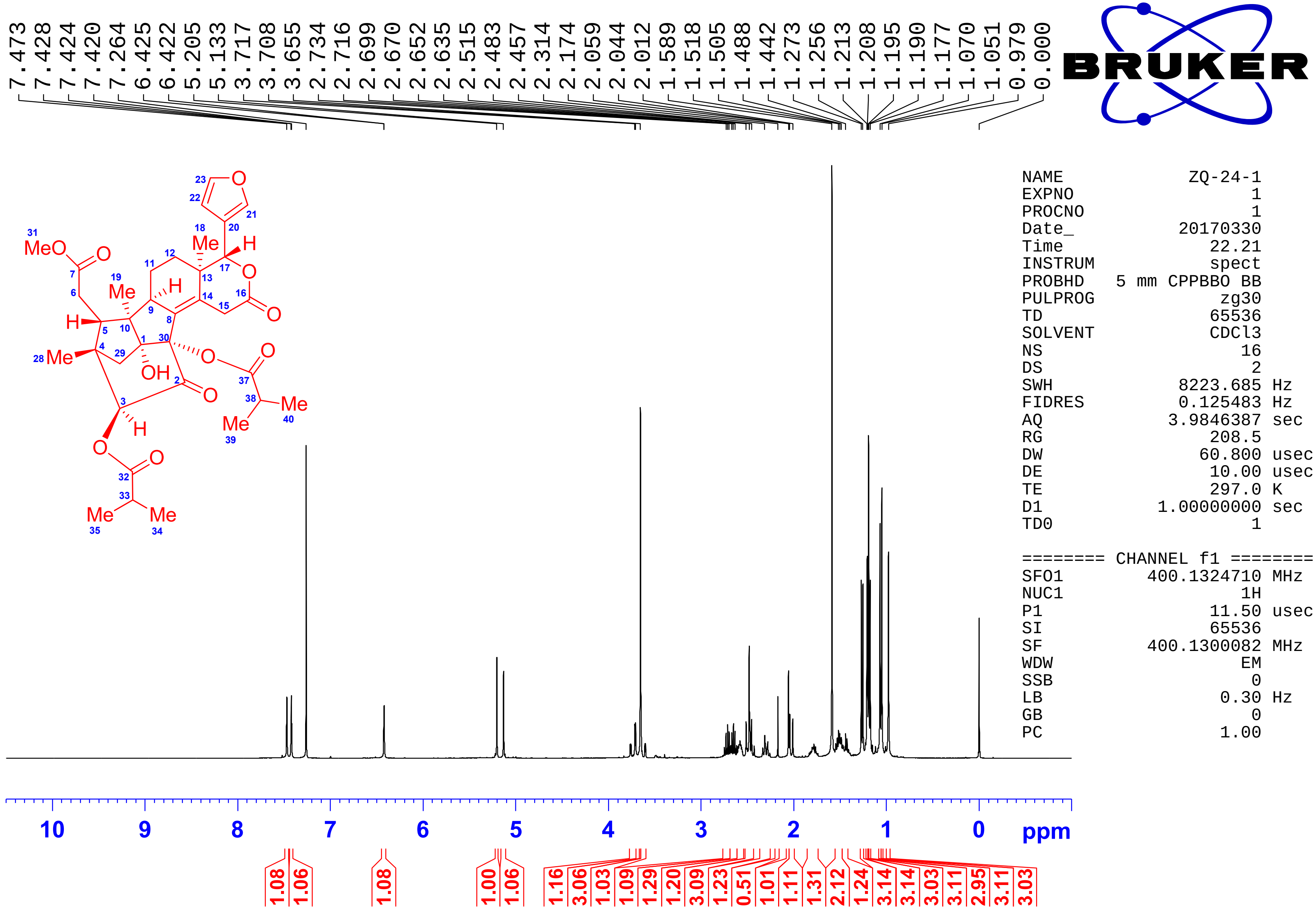

^{1}H NMR (400 MHz) spectrum of Krishnolide D (**4**) in $CDCl_3$

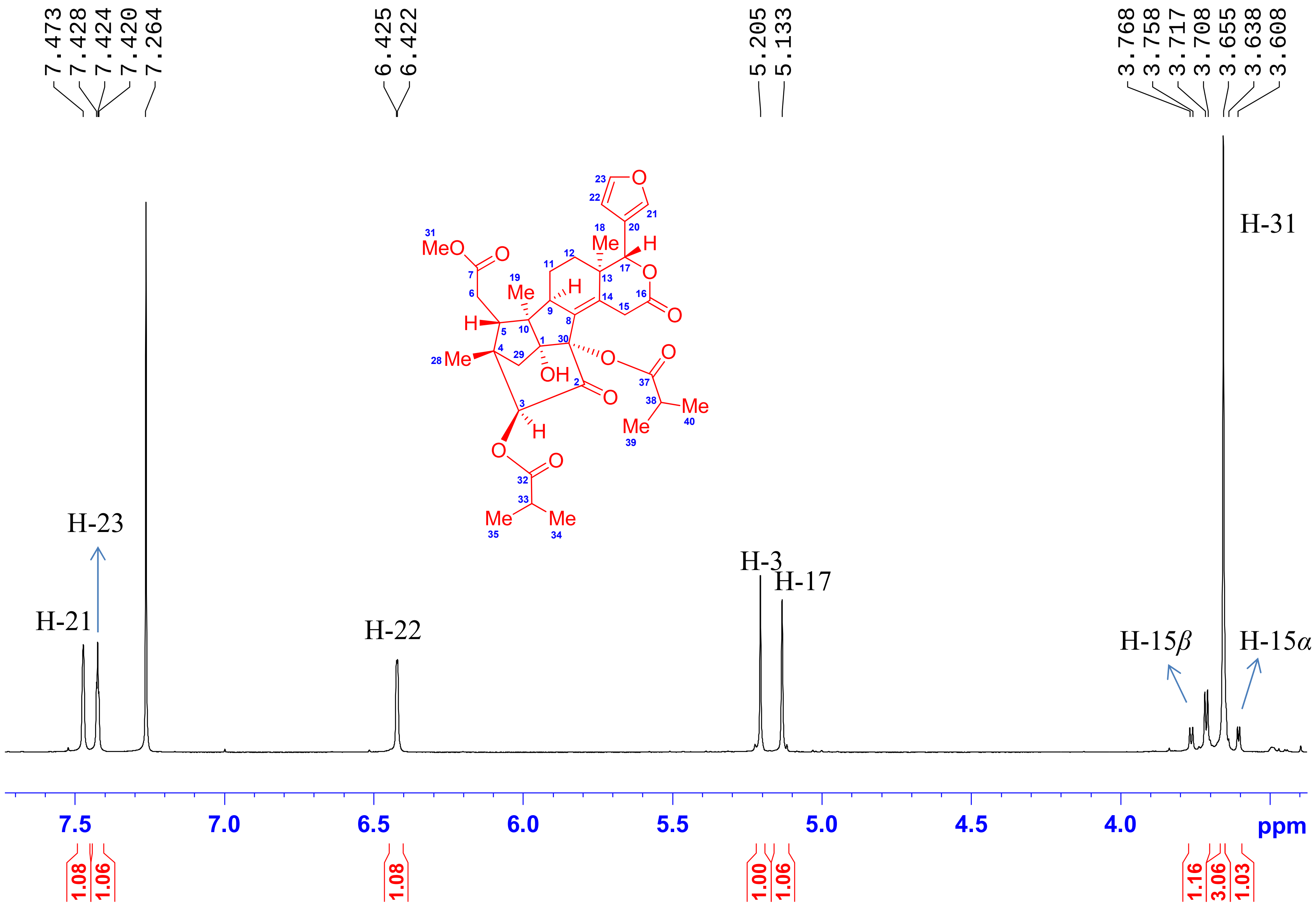

^{1}H NMR (400 MHz) spectrum of Krishnolide D (**4**) in $CDCl_3$

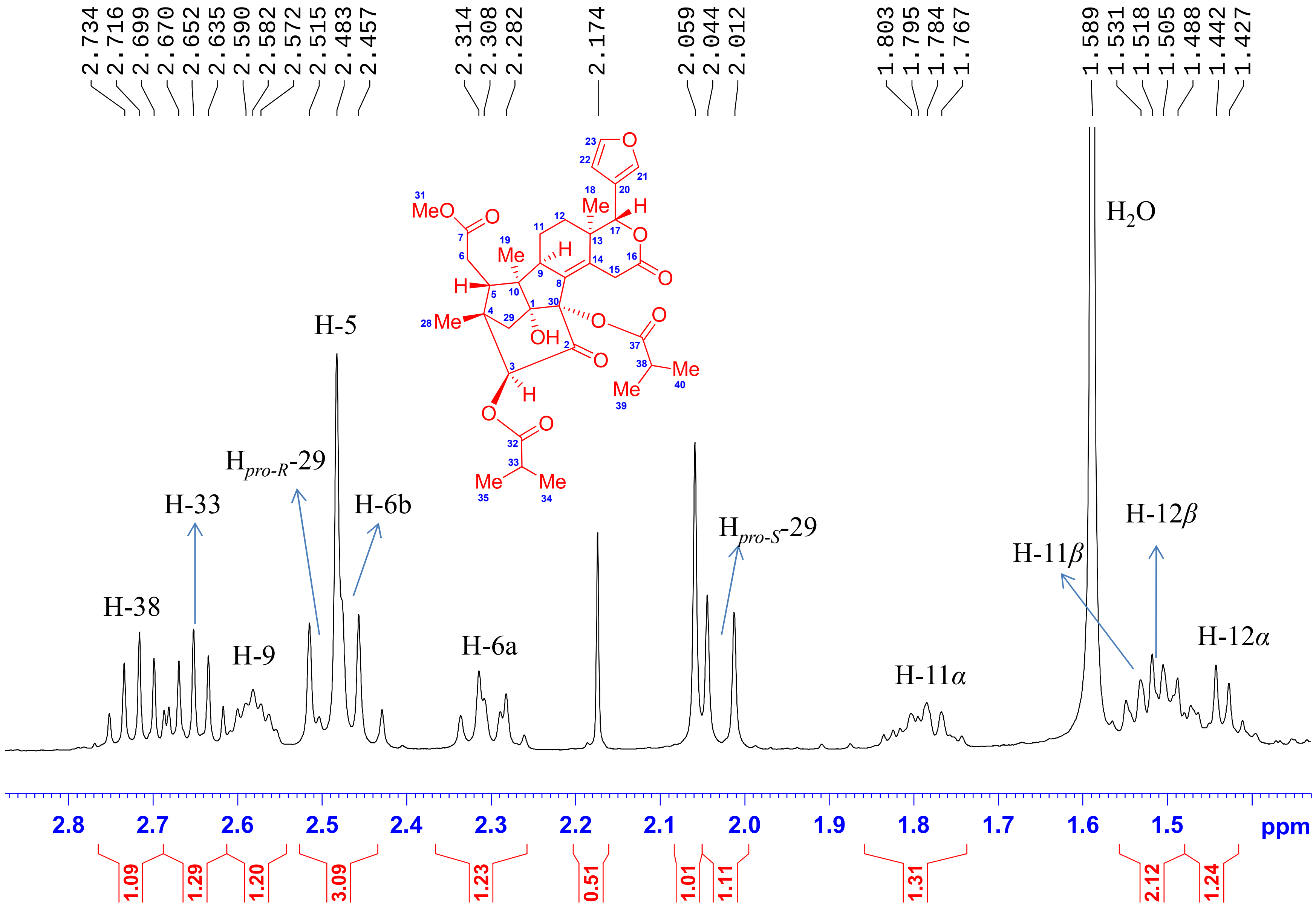

^{1}H NMR (400 MHz) spectrum of Krishnolide D (**4**) in $CDCl_3$

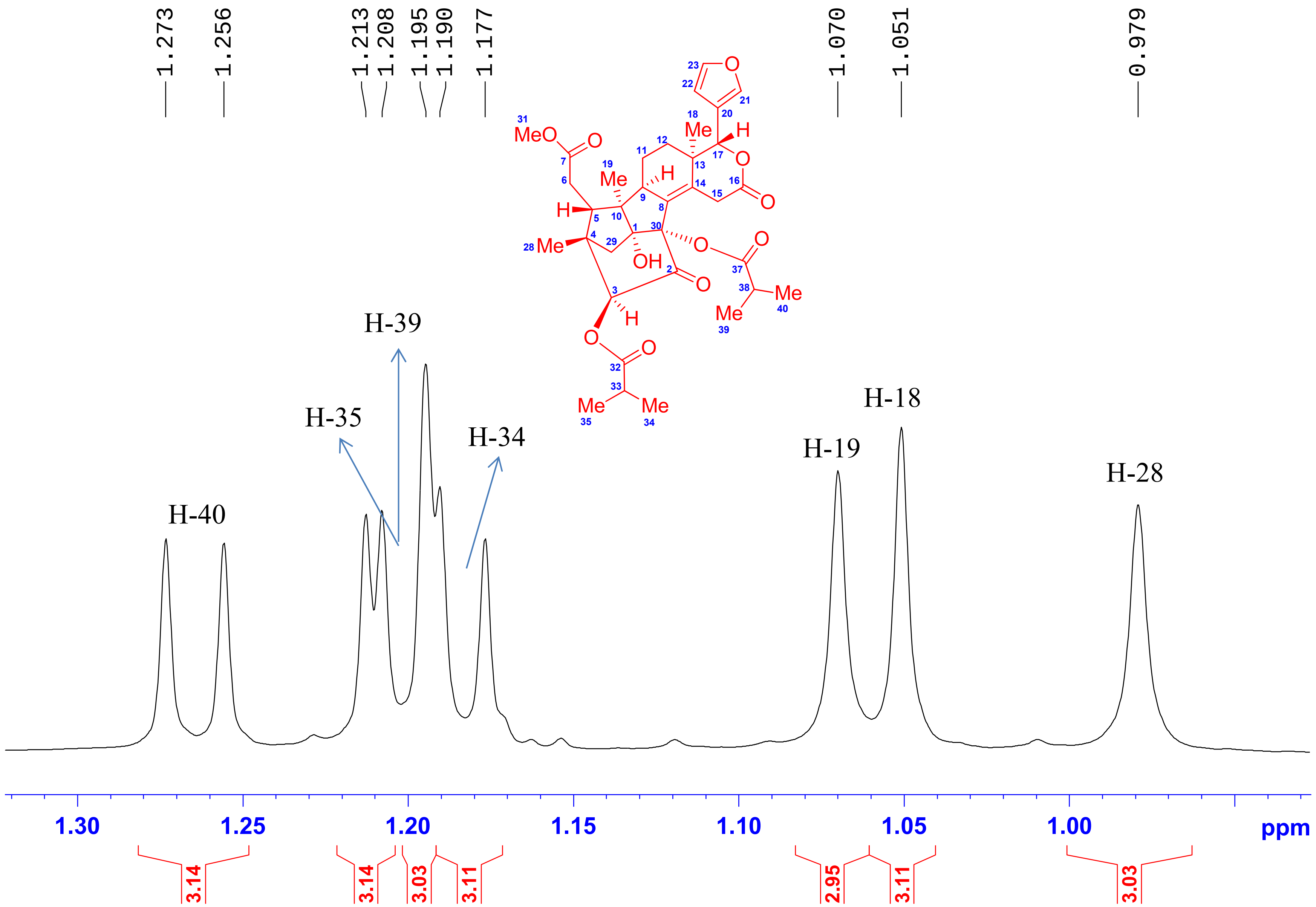

^{13}C NMR (100 MHz) spectrum of Krishnolide D (**4**) in $CDCl_3$

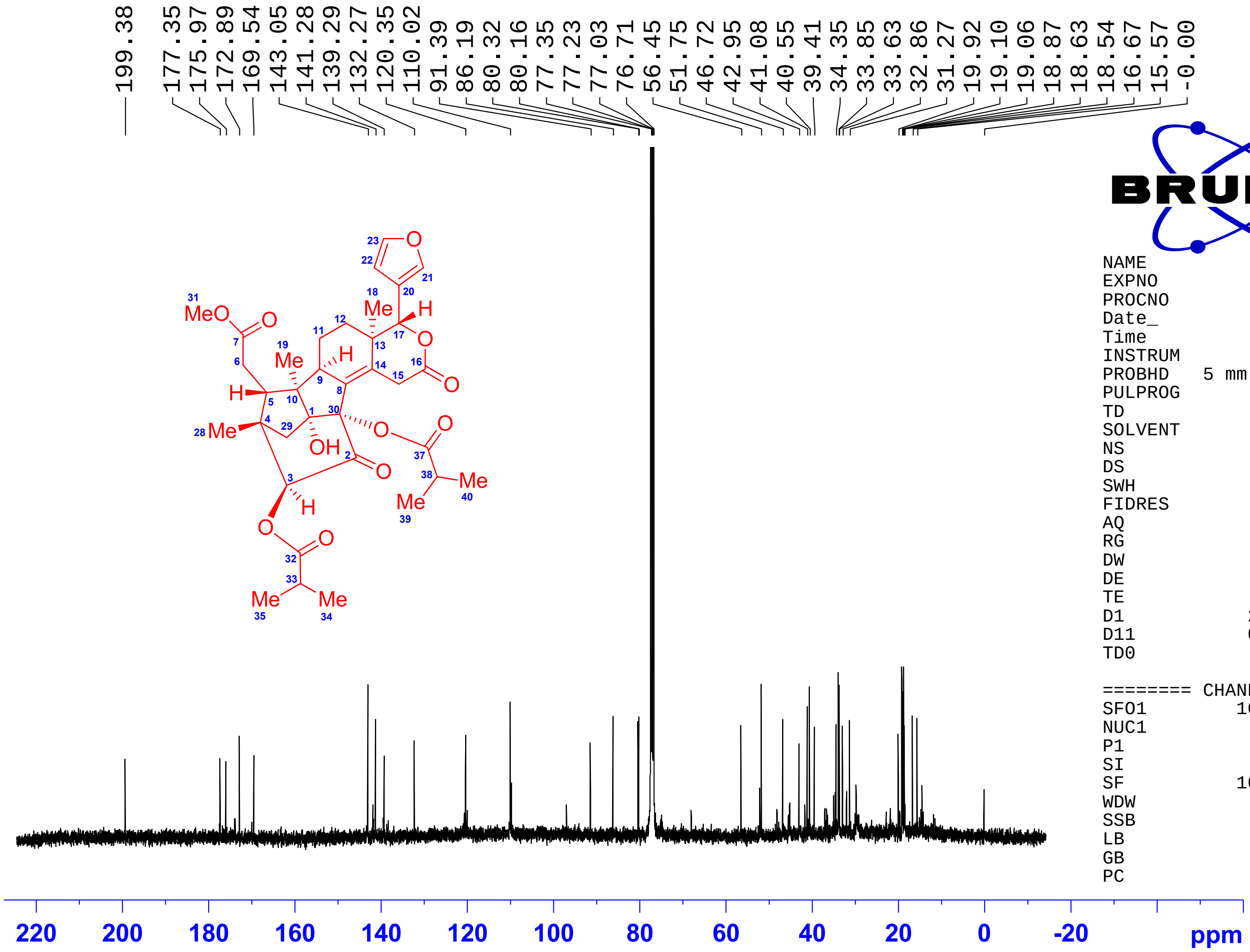

NAME	ZQ-24-1
EXPNO	2
PROCNO	1
Date_	20170331
Time	8.12
INSTRUM	spect
PROBHD	5 mm CPPBBO BB
PULPROG	zgpg30
TD	65536
SOLVENT	CDCl3
NS	1024
DS	4
SWH	24038.461 Hz
FIDRES	0.366798 Hz
AQ	1.3631988 sec
RG	147.94
DW	20.800 usec
DE	18.00 usec
TE	297.0 K
D1	2.00000000 sec
D11	0.03000000 sec
TD0	1

======== CHANNEL f1 ========

SFO1	100.6233324 MHz
NUC1	13C
P1	10.00 usec
SI	32768
SF	100.6127690 MHz
WDW	EM
SSB	0
LB	1.00 Hz
GB	0
PC	1.40

^{13}C NMR (100 MHz) spectrum of Krishnolide D (**4**) in $CDCl_3$

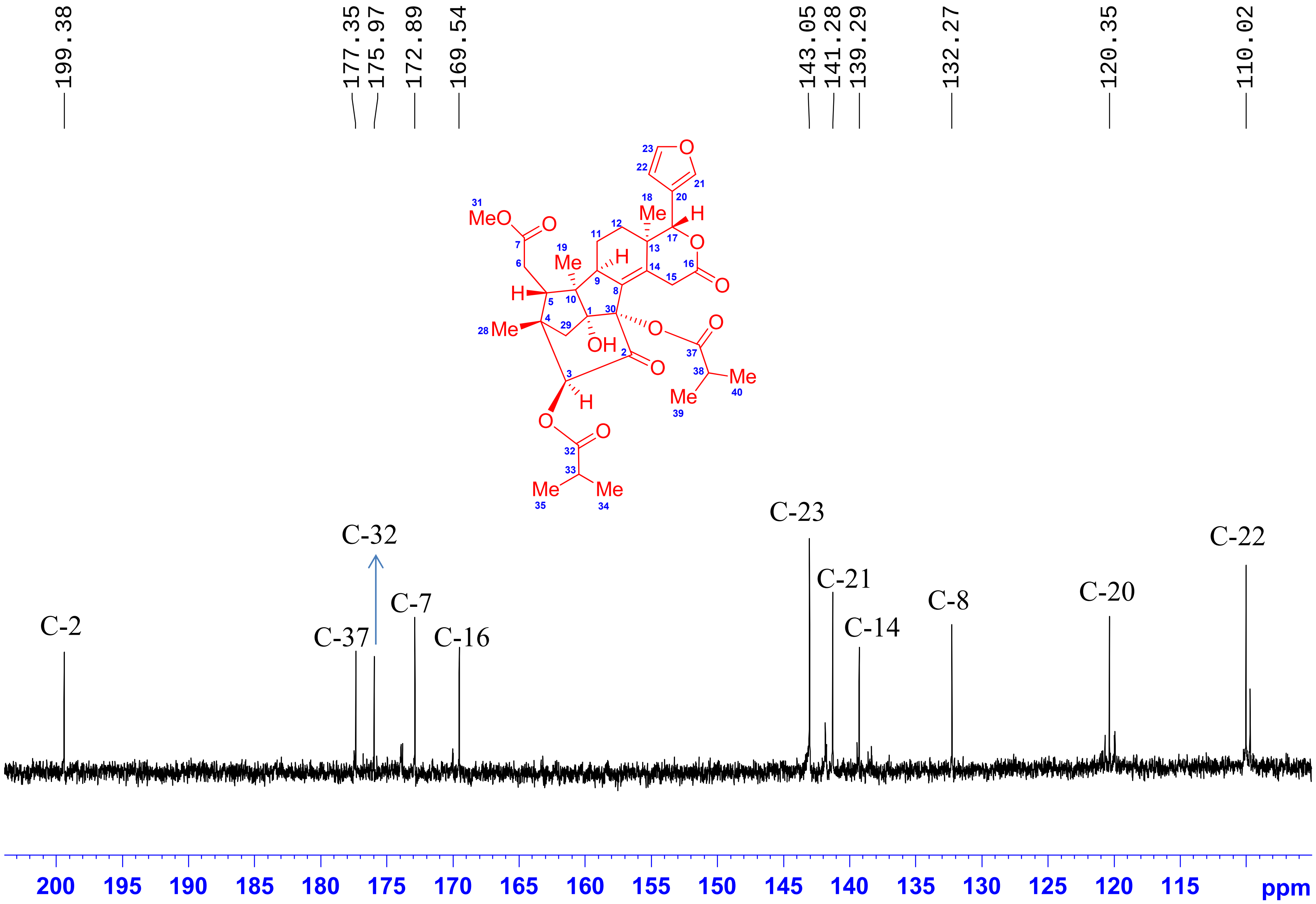

^{13}C NMR (100 MHz) spectrum of Krishnolide D (**4**) in $CDCl_3$

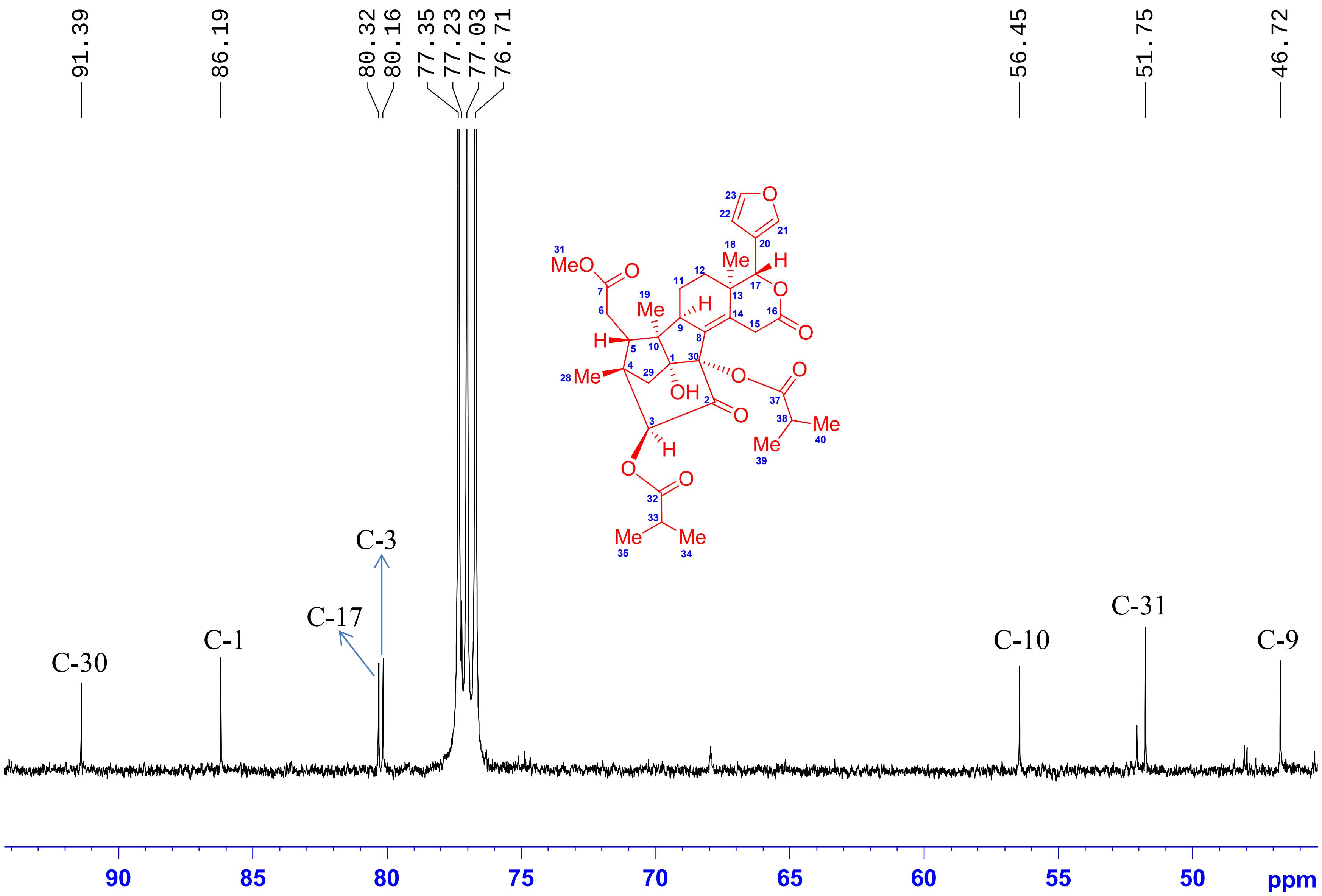

^{13}C NMR (100 MHz) spectrum of Krishnolide D (**4**) in $CDCl_3$

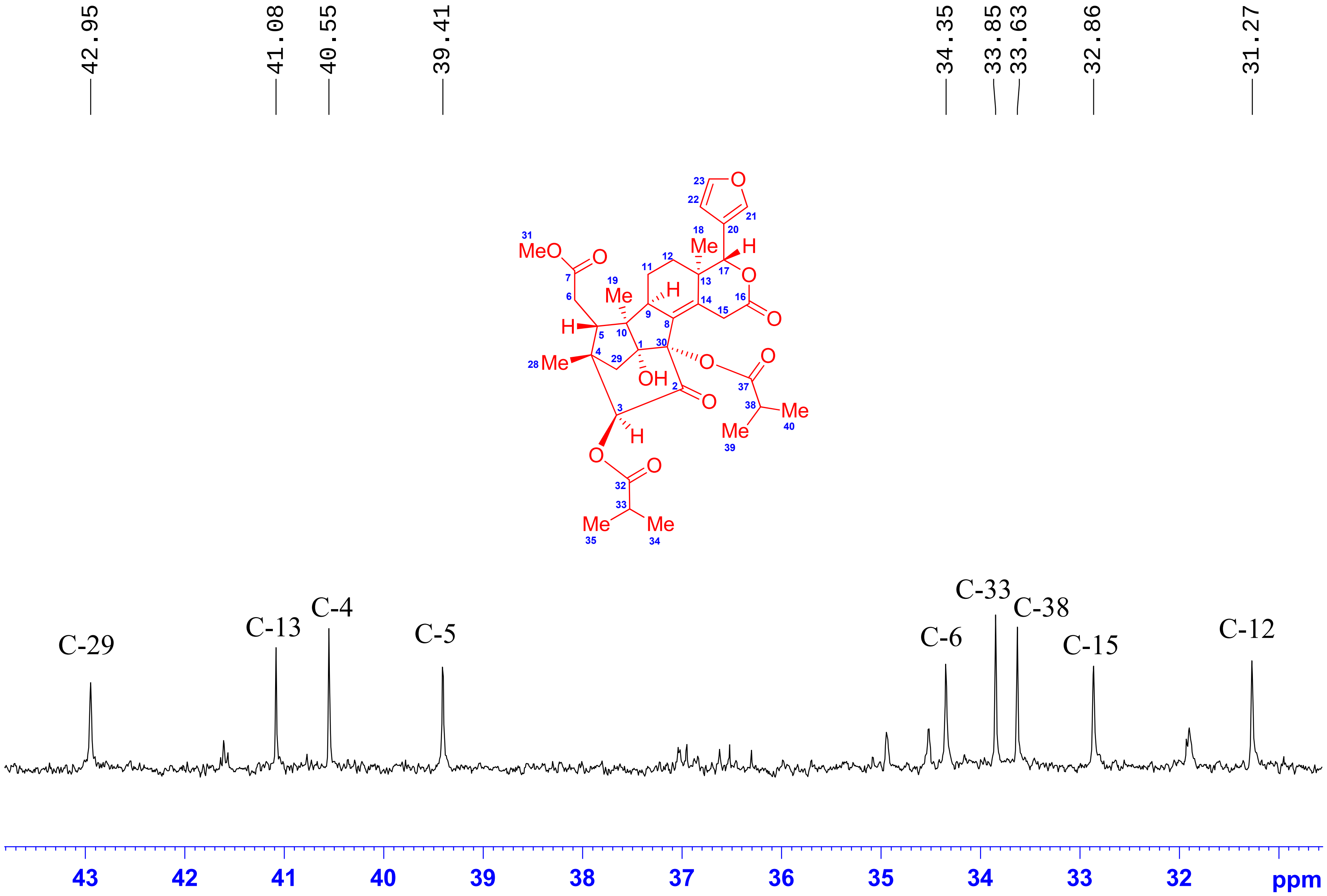

^{13}C NMR (100 MHz) spectrum of Krishnolide D (**4**) in $CDCl_3$

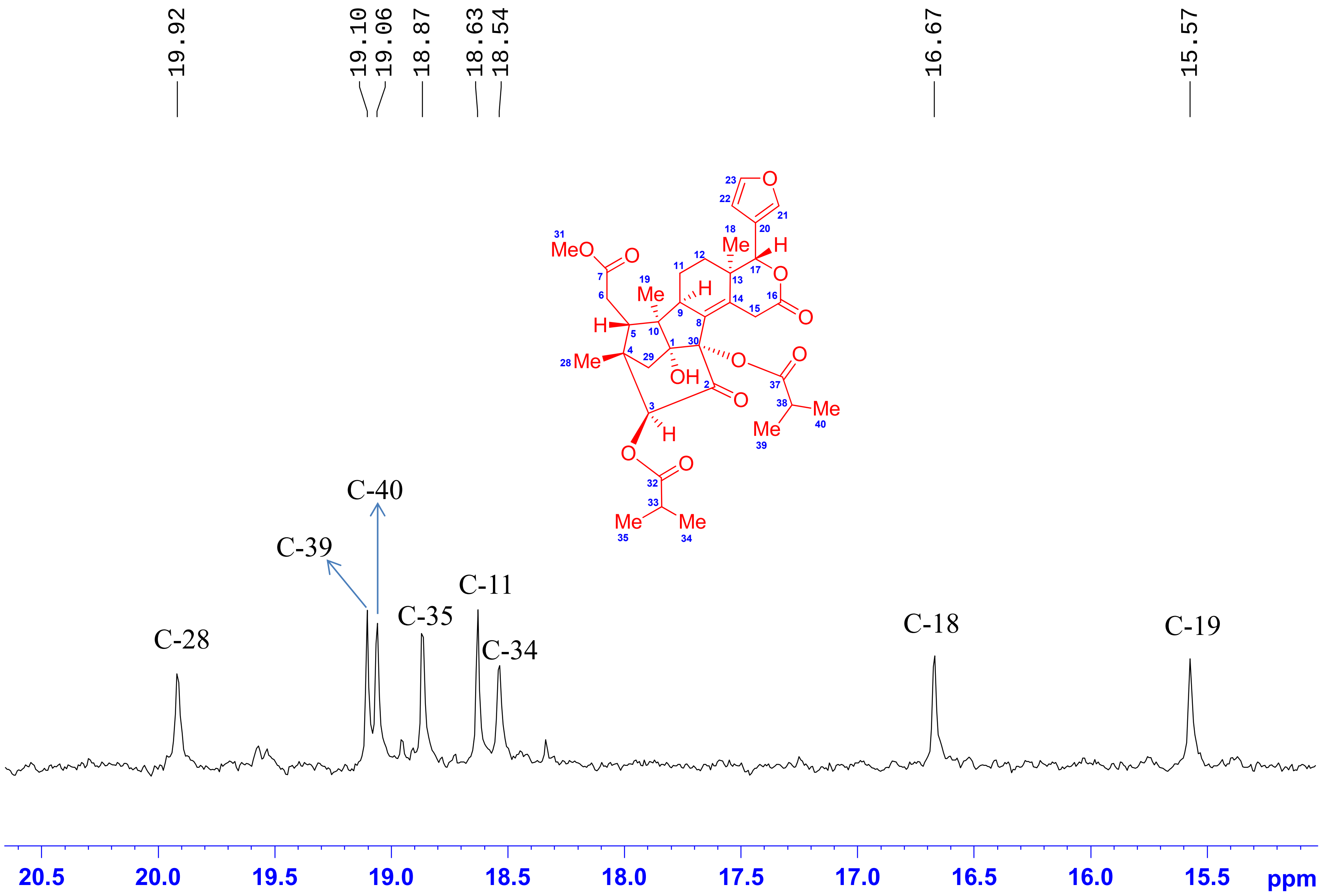

DEPT 135 spectrum of Krishnolide D (**4**) in $CDCl_3$

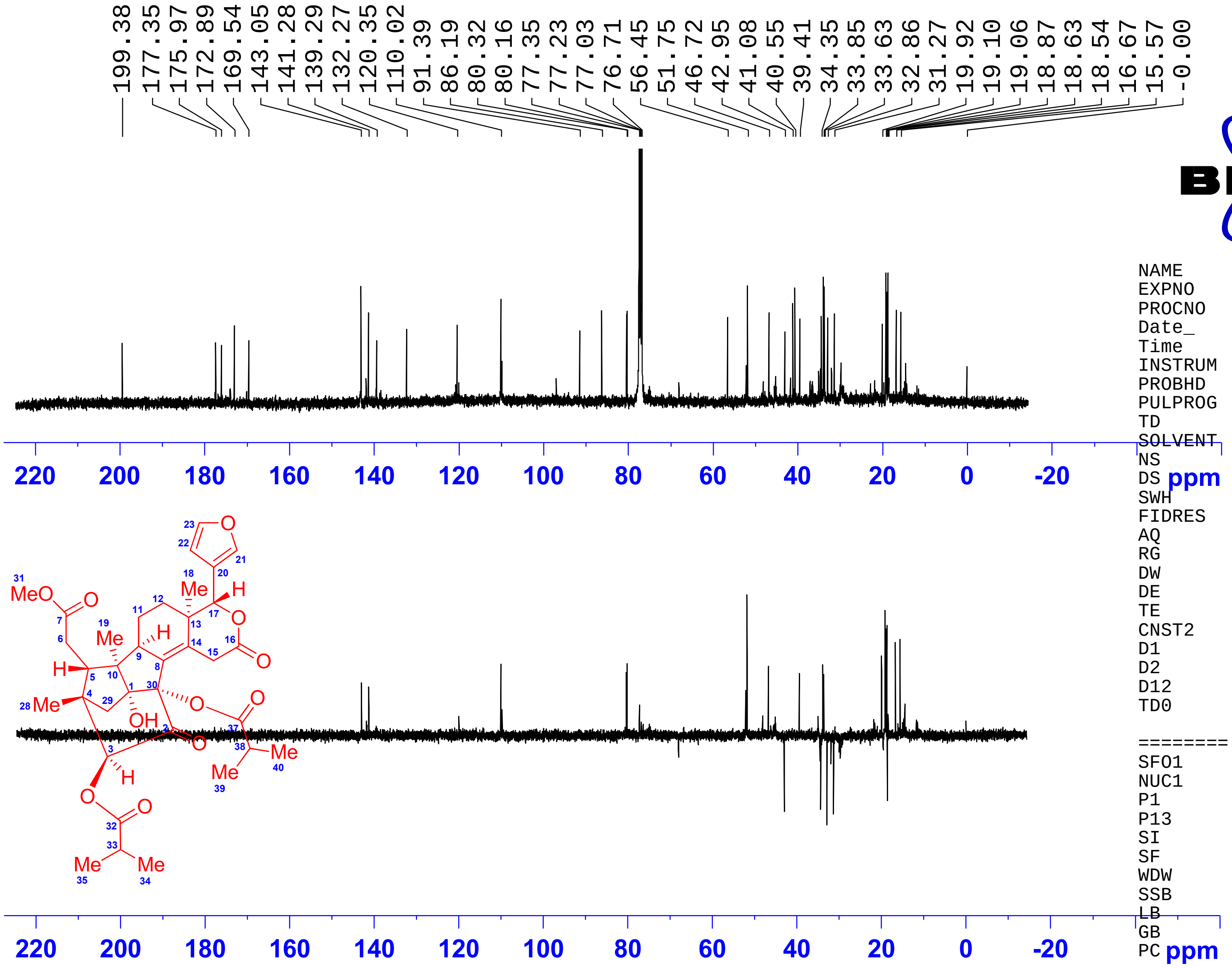

NAME	ZQ-24-1
EXPNO	3
PROCNO	1
Date_	20170331
Time	8.30
INSTRUM	spect
PROBHD	5 mm CPPBBO BB
PULPROG	deptsp135
TD	65536
SOLVENT	CDCl3
NS	300
DS	4
SWH	24038.461 Hz
FIDRES	0.366798 Hz
AQ	1.3631988 sec
RG	130.26
DW	20.800 usec
DE	18.00 usec
TE	297.0 K
CNST2	145.0000000
D1	2.00000000 sec
D2	0.00344828 sec
D12	0.00002000 sec
TD0	1
======== CHANNEL f1 ========	
SFO1	100.6233324 MHz
NUC1	13C
P1	10.00 usec
P13	2000.00 usec
SI	32768
SF	100.6127690 MHz
WDW	EM
SSB	0
LB	1.00 Hz
GB	0
PC	1.40

DEPT 135 spectrum of Krishnolide D (**4**) in $CDCl_3$

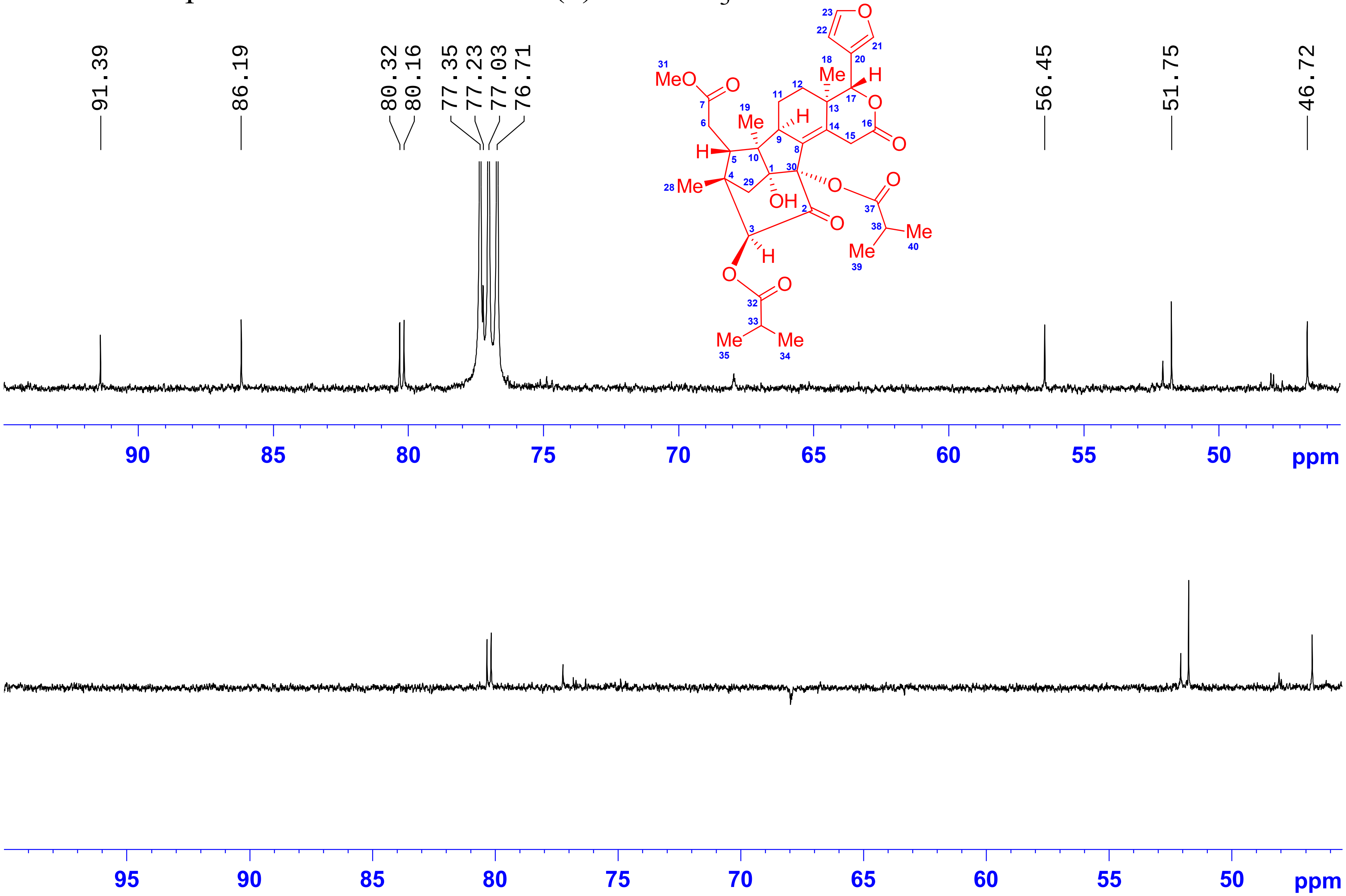

DEPT 135 spectrum of Krishnolide D (**4**) in $CDCl_3$

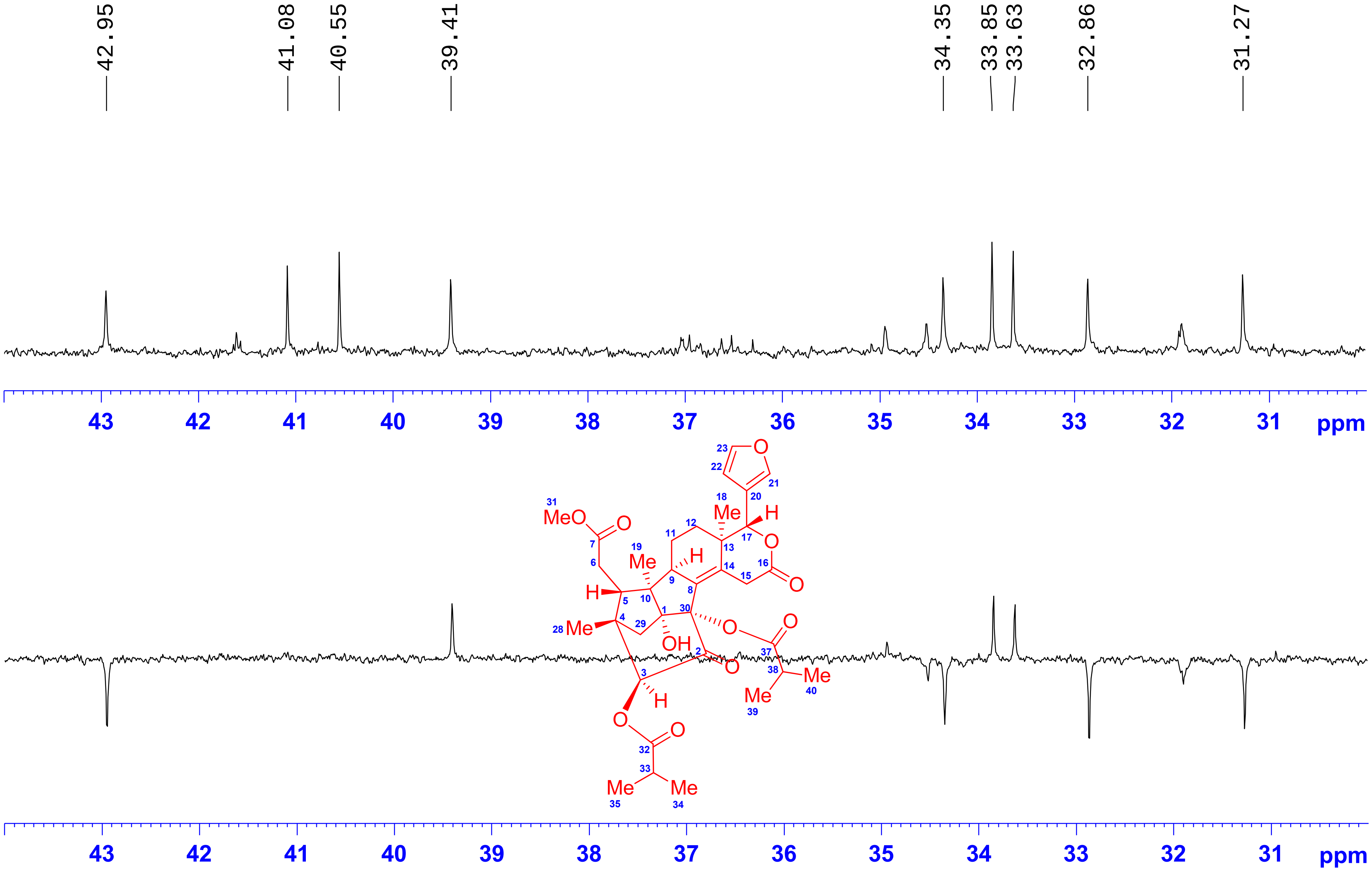

DEPT 135 spectrum of Krishnolide D (**4**) in $CDCl_3$

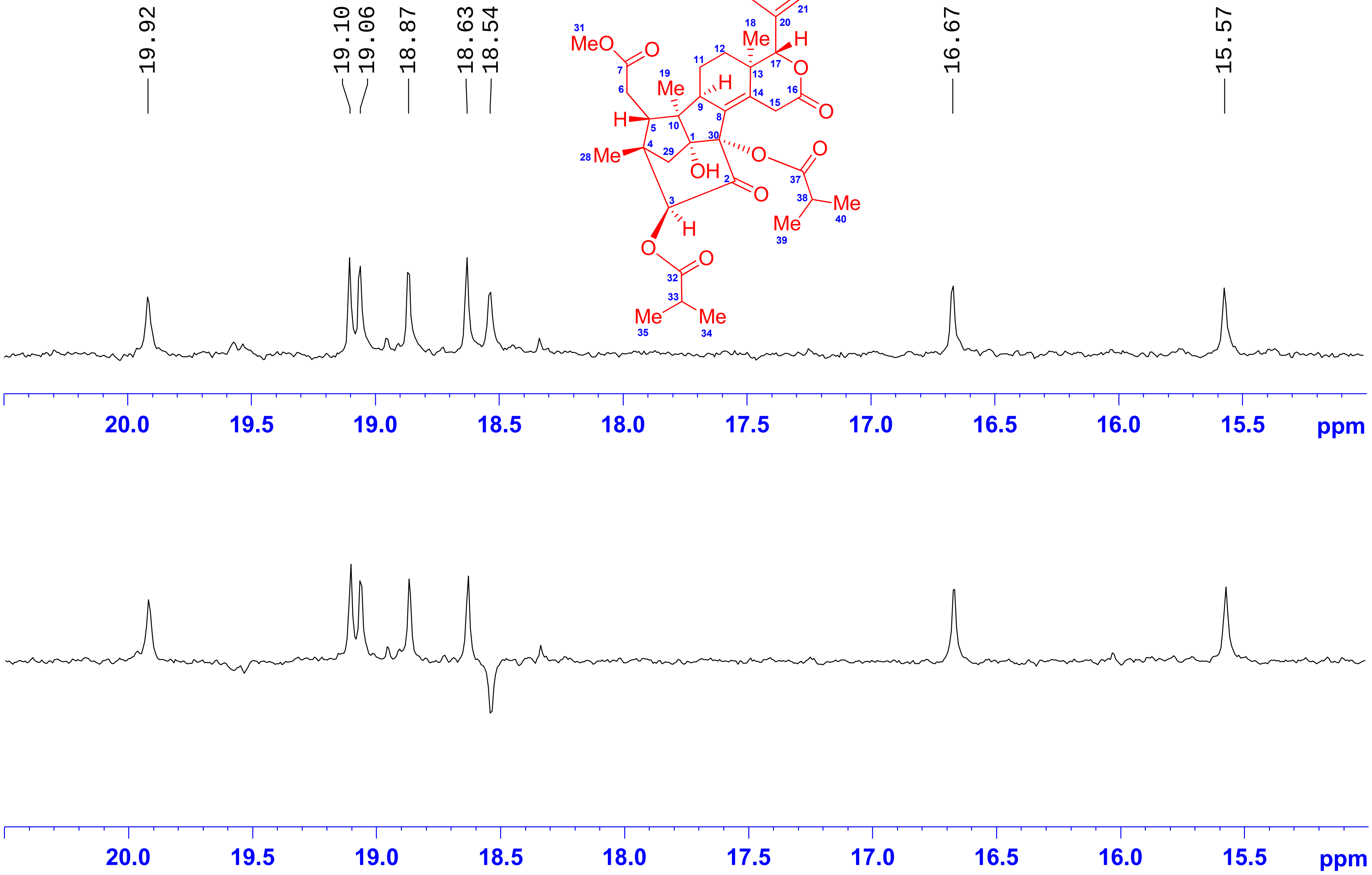

^{1}H-^{1}H COSY spectrum of Krishnolide D (**4**) in $CDCl_3$

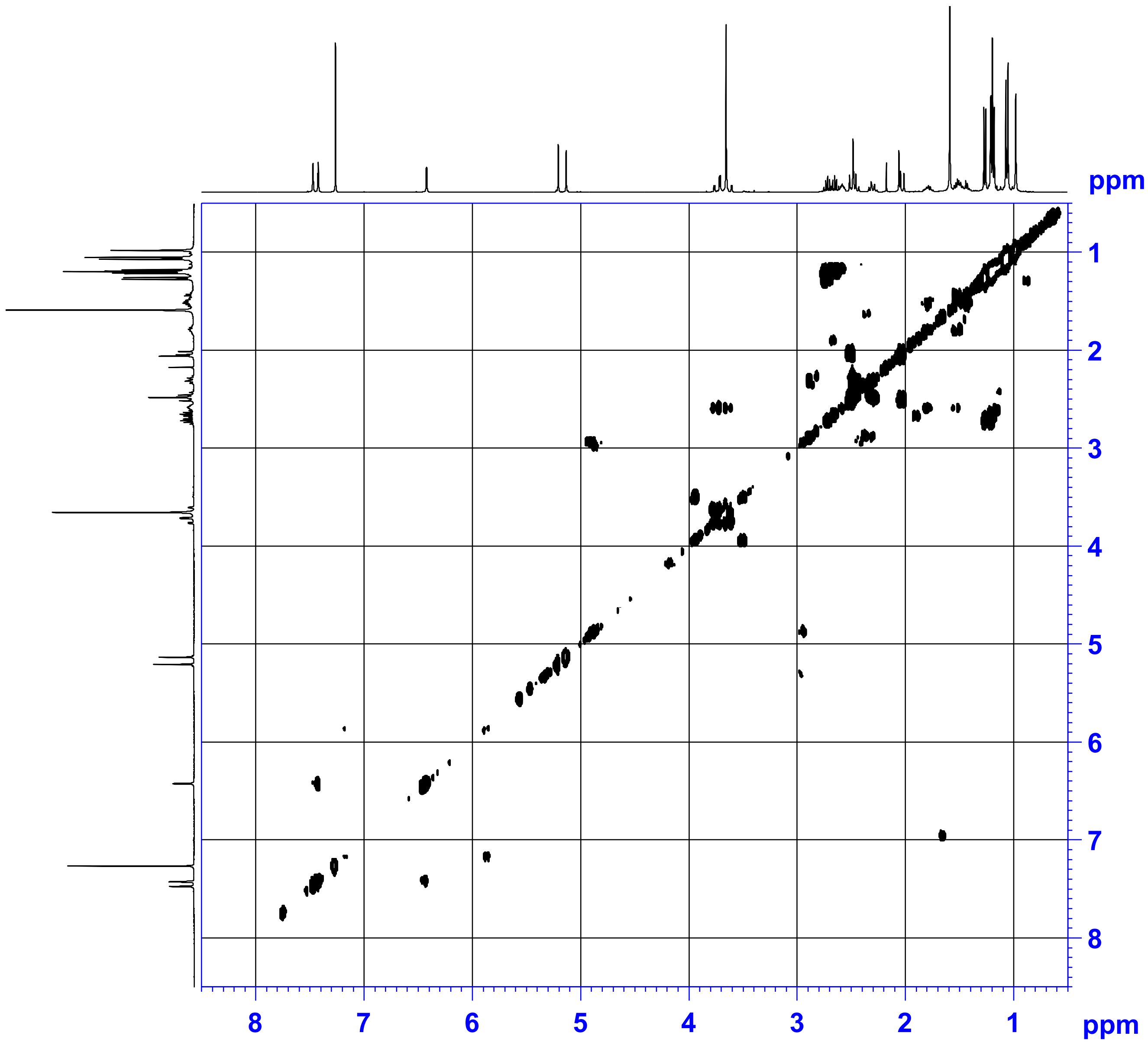

NAME	ZQ-24-1	
EXPNO	4	
PROCNO	1	
Date_	20170401	
Time	8.30	
INSTRUM	spect	
PROBHD	5 mm CPPBBO BB	
PULPROG	cosygpppqf	
TD	2048	
SOLVENT	CDCl3	
NS	8	
DS	8	
SWH	3906.250	Hz
FIDRES	1.907349	Hz
AQ	0.2621940	sec
RG	208.5	
DW	128.000	usec
DE	10.00	usec
TE	297.0	K
D0	0.00000300	sec
D1	1.89678097	sec
D11	0.03000000	sec
D12	0.00002000	sec
D13	0.00000400	sec
D16	0.00020000	sec
IN0	0.00025600	sec
======== CHANNEL f1 ========		
SFO1	400.1318006	MHz
NUC1	1H	
P0	11.50	usec
P1	11.50	usec
P17	2500.00	usec
ND0	1	
TD	128	
SFO1	400.1318	MHz
FIDRES	30.517578	Hz
SW	9.762	ppm
FnMODE	QF	
SI	1024	
SF	400.1300050	MHz
WDW	QSINE	
SSB	0	
LB	0.00	Hz
GB	0	
PC	1.40	
SI	1024	
MC2	QF	
SF	400.1300050	MHz
WDW	QSINE	
SSB	0	
LB	0.00	Hz
GB	0	

^{1}H-^{1}H COSY spectrum of Krishnolide D (**4**) in $CDCl_3$

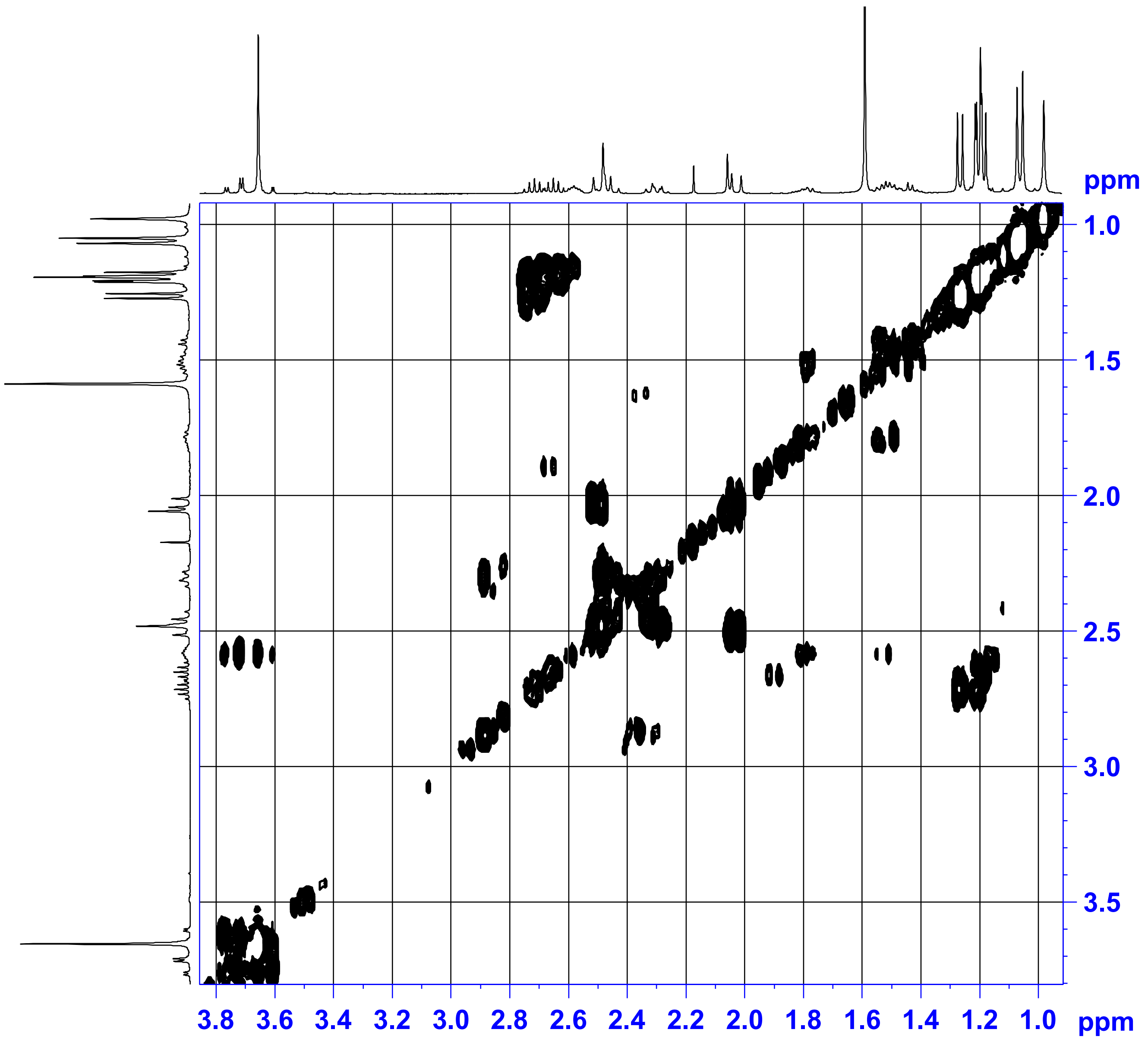

^{1}H-^{1}H COSY spectrum of Krishnolide D (**4**) in $CDCl_3$

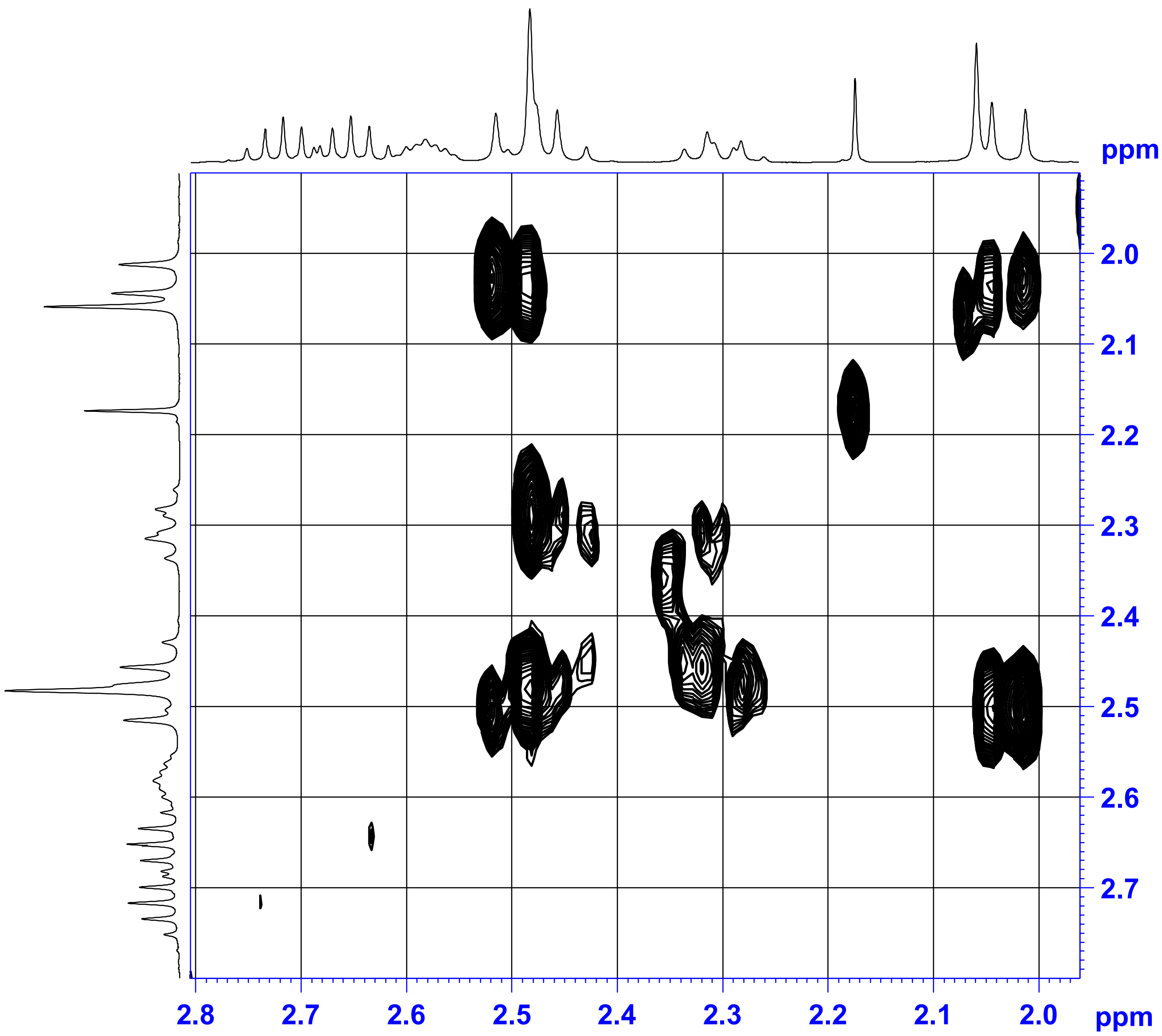

^{1}H-^{1}H COSY spectrum of Krishnolide D (**4**) in $CDCl_3$

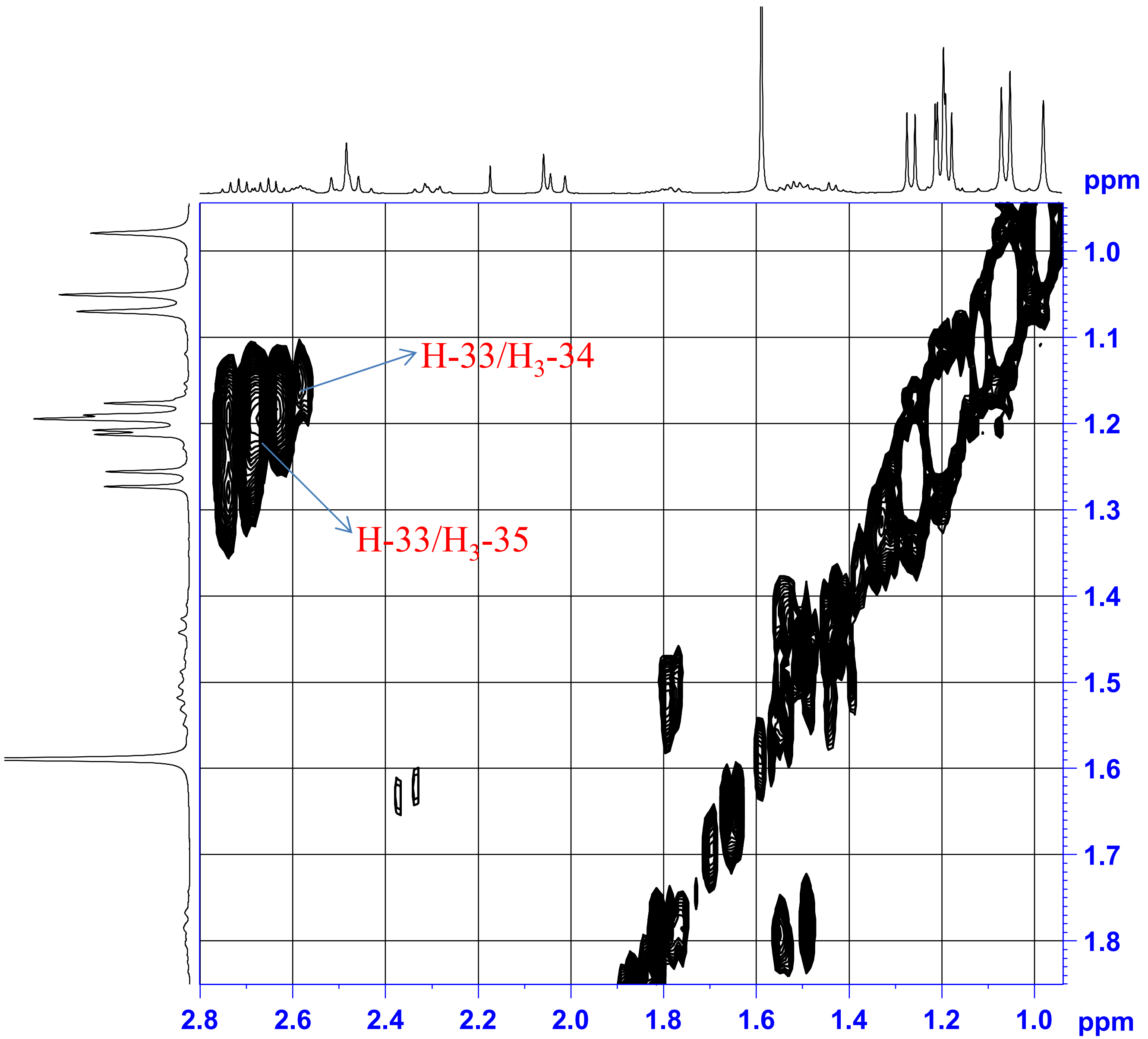

^1H-^1H COSY spectrum of Krishnolide D (**4**) in $CDCl_3$

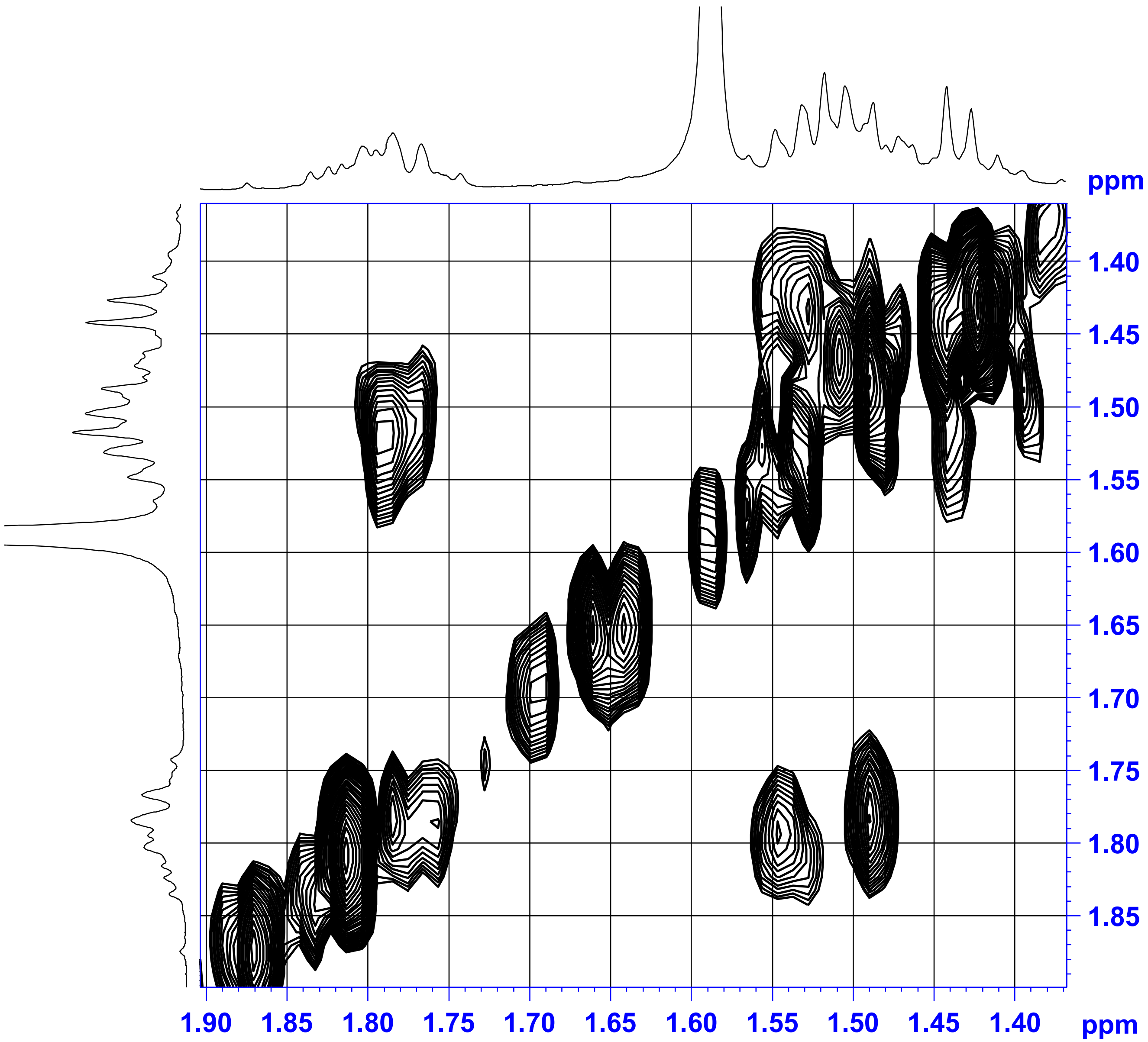

HSQC spectrum of Krishnolide D (**4**) in $CDCl_3$

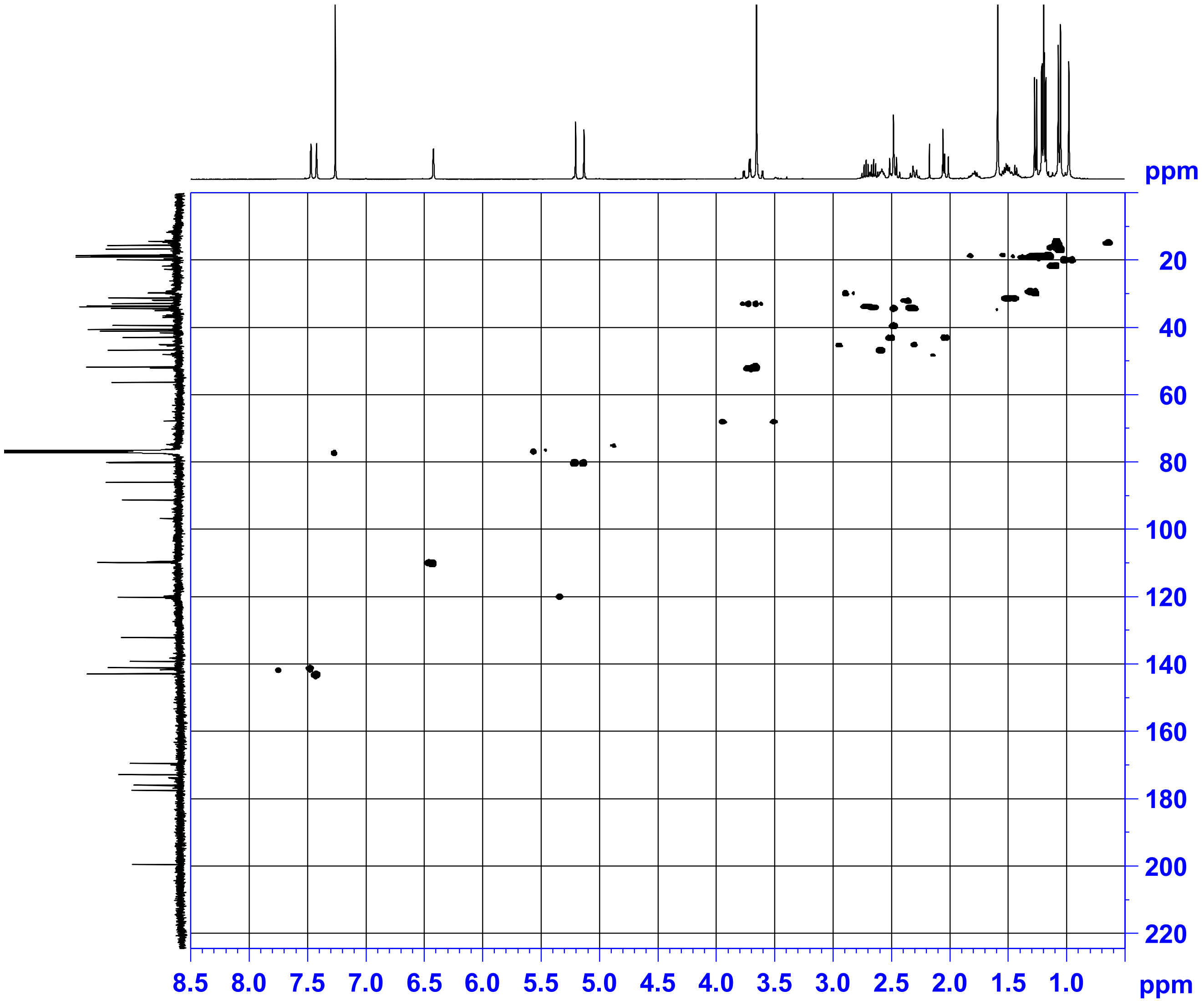

NAME	ZQ-24-1
EXPNO	5
PROCNO	1
Date_	20170401
Time	9.10
INSTRUM	spect
PROBHD	5 mm CPPBBO BB
PULPROG	hsqcetgpsi2
TD	1024
SOLVENT	CDCl3
NS	16
DS	16
SWH	4302.926 Hz
FIDRES	4.202076 Hz
AQ	0.1190388 sec
RG	208.5
DW	116.200 usec
DE	10.00 usec
TE	297.0 K
CNST2	145.0000000
D0	0.00000300 sec
D1	1.46497905 sec
D4	0.00172414 sec
D11	0.03000000 sec
D16	0.00020000 sec
D24	0.00086207 sec
IN0	0.00002080 sec
ZGOPTNS	

======== CHANNEL f1 ========

SFO1	400.1320007 MHz
NUC1	1H
P1	11.50 usec
P2	23.00 usec
P28	0.00 usec
ND0	2
TD	256
SFO1	100.6233 MHz
FIDRES	93.900238 Hz
SW	238.896 ppm
FnMODE	Echo-Antiecho
SI	1024
SF	400.1300050 MHz
WDW	QSINE
SSB	2
LB	0.00 Hz
GB	0
PC	1.40
SI	1024
MC2	echo-antiecho
SF	100.6127593 MHz
WDW	QSINE
SSB	2
LB	0.00 Hz
GB	0

HSQC spectrum of Krishnolide D (**4**) in $CDCl_3$

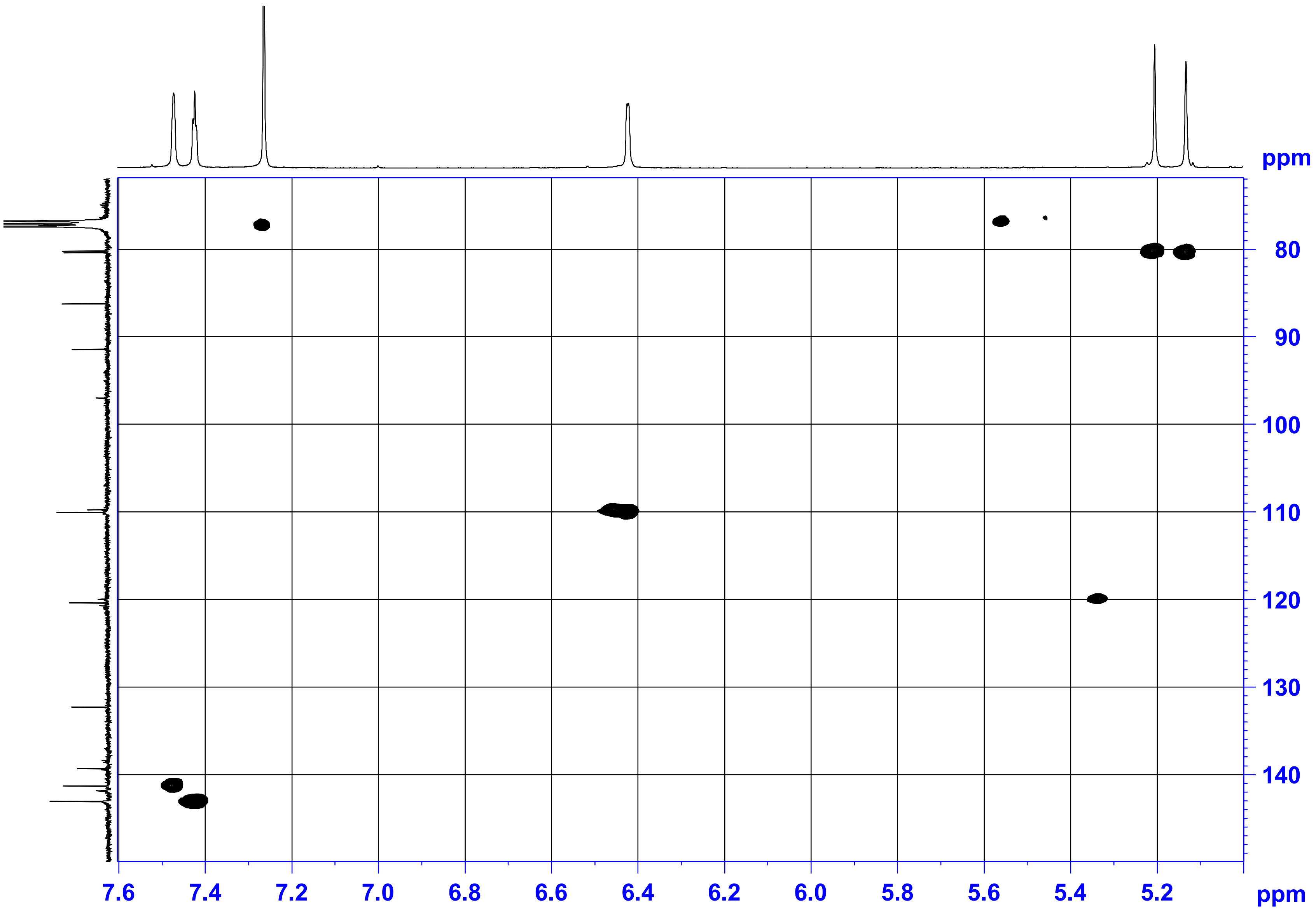

HSQC spectrum of Krishnolide D (**4**) in $CDCl_3$

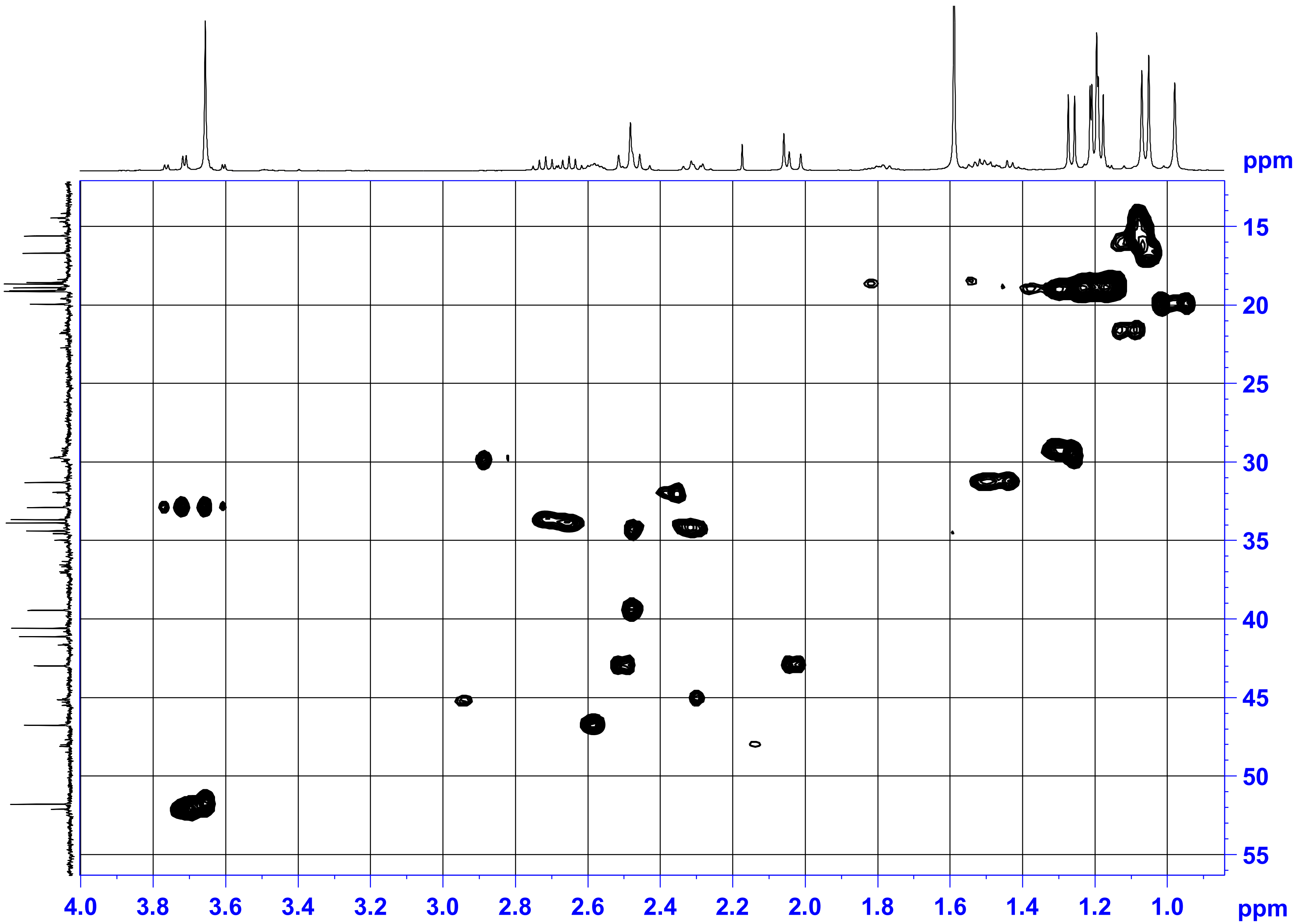

HSQC spectrum of Krishnolide D (**4**) in $CDCl_3$

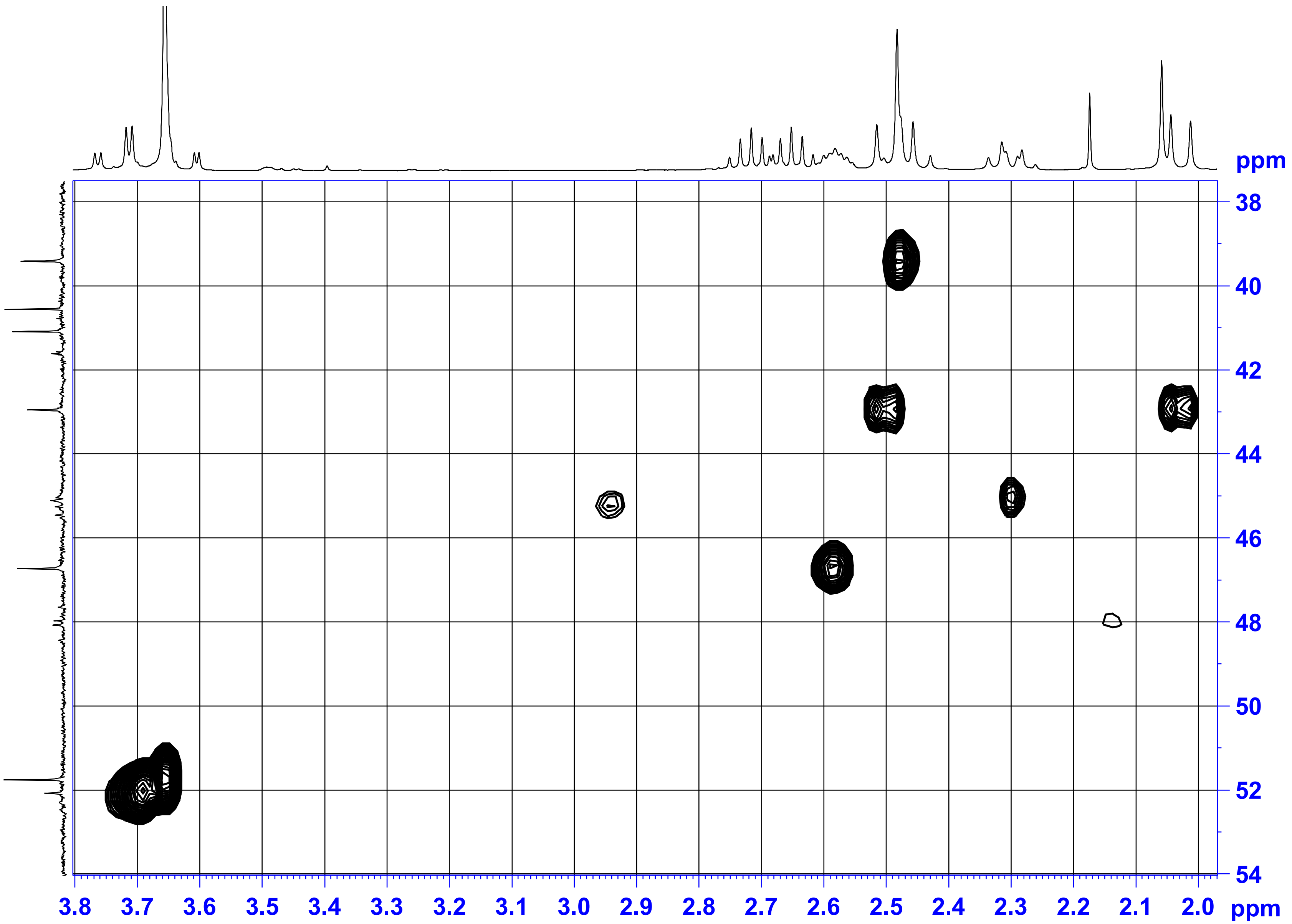

HSQC spectrum of Krishnolide D (**4**) in $CDCl_3$

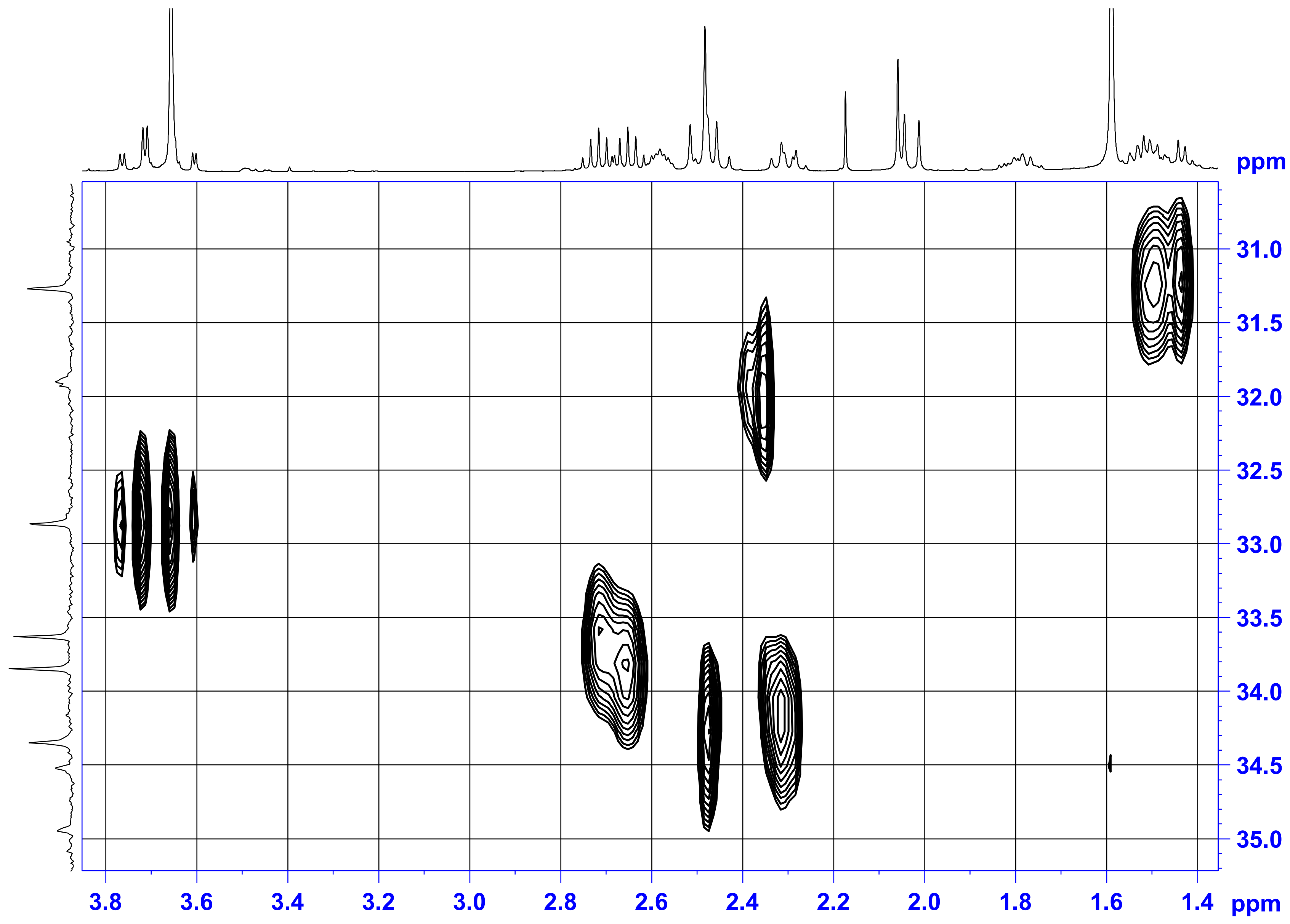

HSQC spectrum of Krishnolide D (**4**) in $CDCl_3$

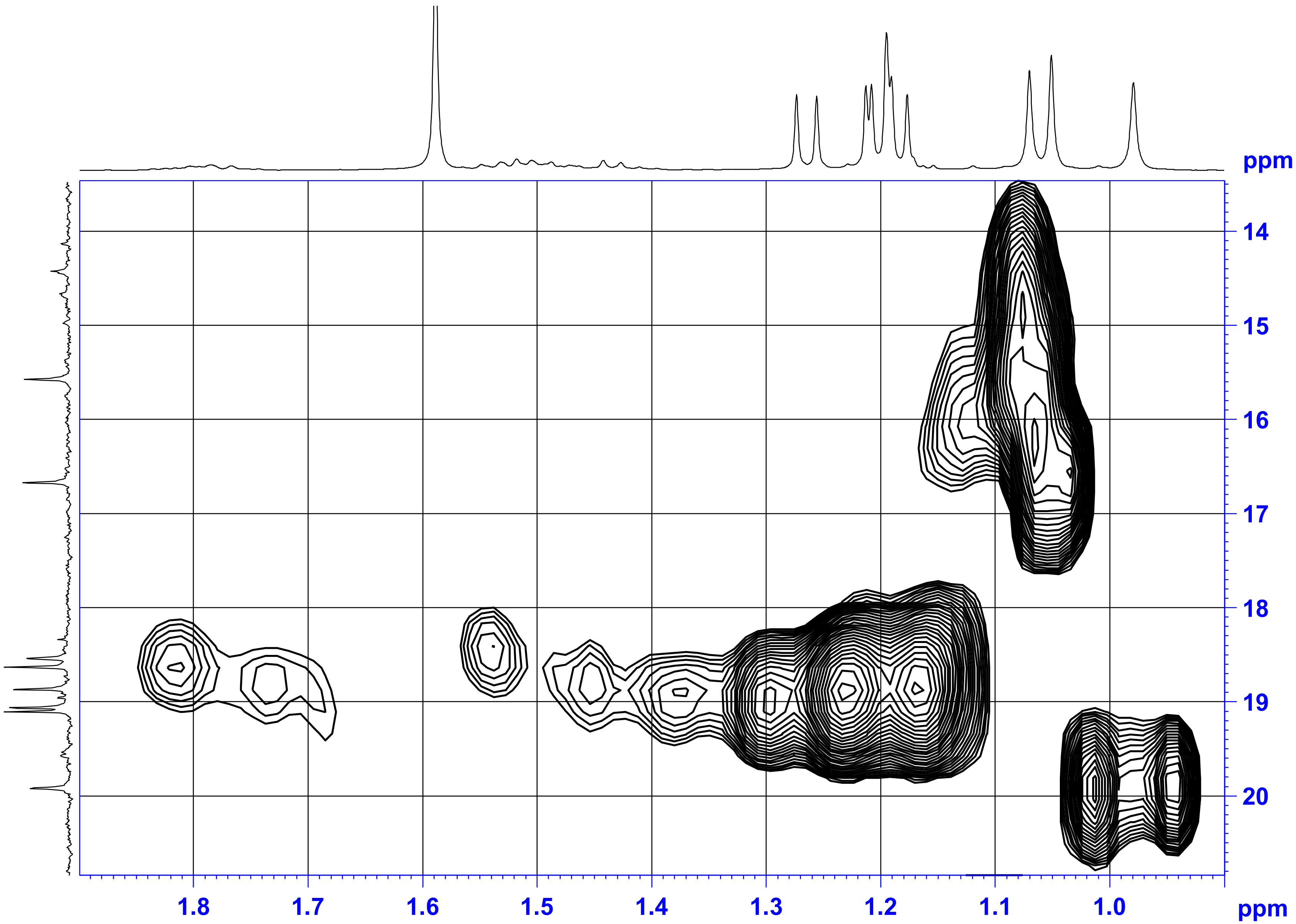

HMBC spectrum of Krishnolide D (**4**) in $CDCl_3$

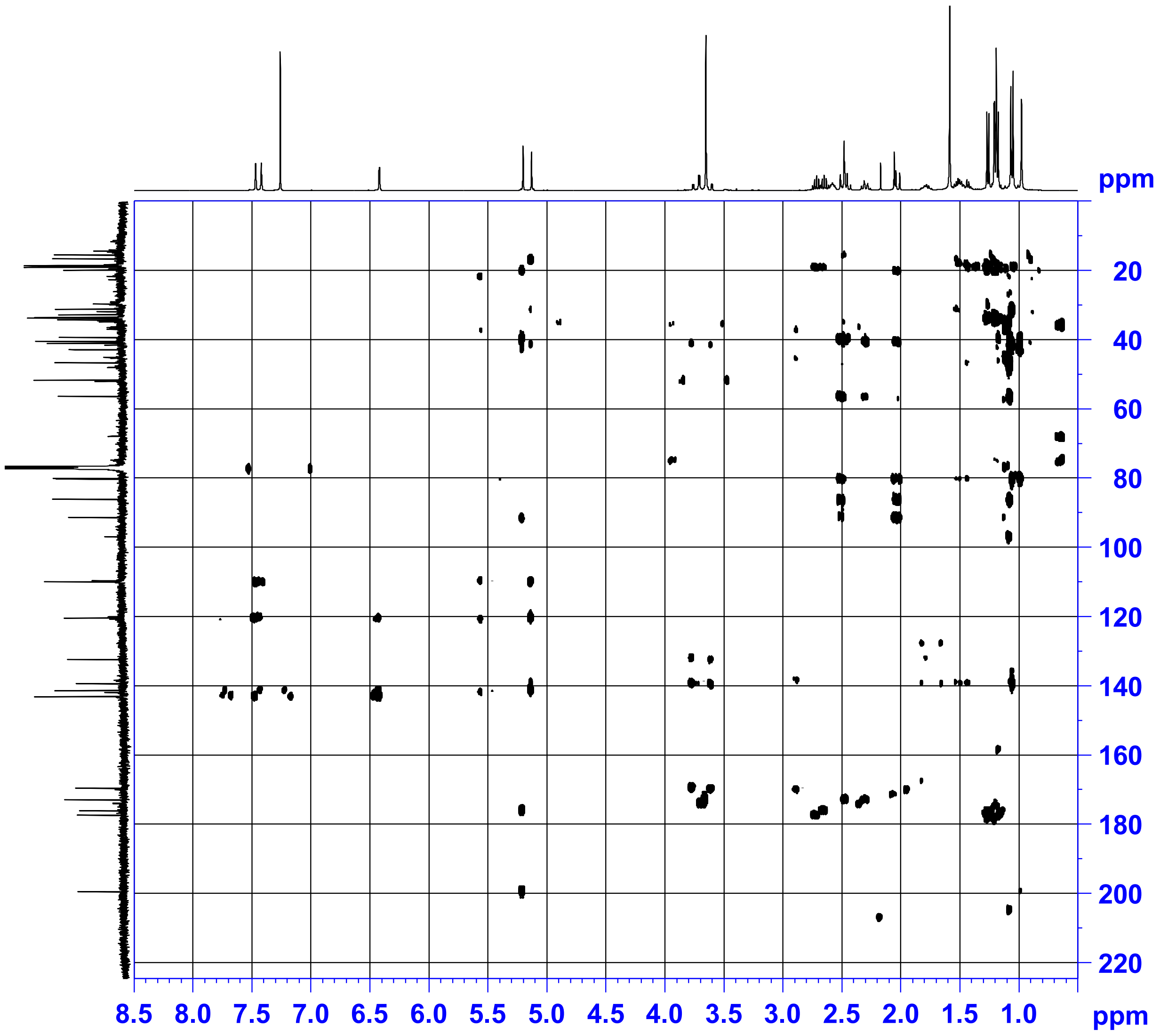

NAME	ZQ-24-1
EXPNO	6
PROCNO	1
Date_	20170401
Time	11.01
INSTRUM	spect
PROBHD	5 mm CPPBBO BB
PULPROG	hmbcgplpndqf
TD	4096
SOLVENT	CDCl3
NS	32
DS	16
SWH	5197.505 Hz
FIDRES	1.268922 Hz
AQ	0.3940852 sec
RG	208.5
DW	96.200 usec
DE	10.00 usec
TE	297.0 K
CNST2	145.0000000
CNST13	10.0000000
D0	0.00000300 sec
D1	1.50000000 sec
D2	0.00344828 sec
D6	0.05000000 sec
D16	0.00020000 sec
IN0	0.00002080 sec
======== CHANNEL f1 ========	
SFO1	400.1323208 MHz
NUC1	1H
P1	11.50 usec
P2	23.00 usec
ND0	2
TD	128
SFO1	100.6233 MHz
FIDRES	187.800476 Hz
SW	238.896 ppm
FnMODE	QF
SI	2048
SF	400.1300050 MHz
WDW	SINE
SSB	0
LB	0.00 Hz
GB	0
PC	1.40
SI	1024
MC2	QF
SF	100.6127593 MHz
WDW	SINE
SSB	0
LB	0.00 Hz
GB	0

HMBC spectrum of Krishnolide D (**4**) in $CDCl_3$

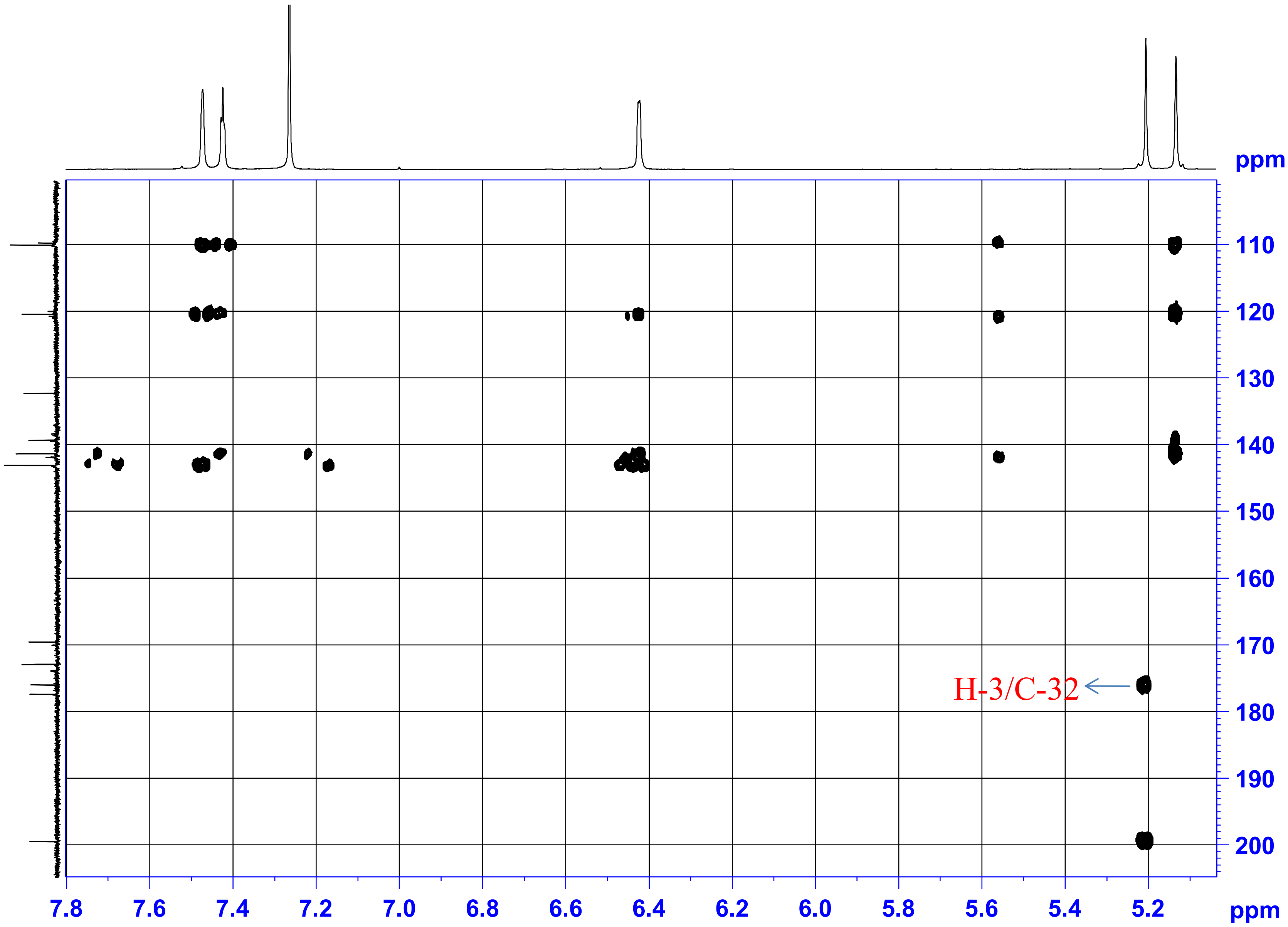

HMBC spectrum of Krishnolide D (**4**) in $CDCl_3$

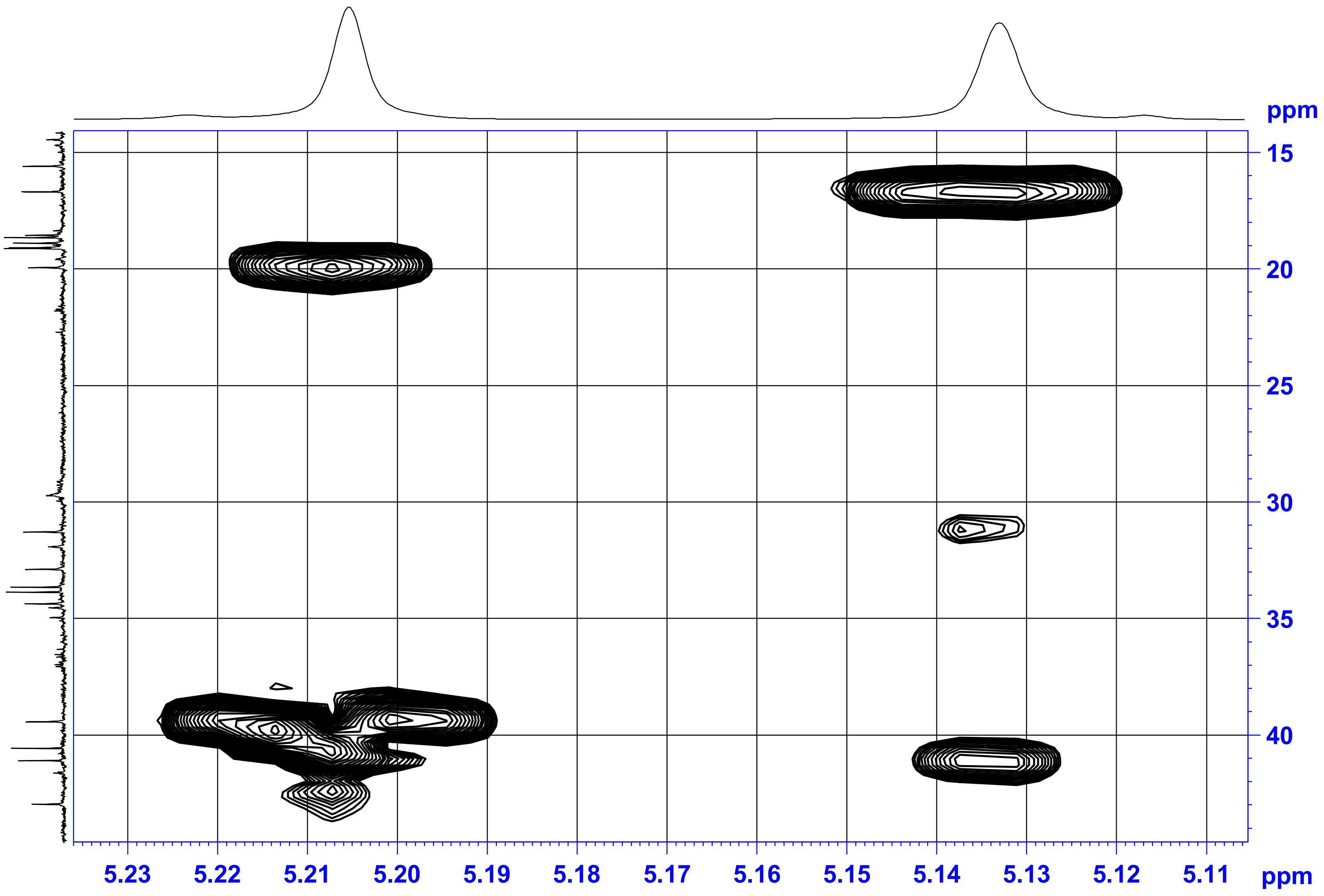

HMBC spectrum of Krishnolide D (**4**) in $CDCl_3$

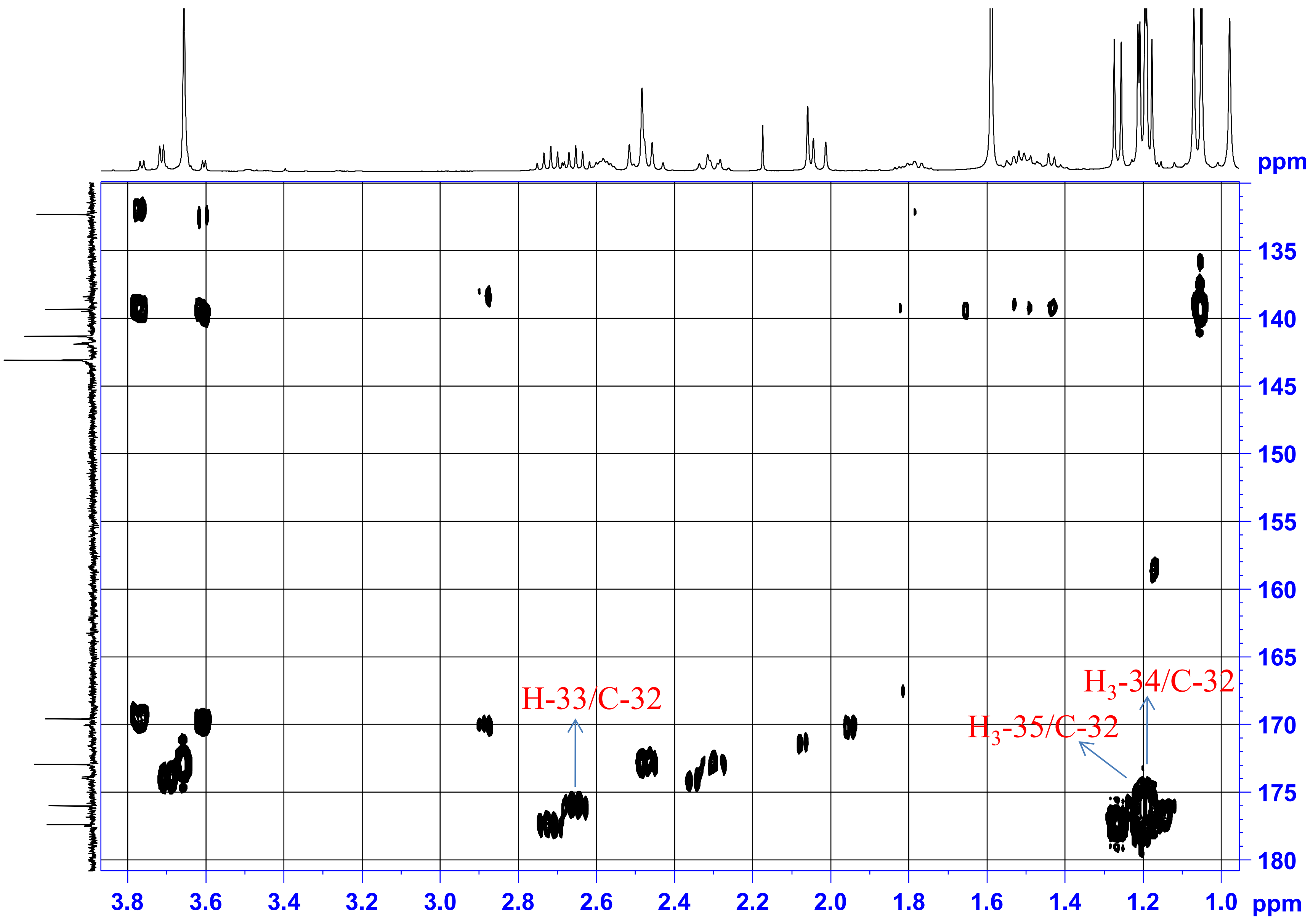

HMBC spectrum of Krishnolide D (**4**) in $CDCl_3$

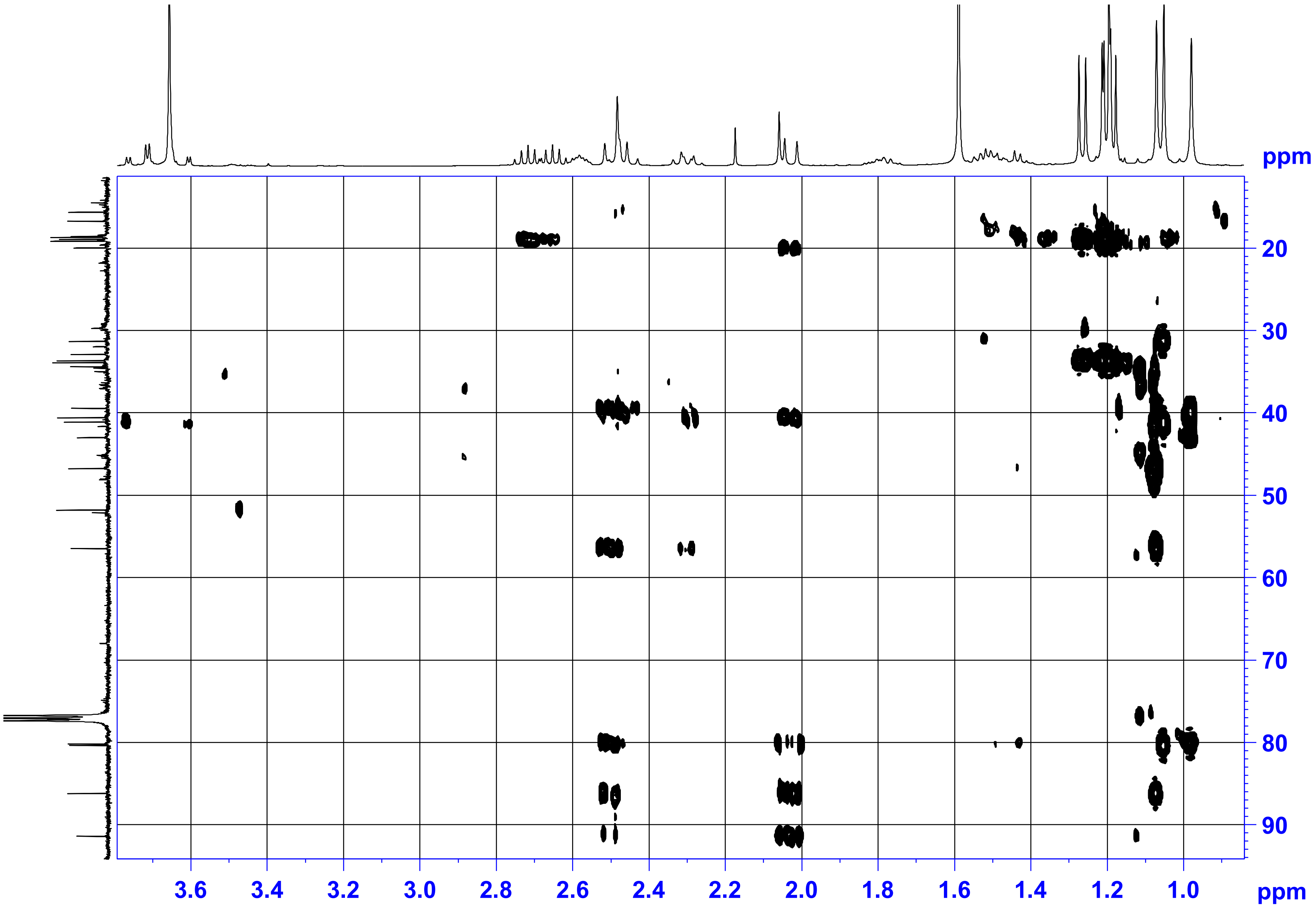

HMBC spectrum of Krishnolide D (**4**) in $CDCl_3$

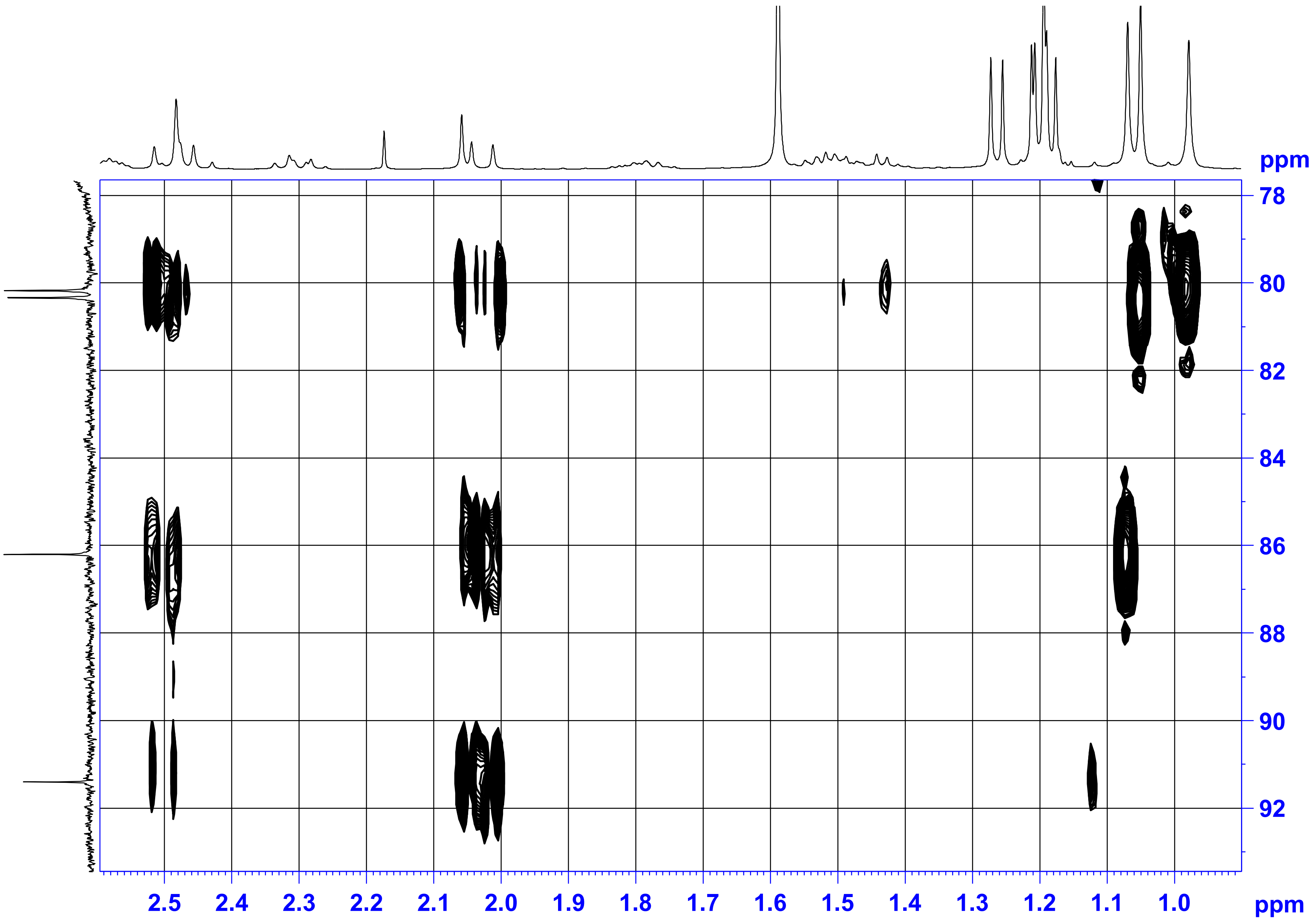

HMBC spectrum of Krishnolide D (**4**) in $CDCl_3$

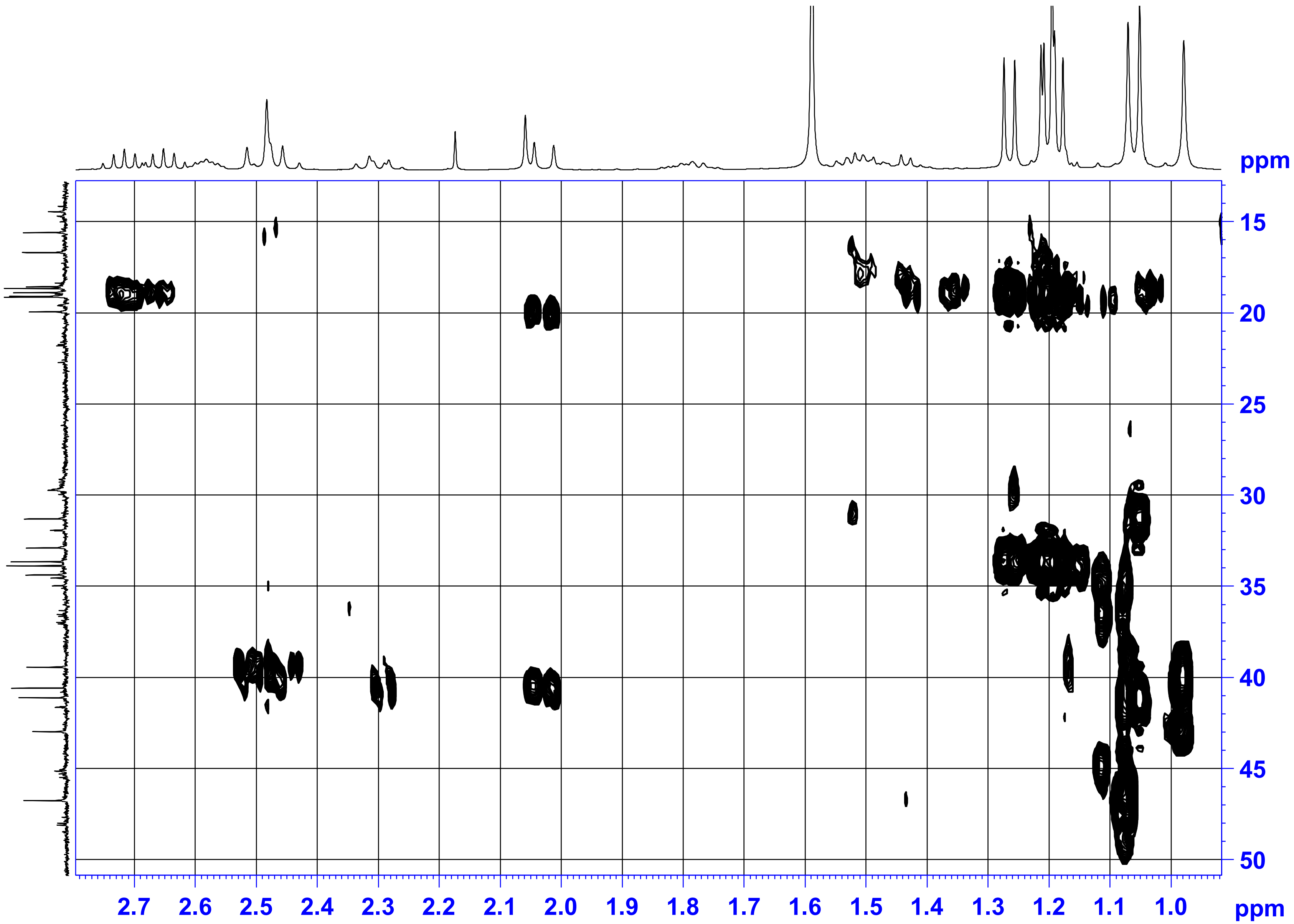

HMBC spectrum of Krishnolide D (**4**) in $CDCl_3$

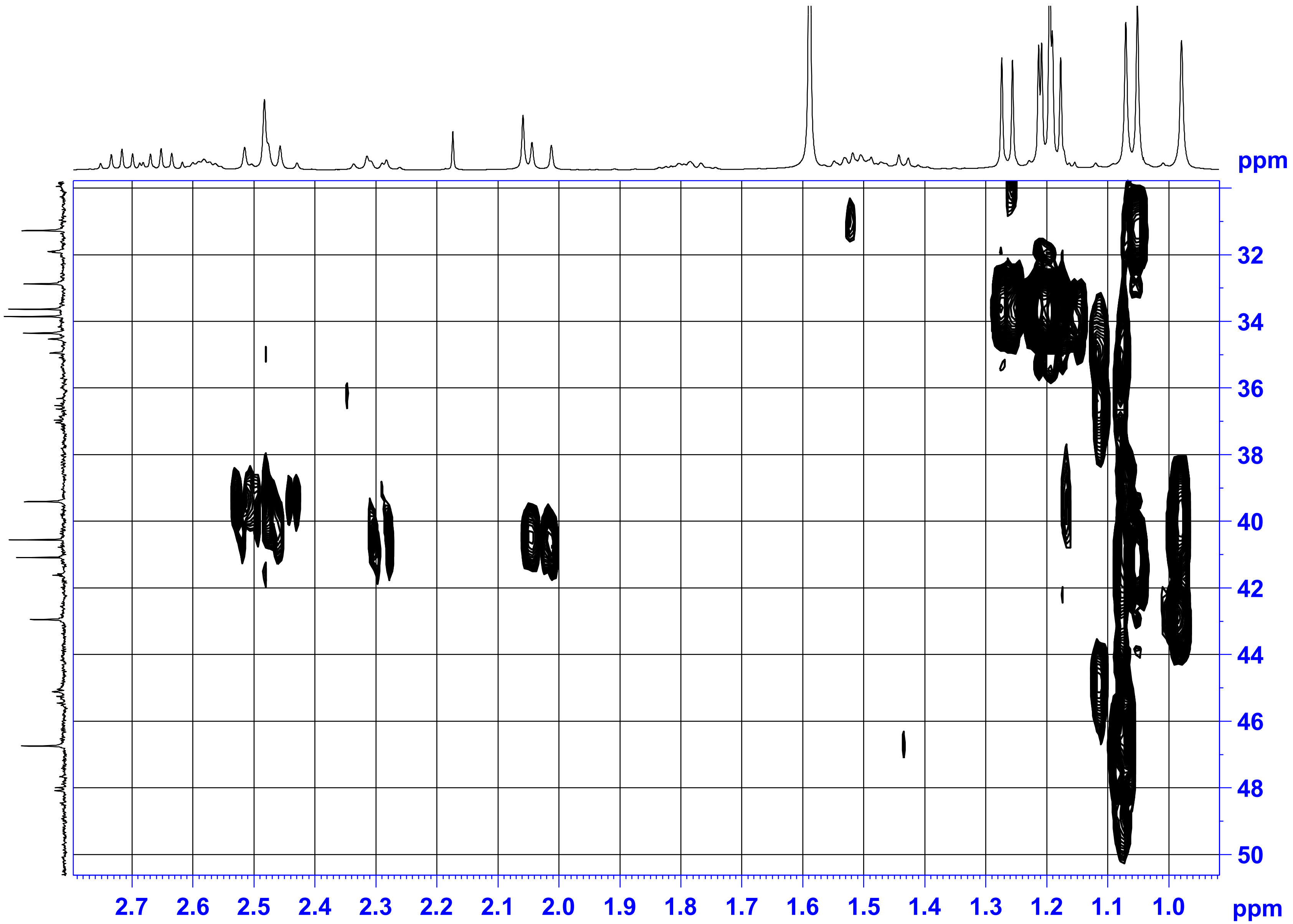

HMBC spectrum of Krishnolide D (**4**) in $CDCl_3$

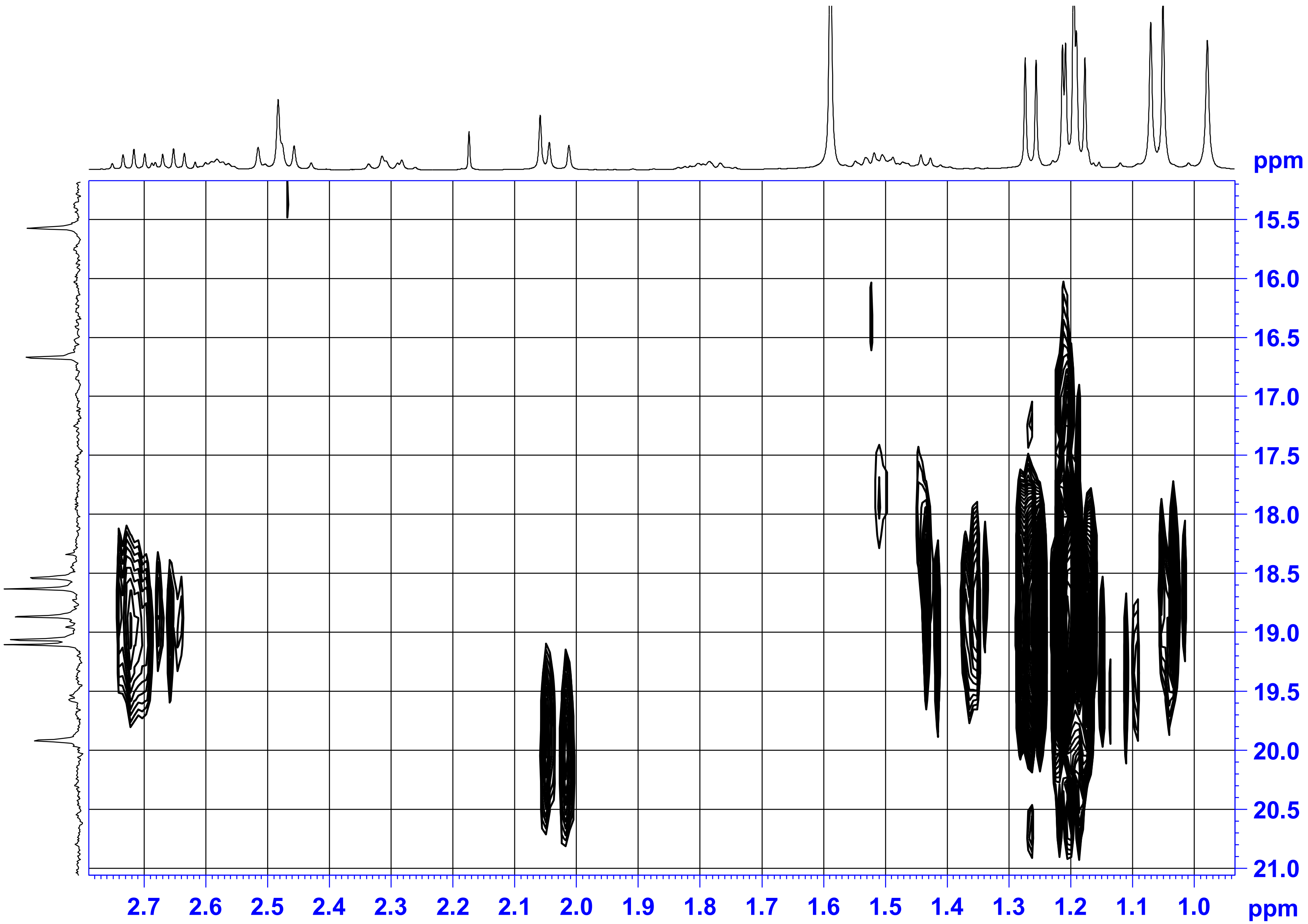

NOESY spectrum of Krishnolide D (**4**) in $CDCl_3$

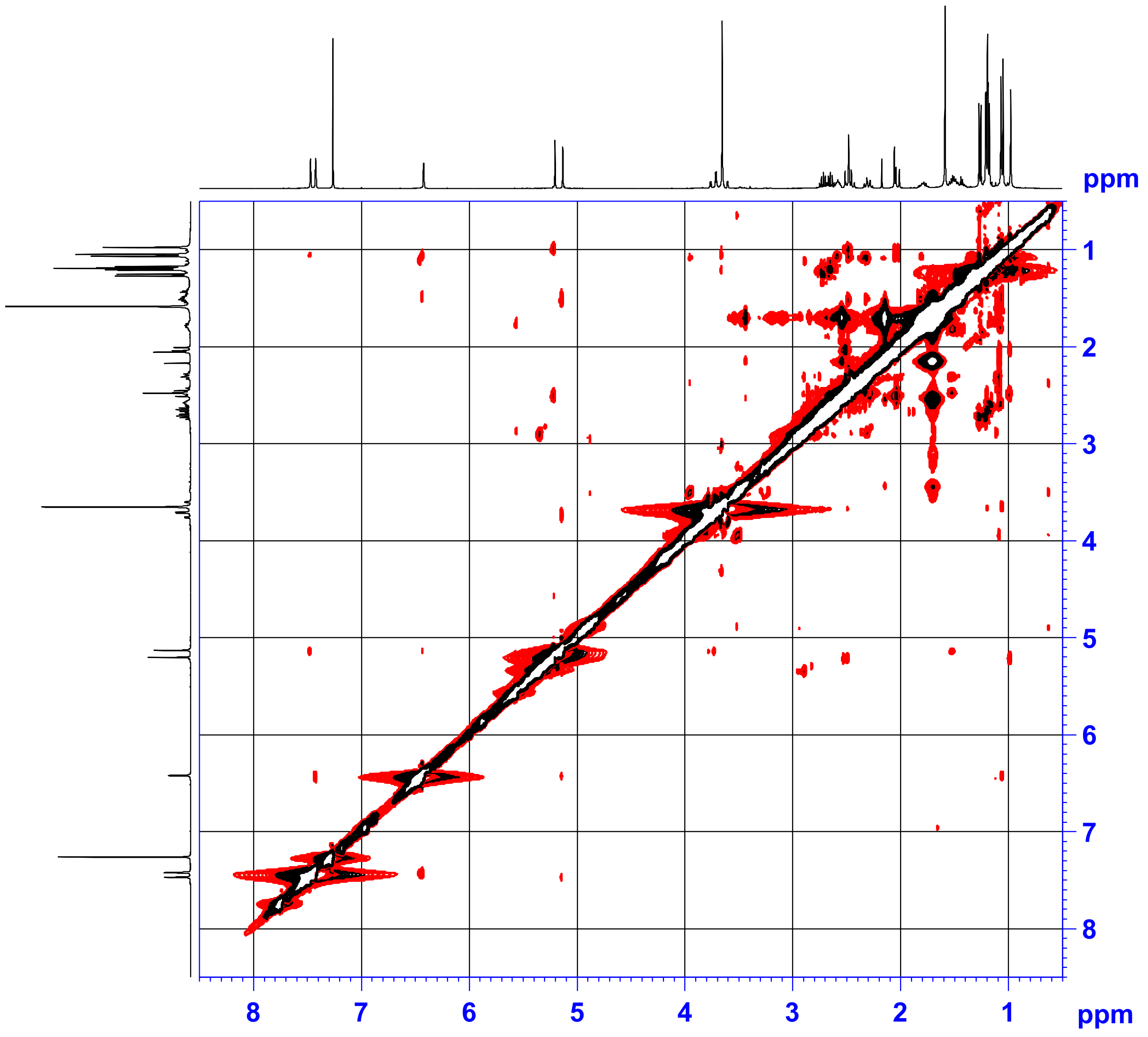

NAME	ZQ-24-1	
EXPNO	7	
PROCNO	1	
Date_	20170401	
Time	13.17	
INSTRUM	spect	
PROBHD	5 mm CPPBBO BB	
PULPROG	noesygpphpp	
TD	2048	
SOLVENT	CDCl3	
NS	16	
DS	32	
SWH	4000.000	Hz
FIDRES	1.953125	Hz
AQ	0.2560500	sec
RG	208.5	
DW	125.000	usec
DE	10.00	usec
TE	297.0	K
D0	0.00011036	sec
D1	1.99385595	sec
D8	0.30000001	sec
D11	0.03000000	sec
D12	0.00002000	sec
D16	0.00020000	sec
IN0	0.00025000	sec
======== CHANNEL f1 ========		
SFO1	400.1318006	MHz
NUC1	1H	
P1	11.50	usec
P2	23.00	usec
P17	2500.00	usec
ND0	1	
TD	256	
SFO1	400.1318	MHz
FIDRES	15.625000	Hz
SW	9.997	ppm
FnMODE	States-TPPI	
SI	1024	
SF	400.1300050	MHz
WDW	QSINE	
SSB	2	
LB	0.00	Hz
GB	0	
PC	1.00	
SI	1024	
MC2	States-TPPI	
SF	400.1300050	MHz
WDW	QSINE	
SSB	2	
LB	0.00	Hz
GB	0	

NOESY spectrum of Krishnolide D (**4**) in $CDCl_3$

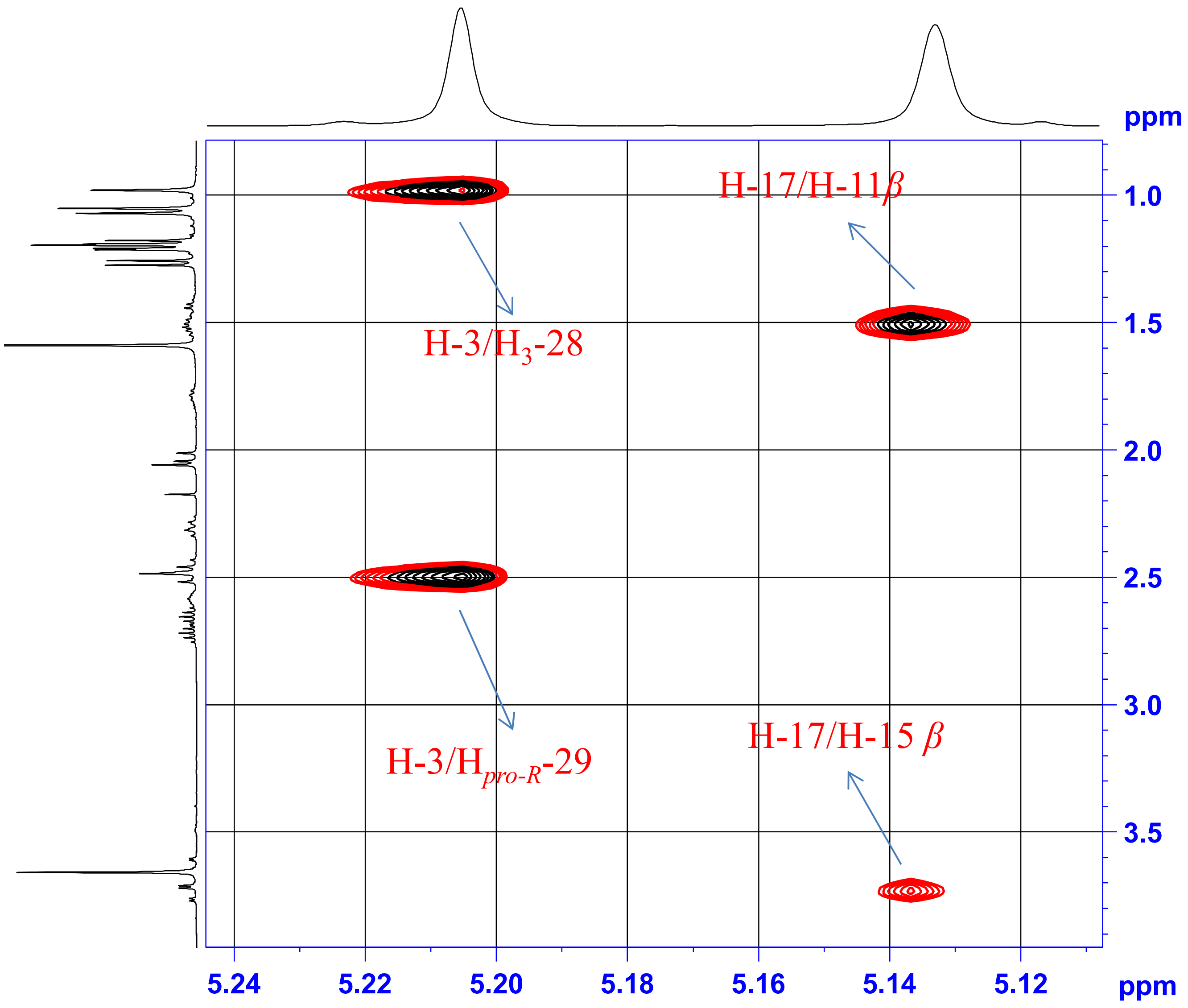

NOESY spectrum of Krishnolide D (**4**) in $CDCl_3$

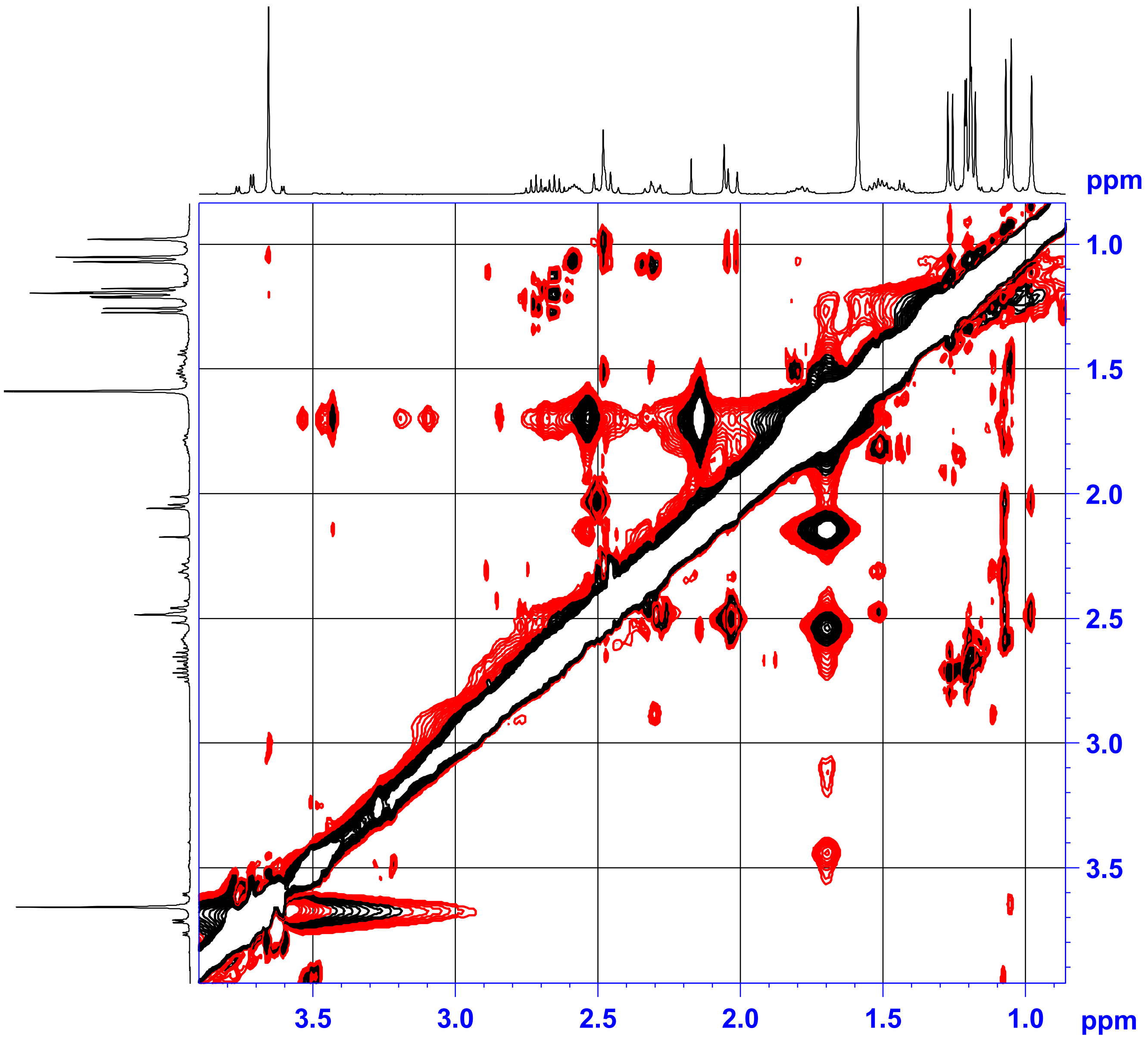

NOESY spectrum of Krishnolide D (**4**) in $CDCl_3$

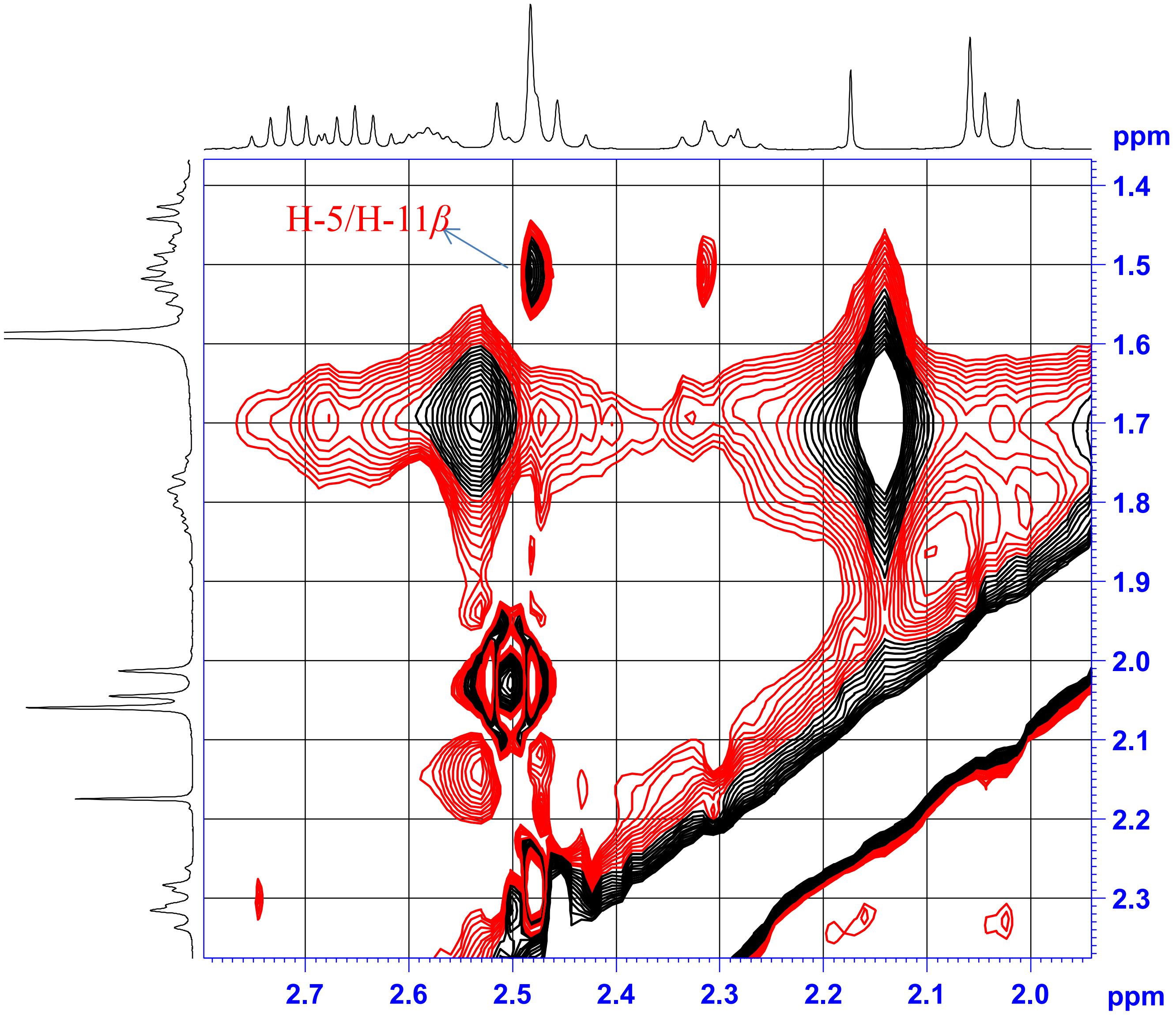

NOESY spectrum of Krishnolide D (**4**) in $CDCl_3$

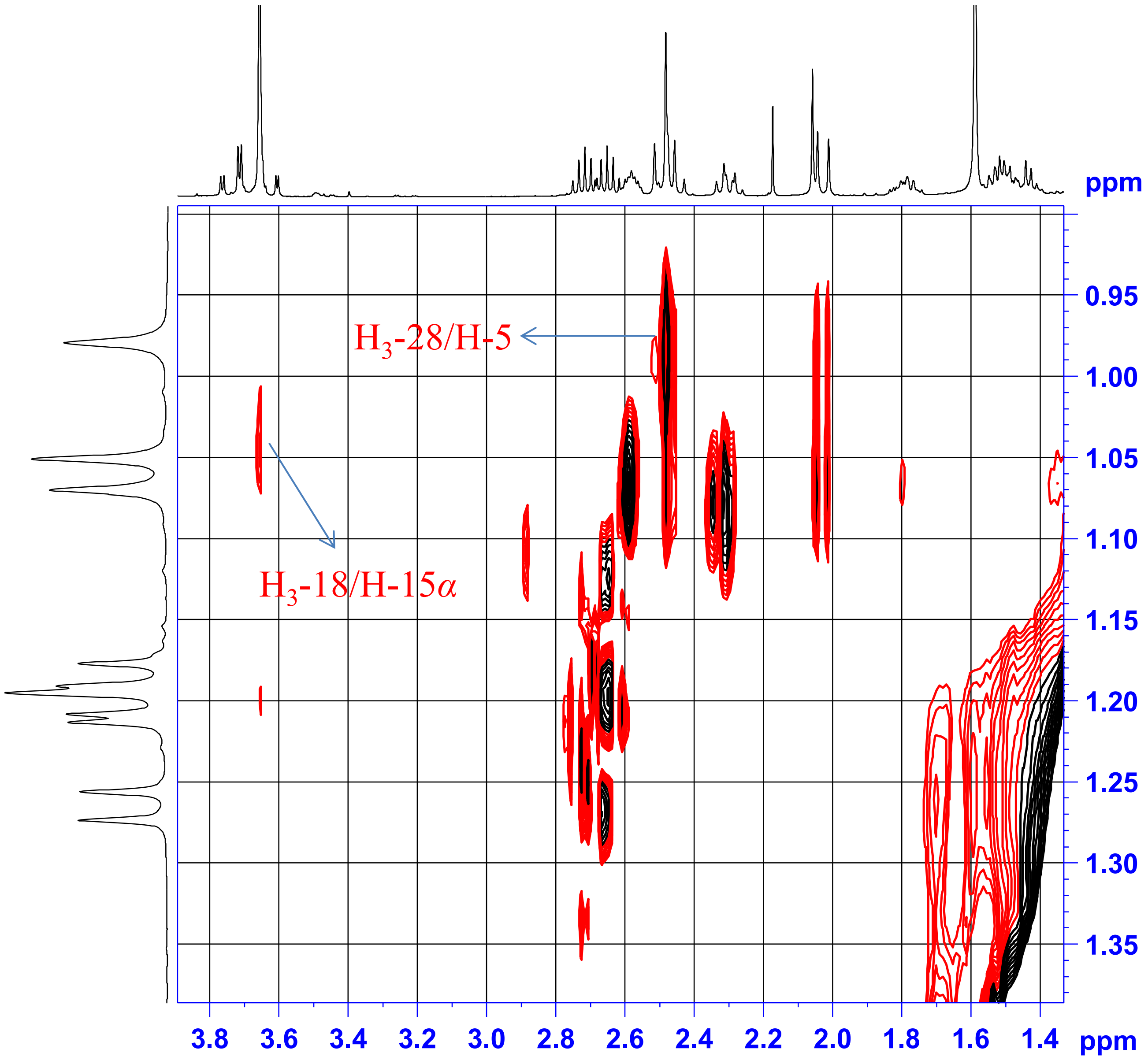

NOESY spectrum of Krishnolide D (**4**) in $CDCl_3$

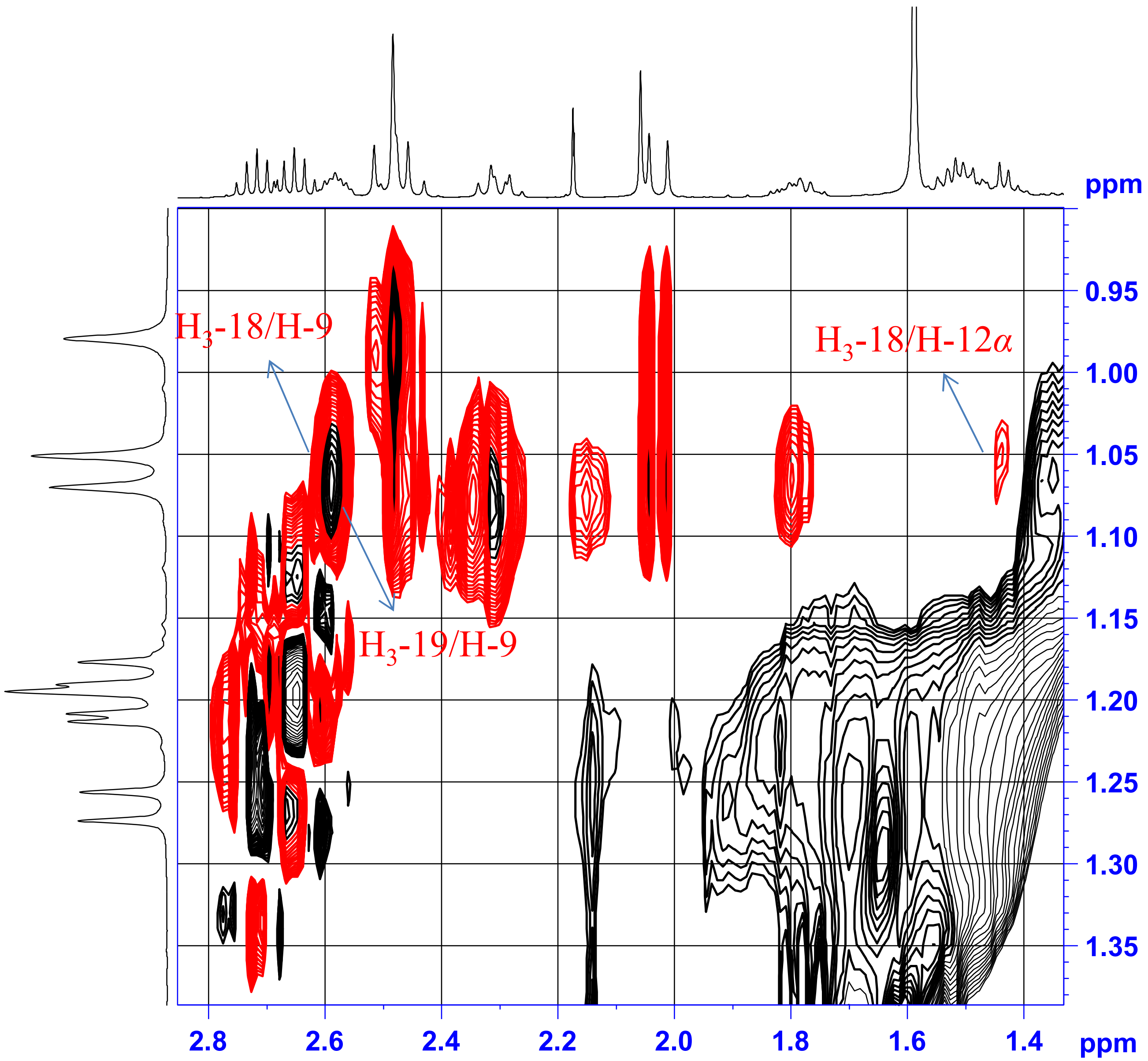

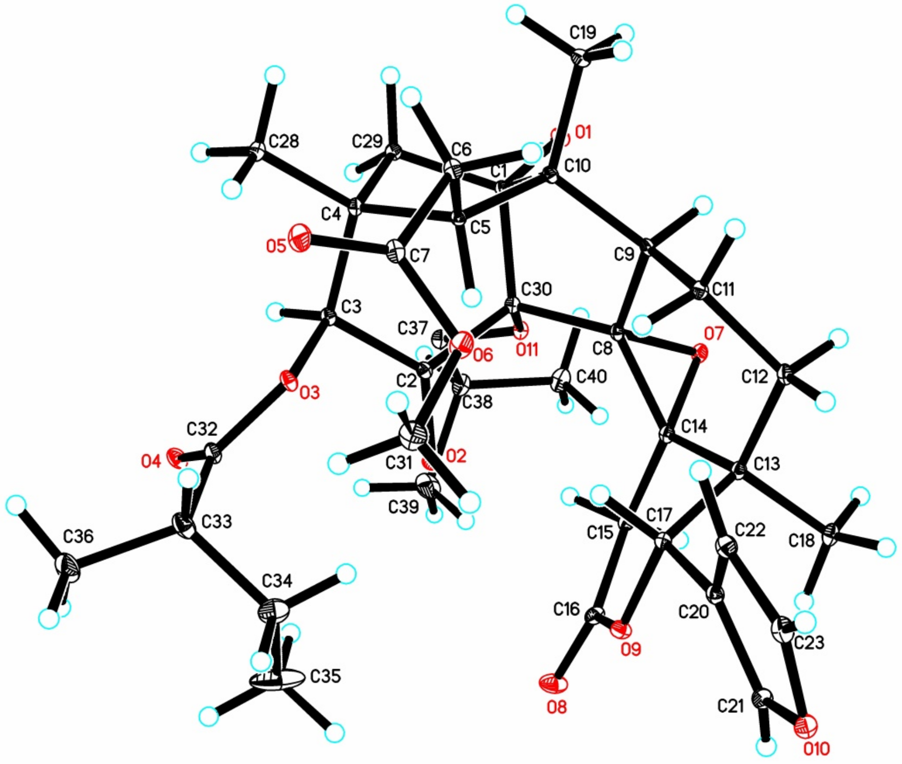

Figure S1. ORTEP illustration of the X-ray structure of Krishnolide A (**1**) . Ellipsoids are given at the 30% probability level.
